# Tolyporphins–Exotic Tetrapyrrole Pigments in a Cyanobacterium—A Review

**DOI:** 10.3390/molecules28166132

**Published:** 2023-08-18

**Authors:** Kathy-Uyen Nguyen, Yunlong Zhang, Qihui Liu, Ran Zhang, Xiaohe Jin, Masahiko Taniguchi, Eric S. Miller, Jonathan S. Lindsey

**Affiliations:** 1 Department of Chemistry, North Carolina State University, Raleigh, NC 27695-8204, USA; uvnguyen@ncsu.edu (K.-U.N.); yzhang95@alumni.ncsu.edu (Y.Z.); qliu26@ncsu.edu (Q.L.); rzhang0915@gmail.com (R.Z.); xjin4@ncsu.edu (X.J.); mtanigu@ncsu.edu (M.T.); 2 Department of Plant and Microbial Biology, North Carolina State University, Raleigh, NC 27695-7612, USA; esm@ncsu.edu

**Keywords:** bacteriochlorin, bioinformatics, biosynthesis, chlorophyll, cyanobacteria, field biology, gene cluster, genomics, natural products, tolyporphins

## Abstract

Tolyporphins were discovered some 30 years ago as part of a global search for antineoplastic compounds from cyanobacteria. To date, the culture HT-58-2, comprised of a cyanobacterium–microbial consortium, is the sole known producer of tolyporphins. Eighteen tolyporphins are now known—each is a free base tetrapyrrole macrocycle with a dioxobacteriochlorin (14), oxochlorin (3), or porphyrin (1) chromophore. Each compound displays two, three, or four open β-pyrrole positions and two, one, or zero appended *C*-glycoside (or –OH or –OAc) groups, respectively; the appended groups form part of a geminal disubstitution motif flanking the oxo moiety in the pyrroline ring. The distinct structures and repertoire of tolyporphins stand alone in the large pigments-of-life family. Efforts to understand the cyanobacterial origin, biosynthetic pathways, structural diversity, physiological roles, and potential pharmacological properties of tolyporphins have attracted a broad spectrum of researchers from diverse scientific areas. The identification of putative biosynthetic gene clusters in the HT-58-2 cyanobacterial genome and accompanying studies suggest a new biosynthetic paradigm in the tetrapyrrole arena. The present review provides a comprehensive treatment of the rich science concerning tolyporphins.

*“To know well the full biodiversity of Earth is not important simply to add figures to textbooks. The real purpose of science must be the original Linnaean goal: to find and take full account of each and every species of organism on Earth”.*

E. O. Wilson [1]

## 1. Introduction to a Singular Discovery

**Figure 1:**
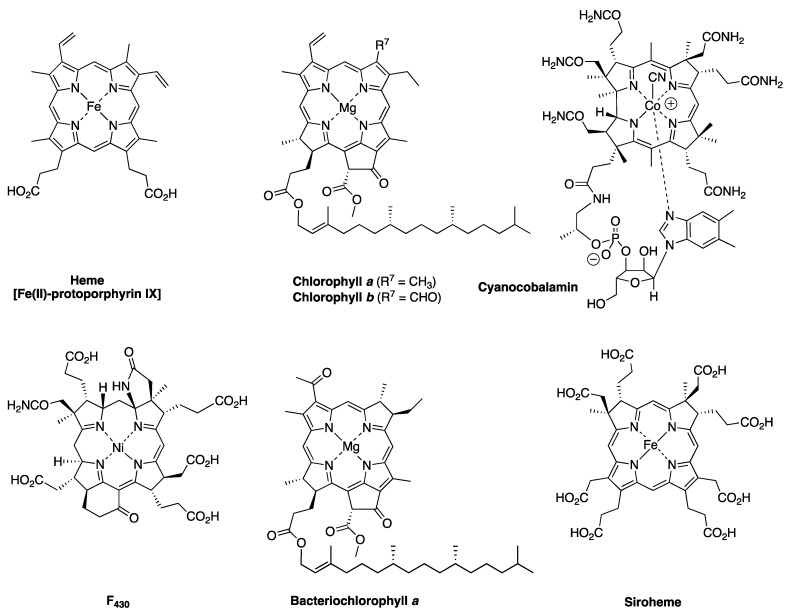
Representative members of the pigments-of-life family.

Naturally occurring tetrapyrroles are often referred to as the pigments of life [2,3,4]. The family includes the venerable heme and chlorophylls, the source of coloring of blood and plants, respectively; bacteriochlorophylls, the pigments of anoxygenic photosynthesis; the majestic cobalamin (i.e., vitamin B_12_), the intrinsic factor for pernicious anemia; and F_430_, an enzymatic cofactor involved in anaerobic methanogenesis. F_430_ was discovered in the 1970s [5]. Otherwise, the major pillars of the family of natural tetrapyrroles were probably regarded as largely in place, if not complete, toward the end of the 20th century, especially given the immense attention devoted to this class of compounds. The structures of selected members of the pigments of life are shown in Figure 1. For example, as of 1990, the most recent newly discovered chlorophyll molecule (other than variants in marine photosynthetic bacteria, such as divinyl chlorophylls) was chlorophyll *d*, which was found in a cyanobacterium in 1943 [6,7]. In 2010, chlorophyll *f* was discovered in a stromatolite cyanobacterial community on the coast of Australia [8]. Each discovery of a new member of the tetrapyrrole family prompts a host of questions, ranging from properties and function to biosynthesis and evolution [9,10]. Yet, the occasional surfacing of a new tetrapyrrole variant, such as divinyl chlorophylls or chlorophyll *f*, has been readily accommodated by the existing paradigms for biosynthesis and biological function.

Cyanobacteria are prokaryotes, yet contain an oxygen-evolving photosynthetic apparatus similar to that of plants [11], are readily adaptable to diverse habitats [12], and are known to be extraordinarily rich repositories of secondary metabolites [13,14,15,16]. The global sweep of cyanobacteria has been known for decades, as described in 1952 by Allen [17]: “The *Myxophyceae*, or *blue-green algae*, are notable for their ability to develop in an extremely wide range of habitats. They are the first organisms to colonize sterile volcanic soils, yet they abound in polluted waters; they develop in masses on the icy deserts of Central Asia and the mountaintops of Japan, and they also develop in masses in the waters of hot springs, at the highest temperatures reported for any living organisms. Many live in association with other organisms, with fungi in lichens, with protozoa in *Cyanophora*, even within the roots of *Cycads* and the rhizomes of *Gunnera*”.

**Figure 2:**
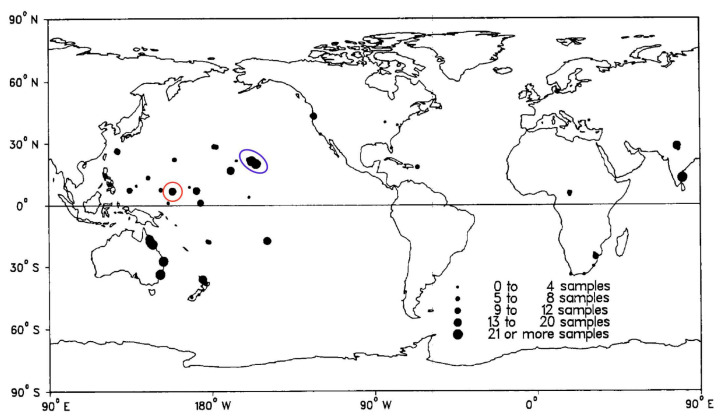
Search for cyanobacteria. The red circle identifies Pohnpei; the blue oval encompasses the Hawaiian islands. Adapted with permission from ref [19]; 1991, John Wiley and Sons, Inc.

Beginning in 1981, a team of researchers led by Richard Moore and Gregory Patterson carried out a worldwide search for new antineoplastic agents derived from cyanobacteria [18]. Given the home base of Moore and Patterson at the University of Hawaiʻi, much of their Linnaean search took place across islands and other regions in the Pacific Ocean [19] (Figure 2).

The team of Moore and Patterson ultimately collected several thousand specimens. The Hawaiʻi team [18] “cultivated and examined extracts from in excess of 1500 strains representing some 400 species of blue-green algae. The strains selected for screening originated from freshwater, terrestrial and marine environments and were chosen without regard to any biological activity of the source (field) material”. In the program [18], “organisms selected for screening were chosen to provide a broad representation of the blue-green algae, both taxonomically and ecologically. Cultures of approximately 20-liter volume were grown to provide cell material from which crude lipophilic and hydrophilic extracts were prepared. The extracts were tested immediately for bioactivity; portions were then archived at low temperature to provide a ‘library’ of extracts for future testing”. From such studies, the extract from one culture (HT-58-2) was identified to reverse multidrug resistance in a cultured human adenocarcinoma cell line [20,21].

**Figure 3:**
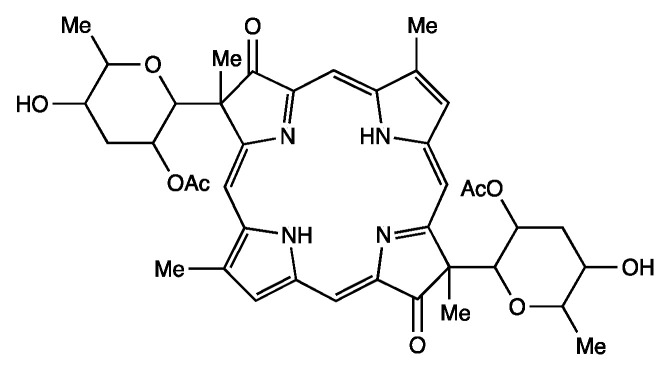
Tolyporphin A from Prinsep et al. [20].

The active compound was found to be tolyporphin A (C_40_H_46_N_4_O_10_, 742.8 Da), a previously unknown tetrapyrrole macrocycle. The structure reported is shown in Figure 3. Tolyporphin A (and the ensuing family of tolyporphins) was not the only major finding from the Moore–Patterson Hawaiʻi collection of cyanobacteria [22]—“in screening extracts of over 1000 blue-green algae (cyanobacteria)”, the peptolide family of cryptophicins also was discovered [22,23]. The Moore–Patterson Hawaiʻi collection remains under investigation for new natural products [24]. The focus here, however, is restricted to tolyporphins. The process of screening lipophilic extracts via biological assays to identify active compounds is described in greater depth in Section 17 concerning the possible pharmacological properties of tolyporphins.

**Figure 4:**
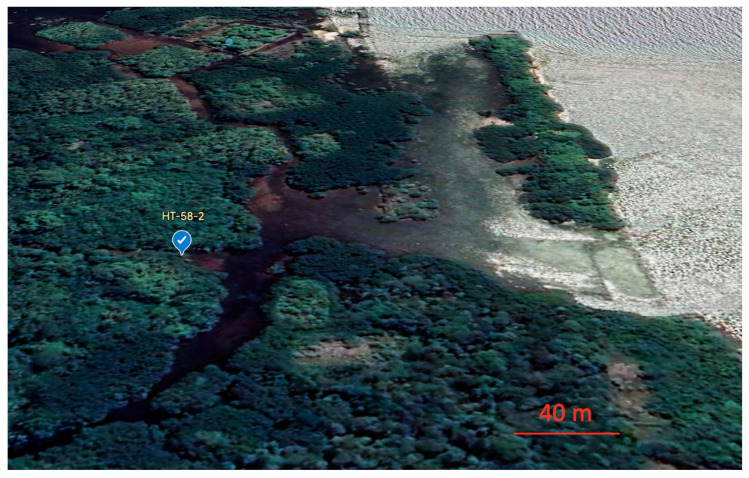
Approximate collection site of HT-58-2 (6 50.33 N, 158 19.57 E); on Temwen Island in the administrative district of Madolenihmw in Pohnpei. Architectural ruins are visible at the top of the image. Images created via Google Earth with permission.

The isolate HT-58-2 was obtained from a soil sample collected in February 1989 in Nan Madol, Pohnpei, an island in Micronesia. A convenient geolocator on a two-dimensional map is that Pohnpei, Hawaiʻi, and southern California form a syzygy, with Hawaiʻi roughly at the midpoint (Figure 2). The indigenous name, Pohnpei, means “upon a stone altar” [25]. Indeed, “Nan Madol–Pohnpei’s spectacular ancient city” [26] contains sprawling megalithic ruins, completed around 1200–1600 AD [26], that may rival those of Central America [27]. Key distinctions being, of course, that Nan Madol is located on a jungle island remote from major continents and population centers, and some of the ruins are partially submerged. The soil sample was collected on the southeast side of the island of Pohnpei, approximately 250 m from the shoreline, near the ruins of Nan Madol (Figure 4). Information concerning the discovery, labeling, and early culture of HT-58-2 is provided in the Supplementary Materials (Section S1; Figures S1–S3).

The biological classification of the various isolates was performed chiefly on the basis of morphology, as was typical in that era. The isolate HT-58-2 was assigned *Tolypothrix nodosa* [20]. The term *Tolypothrix* derives from the Greek words *tolype*, meaning ball of yarn, and *thrix*, meaning hair, and connotes the filamentous cyanobacterium. The term *nodosa* refers to the presence of nodules, thought to be sites (heterocysts) for nitrogen fixation. Given the tetrapyrrole framework of the identified active compound, there followed the term tolyporphin—a portmanteau of *Tolypothrix* and porphyrin, referring to the genus and the macrocycle. It turns out that tolyporphin is a doubly flawed portmanteau, however, because the tolyporphins are generally not porphyrins sensu stricto, and subsequent genomic information likely points to a different genus—*Brasilonema* rather than *Tolypothrix*. One can argue that *T. nodosa* might be renamed, but the names of organic compounds are rarely changed, no matter how flawed. The challenges in cyanobacterial taxonomy are longstanding and well known [28]. Many terms borne in a rivulet of one era float along as the river of science grows inexorably deeper and wider.

Tolyporphins have been noted in a number of reviews [29,30,31,32,33,34] as structural curiosities of unusual provenance. The present review article provides a comprehensive account concerning all aspects of the tolyporphins family reported by a large number of researchers over the past 30 years.

## 2. Structure and Diversity of Tolyporphins

**Figure 5:**
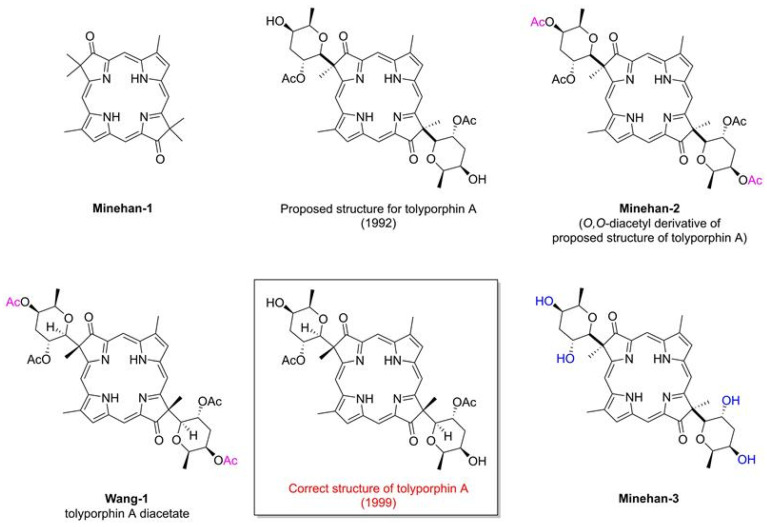
Structures at the outset of the tolyporphins saga.

The field of tolyporphins studies was initiated by the Moore research group [20], who proposed an initial structure for tolyporphin A and reported results concerning biological activity [20]. The chemical synthesis was pursued by Yoshito Kishi (1937–2023) [35,36,37] and coworkers beginning in 1992, with reports emerging in 1997–1999. The first report, by Minehan and Kishi [38], described the synthesis of the core skeleton of tolyporphin A (here termed **Minehan-1** for clarity), sans glycosides, but bearing a gem-dimethyl group in each pyrroline unit. The macrocycle was constructed by extension of methodology established by Eschenmoser during the synthesis of cobalamin (Figure 5). The second paper investigated strategies for *C*-glycosidation [39]. The third paper reported the remarkable total synthesis of the *O*,*O*-diacetyl derivative of the proposed structure of the natural product tolyporphin A, termed tolyporphin diacetate (**Minehan-2**), only to find a key discrepancy between the synthetic and native substances [40]. Evidence in this regard was achieved by (1) *O*,*O*-diacetylation of native tolyporphin A, which yielded a diastereomer of **Minehan-2** (not shown) and (2) tetra-deacetylation of **Minehan-2** to form the tetra-alcohol **Minehan-3**. The tetra-alcohol **Minehan-3** also was a diastereomer of the tetra-alcohol derived by di-deacetylation of native tolyporphin A. (The di-deacetylated native tolyporphin A is identical with native tolyporphin D, as described in the next few paragraphs.) In short, the discrepancy between the synthetic tolyporphin **Minehan-2** (created on the basis of the proposed structure for tolyporphin A) and the actual *O*,*O*-diacetyl derivative of native tolyporphin A (isolated from HT-58-2) indicated that the proposed structure was in error with regards to stereochemical configuration.

The fourth paper reported structural and spectroscopic studies of derivatives of native tolyporphin A, along with analogous synthetic compounds (including **Minehan 2**) [41]. The correct structure of native tolyporphin A was identified [41] to be as shown in Figure 5. In short, the original proposal [20] put both *C*-glycoside groups on the β-face of the macrocycle, whereas the revised structure positioned both *C*-glycoside groups on the α-face of the macrocycle [41,42]. In the proposed and correct structure of tolyporphin A shown in Figure 5, the β-face is presented to the viewer, whereas the α-face is away from the viewer. A description of nomenclature for heterotopic ligands is provided by Eliel and Wilen [43]. The core tetrapyrrole scaffold (apart from the stereochemical configuration of substituents in the pyrroline units) was identical in the original and revised assignments.

In the fifth and final paper on the topic (entitled “Synthesis and Structure of Tolyporphin A *O*,*O*-Diacetate”), the compound **Wang-1**, having the same stereochemical configuration as authentic native tolyporphin A, was synthesized by Wang and Kishi (Figure 5) [42]. We note that the appellation tolyporphin A *O*,*O*-diacetate is disfavored (because “acetate” already implies the “O” atom attachment); accordingly, hereafter, the equivalent terms tolyporphin A diacetate and *O,O*-diacetyl tolyporphin A are employed. A comprehensive review of the synthesis of tolyporphin A diacetate has been reported [44]. While tolyporphin A can be converted to tolyporphin A diacetate by diacetylation, the reverse may be difficult, given that tolyporphin A itself contains two *O*-acetyl groups, and methods for selective removal of two of the four *O*-acetyl groups have not been demonstrated. Regardless, the towering chemical synthesis of Minehan, Wang, and Kishi has incontrovertibly established the molecular structure of native tolyporphin A.

**Figure 6:**
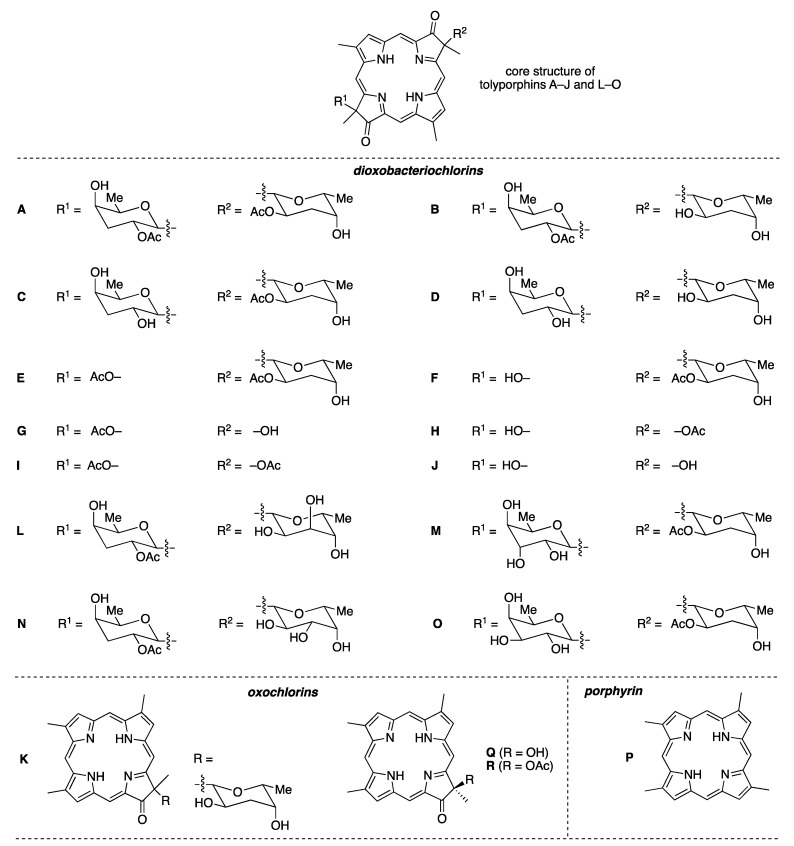
Known universe of natural tolyporphins.

Ongoing studies of the HT-58-2 culture with application of careful fractionation methods revealed a collection of tolyporphin analogues. Prinsep isolated and identified tolyporphins B–I [45], J and K [46], and L and M [47]. Subsequent studies by Williams and coworkers isolated and identified tolyporphins N–R [48]. The molecular structures of tolyporphins B–R, on the basis of the most recent findings, are provided in Figure 6. Caution is exercised concerning structures because several compounds were co-isolated. The co-isolates include compounds B + C [45]; G + H [45]; and L + M [47]. All of the structures displayed in Figure 6 have been rotated by 180° about the horizontal axis of tolyporphin A, shown in Figure 5, whereupon the *C*-glycosides are on the front face of the macrocycle and project toward the viewer. The rationale for the display shown in Figure 6 is to comport with the traditional display of chlorophylls and bacteriochlorophylls, as described in the next paragraphs. The structural displays in Figure 5 and Figure 6 are revisited upon consideration of the biosynthesis of tolyporphins and other native tetrapyrrole structures (see Section 15 and Section 16).

In general, the structural assignments of tolyporphins beyond A have been derived on the basis of mass spectrometry, NMR spectroscopy, and absorption spectroscopy. The stereochemistry has been confirmed for tolyporphins A, E, and R by single-crystal X-ray structure analysis [48], whereas that for the other tolyporphins remains undefined. Tolyporphin Q likely shares the same stereochemistry as that of tolyporphin R [48]. At a time when the known inventory of tolyporphins included members A–K, Wang and Kishi cautiously conjectured that the stereochemistry of tolyporphin A may be a common feature across the tolyporphins family [42]. More recent studies that show different stereochemistry [48] prompted the statement that [48] “configurational assignments based on a presumed shared biosynthesis must be done with caution, particularly for structures with a high degree of novelty arising from uncharacterized biosynthetic pathways”.

**Figure 7:**
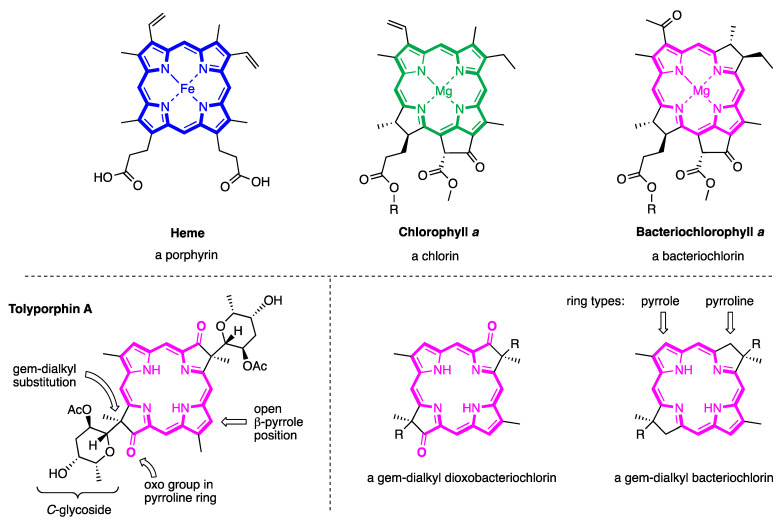
Porphyrin, chlorin, and bacteriochlorin chromophores (**top**). Types of bacteriochlorins (**bottom right**). Features of tolyporphin A (**bottom left**).

The known tolyporphins have a number of distinctive features in comparison with all other members of the tetrapyrrole family. The features are shown in Figure 7 and outlined as follows. These features have been articulated in part by Brückner [34].

**Figure 8:**
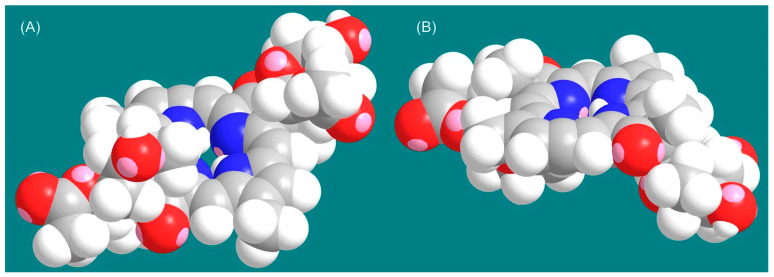
CPK diagrams showing the Janus faces of tolyporphin A [49]. (**A**) The α-face is shown, with the two *C*-glycosides pointing upward toward the viewer. (**B**) The β-face is shown, with the two methyl groups pointing upward and the two *C*-glycosides pointing downward with respect to the viewer. The two displays are interconverted by 180° rotation about the horizontal axis. Adapted with permission from ref [49]; 2017, John Wiley and Sons, Inc.

- First, tolyporphins A–J and L–O are bacteriochlorins; tolyporphins K, Q, and R are chlorins; and tolyporphin P is a porphyrin. The porphyrin, chlorin, and bacteriochlorin chromophores are highlighted using heme, chlorophyll *a*, and bacteriochlorophyll *a* as archetypes in Figure 7. The distinction between porphyrin, chlorin, and bacteriochlorin may seem simple, but the consequences are profound in terms of absorption spectra, biosynthesis, and physiological function.
- Second, the pyrroline ring (i.e., the reduced ring) in all tolyporphins (except P, which has no reduced ring) bears an oxo group and a flanking geminal disubstituted motif. Accordingly, tolyporphins A–J and L–O are dioxobacteriochlorins, and tolyporphins K, Q, and R are oxochlorins. The gem-disubstitution motif is distinct from the *trans*-dialkyl substitution motif of native photosynthetic tetrapyrroles—the members of the chlorophyll and bacteriochlorophyll family. The respective structural motifs in the pyrroline rings—gem-disubstitution versus *trans*-dialkyl—underpin a fundamental dichotomy between the two families of macrocycles.
- Third, each geminal disubstituted motif contains one methyl group and one variable entity composed of a *C*-glycoside, *O*-acetyl, or OH group. The two *C*-glycosides, at least for tolyporphins A, E, and R, are known to be positioned on the same face of the macrocycle (Figure 8) [49], affording a Janus-like structure (methyl groups on one face, *C*-glycosides on the other). A similar diagram, illustrating the location of both *C*-glycosides on the same face of the tolyporphin A macrocycle, is shown by Smith et al. [21].
- Fourth, each pyrrole ring bears one unsubstituted (i.e., open) β-position, which is exceptionally rare among natural tetrapyrroles. By contrast, heme contains vinyl, methyl, or propionic acid groups at all eight β-pyrrolic positions. Chlorophylls at the six β-pyrrolic positions contain methyl, ethyl, β-ketoester, vinyl, acetyl, and formyl substituents.
- Fifth, a metal is absent, whereas the vast majority of natural tetrapyrroles occur as the metal chelate: iron in heme, magnesium in the (bacterio)chlorophylls, cobalt in cobalamin, and nickel in F_430_.

The structural diversity among tolyporphins—encompassing the nature of the chromophore, modifications about the macrocycle periphery, and modifications of the *C*-glycoside (or OH and *O*-acetyl) groups—is reminiscent of secondary metabolites (i.e., natural products [50]) rather than products of core metabolism, given that core metabolism typically produces singular end products. Accordingly, tolyporphins are, simply put, very unusual members of the tetrapyrrole family for both their distinctiveness and diversity of structures. It is a tribute to the persistence of Prinsep and Williams and their respective research groups that the family of tolyporphins macrocycles is now known.

## 3. Resuscitation of HT-58-2

The flurry of research concerning tolyporphins and HT-58-2 in the 1990s [20,21,40,41,42,45,46,51,52] was followed by 15 years of near complete quiescence, a period of complete silence if not for the lone report concerning identification of two new tolyporphins (L and M) from HT-58-2 by use of mass spectrometry [47]. The quiescence may owe to several factors: the limited support for maintaining culture collections; the natural ebb and flow of key personnel in academic research teams (including the return by Dr. Prinsep to New Zealand following a 12-month postdoctoral stay in 1990–1991); and the unfortunate passing of Dr. Richard Moore (1933–2007) [53]. The innocuous desuetude of the Moore–Patterson collection of cyanobacteria at the University of Hawaiʻi—a veritable treasure trove for natural products discovery—led by all accounts to the loss of the precious HT-58-2 culture.

**Figure 9:**
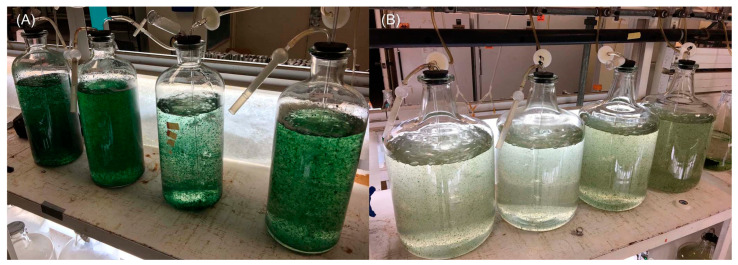
HT-58-2 in culture in the laboratory of Prof. Philip G. Williams at the University of Hawaiʻi. (**A**) 10 L flasks. (**B**) 20 L flasks.

Isolates in the Moore–Patterson collection were numbered and categorized as picked from plated environmental samples. For example, plating a soil sample designated AA-01 might produce colony isolates AA-01-1, AA-01-2, etc. Because only cyanobacteria were retained, the collection has gaps in numbering. After AA-99 was reached, the samples were designated AB-01 and so forth. The samples were traditionally stored on slants and in liquid nitrogen Dewars that were well maintained from the time of initial collection until 2003. The limited supervision and staffing from 2003 to 2010 was accompanied by inevitable emptying (or inadvertent overfill) of some of the Dewars. As of 2010, most of the strains on slants were dead, not having been passaged for years. The collection was culled to focus on the known producers of interesting natural products. Eventually, through the heroic work of Prof. Philip Williams, a sample of HT-58-2 was found—initially “misread as UT-58-2 owing to a smudged grease-pencil marker” [54]—and demonstrated to produce tolyporphins upon culturing in A3M7 medium (see the Supplementary Materials, Section S1, Figures S2 and S3). The lone sample has been the focus of subsequent studies, as described in the following sections. The culture grown at impressive scale at the University of Hawaiʻi (27 July 2017) in the laboratory of Prof. Philip G. Williams is shown in Figure 9.

## 4. Fascination with Tolyporphins

### 4.1. Gem-Dialkyl Motif in Hydroporphyrins

**Figure 10:**
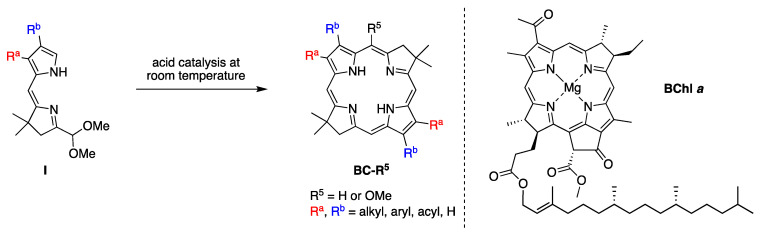
Synthesis of gem-dimethyl-substituted bacteriochlorins (**left**) [55,56] and structure of native bacteriochlorophyll *a* (**right**).

The authors’ interests in tolyporphins arose first from a sphere of research far from the main flow of natural products research. To gain facile access to synthetic analogues of bacteriochlorophylls, and thereby gain entrée to tailorable chromophores active in the near-infrared spectral region, we developed a synthesis of gem-dimethyl-substituted bacteriochlorins [55]. The route is shown in Figure 10, along with the structure of bacteriochlorophyll *a* (**BChl *a***). The route entails self-condensation of **I** under acid catalysis at room temperature to yield the bacteriochlorin **BC-R^5^** [56]. The presence of a gem-dimethyl group in each pyrroline ring precludes adventitious dehydrogenation and secures the hydroporphyrin chromophore in aerobic environments, but has a benign effect on intrinsic spectroscopic and photophysical properties. The decision to use gem-dimethyl groups stemmed from the long development of parallel chemistry *en route* to synthetic chlorins [57].

The success achieved with synthetic, gem-dimethyl-substituted bacteriochlorins nearly 20 years ago [55] prompted immediate scrutiny of the literature for the existence of any natural bacteriochlorins that contained gem-dimethyl motifs. Members of the tolyporphins family do not contain gem-dimethyl groups, but do contain a surprising gem-disubstitution motif in each pyrroline ring. The gem-disubstitution motif comprises a dialkyl (tolyporphins A–D, L–O, and K), hydroxy/alkyl (tolyporphins F–H, J, and Q), or acetoxy/alkyl (tolyporphins E, G–I, and R) moiety (Figure 6). A constant among the perhaps bewildering diversity of 18 tolyporphins is that in each case, each pyrroline ring contains methyl as one of the alkyl groups. When two alkyl groups are present, one is methyl and the other is a *C*-glycoside. The structures of tolyporphins raise questions concerning their biosynthetic pathways. The invariant presence of the aforementioned methyl group in each pyrroline ring, and the pattern of methyl groups about the perimeter of the macrocycle, which also is invariant, both enable deep insights into possible biosynthetic pathways leading to this remarkable collection of compounds.

### 4.2. Uroporphyrinogen III and Tolyporphins

**Figure 11:**
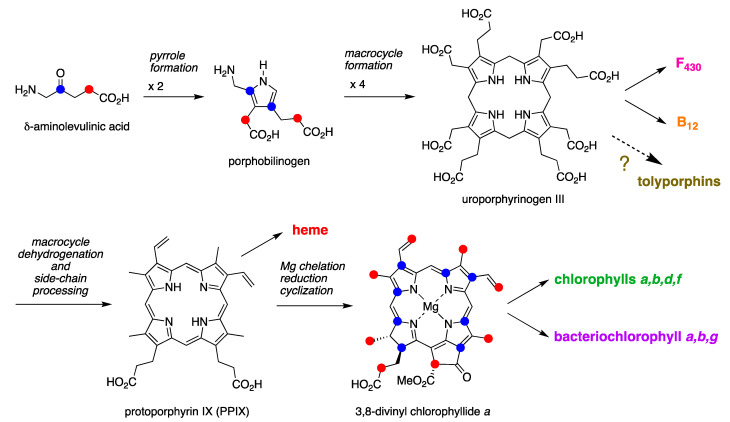
Outline of tetrapyrrole biosynthesis.

The biosynthesis of tetrapyrrole macrocycles is extraordinarily rich and affords complex molecular architectures of myriad biological function. The most comprehensive recent review is by Bryant, Hunter, and Warren [58]. A sketch of the biosynthesis of tetrapyrrole macrocycles, suitable for this discussion, is provided in Figure 11. Two molecules of δ-aminolevulinic acid self-condense to form the pyrrole porphobilinogen [59], which upon tetramerization [60] affords uroporphyrinogen III [61], the juncture where tetrapyrrole biosynthesis diverges. Heme is perhaps the architecturally simplest product derived from uroporphyrinogen III; the derivation process entails conversion of two propionic acid groups to two vinyl groups, dehydrogenation (6e^–^, 6H^+^) of the macrocycle (to convert the non-aromatic porphyrinogen to the aromatic porphyrin), and insertion of iron. The somewhat late-stage derivatization ensuing from uroporphyrinogen III is referred to as the Ni branch (yielding F_430_) and the Co branch (yielding cobalamin, or vitamin B_12_), whereas ensuing from protoporphyrin IX are the Fe branch (yielding heme) and the Mg branch (yielding chlorophylls and bacteriochlorophylls) [62,63,64].

**Figure 12:**
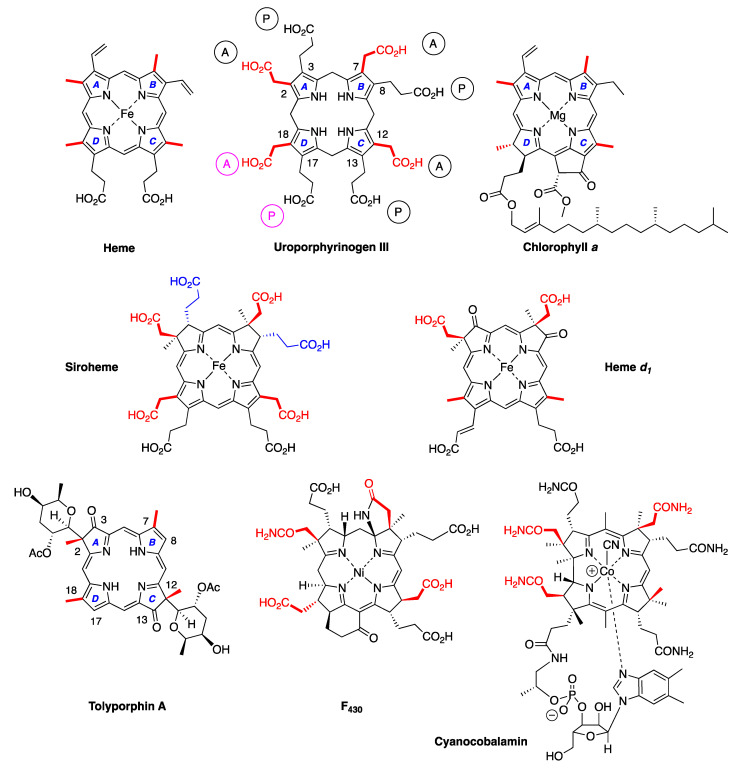
Common pattern of peripheral substituents in naturally occurring tetrapyrrole macrocycles. The numbering system of peripheral carbon atoms is displayed only for uroporphyrinogen III and tolyporphin A for clarity. The substituents displayed in red equate or correspond to Me in the pattern MeX-MeX-MeX-XMe (positions 2,3–7,8–12,13–17,18, respectively) upon circumnavigating the macrocycle. Adapted with permission from ref [66]; 2021, The Royal Society of Chemistry.

The structures of a collection of native tetrapyrrole macrocycles are shown in Figure 12. Uroporphyrinogen III, a biosynthetic intermediate, is the last universal common precursor to all naturally occurring tetrapyrroles. As an organizational feature, it is customary to label the pyrrolic rings *A–D* upon circumnavigating the macrocycle [65], beginning with the pyrrole in the upper left corner. The pyrrole bearing label A is also where the atom numbering begins, as shown for uroporphyrinogen III. The β-pyrrolic carbons are thus numbered 2, 3, 7, 8, 12, 13, 17, and 18 in circumnavigating the macrocycle.

The pattern of substituents about the perimeter of the uroporphyrinogen III macrocycle, beginning in ring A and proceeding to ring D, is AP-AP-AP-PA (tracking positions 2,3–7,8–12,13–17,18, respectively), where A is acetic acid and P is propionic acid [60,61]. The orientation of the “AP” groups is reversed (i.e., “PA”) in ring *D* relative to the AP groups in rings *A–C*. The acetic acid groups undergo decarboxylation to afford methyl (Me) groups in coproporphyrinogen III (not shown), giving MeP-MeP-MeP-PMe in proceeding from rings *A–D*. The same pattern of reversed substituents is manifested in ring *D* of heme and chlorophylls, regardless of other transformations to the peripheral groups; thus, the pattern is MeX-MeX-MeX-XMe, where X describes various substituents derived from the propionic acid units. The reversed pattern is emphasized for various pigments-of-life macrocycles, as shown in bold red in Figure 12. The reversed pattern prevails for F_430_ and cobalamin, notwithstanding the elaborate diversification that ensues between uroporphyrinogen III and these architecturally rich end products of biosynthesis [66].

Inspection of the structure of tolyporphin A shows the same pattern of substituents—MeX-MeX-MeX-XMe—in proceeding from ring A to D (tracking positions 2,3–7,8–12,13–17,18, respectively) as occurs as with other native tetrapyrroles (Figure 12). The structural paradigm also occurs for all known tolyporphins, including the dioxobacteriochlorin-type, oxochlorin-type, and porphyrin-type macrocycles (see Figure 6). The pattern is a telltale hallmark, a “smoking gun” as it were, for derivation of tolyporphins from uroporphyrinogen III [66]. Realization that tolyporphins A–R almost certainly derive from uroporphyrinogen III implies the existence of one or more enzymes that must remove propionic acid groups from uroporphyrinogen III or a downstream intermediate [66]. Such enzymes are extremely rare (see Section 11, Section 15 and Section 16) and potentially valuable for other applications. Considerations about the biosynthesis of tolyporphins [66] are further developed in Section 15 and Section 16, and are animated by the questions posed in the next section.

### 4.3. Questions

One important modus operandi of modern science is to study something specific in hopes of learning something general. Our interests in tolyporphins have centered on three large questions:

(1) All tolyporphins display at least two open β-pyrrole positions, and most contain at least one gem-dialkyl motif. Could enzymes from the tolyporphins biosynthetic pathway be exploited in chemoenzymatic syntheses of sparsely substituted, gem-dialkyl-equipped tetrapyrrole macrocycles to create tailored target compounds [66]?
(2) Tolyporphins collectively certainly might be regarded as *un mélange bizarre*—all like chlorophyll per provenance in a cyanobacterium, many part bacteriochlorophyll by virtue of the π-chromophore, many part chlorophyll on the basis of the absorption spectral properties (see Section 5), almost all part heme *d_1_* given the gem-dialkyl groups, and many like other natural products given the appended *C*-glycosides with structural diversity therein. Could knowledge of the biosynthesis of tolyporphins deepen understanding of tetrapyrrole biosynthesis broadly, as well as perhaps shed light on the (evolutionary) dichotomy between gem-dialkylated hydroporphyrins (cobalamin, F_430_, siroheme, and heme *d_1_*) and *trans*-dialkylated hydroporphyrins (chlorophylls and bacteriochlorophylls) [66]? What can be said or learned about the origin and distribution of putative enzymes for removing propionic acid groups from tetrapyrrole macrocycles? If such questions can be addressed, the richness of the repertoire of tolyporphins may prove to be an invaluable scientific portal.
(3) Cyanobacteria are known to produce abundant quantities of chlorophyll *a*, as required for photosynthesis, yet it is unprecedented for cyanobacteria to produce bacteriochlorin macrocycles of any type (14 of the 18 known tolyporphins are dioxobacteriochlorins). What are dioxobacteriochlorins doing, after all, in a photosynthetic bacterium [49]? An evolutionary model of the origin of natural molecular diversity [50,67,68] posits that [68] “most natural products will possess no biological activity of value to the producer and any biological activity found could well be fortuitous”. An ultimate finding that the existence of tolyporphins is nugatory with regards to physiological activity, for example, would not detract from the two aforementioned opportunities.

Attempts to answer the three large questions have animated many of the studies described in the following sections.

## 5. Photochemical Activity of Tolyporphin A

**Figure 13:**
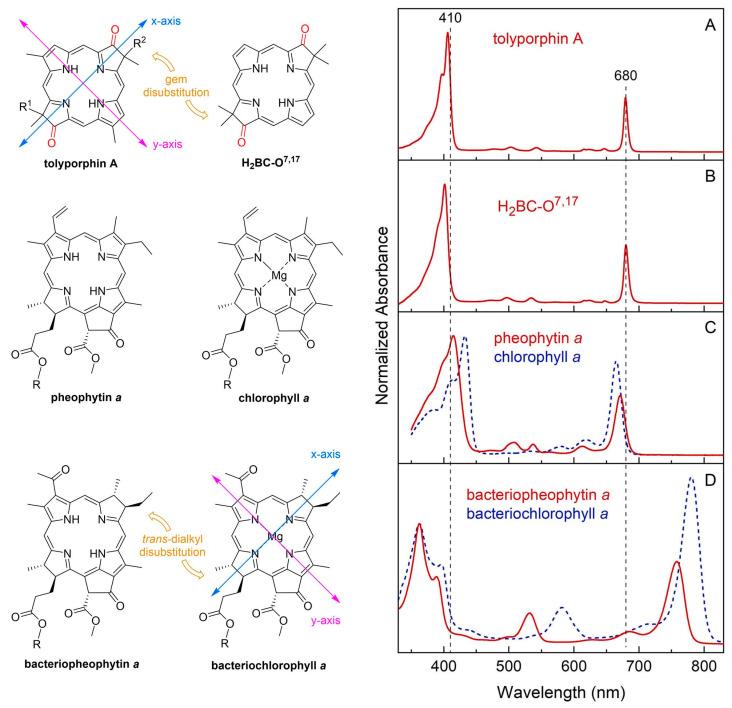
Right panel: Absorption spectra (normalized at the B band) in toluene at room temperature for tolyporphin A (**A**), synthetic bacteriochlorin **H_2_BC-O^7,17^** (**B**), native chlorins (**C**), and native bacteriochlorins (**D**). Left panel: Structures of compounds. The *x*-axis and *y*-axis are displayed for tolyporphin A and bacteriochlorophyll *a*; the same apply for the other tetrapyrroles, but are omitted for clarity. Adapted with permission from ref [49]; 2017, John Wiley and Sons, Inc.

The question “is tolyporphin A photochemically active?” stemmed from consideration of whether tolyporphins might serve a photosynthetic or photodynamic role in the cyanobacterium [49]. The absorption spectrum of tolyporphin A is shown in Figure 13 (right column, panel A). The spectrum is compared with that of a synthetic dioxobacteriochlorin (**H_2_BC-O^7,17^**, panel B) and four native photosynthetic tetrapyrroles (panels C and D). The synthetic dioxobacteriochlorin contains a gem-dimethyl group flanking the oxo moiety in each pyrroline ring, mirroring that of tolyporphin A. The photosynthetic tetrapyrroles include chlorophyll *a* and bacteriochlorophyll *a*, both of which are magnesium chelates, and pheophytin *a* and bacteriopheophytin *a*, the respective free base analogues. The structures are shown in Figure 13 (left panel). The gem-dialkyl group in each pyrroline ring of tolyporphin A (and the synthetic model **H_2_BC-O^7,17^**) is distinct from the *trans*-dialkyl motifs in the native photosynthetic tetrapyrrole macrocycles.

The spectroscopic axes are displayed for tolyporphin A and bacteriochlorophyll *a*. The *y*-axis bisects the nitrogen atoms of the two pyrrole units, whereas the *x*-axis bisects the nitrogen atoms of the two pyrroline units (Figure 13, left panel).

The absorption spectrum of tolyporphin A displays an intense band in the near-ultraviolet region known as the B band, which here is split into the B_y_(0,0) shoulder (397 nm) on the B_x_(0,0) peak maximum (406 nm). The spectrum also displays a band in the far-red region (678 nm) that reflects absorption causing transition from the ground state (S_0_) to the lowest singlet excited state (S_1_). The long-wavelength band is referred to as the Q_y_(0,0) band. The “y” label derives from spectroscopy, wherein the Q_y_(0,0) band stems from a transition that is largely polarized along the *y*-axis of the molecule. Weakly absorbing bands in the region between the B bands and the Q_y_(0,0) band stem from Q_x_ transitions or from the vibrational progression associated with the Q_y_ manifold. The reader is referred to ref [49] and references therein for further information in this regard. The topic of absorption spectroscopy of tolyporphins also appears in Section 9.2 and Section 10.

The spectral features of tolyporphin A agree very closely with those of the synthetic dioxobacteriochlorin **H_2_BC-O^7,17^**, and generally resemble those of chlorophyll *a* and pheophytin *a*. One key difference, however, is the very narrow Q_y_ band, as evidenced by the full-width-at-half-maximum (fwhm). The fwhm of the Q_y_ band of tolyporphin A is ~8 nm, to be compared with ~18 nm for that of chlorophyll *a* [69,70]. The molar absorption coefficient (ε) for the B_x_ band of tolyporphin A has been reported to be 148,000 M^−1^cm^−1^ (at 402 nm in ethanol) [52], which coheres with that of known dioxobacteriochlorins. The values of the molar absorption coefficient of tolyporphin A and of other tolyporphins are key to many detection schemes and have been revisited over time (see Section 9.2). All values of ε reported herein are in units of M^−1^cm^−1^, unless noted otherwise.

The spectral and photophysical properties of tolyporphin A, two native chlorins (chlorophyll *a* and pheophytin *a*), and two native bacteriochlorins (bacteriochlorophyll *a* and bacteriopheophytin *a*) are listed in Table 1. All spectra and results were obtained for the compounds in degassed toluene solution at room temperature. The purity of the tolyporphin A sample employed in these studies, provided by the Williams group at the University of Hawaiʻi, was an impressive 98.7% (Supplementary Materials, Section S2, Figure S4). The photophysical parameters listed in Table 1 are the singlet excited-state lifetime (τ_s_), the fluorescence quantum yield (Φ_f_), the intersystem crossing quantum yield (Φ_isc_), and the internal conversion quantum yield (Φ_ic_). Values of τ_s_, Φ_f_, and Φ_isc_ for bacteriochlorophyll *a* and bacteriopheophytin *a* in toluene have been reported [71,72]. The Φ_f_ values for pheophytin *a* and chlorophyll *a* in toluene also have been reported [73]. Tolyporphin A is fluorescent, with emission at 681 nm and a Φ_f_ value of 0.14. Fluorescence can legitimately be regarded as photochemically irrelevant—“the exhaust of the photochemical engine”—yet the assessment of fluorescence is important for two reasons: (1) the existence of fluorescence is often regarded as a proxy for photoactivity [74], and (2) the position of the fluorescence maximum sets an upper limit on the energy available in an excited-state process [57,75,76]. In this regard, tolyporphin A is typical of a broad sweep of tetrapyrrole macrocycles. A more explicit measure of potential photoactivity is given by the value of τ_s_, which indicates the timeframe available for reaction of the excited-state species; the value for tolyporphin A is 3.9 ns. The latter value is within a factor of 2 of those of the native photosynthetic tetrapyrroles in this comparison.

The spectral and photophysical features of tolyporphin A also were examined in solvents of diverse polarity (Table 2) [49]. The spectral features were largely constant across solvents. The yields of triplet states (given by Φ_isc_) are high, as expected, and reaction with ground-state oxygen (^3^O_2_) to generate singlet oxygen (^1^O_2_) is essentially diffusion-controlled.

In short, tolyporphin A is a typical tetrapyrrole macrocycle with rather normal excited-state behavior, both in the singlet state and in the triplet states. Accordingly, tolyporphin A is a photoactive compound similar to that of many other tetrapyrrole macrocycles. What is striking about the spectral properties of tolyporphin A, however, is that the position of the long-wavelength (Q_y_) band is shorter than that of the native bacteriochlorins bacteriochlorophyll *a* and bacteriopheophytin *a*. Tolyporphin A is a bacteriochlorin, more specifically, a dioxobacteriochlorin, with a chromophore identical to that of the synthetic dioxobacteriochlorin **H_2_BC-O^7,17^**. The two oxo groups are positioned along the *x*-axis of the molecule and cause the Q_y_ band to shift to a shorter wavelength than otherwise would be the case for a simple, unadorned bacteriochlorin. The role of the two oxo auxochromes has been exploited in the development of a fluorescent assay for the detection of tolyporphins (see Section 10.8).

## 6. Biology of the Tolyporphins-Producing Culture

### 6.1. The Culture

**Figure 14:**
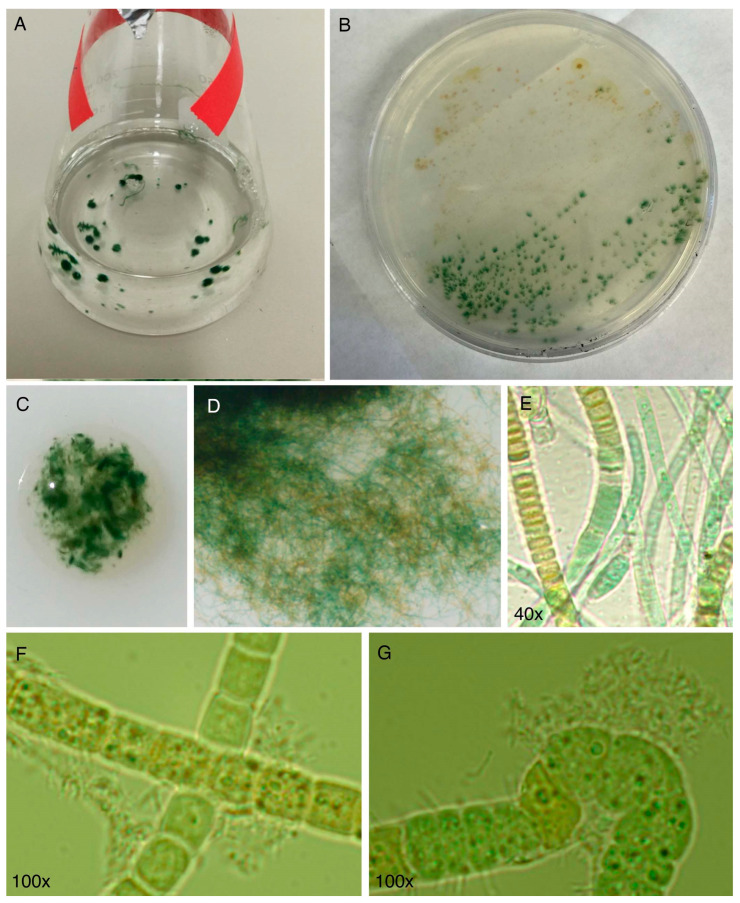
Photographs showing the non-axenic and filamentous nature of the HT-58-2 culture. Samples are as follows: In BG-11 media (**A**), on a BG-11 agar dish (**B**), suspended in water (**C**,**D**), and under an optical microscope (**E**–**G**).

**Figure 15:**
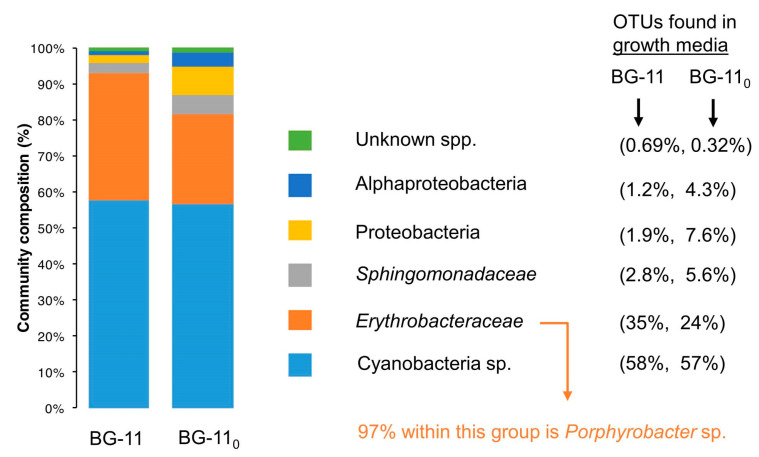
Operational taxonomic units (OTUs) were determined from HT-58-2 cultures grown in BG-11 or BG-11_0_ media. 16S rRNA V3-V4 regions from a culture grown in light for 20 days were amplified and sequenced using Illumina MiSeq. The absence of nitrate (BG-11_0_) slightly alters the distribution of HT-58-2 community bacteria. One *Porphyrobacter* sp. comprises 97% of the *Erythrobacteraceae* OTUs [79].

**Figure 16:**
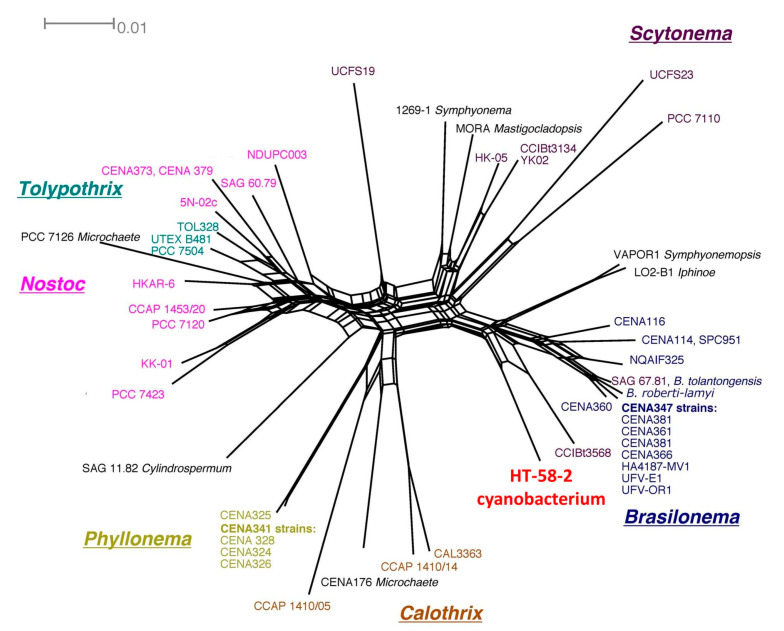
16S rRNA SplitsTree phylogenetic analysis of the HT-58-2 cyanobacterium. 16S rRNA sequences of filamentous cyanobacteria related to HT-58-2 were obtained from GenBank using BlastN; short clones and uncultured entries were omitted. All sequences were trimmed to the same 1140 bp from position 129 to 1269, covering six 16S rRNA variable regions (V2–V7). Sequences were aligned using ClustalW [85], followed by SplitsTree [86] neighbor-joining phylogenetic tree construction. Highly similar 16S rRNA sequences (identity greater than 99%, no gaps) are represented in the same terminal branch. Strains clustering together (clades) are color-coded. Other strains are coded in dark gray. The HT-58-2 16S rRNA sequence aligns closest to strain CCIBt3568, which is classified as a *Scytonema* [81], but both strains align with the larger collection of CENA *Brasilonema* strains. The scale bar represents the average number of nucleotide substitutions per site (here, 0.01 per 1 cm). Additional information is provided in ref [79].

The cyanobacterial culture HT-58-2 was originally described as a strain of *Tolypothrix nodosa* with the ability to produce tolyporphins, but no information was available about the culture. The culture was graciously provided to the authors at NC State University in late 2015 by Prof. Philip Williams, who as stated in Section 3, rather heroically rescued the strain from near oblivion. The culture arrived on a slant and was cultured successfully in our labs. Culturing was carried out with illumination in Erlenmeyer flasks containing either BG-11 or BG-11_0_ media. BG-11 media is widely used to culture cyanobacteria and contains a large quantity of nitrate as a soluble nitrogen source [77]. BG-11_0_ media is identical to BG-11, but lacks nitrate, and can be used for nitrogen-fixing cyanobacteria [77]. The compositions of BG-11 media reported may differ slightly in trace metals content [77,78]. There remains a minute amount of nitrogenous entities (ammonium or nitrate) in BG-11_0_ as counterions of trace species, but the total quantity is <0.5% of that in BG-11. The formulas used for BG-11 and BG-11_0_ media are listed in the Supplementary Materials (Section S3, Table S1). Herein, all examples that concern culturing of cyanobacteria refer to the use of continuous illumination with white light, unless noted otherwise. A typical culture condition was 28 °C with shaking at 190 rpm under continuous white light (62 μmol m^−2^ s^−1^) [79]. The following findings are significant:

(1) The culture is not axenic. Examination by light microscopy, fluorescence microscopy, and scanning electron microscopy revealed cyanobacterial filaments, not single cells, with filaments decorated with attached bacteria and the presence of free bacteria (Figure 14). In other words, HT-58-2 is not a pure culture; indeed, HT-58-2 could not be made axenic, regardless of treatment applied including organic solvents (dimethyl sulfoxide), biofilm inhibitors, antibiotics, cycloserine/cycloheximide, arsenite, and anaerobiosis [79]. The non-axenic nature of the culture has significant consequences for numerous studies. The filamentous nature of the cyanobacterium also presents challenges to investigation.

(2) Metagenomic surveys of the HT-58-2 culture in either BG-11 or BG-11_0_ media revealed a single cyanobacterium accompanied by diverse bacteria. Other operational taxonomic units (OTUs) in the cultures were dominated by the bacterial family *Erythrobacteraceae*, representing 35% and 24% of all OTUs in BG-11 and BG-11_0_, respectively. The dominant OTU within the *Erythrobacteraceae* family aligned with the 16S rRNA of Porphyrobacter sp [80], which accounted for 97% of the reads under both growth conditions. The remaining OTUs represented *Sphingomonadacea*, Proteobacteria, alphaproteobacteria, and unknown bacteria (Figure 15). Further studies of the effects of nutrients on the community bacterial population would be required if the production of tolyporphins requires participation by community bacteria.

(3) The 16S rRNA sequence of the HT-58-2 cyanobacterium showed close alignment with the proposed *Brasilonema* clade and not with the *Tolypothrix* clade. The 16S rRNA phylogenetic map is shown in Figure 16. The closest strain to the HT-58-2 cyanobacterium is assigned *Scytonema CCIBt3568* [81]. The genus *Brasilonema* was first described only 16 years ago by Fiore as a genus apart from that of *Scytonema* [82]. Since then, other *Brasilonema* species have been identified [83,84]; indeed, at the time of this writing, a Web of Science search (all databases) for “cyanobacteria and brasilonema” resulted in 62 hits.

### 6.2. The Cyanobacterium

**Figure 17:**
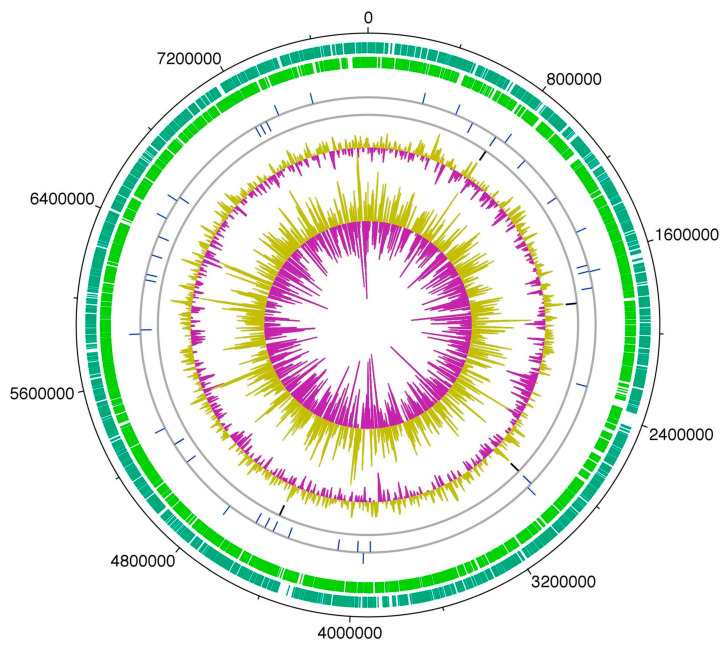
The genome of the HT-58-2 cyanobacterium is composed of dsDNA in a circular configuration (7.85 Mbp). Altogether, 6581 genes are distributed evenly throughout both strands of the genome (outer green rings). tRNAs (44) occur on both strands (first gray ring), whereas rRNA operons (4) are only on the reverse strand (second gray ring). The G+C content shows peaks at the rRNA operons and at other specific sites in the genome (first gold/purple plot). The GC skew (innermost gold/purple ring) does not show a strong strand bias. The genome is oriented with *dnaA* on the positive, clockwise strand starting 100 bp from position 1 [79].

Whole-genome sequencing of the HT-58-2 cyanobacterium proved surprisingly difficult, because the abundant, attached, community bacteria were readily lysed, whereas the cyanobacteria, equipped with a tough outer sheath, gave up their DNA less willingly. Eventually overcoming this impediment, and employing an array of PacBio, Illumina, and PCR sequencing methods, the cyanobacterial genome assembly was completed [79]. The assembly of genomes of filamentous cyanobacteria is known to be vexing [87]. The genome is circular, consists of 7.85 Mbp (42.6% G+C), and encodes 6581 genes (Figure 17). The genes for biosynthesis of tetrapyrroles (e.g., heme, chlorophyll *a*, and phycocyanobilin) were found to be dispersed throughout the chromosome, not localized in a cassette (see Section 11). Two plasmids also were found. The genome for the filamentous *Nostocales* cyanobacterium HT-58-2 is deposited in GenBank under accession number CP019636 [79,88].

The size of the genome of the HT-58-2 cyanobacterium (7.85 Mbp) is somewhat toward the larger end of known cyanobacterial genomes. As a general rule, filamentous cyanobacteria tend to have larger genomes than unicellular cyanobacteria. For example, the genome of *Anabaena* sp. 33047, a filamentous, nitrogen-fixing cyanobacterium, is 5.55 Mbp [89]. On the other hand, the genome of the unicellular freshwater cyanobacterium *Synechocystis* sp. PCC6803 is 3.57 Mbp [90]. Strains of the tiny unicellular cyanobacterium *Prochlorococcus*, which have undergone genome streamlining, are characterized by genomes of <2.7 Mbp [91]. All such genomes can be compared with that of the projected ancestral genome of cyanobacteria, which has 4.5 Mbp [87]. A recent review by the group of Fiore indicates that, to date, the largest cyanobacterial genome is 15.33 Mbp, whereas the smallest is 0.19 Mbp; the average size of some 516 known genomes is stated to be 4.8 Mbp [28].

### 6.3. Community Bacteria

**Figure 18:**
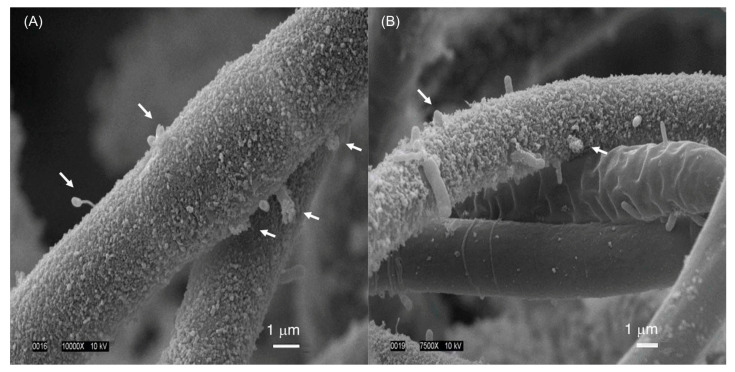
SEM images of HT-58-2 cyanobacterial filaments and attached community bacteria. The culture was grown in BG-11_0_ (devoid of nitrate) for 14 days following transfer from a 30-day culture in BG-11 (contains nitrate). Attached bacteria embedded in the cyanobacterial sheath are indicated with arrows (**A**), as are bacteria that appear more embedded in the cyanobacterial coating (**B**) [79].

The HT-58-2 isolate was cultured in BG-11 or BG-11_0_ media. The culture was examined by scanning electron microscopy (SEM). SEM images show cell morphologies attached and closely associated with the cyanobacterial filaments in both types of media (Figure 18). Several were rod-shaped, whereas others were prosthecate, stalked bacteria. Examination of samples grown in BG-11_0_ also showed the presence of an extracellular material coating the cyanobacterial filaments, along with some community bacteria apparently imbedded in the cyanobacterial sheath. The extensive washing of HT-58-2 that was carried out in preparation for SEM imaging appeared to have removed essentially all unattached bacteria, leaving only those bacteria that were physically attached to the filaments.

**Figure 19:**
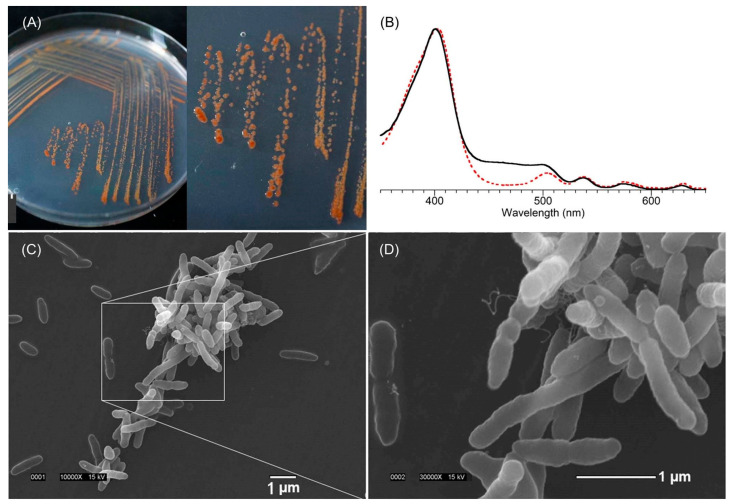
(**A**) During the initial isolation, strain BG-11.16 (hereafter termed *Porphyrobacter* sp. HT-58-2) was streaked onto PE agar [93] and incubated for six days in the light at 28 °C. The cells appeared as orange-pigmented colonies, along with slightly darker-pigmented colonies. (**B**) Overlay of the absorption spectra (in acetone/methanol, 7:2) of an organic extract of *Porphyrobacter* sp. HT-58-2 (black solid line) and protoporphyrin IX (red dashed line). (**C**,**D**) SEM images are shown of *Porphyrobacter* sp. HT-58-2 following growth for four days in PE medium [93] with continuous illumination [92]. Adapted with permission from ref [92]; 2018, Microbiology Society.

A key rationale for focusing on the attached bacteria was the concern that tolyporphins biosynthesis might be carried out by the attached community bacteria rather than the cyanobacterium, or that the biosynthesis might be carried out in synergistic fashion via the cyanobacterium–community bacteria holobiont [92]. Plating the HT-58-2 isolate on yeast–tryptone (YT) agar with growth under illumination led to isolation of 26 bacterial strains (all confirmed to be present in the HT-58-2 culture), of which 18 were identified as different Alphaproteobacteria and four to a single genus (*Variovorax*) of Betaproteobacteria. None was able to grow in BG-11 medium in the absence of the cyanobacterium. An orange-pigmented strain (Figure 19, panel A), designated BG-11.16, was found to have a 16S rRNA sequence matching that of the dominant *Porphyrobacter* sp. in the community analysis. Isolate BG-11.16, termed *Porphyrobacter* sp. HT-58-2, was sequenced; the genome is a single, circular dsDNA of 3,279,145 bp with genes distributed on both forward and reverse strands. The complete genome sequence of *Porphyrobacter* sp. HT-58-2 is deposited in GenBank with accession number CP022600 [92]. Annotation of the genome of *Porphyrobacter* sp. HT-58-2 showed the complete genes for the biosynthesis of heme and bacteriochlorophyll *a*, but no genes were identified resembling those in the putative biosynthetic gene cluster for tolyporphins (see Section 11) [92]. Growth of *Porphyrobacter* sp. HT-58-2 under diverse conditions, followed by extraction and scrutiny by absorption spectroscopy, did not reveal the characteristic signature bands of tolyporphins. Despite the presence of genes for the biosynthesis of bacteriochlorophyll *a*, extensive efforts at detection also did not identify the presence of bacteriochlorophyll *a* (or chlorophyll *a*). The absence of bacteriochlorophyll *a* in this case implies the absence of phototrophic growth under the conditions examined. Under some conditions, a spectrum resembling heme was observed, whereas in others, the immediate free base precursor to heme, protoporphyrin IX, was apparently formed (Figure 19, panel B). Scanning electron microscopy (SEM) images showed pleomorphic, albeit often rod-shaped, cells; some dividing cells appeared to be budding, while others were undergoing binary fission (Figure 19, panels C and D) [92].

**Figure 20:**
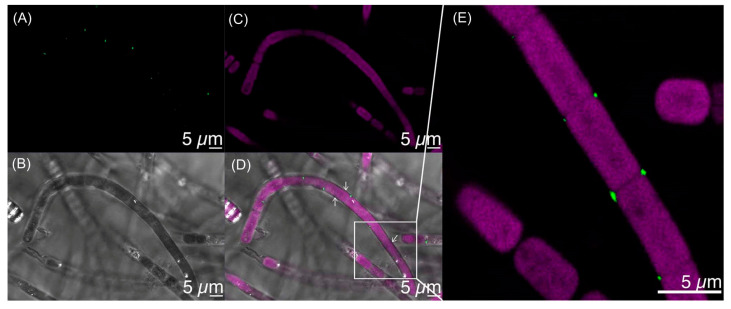
FISH of the HT-58-2 cyanobacterium–microbial culture using a fluorescein probe specific for *Porphyrobacter* sp. HT-58-2. Upon excitation at 488 nm, probe emission (499–535 nm) is displayed in green (**A**). The sample was observed without laser excitation under light microscopy (**B**). Upon excitation at 488 nm, filament autofluorescence (671–740 nm) is displayed in false-color magenta (**C**). A combination of panels A–C is shown in panel D. Partial magnification of one filament (**D**) is shown in panel (**E**) [92]. Reprinted with permission from ref. [92]; 2018, Microbiology Society.

To explore interaction of *Porphyrobacter* sp. HT-58-2 with the HT-58-2 cyanobacterium, a fluorescence in situ hybridization (FISH) experiment was performed. A fluorescein-labeled DNA probe targeted to the 16S rRNA of the *Porphyrobacter* sp. HT-58-2 was incubated with an HT-58-2 cyanobacterium–microbial sample (pre-washed with 10% DMSO to remove most other community bacteria). The fluorescence of fluorescein was evident at various locations on the filaments (Figure 20). Fluorescence also was detected at what appeared to be cell septa, at the apex of filaments, and likely under the sheath. The observations established the intimate association of *Porphyrobacter* sp. cells with the HT-58-2 cyanobacterial filaments [92].

The environment of the filamentous cyanobacterium viewed from a physical perspective (light-rich and aerobic) and nutrient perspective (exopolysaccharides, vitamins, and amino acids) reveals manifold growth opportunities for heterotrophs, such as *Porphyrobacter* spp. Further study is required to evaluate the cyanobacterial–microbial consortium particularly with regard to nutrient exchange and the role, if any, of tolyporphins [92]. (We note that a cyanobacterium is a microbe, hence the term “microbial community” or “microbial consortium” suffices to describe the entirety of the HT-58-2 culture, yet we retain the redundant term “cyanobacterium–microbial” for clarity and emphasis on the distinct organisms of interest.) Open questions are whether *Porphyrobacter* sp. HT-58-2 is a symbiont of the HT-58-2 cyanobacterium, and whether the two bacteria exchange nutrients in a process of metabolic syntrophy [94,95,96].

The comments of Allen [17] some 70 years ago remain relevant: “It might be expected that the biochemical and physiological problems posed by a group of organisms capable of such extraordinary versatility would attract many investigators to their study. The number of papers which have been published on such problems attests to the attraction, but the progress which has been made is relatively small. Much of this lack of progress must be traced to the difficulty which has been encountered in obtaining pure cultures of *Myxophyceae*, and the consequent inability to perform well-controlled experiments”. The two complicating biological features of the HT-58-2 culture, thus, are the presence of community bacteria attached to the cyanobacterium and the filamentous nature of the cyanobacterium.

### 6.4. Cyanobacterial Genomics Broadly

**Figure 21:**
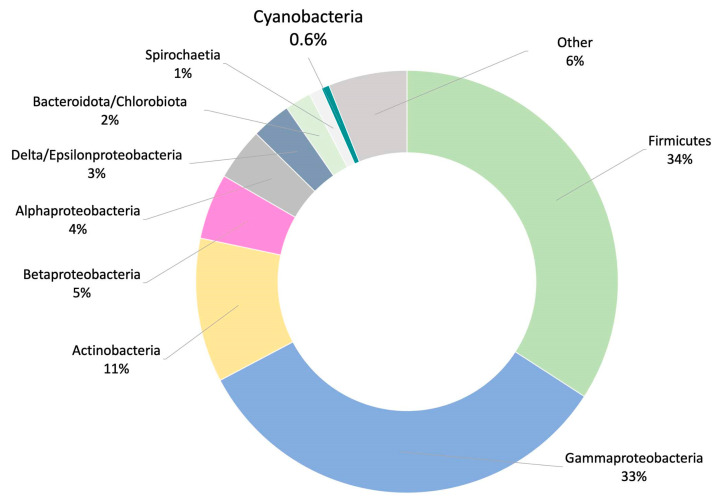
Known cyanobacterial genomes (as of 2017) account for ~0.6% of all prokaryotic genomes. Adapted from ref [87]; 2017, Frontiers Media S. A.

Cyanobacteria remain strikingly underrepresented in genomic databases versus that of other bacteria, as well as Archaea, as shown in Figure 21 [87]. As of this writing (June, 2023), GenBank entries at NCBI report 1,559,383 for Bacteria (41,328 complete genomes), of which 4257 are entries for cyanobacteria, with only 265 listed as complete (still ~0.6% versus Bacteria, as in 2017). Most others are scaffold or contig entries only. For further comparison, there were 14,698 genome entries for Archaea, of which 608 are listed as complete. Overall, given their importance to the Earth biosphere and their wide distribution in essentially every conceivable environmental niche, cyanobacterial genomes would seem to be vastly underrepresented in GenBank.

The known cyanobacterial genomes also are not representative of the geographic swath across the planet, a skew that likely has biased understanding of phylogenetic diversity [28]. As Leão and coworkers pointed out in 2021, “about 61% (1172 out of 1923) of the available National Center for Biotechnology Information (NCBI) strains (*sic.*) are from the unicellular genera *Prochlorococcus* and *Synechococcus* (subsection II), probably due to their ease of culturing, small genome sizes and their importance in oceanic nitrogen fixation and photosynthesis [97]. Moreover, organisms from these genera usually are less contaminated by associated heterotrophs in laboratory cultures. Finally, it has generally been found that members of these genera have a small number of biosynthetic gene clusters (BGCs, known for encoding the natural product biosynthetic machinery in microbes). Altogether, these features tend to facilitate genome sequencing and assembly [87,98]. However, these genera are less relevant for genome mining and drug discovery efforts due to the low quantity and diversity of their BGCs… Reasons for the relative scarceness of sequenced natural products rich cyanobacterial genomes may result from difficulty in culturing these types of filamentous marine cyanobacteria, the repetitive elements found in their genomes and their larger genome sizes”. Hence, the skew in understanding the ecological context of cyanobacteria is not only geographically biased, but also heavily biased toward biological simplicity.

The HT-58-2 cyanobacterium is tropical in origin. In general, tropical cyanobacteria have been comparatively little investigated [99]. Estimates suggest that <10% of tropical cyanobacterial diversity is known [100]. The Amazon Rainforest and River basin, for example, which accounts for some 20% of the world’s freshwater, is largely terra incognita with regards to knowledge of cyanobacteria [101,102]. New strategies also are needed to better understand the scope of natural products in this vast, relatively unexplored world of cyanobacteria [103]. In short, the overall dearth of knowledge is concerning, given the importance of cyanobacteria in fundamental ecological, evolutionary, and photosynthetic considerations, regardless of the possible practical, agricultural, and pharmaceutical benefits.

## 7. The Locale of Tolyporphins

**Figure 22:**
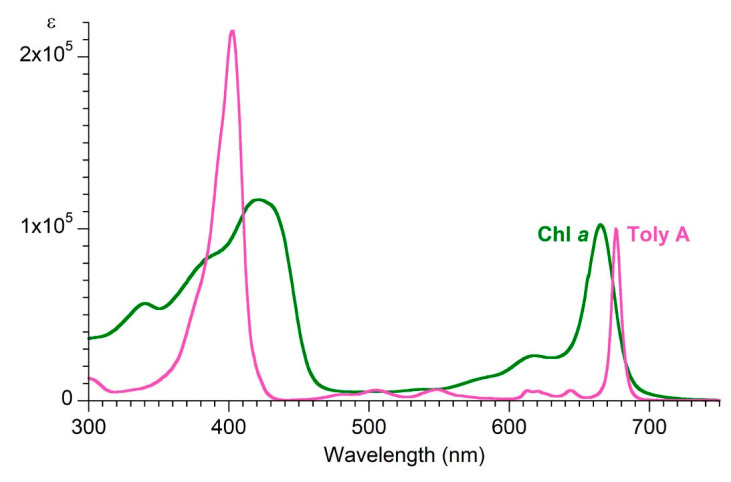
Absorption spectra (in methanol at room temperature) of chlorophyll *a* and tolyporphin A.

The existence of community bacteria embedded in the sheath of the filamentous cyanobacterium raised the question of where tolyporphins are located. The specific question arose as to whether the tolyporphins were located in the cyanobacterium or in the community bacteria. If this were a heterogeneous collection of unicellular organisms with tolyporphins as the only significant visible absorber, the question likely could be addressed in a straightforward manner. Tools of microscopy and/or flow cytometry could be brought to bear. But, the HT-58-2 culture contains photosynthetically active cyanobacteria, which are rich in chlorophyll and other pigments, such as phycobilisomes (combined phycocyanin and phycobilin species), in addition to tolyporphins. The absorption spectrum of chlorophyll *a* overlaps the spectrum of each individual type of tolyporphin, as illustrated by the spectra of tolyporphin A (Q_y_ maximum at 676 nm) [104] and chlorophyll *a* (Q_y_ maximum at 666 nm) [70] in methanol, as shown in Figure 22. Both chlorophyll *a* and tolyporphin A exhibit a Q_y_ band with ε ~100,000 M^−1^cm^−1^, as shown in Figure 22 (see Section 9.2 and Section 10.4 for more expansive description of this topic). Moreover, the cyanobacterial cells are organized in filaments, rather than as single cells. Hence, identification of the locale of tolyporphins was expected to be challenging.

### 7.1. Imaging Studies

Hyperspectral confocal fluorescence microscopy (HCFM) has been used previously to image the location of pigments in cyanobacteria [105]. The same technique was employed to probe the locale of tolyporphins within live cells under growth conditions that varied in the composition of the media, extent of illumination, and age of the culture [106]. A representative image set was subjected to multivariate curve resolution (MCR) to obtain unbiased spectra of pure components. For individual pixels in each image, the relative abundancies of the respective components were calculated, which enabled renderings in three dimensions in the organisms.

**Figure 23:**
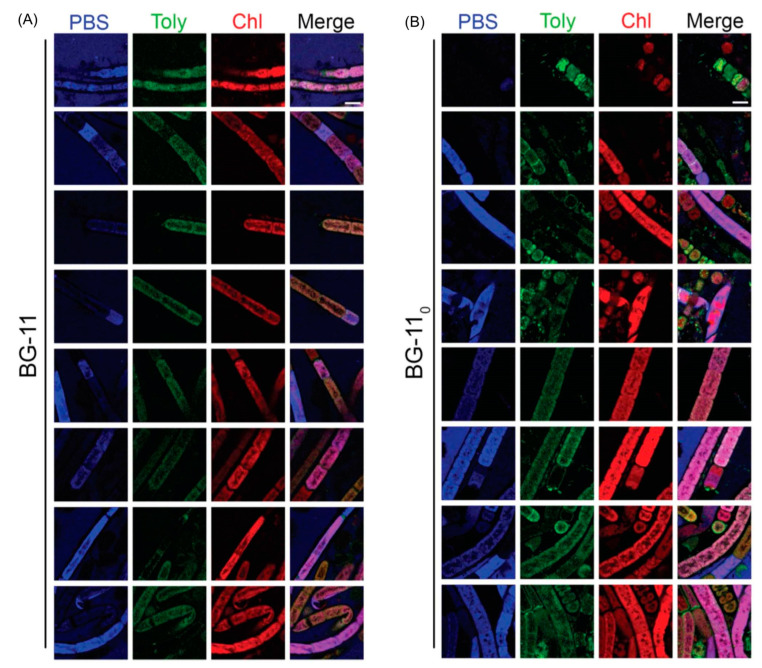
Representative MCR concentration maps of HT-58-2 cultures grown for 30 days in BG-11 media (**A**) or BG-11_0_ media (**B**). The three main components include phycobilisomes (PBSs), tolyporphins (Toly), and chlorophyll *a* (Chl). Image intensities in panels A and B are scaled equally. The scale bar represents 5 μm. The colors of the constituent labels at the top of both panels constitute the false-colors used for the corresponding signals of those constituents in the images [106]. Reprinted with permission from ref [106]; 2019, Springer Nature.

Images obtained upon growth for 30 days (under constant white illumination, 28 °C) are shown in Figure 23. Cultures grown in nitrate-depleted media (BG-11_0_ versus nitrate-rich, BG-11) over this time period profoundly increase the production of tolyporphins to levels rivaling that of chlorophyll *a*. Regardless of the presence or absence of nitrate, the chlorophyll *a* and phycobilisomes (combined phycocyanin and phycobilin components) co-localize in the filament outer-cytoplasmic region. A phycobilin-rich biofilm appears to wrap the HT-58-2 filaments and is present in the extracellular matrix, regardless of culture conditions. Tolyporphins are observed in a diffuse pattern, mimicking the chlorophyll *a* localization, upon growth in BG-11. On the other hand, nitrate deficiency (BG-11_0_) results in localization of tolyporphins in a distinct peripheral pattern in cells, including distinct puncta of tolyporphins at the septa between cells and at the end of filaments. Upon continued growth in BG-11_0_ media for 60 days, the peripheral localization of tolyporphins was lost [106].

**Figure 24:**
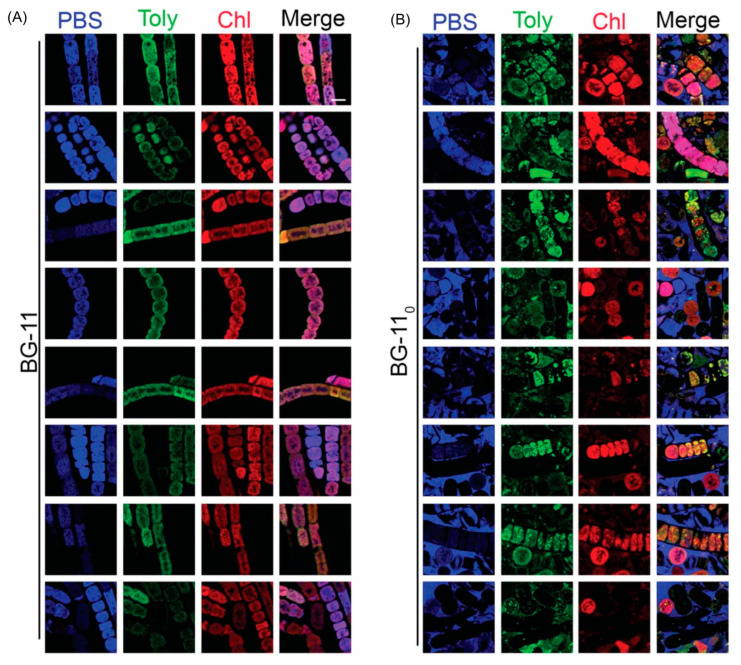
Representative MCR concentration maps of HT-58-2 cultures grown for 33 days and then dark-adapted for 24 hours. (**A**) BG-11 media. (**B**) BG-11_0_ media. The three main components include phycobilisomes (PBSs), tolyporphins (Toly), and chlorophyll *a* (Chl). Image intensities in panels A and B are scaled equally. The scale bar represents 5 μm. The colors of the constituent labels at the top of both panels constitute the false colors used for the corresponding signals of those constituents in the images [106]. Reprinted with permission from ref [106]; 2019, Springer Nature.

Additional images were collected for cells grown under constant white-light illumination (28 °C) for 33 days and then subjected to ~16–24 h dark adaptation (Figure 24). Dark adaptation did not influence the localization and abundance of tolyporphins; however, the phycobilin-rich biofilm that wraps the HT-58-2 cyanobacterium was greatly modulated upon growth under BG-11_0_ conditions.

**Figure 25:**
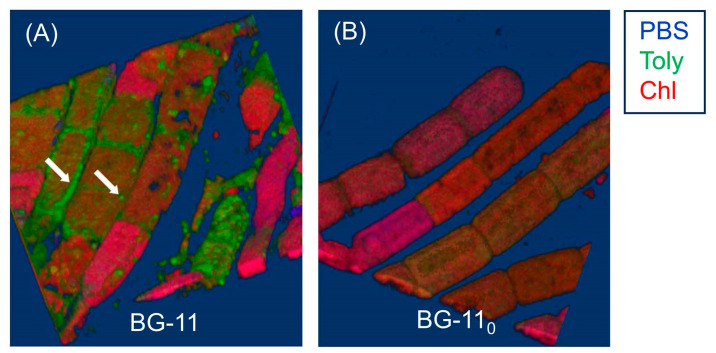
Three-dimensional volume renderings of HCFM images obtained from 30-day HT-58-2 filamentous cells grown in BG-11 media (**A**) or BG-11_0_ media (**B**). The signal of tolyporphins (false-colored green) in BG-11_0_ media is enriched at the septa between cells, as well as in puncta within individual cells (highlighted with white arrows). The other components are false-colored, as shown in the legend and in Figure 23 and Figure 24 [106]. Reprinted with permission from ref [106]; 2019, Springer Nature.

The images in Figure 23 and Figure 24 are displayed in two dimensions. The images were single optical slices through the near-center of the filaments. HCFM image stacks also were collected. The MCR concentration maps from these stacks were used to generate 3-dimensional volume renderings (Figure 25). The tolyporphins in cells grown in BG-11_0_ are revealed in punctate, intracellular patterns. Tolyporphins are enriched at the septa between the cells in each filament. The distinct localization pattern of tolyporphins under nitrogen stress (BG-11_0_) suggests purposeful function, delineation of which will require additional studies.

### 7.2. Estimates of Polarity

The apparent preferential localization of tolyporphins in the outer sheath, presumably membrane-like, environment is not surprising. Equilibrium partition coefficients between octan-1-ol (more generally referred to as *n*-octanol) and water, where octan-1-ol comprises a surrogate for the bilayer lipid membrane, were calculated [106]. The values are regarded as a measure of membrane permeability and localization. The calculations were carried out using the program Chemdraw 16.0 (Perkin-Elmer Informatics, Inc., Waltham, MA, USA). The results are termed cLog*P* values, where *P* = ([tolyporphin] in octan-1-ol)/([tolyporphin] in water) and “c” refers to calculated as opposed to experimentally determined. The cLog*P* values for tolyporphins generally fall in the range of 1.7–3.4, implying high preference for membrane versus aqueous localization (Table 3). The values in entries 1–13, 19, and 20 were reported previously [106], whereas the values of entries 14–18 concern recently discovered tolyporphins and are reported here. The cLog*P* values were calculated for all of the compounds using Chemdraw 22.2.0, which yielded identical results for those assessed previously using Chemdraw 16.0.

The cLog*P* values of the tolyporphins, all of which contain oxo groups (and typically two hydroxy and two ester groups) are much lower than that of the very hydrophobic chlorophyll *a* (14.8) and bacteriochlorophyll *a* (13.3). The cLog*P* values have at least two limitations with regards to meaningful interpretation. First, the values calculated depend significantly on the method of calculation employed, although a comparative analysis showed consistent trends across multiple software programs [107]. The comparative analysis concerned diverse tetrapyrrole macrocycles and polycyclic aromatic hydrocarbons [107]. A previous report stated the cLog*P* value of tolyporphin A to be 0.11, which corresponds to a ratio of 1.3:1 in partitioning between octan-1-ol versus water [21]; the reported value implies a surprising hydrophilicity. Recently, the cLog*P* value of tolyporphin K was stated to be 4.0 [108]. Second, the calculations do not take into account passive binding interactions with biomolecules or possible active localization processes, only equilibrium partition between two distinct solvent phases. On the basis of the cLog*P* values, as well as chemical intuition, it is expected that tolyporphins would preferentially localize in membranous structures, consistent with the HCFM results. The suggestion of purposeful function stated above thus bears on the rationale for enhanced production under soluble nitrogen deprivation, given that membranous localization is expected to occur spontaneously on the basis of the lipophilicity of all members of the tolyporphins family.

The questions about tolyporphins can be colloquially expressed as what (molecular composition), how (biosynthesis), why (function), how much (quantity), and where (locale). The use of HCFM has enabled the last issue to be addressed, by establishing the relative abundance and envelope localization of tolyporphins in filamentous cells under various growth conditions. The ability to identify novel tetrapyrroles in the presence of chlorophyll *a* within a very complex and heterogeneous filamentous cyanobacterium–microbial consortium suggests broad and as-yet unrealized applications in the photosynthetic sciences. The recent advent of spectral databases of endogenous pigments (chlorophylls [70], phyllobilins [109], flavonoids [110], and bilins and phycobilins [111]) may be facilitative in this regard. Questions concerning the isolation of tolyporphins are described in the next section.

## 8. Isolation of Tolyporphins

The isolation of tolyporphins has been reported on a number of occasions over a 25-year period [20,45,46,47,69,112]. The first isolation yielded solely tolyporphin A [20]. To our knowledge, the next three reports, by Prinsep and coworkers in 1995 [45], 1998 [46], and 2011 [47], appear to have been fractionations from a single large-scale culture carried out no later than 1995; this large-scale culture is referred to here as the “1995 culture”, completely cognizant that the culture could have been grown in the period since discovery in 1989 through 1995. As Prinsep recounted about her postdoctoral work at the University of Hawaiʻi (September 1990–September 1991) and thereafter upon return to New Zealand (NZ) [113] “I worked on the original culture grown in Hawaiʻi and cultured some myself there for labelling experiments (which didn’t work). It was cultured several times again after I left Hawaiʻi and sent to me in NZ. Looking at my old lab notebook, I received 4 batches of material in total, one in March 1992, one in March 1993 and two in November 1993, with the latter two representing low and high passage cultures”. Hence, the size of the cultures and the exact number of cell passages that gave rise to such materials (dried cell mass) are not known. The presumed, single large-scale culture was accompanied by fractionation and identification of tolyporphins B–M. The yields from the large-scale culture are provided in Table 4. From 93 g of dried cell mass was obtained 123 mg of tolyporphin A. The total percentage of tolyporphins B–I (0.12%) is comparable to that for tolyporphin A alone (0.13%). Thus, production of tolyporphin A dwarfs that of any other individual tolyporphin member, which likely explains the original isolation of tolyporphin A [20]. The first isolation of tolyporphin A from the HT-58-2 culture reported a yield of 0.03% (not shown in Table 4), but further information was not presented, as the focus at the time was on the proof of structure of tolyporphin A [20]. For perspective, the average yield of dried cell mass in the next reports was 0.2 g/L [45] or 0.17–0.33 g/L [46].

**Figure 26:**
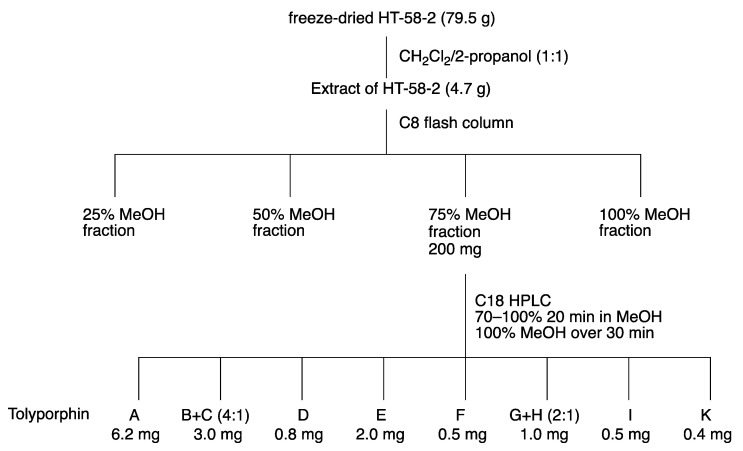
Flowchart for isolation of tolyporphins from the HT-58-2 culture [69]. Reprinted with permission from ref [69]; 2018, John Wiley and Sons, Inc.

The emphasis on the research timeline has a rationale. The culture was effectively “lost” for nearly 20 years, but was resurrected by the Williams group at the University of Hawaiʻi, as described in Section 3. The Williams group at the University of Hawaiʻi carried out a large-scale preparation (“2017 culture”), leading to the 2017 report [69]. The fractionation procedure is outlined in the flowchart of Figure 26. The cell mass is freeze-dried and then extracted with dichloromethane/2-propanol (1:1), followed by chromatography. The results are shown in Table 4 for comparison with those obtained from the 1995 culture. The yields of the various tolyporphins showed similar trends as reported from the 1995 culture [45], particularly, that tolyporphin A was the dominant species among the tolyporphins, but the quantities were diminished. Tolyporphin J was not isolated upon fractionation of the extract from the 2017 culture. The factor of decline is provided in the rightmost column of Table 4, which shows declines ranging from 2–21-fold. This decline may have a host of origins, including a weakened strain, as well as non-optimal culture conditions.

The report by Prinsep and coworkers [45] states that “uniaxial, nonaxenic cultures of HT-58-2 were grown on BG-11 medium in 20-litre glass bottles as described elsewhere [19]”, yet the report referred to describes a variety of culture conditions. Patterson et al., referring to the overall mission concerning ~1000 cyanophyte strains, state that [19] “Each isolate was tested in a variety of growth media to determine which of the various standard formulations yielded acceptable growth. The most commonly used medium was A3M7, a modification of Allen’s medium #3 [17]. Other freshwater media included BG-11 [114] and Allen’s [115]. Marine media were prepared by supplementing BG-11 or Allen’s medium with 1 or 2% (*w*/*v*) NaCl”. Thus, there remains some uncertainty as to the composition of the medium employed in the original culture conditions that gave rise to the disparate production in 2017 versus 1995. Original notebook pages offer no clarity, but the mention of A3M7 may be a salient point (Supplementary Materials, Section S1, Figures S2 and S3).

The results from 2017 shown in Table 4 were obtained in BG-11 medium [69]. The 2017 culture was carried out in BG-11m medium, which is identical to BG-11, but replaces citric acid and 6.0 mg/L of ferric ammonium citrate in BG-11 with 3-(*N*-morpholino)propanesulfonic acid (MOPS) buffer and ferric chloride hexahydrate. The large-scale culture was carried out twice in 12 × 20 L glass carboys (480 L total; e.g., Figure 9). Other interpretations concerning such diminution in production versus the original culture include a weakened cyanobacterial strain or some systematic error [69]. The ingredients in the A3M7 and BG-11 media are listed in Supplementary Materials, Section S3, Table S1.

Studies of culture conditions have continued for HT-58-2 [48]. HT-58-2 was cultured for 30 days in A3M7 and BG-11. Both A3M7 and BG-11 contain a soluble nitrogen source; the former contains sodium nitrate and ammonium chloride (and ammonium vanadate), whereas the latter contains a large quantity of sodium nitrate and trace amounts of other nitrogenous salts (ferric ammonium citrate, cobalt nitrate) [19,114]. In general, cyanobacteria can assimilate atmospheric nitrogen, as well as soluble nitrogenous compounds (nitrate; nitrite; ammonium; cyanate; and some amino acids, such as arginine, glutamine, and glutamate) [116]. The freeze-dried cell mass was extracted in the standard way with dichloromethane/2-propanol (1:1). Instead of chromatography to fractionate the tolyporphins into isolated components, the mixture of tolyporphins was analyzed by liquid chromatography coupled to mass spectrometry (LC-MS); the analytical technique is incisive (see Section 10.5). The results showing the relative quantities of the various tolyporphins (on the basis of internal standards) are shown in Table 5.

Several results are salient. First, tolyporphin A is the dominant member in the tolyporphins family in the two media. Second, the relative amount of tolyporphin A is twice as much in A3M7 compared to BG-11. These results and the 1989 notebook scrawl (Supplementary Materials, Section S1, Figures S2 and S3) strongly suggest the original culture medium may have been A3M7. Nonetheless, studies in the past few years of the resuscitated HT-58-2 culture in A3M7 still do not result in production levels comparable to those reported for the 1995 culture [104]. The use of LC-MS has enabled monitoring of tolyporphins *vis à vis* growth over time and, in so doing, ferreting out in-depth information concerning the evolution of individual tolyporphin species. Analysis in this manner has provided insights into the timecourse of production of various hydroxyl versus *O*-acetyl species [104]. As the genes for tolyporphins production are confirmed (see Section 16), it will be interesting to study the interplay of environmental conditions (light, nutrients) with gene expression and biosynthetic production of individual tolyporphins, and perhaps learn how to elevate production of tolyporphins individually and collectively.

## 9. Analytical Features of Tolyporphins

### 9.1. Mass Spectrometry 

Mass spectrometry is an essential tool for chemical characterization. In this regard, for the tolyporphins family, consideration is warranted of the presence of isomers, the molecular weight, the number of appended sugars (which can affect fragmentation patterns), and the nature of the chromophore. All of these features are summarized in Table 6. The table is organized first on the basis of chromophore type, including 14 dioxobacteriochlorins, 3 oxochlorins, and 1 porphyrin. The table is organized second on the basis of molecular weight, in descending order. There are three sets of isomers: tolyporphins L–O; B and C; and G and H. The presence of isobaric species causes the 18 distinct tolyporphins to appear as 13 distinct masses in the absence of any fractionation method. The use of mass spectrometry for detection and identification of tolyporphins is described in Section 10.

### 9.2. Absorption Spectroscopy

Absorption spectroscopy is not applied very frequently in organic chemistry on the whole given the greater incisive power of NMR spectroscopy for structure elucidation, but remains a central tool in the tetrapyrrole field owing to the strong and distinctive spectral bands exhibited by the tetrapyrrole chromophore. Absorption spectroscopy is often the simplest method of detection and analysis for tetrapyrroles given the strong absorption bands in regions of the spectrum, namely, 350–700 nm, where few other natural products absorb. The absorption spectrum of tolyporphin A is displayed in Figure 13. The spectrum exhibits a strong near-ultraviolet (NUV) band and a comparatively weaker, but still strong, long-wavelength band in the red spectral region. The NUV band bears the spectroscopic label B; the B band is also known as the Soret band in honor of Jacques-Louis Soret [117], who noticed the extinction of light in this region caused by blood (i.e., by the porphyrin heme). The long-wavelength band bears the spectroscopic label Q_y_.

The B band is the manifestation of two transitions, the B_x_ and B_y_ bands. In bacteriochlorins, the two transitions are typically observed as distinct peaks. In dioxobacteriochlorin-type tolyporphins, the two transitions are largely coalesced. Subtle changes in molecular structure and environment can cause slight shifts in the positions of the B_x_ and B_y_ bands, in which case the resulting observed B band may be broader and less intense, or sharper and more intense. A brief overview of tetrapyrrole spectroscopy is provided in a recent review [118], whereas a more expansive description is provided in the classic treatise of Gouterman [119]. Regardless of band shape, the molar absorption coefficient (ε, in M^−1^cm^−1^) is typically given at the peak of the band. A band may be broadened and decreased in intensity, or sharpened and increased in intensity, with relatively constant integrated absorption due to such spectral changes. Still, the ε value measures peak intensity, not integrated intensity. Hence, slight changes in structure or environment can cause a change in the ε value. On the other hand, the Q_y_ band is more of a “pure” transition and is less prone to such changes.

**Figure 27:**
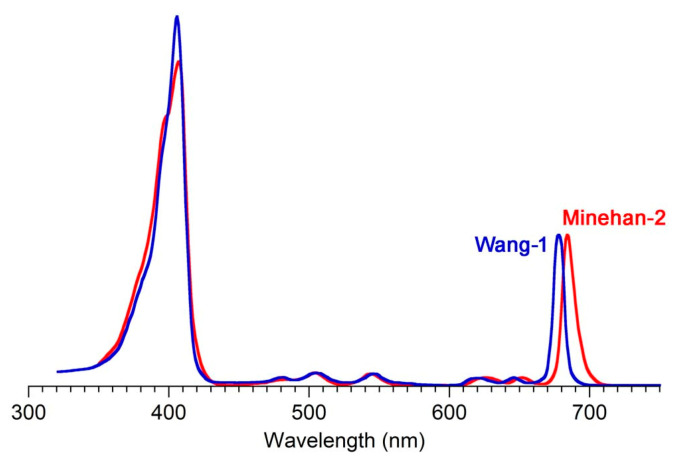
Absorption spectra (normalized at the Q_y_ band) in CH_2_Cl_2_ of a diastereomer of tolyporphin A diacetate (**Minehan-2**, red trace) [120] and tolyporphin A diacetate (**Wang-1**, blue trace) [69]. Adapted with permission from ref [69]; 2018, John Wiley and Sons, Inc.

The absorption spectra of two synthetic tolyporphins, the *O*,*O*-diacetyl derivative of tolyporphin A (**Wang-1**) and the *O,O*-diacetyl derivative of the diastereomer of tolyporphin A (**Minehan-2**), are shown in Figure 27. The origin of the two synthetic tolyporphins is described in Section 2. The structures are displayed in Figure 5. The spectra—provided in print form from a notebook [69] and a PhD thesis [120]—were digitized in a partially automated process [121]. The B band of **Minehan-2** shows a hint of the individual B_x_ and B_y_ transitions given the apparent shoulder on the short-wavelength side, whereas the B band of **Wang-1** is smoother and sharper. The B band of **Wang-1** is slightly more intense versus that of **Minehan-2** upon normalization of the spectra at the Q_y_ band.

**Figure 28:**
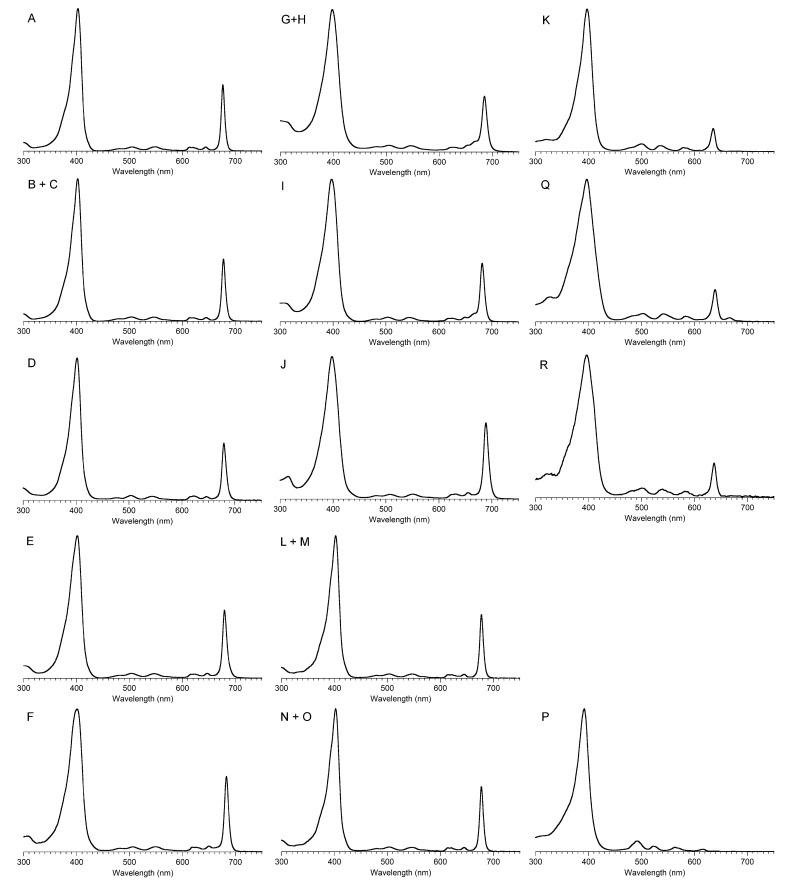
Absorption spectra (normalized at the Soret band) of tolyporphins in methanol at room temperature [104].

The possible coalescence or distension of the B_x_ and B_y_ bands and consequent effects on the measured ε value notwithstanding, the reported values for ε have varied substantially across the family of tolyporphins. The reported values are listed in Table 7, column 4. The value of ε for the Q_y_ band of dioxobacteriochlorin-type tolyporphins ranges from 1500 to 68,500 M^−1^cm^−1^. Comparative analysis with six synthetic dioxobacteriochlorins indicates a more reasonable (and, hence, proposed) value of ε for the Q_y_ band of all dioxobacteriochlorin-type tolyporphins is 100,000 M^−1^cm^−1^, as shown in the rightmost column of Table 7. Similar analysis for the oxochlorin-type tolyporphins (K, Q, and R) indicates a consensus (and, hence, proposed) value of ε for the Q_y_ band is 35,000 M^−1^cm^−1^. The absorption spectra of all native tolyporphins are shown in Figure 28.

For oxochlorins and dioxobacteriochlorins, a progression of bands is observed across the visible region (at longer wavelengths than the B band and edging near, if not into, the near-infrared region). Such bands collectively are referred to as Q bands. The longest wavelength band invariably stems from an “origin” transition between the lowest vibrational level of the ground state and the lowest vibrational level of the excited state, which would be indicated by the label Q_y_(0,0). The “(0,0)” label indicating an origin transition is generally omitted to describe the long-wavelength transition of chlorins and bacteriochlorins, unless distinction is called for.

With free base porphyrins, the longest wavelength band, labeled Q_x_(0,0), is very weak, with an ε value often of only 1000–5000 M^−1^cm^−1^. In free base porphyrins, the *x*-axis and *y*-axis labels are such that the second band in a typical five-band progression proceeding from short to long wavelength is labeled Q_y_(1,0) [122]. The Q_y_(1,0) band is often the most intense absorption band in the visible region (excluding the Soret band). Hence, for tolyporphin P, a porphyrin, the proposed value of ε for the Q_y_(1,0) band is 14,500 M^−1^cm^−1^. The refinement of the molar absorption coefficients as outlined was reported only recently, in 2021 [104].

The absorption spectra of all native tolyporphins shown in Figure 28 are organized in accord with compound class. Tolyporphins A–J and L–O are dioxobacteriochlorins and exhibit spectral properties that are similar to one another, but still not quite identical to each other, as indicated by the wavelength for the peak maximum in the red region. Tolyporphins K, Q, and R are oxochlorins and exhibit spectral properties that are similar to one another, but still not quite identical to each other. Tolyporphin P is a porphyrin and, as expected, lacks the intense long-wavelength band characteristic of the dioxobacteriochlorins and oxochlorins [104]. The spectra of tolyporphins [104] are available at the website www.photochemcad.com (accessed on 12 July 2023), along with spectra for β-substituted porphyrins [107], chlorophylls [76], phyllobilins [109], bilins and phycobilins [111], flavonoids [110], and >300 common organic compounds [123].

**Figure 29:**
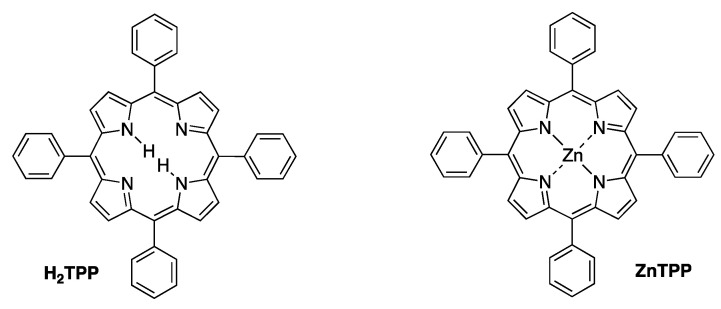
Benchmark porphyrins.

The question frequently arises as to why the reported molar absorption coefficient (ε) values for tetrapyrrole macrocycles are often low or vary across a wide range. This issue has been studied in detail, and a litany of possible explanations has been proffered [118]. One common occurrence with tetrapyrrole compounds is the use of very tiny quantities following chromatography, where weighing of samples is fraught, and non-light absorbing contaminants (solvent, chromatographic media, etc.) often are present and not detected; such conditions are prone to elicit error. Many of the tolyporphins listed in Table 7 were obtained in small quantities. The benchmark synthetic macrocycles in the tetrapyrrole field are free base *meso*-tetraphenylporphyrin (**H_2_TPP**) and the zinc chelate thereof, *meso*-tetraphenylporphinatozinc(II) (**ZnTPP**). The structures are shown in Figure 29. The molar absorption coefficient values for these two benchmark compounds have been determined hundreds of times over a period of nearly three-quarters of a century. Still, the reported ε values for the Soret band of **H_2_TPP** and of **ZnTPP** encompass an astonishing range of <100,000 M^−1^cm^−1^ to >1,000,000 M^−1^cm^−1^ [118]. Regardless of such inconsistencies, the spectra in Figure 28 illustrate the strong similarities and small differences among members of the dioxobacteriochlorin-type and oxochlorin-type tolyporphins, as well as the significant differences in the long-wavelength region of the tolyporphins with a dioxobacteriochlorin, oxochlorin, or porphyrin chromophore. The proposed values for ε listed in Table 7 should be valuable for future quantitative purposes. In short, absorption spectroscopy provides a convenient first approach for identifying and quantifying tolyporphins produced by HT-58-2 and by other potential strains. The next section considers a host of analytical methods for qualitative and quantitative assessment of tolyporphins.

## 10. Quantitative Analysis of Tolyporphins

- What quantities of tolyporphins are biosynthesized?
- How does the production change with growth conditions?
- How can tolyporphins be detected easily in the search for new producers?

Each question above is ostensibly simple and would be expected to elicit a straightforward answer, but in actuality, there are significant challenges to be confronted. The challenges stem from (1) the presence of 18 distinct tolyporphins among three classes of macrocycles: 14 dioxobacteriochlorins, 3 oxochlorins, and 1 porphyrin. Within a given class, most have an absorption spectrum that is similar, but not identical, to other members. And, among members in the family, there are isomers. (2) The origin of tolyporphins in a filamentous cyanobacterium–microbial consortium. (3) The presence of a large quantity of chlorophyll *a*, the absorption of which typically dwarfs that of the tolyporphins.

The presence of an abundance of chlorophyll *a* is a serious obstacle to many schemes for detection of tolyporphins in cyanobacteria. The quantity is substantial, as shown in the examples of Table 8. The quantities are expressed as the amount of chlorophyll *a* per gram of dry weight of the organism. The range of 2.6 μg–16 mg of chlorophyll *a* per g of dry cyanobacterial mass presents challenges, at least at the upper end of chlorophyll *a* content, to detection therein of smaller quantities of tolyporphins. The HT-58-2 cyanobacterium is quite similar to other cyanobacteria, at least on the basis of these limited comparisons. A rule of thumb drawn from the 2017 culture of HT-58-2 grown in BG-11 is that the content of chlorophyll *a* (~1%; Table 8) is ~100 times greater than that of tolyporphin A (~0.01%; Table 4).

A range of methods to identify tolyporphins in cyanobacterial cultures has been explored. The methods encompass sample extraction protocols and analytical characterization. The analytical characterization relies on instrumentation for absorption spectroscopy, fluorescence spectroscopy, and mass spectrometry. The various instrumental analyses require companion protocols for sample preparation of varying degrees of complexity. The choice of method depends on the scientific goal—to quantitate the production of tolyporphins from a culture that is a known producer of tolyporphins (i.e., *what quantities of tolyporphins in total are made?*) is quite different from surveying unknown cultures to identify those that produce tolyporphins (i.e., *does this strain produce any tolyporphins?*).

**Figure 30:**
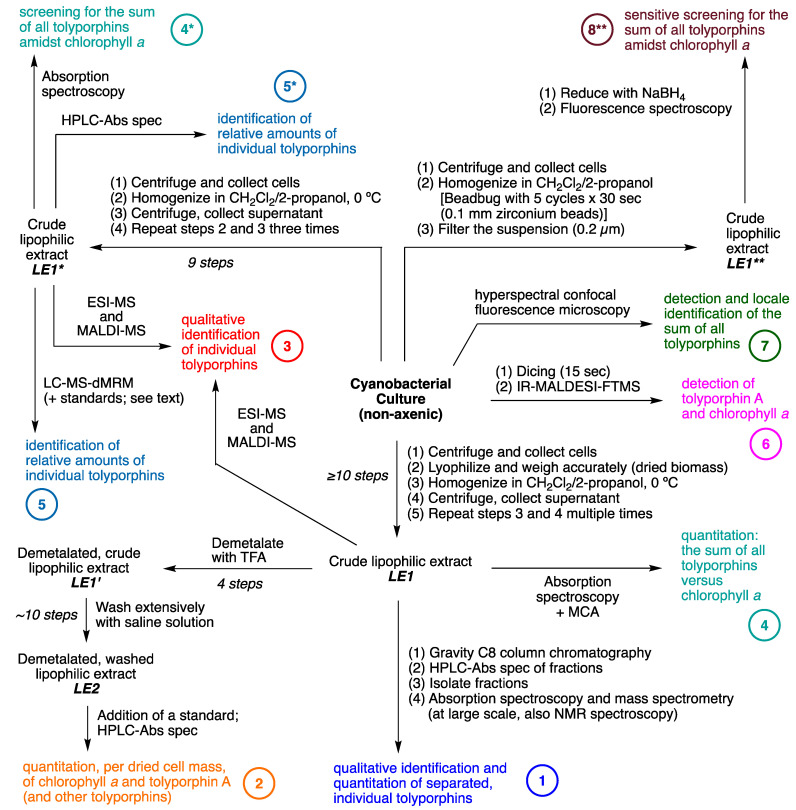
Processes for analysis of cyanobacterial samples. Numbers shown in circles are listed in chronological order of development and are described in Section 10.1, Section 10.2, Section 10.3, Section 10.4, Section 10.5, Section 10.6, Section 10.7 and Section 10.8. The initial method for generating the chief lipophilic extract (***LE1***) has undergone process simplification, which has generated lipophilic extracts, denoted by LE1* or LE1** (see the text).

A comprehensive summary of the methods developed and applied as part of the program to detect, identify, and quantitate tolyporphins is provided in Figure 30. The processes outlined in the diagram are described in depth in the following subsections. All of the methods have been applied to HT-58-2 cultures. Selected methods also have been utilized in the search for new cyanobacterial producers of tolyporphins.

### 10.1. Process 1

Process 1 is for the qualitative identification of individual tolyporphins upon separation by HPLC and subsequent quantitation of individual tolyporphins by gravimetry. This first process was employed at the University of Hawaiʻi to isolate tolyporphins from large-scale cultures (the 1995 culture [45] and the 2017 culture [69]). The lipophilic extract is termed ***LE1***. The process also has been used for small-scale (2–50 mL) cultures. The process at small scale entails (1) centrifugation to collect the mass of cells; (2) lyophilization (i.e., freeze-drying), followed by weighing to determine the dried cell mass, which is important for assessments of relative yields of tolyporphins; (3) homogenization in the mixed organic solvent dichloromethane and 2-propanol, which is typically carried out using mechanical treatment and at 0 °C to avoid thermal degradation of natural products; and (4) centrifugation to separate the organic extract from cellular debris. The latter two steps were typically carried out several times, until no green pigmentation remained in the cellular debris. In this manner, generating ***LE1*** would typically require at least 10 steps.

To isolate individual tolyporphins required fractionation of ***LE1***. The process entailed chromatography on a reversed-phase (C8) column with gravity-induced elution, followed by HPLC coupled with absorption spectral detection or mass spectral detection. Isolation of fractions was followed by a battery of characterization techniques. At small scale, the techniques include absorption spectroscopy and mass spectrometry, whereas at larger scale, NMR spectroscopy was applied. In general, identification of the individual tolyporphins has been achieved by application of absorption spectroscopy, NMR spectroscopy, and mass spectrometry. The methods of mass spectrometry have included electrospray ionization mass spectrometry (ESI-MS) and other high-resolution methods for accurate mass determination, as well as matrix-assisted laser desorption ionization mass spectrometry (MALDI-MS), which affords low-resolution analysis. The results concerning individual tolyporphin yields are shown in Table 4 [69].

### 10.2. Process 2

**Figure 31:**
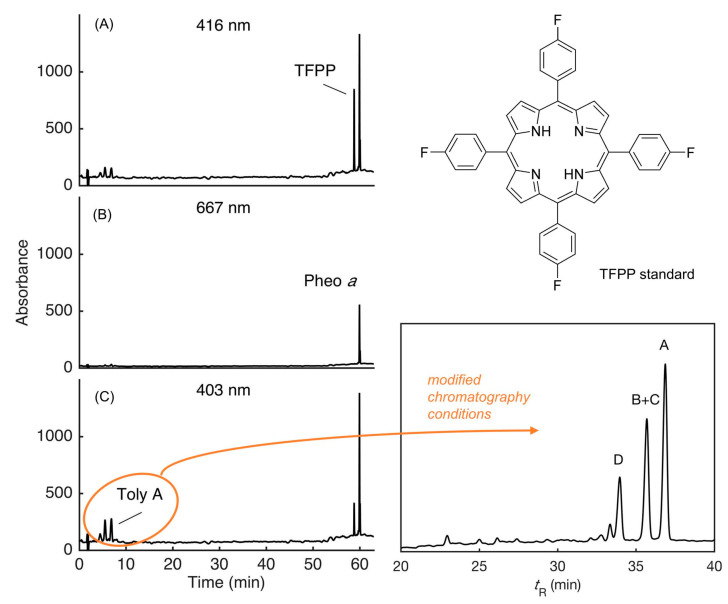
HPLC traces for the lipophilic extract (***LE2***) from small-scale cultures. Left column: Absorption detection at distinct wavelengths: (**A**) TFPP, 416 nm; (**B**) pheophytin *a*, 667 nm; and (**C**) tolyporphin A, 403 nm. Right column: Absorption detection at 401 nm. The assignment of tolyporphins A, B + C, and D was achieved by applying MALDI-MS to isolated fractions [69]. Adapted with permission from ref [69]; 2018, John Wiley and Sons, Inc.

Process 2 is for quantitation of tolyporphin A and chlorophyll *a* from a given culture size. By contrast, whereas Process 1 afforded quantitative data concerning individual tolyporphins, no data were obtained concerning chlorophyll *a*. Moreover, Process 1 was suited for large-scale cultures. For quantitative analysis of tolyporphin A and chlorophyll *a* per the dried cell mass in small-scale (2–50 mL) cultures, an elaborate purification protocol was developed following from ***LE1***. Treatment with trifluoroacetic acid (TFA) was carried out to convert chlorophyll *a* to the free base form, pheophytin *a*. This process was effectuated to avoid any adverse effects due to adventitious demetalation during the course of analysis; for example, absorption spectroscopic quantitation of a partially demetalated sample would be complicated, given that chlorophyll *a* and pheophytin *a* exhibit distinct spectral features. Magnesium chelates are more polar than the free base species; the magnesium also is readily removed upon exposure to weak acid (including silica, as used particularly in chromatography) [75]. Because the magnesium chelate and the free base species afford distinct chromatographic properties, the complete demetalation of the magnesium chelate (i.e., conversion of chlorophyll *a* to pheophytin *a*) was considered advisable. An internal standard was added to ensure quantitative accounting, even in the presence of sample losses, thereby affording lipophilic extract ***LE2***. Sixteen internal standards were examined to find one that was suitable, which was *meso*-tetrakis(4-fluorophenyl)porphyrin (TFPP), for which calibration curves were established. HPLC analysis with absorption spectral detection was carried out using a value of the molar absorption coefficient (ε) of 110,000 M^−1^cm^−1^ for all dioxobacteriochlorin-type tolyporphins (which includes tolyporphin A). Representative HPLC traces are shown in Figure 31 [69]. The process enabled quantitation of tolyporphin A and the total chlorophyll *a* relative to the amount of dried cell mass under various growth conditions [69].

Information concerning the relative amounts of other tolyporphins was obtained unintentionally as a consequence of use of HPLC. The relative amounts of dioxobacteriochlorin-type tolyporphins were assessed on the assumption of identical molar absorption coefficients (at 401 nm) upon HPLC elution and detection by absorption spectroscopy. Accordingly, the percentage of tolyporphin A among all dioxobacteriochlorin-type tolyporphins was estimated to be 40%.

The generation of extract ***LE2*** is painstaking, requiring ~25 steps, yet affords quantitation of chlorophyll *a* and tolyporphin A (and selected other individual tolyporphins) per the amount of dried cell mass. The technique can be applied to small-scale cultures. The only assumption for quantitation of diverse tolyporphins is the value chosen for the molar absorption coefficient (ε), as described in Section 9.2. It warrants emphasis that the ordinary measurements in a microbiological laboratory of optical density, cell counts, or colony counts could not be used to accurately measure growth given the filamentous cyanobacterium. Moreover, measurements of cell mass do not necessarily reflect or even correlate with the amount of the cyanobacterium alone given the non-axenic nature of the culture. Finally, perspective accrues from noting that methods for extraction of pigments (e.g., chlorophylls, carotenoids) from filamentous cyanobacteria (and algae) remain an ongoing area of investigation; chief concerns are quantitative removal of all pigments without causing pigment degradation [125].

**Figure 32:**
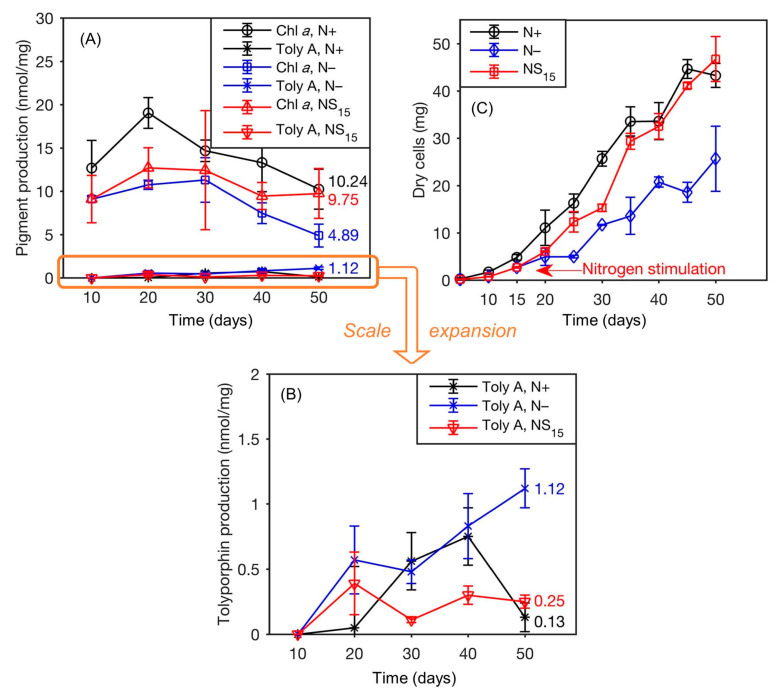
Production in 25 mL cultures of chlorophyll *a* and tolyporphin A (**A**), tolyporphin A in expanded display (**B**), and dried cell mass (**C**). The growth media included BG-11 (N+, black), BG-11_0_ (N–, blue), or initially in BG-11_0_ then changed to BG-11 after 15 days (NS_15_, red). Errors were estimated upon standard deviation on the basis of three replicates [69]. Adapted with permission from ref [69]; 2018, John Wiley and Sons, Inc.

Process 2 was applied to assess production of tolyporphin A and chlorophyll *a* (via the derivative pheophytin *a*) over time under various growth conditions [69]. The HT-58-2 isolate was grown in 25 mL cultures in BG-11 (designated N+ for clarity); in BG-11_0_ (N−); or initially in BG-11_0_, but changed to BG-11 at day 15 to study nitrogen stimulation (via the presence of nitrate) of cell growth (NS_15_). The key results are as follows:

(1) Regardless of conditions or the growth period, the HPLC peak due to tolyporphin A was the highest among all discernible tolyporphins. The percentage of tolyporphin A among all tolyporphins in the small-scale culture was ~40%, to be compared with 55% in large-scale growth experiments [45].
(2) The yield of tolyporphin A was lower than that of chlorophyll *a* under all growth conditions (Figure 32, panels A and B). Note that panel B is an expansion of the tolyporphin A data in panel A.
(3) More chlorophyll *a* was produced in N+ than in N− or NS_15_ during the 50-day period. Upon nitrogen stimulation at day 15, the production of chlorophyll *a* increased to 9.75 nmol/mg dry cells at day 50, which is comparable to that in N+ (10.2 nmol/mg) and greater than that in N− (4.89 nmol/mg). For units conversion, given the molecular weight of chlorophyll *a* of 893.5 Da, 10 nmol/mg corresponds to 8.95 mg/g or 0.895%, which is in accord with values given in Table 8.
(4) The yield of tolyporphin A was higher in conditions of N− than in N+ or NS_15_. The maximum yield of tolyporphin A was 1.12 nmol/mg, which was reached on day 50 in N−; the yield was approximately 10 times greater than for the sample in N+ (0.13 nmol/mg) or NS_15_ (0.25 nmol/mg), but still less than the yield of chlorophyll *a* in N− (4.89 nmol/mg) (Figure 32, panels A and B).

In summary, the increased yield of tolyporphins upon nitrogen stress (deprivation of soluble nitrate) comports with general phenomena of increased microbial production of secondary metabolites during nutrient limitation [127,128,129]. While valuable, acquisition of such data for HT-58-2 was exceptionally laborious due to the following: (1) the requirement to exhaustively extract the filamentous cyanobacterial cell mass to ensure complete removal of all tolyporphins and (2) the use of HPLC for quantitation. The laborious nature of Process 2 prompted exploration of more expedient methods of analysis.

### 10.3. Process 3

Process 3 is for the qualitative identification of individual tolyporphins without use of HPLC. To assess what tolyporphins are present in a mixture, the lipophilic extract ***LE1*** was examined directly by MALDI-MS and also by ESI-MS [69]. MALDI-MS provides a very effective tool for the analysis of tetrapyrrole macrocycles. Many tetrapyrrole samples can be analyzed without use of a matrix; one interpretation is that tetrapyrrole molecules, which are disc-shaped, alone in neat form tend to make an effective matrix [130,131]. Mass spectral analysis of tolyporphins has been carried out in the absence of a matrix [47]. The studies with MALDI-MS for tolyporphins described below employed a matrix of 1,5-diaminonaphthalene (DAN), which had been established previously for use with chlorophyll *a* [132].

**Figure 33:**
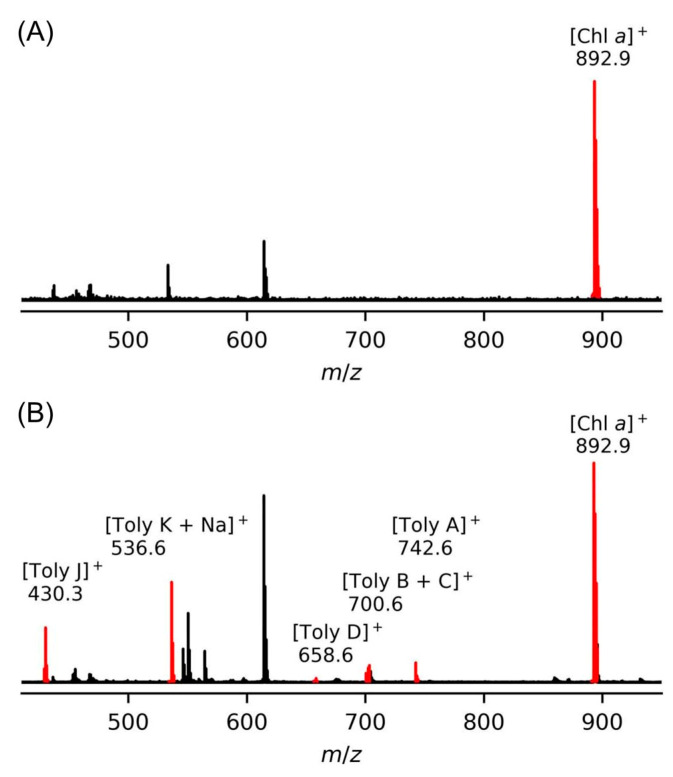
MALDI-MS (DAN matrix) spectra. (**A**) Authentic chlorophyll *a*. (**B**) HT-58-2 ***LE1*** [133]. Reprinted with permission from ref [133]; 2017, World Scientific Publishing Co Pte Ltd.

The mass spectral methods were applied to ***LE1*** without any chromatographic fractionation. The results from MALDI-MS analysis are shown in Figure 33. Authentic chlorophyll *a* was used for comparison with HT-58-2 ***LE1*** (panel A). In HT-58-2 ***LE1***, the peak of chlorophyll *a* was observed at *m*/*z* 892.9 (exact mass for C_55_H_72_MgN_4_O_5_), which is consistent within the error of the measurement with the spectrum of authentic chlorophyll *a* (*m*/*z* = 893.4) [69]. Peaks assigned to tolyporphin A (C_40_H_46_N_4_O_10_), tolyporphins B + C (C_38_H_44_N_4_O_9_), tolyporphin D (C_36_H_42_N_4_O_8_), tolyporphin J (C_24_H_22_N_4_O_4_), and the sodium adduct of tolyporphin K (C_30_H_32_N_4_O_4_Na) were also detected at *m*/*z* 742.6, 700.6, 658.6, 536.3, and 430.3, respectively (panel B). Studies with ESI-MS yielded the sodium or potassium cationized product for each tolyporphin. A complete set of data from laser desorption ionization mass spectrometry (LDI-MS, no matrix) of individual tolyporphins A–M (with isomeric pairs B + C; G + H; and L + M), as well as tolyporphin A diacetate, has been published [47]. (Tolyporphins N and O were not identified in this study. Tolyporphins N and O, which are isomers of L and M (Table 6), were first identified [112] nearly a decade after the work reported in this section was completed.) Strong positive ions and strong negative ions were reported in the two ion modes with relatively little fragmentation. Embracing the results obtained with tolyporphins and looking beyond, Prinsep and Puddick have articulated the broad value of the MALDI-MS method in searching for natural products in cyanobacteria [30].

The results of the mass spectral methods are summarized in Table 9. A subset of tolyporphins A–M was detected by MALDI-MS and also by ESI-MS, but the subsets were overlapping; each tolyporphin A–M was detected by one of the two methods. The absence of detection does not necessarily reflect an intrinsic deficiency of the method of analysis, but may be limited by insufficient sample quantity in the mixture. Absorption spectroscopy and again MALDI-MS were applied to fractions obtained by HPLC separation. The results by the mass spectral methods are qualitative, not quantitative, but provide a valuable and expeditious method for analysis.

The mass spectral examination also could be carried out with a simplified lipophilic extraction. For the objective of qualitative analysis, there is no reason to lyophilize and accurately weigh the dried cell mass as specified to generate ***LE1***. This step is particularly tedious and generally requires a 25 or 50 mL culture versus a simpler 2 mL culture. The lipophilic extract prepared without lyophilization and determination of dried cell mass is termed LE1* (Figure 30). Hence, mass spectrometric analysis can be implemented on lipophilic extracts ***LE1*** and LE1*. In summary, MALDI-MS offers the advantages of small sample size, easy sample preparation, fast analysis, and implementation of multiple samples in parallel. The disadvantages, however, are (1) lack of amenability to quantitation and (2) instrumental resolution precludes unambiguous assignment of ion masses to molecular compositions. Hence, the peak assignments stated above are consistent with the species, but not alone sufficient for unambiguous assignment. ESI-MS offers the advantages of sufficiently high resolution to achieve accurate mass determination of tolyporphin members, but suffers from low sensitivity and can be thwarted by the presence of non-ionizable constituents in the sample (e.g., salts), which together may limit utility for certain samples of tolyporphins.

### 10.4. Processes 4 and 4*

Process 4 is for the quantitation of the sum of all tolyporphins without chromatographic fractionation. In many cases, one wants to know the total quantity of tolyporphins, but knowledge of the distribution and yields of individual species is less important. In such cases, the absorption spectrum of ***LE1*** can be recorded directly without further fractionation [69]. The resulting absorption spectrum reflects all tolyporphins present. Process 4 enables quantitation of the sum of all tolyporphins, quantitation of chlorophyll *a*, and determination of the quantity of both per the amount of originating dried cell mass. Such quantitation is only achievable, however, in cases where the quantity of tolyporphins is comparable to that of chlorophyll *a*, as described in the following.

The challenges to use of absorption spectroscopy to screen pigments in biomass are well known and chiefly arise from the large absorption due to chlorophylls [134]. The absorption spectra of the cell suspensions of cyanobacterial strains show broad bands [135], hence absorption spectral detection of tolyporphins in the presence of chlorophyll *a* requires examination of the lipophilic extract in an organic solvent.

**Figure 34:**
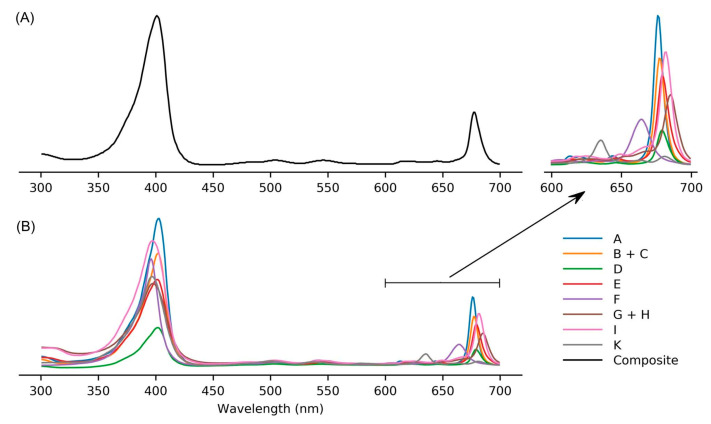
Composite absorption spectrum (**A**) of tolyporphins A–I and K generated from individual constituents (**B**) on the basis of the mole fractions. The expansion in panel C shows the challenge associated with spectral discrimination of individual constituents.

To quantitate the total amount of tolyporphins, the following method was employed. First, a simulated composite spectrum was generated. The simulated composite spectrum was created by summing the spectra of all tolyporphins weighted by the mole fraction present, where the latter is given by the quantity of individual tolyporphin members isolated upon purification. In this case, the quantities used were those obtained from the 2017 culture (Figure 26, Table 4) [69]. In that isolation, the tolyporphins detected were A–I (dioxobacteriochlorin-type) and K (oxochlorin-type). The peak maximum for each dioxobacteriochlorin-type tolyporphin is as follows (in nm; see Table 7 and Figure 28): A (676), B and C (677), D (679), E (679), F (664), G and H (685), and I (682). Tolyporphin K makes no contribution to the Q_y_ band of the dioxobacteriochlorin-type tolyporphins; hence, this method is suitable only for the dioxobacteriochlorin-type tolyporphins. The individual spectra, weighted by mole fractions of components obtained in the large-scale (2017) isolation, are overlaid in Figure 34. The composite spectrum also is displayed. The peak of the composite spectrum is at 677 nm. While the value of the molar absorption coefficient (ε) of each dioxobacteriochlorin-type tolyporphin was set at 110,000 M^−1^cm^−1^, the value of ε of the Q_y_ band of the composite spectrum was 51,530 M^−1^cm^−1^ owing to the spread in peak maxima across the nine tolyporphin constituents.

Understanding the complexity of the use of absorption spectroscopy is perhaps more apparent if approached in the direction opposite from generating the composite spectrum from individual constituents; thus, take the observed spectrum from ***LE1*** and deconvolve the Q_y_ region into the distinct constituents. The presence of peak maxima in a congested region of <10 nm for at least nine components (each with a band having fwhm of ~10 nm) makes quantitation of individual components infeasible. This issue is the problem [136] of “aliasing”—the close similarity of the absorption spectra of the various distinct absorbers thwarts detection and identification of the individual components [69]. Another view of the problem is that [75] “the relationship between structures and spectra is unidirectional and nonexclusive (what mathematicians would refer to as surjective): one structure gives rise to one spectrum, but a given spectrum could stem from a number of distinct structures”. Note again that the porphyrin tolyporphin P and the oxochlorins tolyporphins K, Q, and R are not accounted for through use of the composite absorption spectrum at 677 nm. The remainder of the text in this section pertains to dioxobacteriochlorin-type tolyporphins alone, unless noted otherwise.

**Figure 35:**
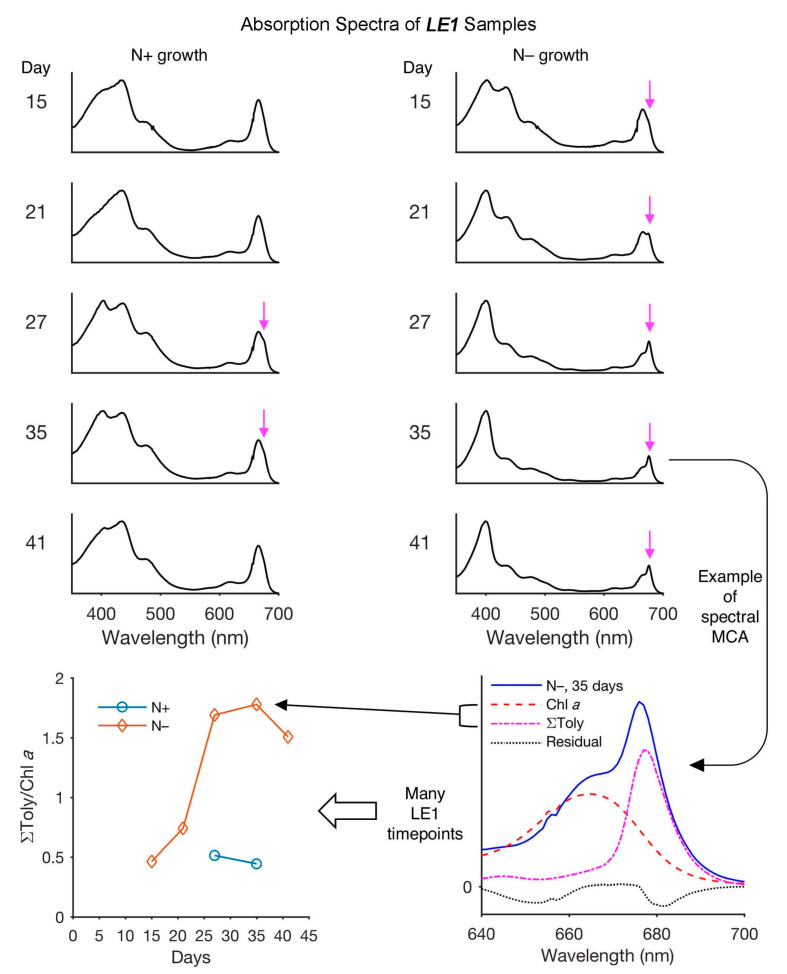
Absorption spectra of ***LE1*** samples over time from HT-58-2 grown in media that contains nitrate (N+) or lacks nitrate (N−). The vertical magenta arrow shows the emergence of the band of dioxobacteriochlorin-type tolyporphins. MCA analysis (shown for the 35-day timepoint in N− media) quantitates the relative amount of dioxobacteriochlorin-type tolyporphins and chlorophyll *a* (lower right). A number of such timepoints gives rise to the growth curves of dioxobacteriochlorin-type tolyporphins versus chlorophyll *a* (lower left) [69]. Adapted with permission from ref [69]; 2018, John Wiley and Sons, Inc.

The HT-58-2 culture was grown in BG-11 (contains nitrate, designated N+) or BG-11_0_ (devoid of nitrate, designated N−) with continuous illumination over 45 days. The cultures were very small scale (2 mL), which was possible because the weight of the dried cell mass was not necessary given direct absorption spectral interrogation of ***LE1*** (versus the use of 25 or 50 mL cultures for the aforementioned HPLC analyses to generate the data shown in Figure 32). Timepoints were collected by sacrificing cultures at specified times and performing an extraction to generate ***LE1***, which were examined by absorption spectroscopy (Process 4 in Figure 30). In the presence of nitrate, at days 27 and 30, there appears a long-wavelength shoulder on the strong Q_y_ band of chlorophyll *a* (Figure 35). The emerging shoulder is illustrated with a vertical magenta arrow when visibly evident, namely, at timepoints from 27 days onward. The Q_y_ absorption band of chlorophyll *a* is strong (ε = 89,800 M^−1^cm^−1^) [137] and ordinarily a quite distinct marker in the red region. In this case, however, the Q_y_ band of tolyporphins overlaps with the Q_y_ band of chlorophylls. Application of multicomponent analysis (MCA) [138] with two component spectra—the tolyporphins composite spectrum and the spectrum of chlorophyll *a*—enabled the quantity of the two to be dissected. The quantity of tolyporphins was ~1/2 that of chlorophyll *a*, as is shown in the lower left panel of Figure 35.

Growth in N– medium elicited a more profound effect, where the absorption due to tolyporphins was quite visibly evident during the period of 3–6 weeks. The quantity of tolyporphins exceeded that of chlorophyll *a* by up to 1.8-fold (Figure 35, lower left panel).

The MCA method has limitations and must be applied with care. A key issue is that the residual following application of MCA must be less than that of either component, else the analysis is not reliable. The MCA result is shown for one example with the ***LE1*** sample from one timepoint. In short, the MCA method is applicable only in those instances where the relative quantity of tolyporphins is substantial, beginning to rival that of chlorophyll *a*.

The increased production of tolyporphins relative to that of chlorophyll *a* during growth under nitrogen deprivation is shown in Figure 32 and in Figure 35. The results cohere qualitatively, but not quantitatively. The results obtained by HPLC fractionation (Figure 32) show tolyporphin A to be at most 0.23 times that of chlorophyll *a*, whereas those by absorption spectroscopy (Figure 35) show all tolyporphins to be up to 1.8 times that of chlorophyll *a*. Given a ratio of all tolyporphins to tolyporphin A of 2.3, the implied ratio of all tolyporphins by HPLC fractionation is maximally 0.53, to be compared with 1.8 by direct absorption spectroscopy. Several possible interpretations of this disparity are as follows:

(1) The results obtained by HPLC fractionation were obtained with 25 mL cultures, whereas those by absorption spectroscopy were with 2 mL cultures. Different scales of growth may alter the inherent production of chlorophyll *a* and tolyporphins.
(2) The HPLC fractionation requires more extensive sample processing of ***LE1*** (Process 2, Figure 30), whereas absorption spectroscopy directly interrogates ***LE1*** (Process 4, Figure 30). Components may be lost upon performing HPLC. Any yield (of minor components) determined by gravimetry of minute samples may be fraught, just as determination of molar absorption coefficients from minute samples of tetrapyrroles has proven historically to be a precarious endeavor.
(3) There may be compounds other than tolyporphins and chlorophyll *a* that absorb in the Q_y_ region and make MCA inaccurate.
(4) The mole fraction-weighted basis of isolated samples to generate the composite spectrum depends on the uniform representation of tolyporphins in ***LE1*** from the cyanobacterial sample. The extraction may not be uniform or degradation could occur (particularly of the acetates and the *C*-glycosides). The incomplete or skewed extraction may not be a significant concern for examination of ***LE1*** by absorption spectroscopy, but the generation of the composite absorption spectrum relies not only on extraction, but also accurate fractionation to determine the mole fraction. While a somewhat circular process, possible pitfalls are likely mitigated by the similar distribution of tolyporphin members under various culture conditions, and the close similarity of the absorption spectra among all dioxobacteriochlorin-type tolyporphins, as described in ensuing paragraphs.
(5) The accurate quantitative extraction of chlorophyll from cells presents challenges that have long been recognized, including demetalation, as well as enzymatic degradation [139,140].

Regardless of culture size or analysis methods, the minimum conclusions are that the amount of tolyporphin A at early growth stages in the presence of soluble nitrogen is tiny (~1/600th) versus that of chlorophyll *a*, whereas the amount of all tolyporphins may approach, if not slightly exceed, that of chlorophyll *a* upon deprivation of soluble nitrogen at long (> 30 days) growth times. The presence of tolyporphins at such a high level relative to that of chlorophyll *a* under stress of nitrogen deprivation suggests an interesting, albeit unresolved, physiological function of tolyporphins [69].

**Figure 36:**
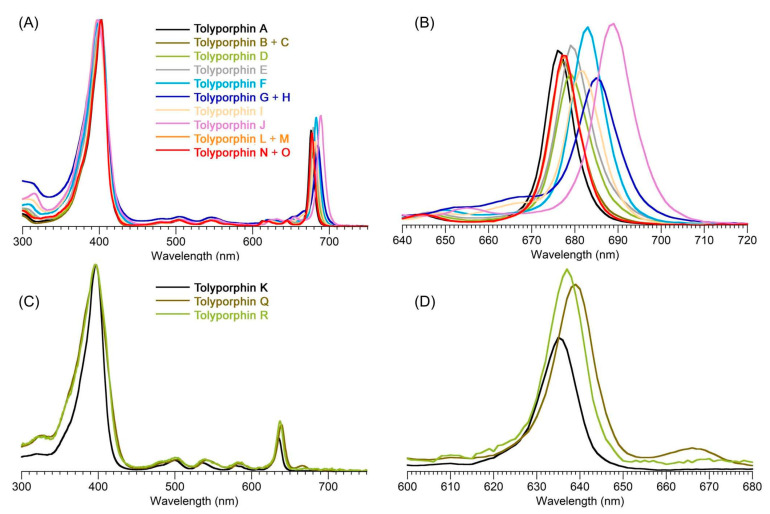
Overlay of spectra of members of a given class of tolyporphins. Dioxobacteriochlorin-type tolyporphins are shown (**A**, normalized), along with spectral expansion (**B**). Oxochlorin-type tolyporphins are shown (**C**, normalized), along with spectral expansion (**D**) [104].

Given the value of direct examination by absorption spectroscopy of a lipophilic extract from the culture, generation of the composite spectrum of tolyporphins was re-examined [104]. The composite spectrum shown in Figure 34 included only those tolyporphins obtained upon fractionation of the large-scale 2017 culture: A–I and K. Since then, known tolyporphins L and M [47] were characterized [48], and new tolyporphins N–R have been discovered [48]. Tolyporphins K, Q, and R are oxochlorins, whereas tolyporphin P is a porphyrin (Figure 6 and Table 6). The overlaid spectra of the dioxobacteriochlorin-type tolyporphins and oxochlorin-type tolyporphins are shown in Figure 36 [104].

**Figure 37:**
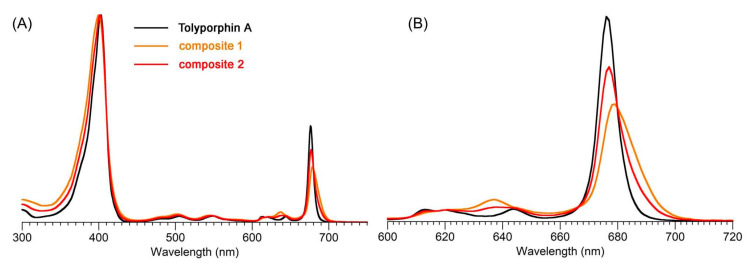
(**A**) Normalized overlay of the absorption spectrum of tolyporphin A (black), the composite spectrum of tolyporphins A–R in equal ratio (orange), and the composite spectrum of tolyporphin A = 50% with the sum of B–R = 50% (red). (**B**) Expanded Q-band region for the same spectra as in panel A [104].

A composite spectrum of all known native tolyporphins (A–R) is shown in Figure 37. Two compositions were prepared. In one case (composite 1), the mole fraction of each tolyporphin was identical, 5.55% for each A–R, which is shown as the orange trace. For a second case (composite 2), tolyporphin A was set at 50%, and the remaining 17 tolyporphins constituted the remaining 50%, which is shown in the red trace. The spectra are compared with that of tolyporphin A alone. In this work, uniform molar absorption coefficient (ε) values were applied: 100,000 M^−1^cm^−1^ for the Q_y_ band of tolyporphins A–J and L–O; 35,000 M^−1^cm^−1^ for the Q_y_ band of tolyporphins K, Q, and R; and 14,500 M^−1^cm^−1^ for the Q_y_(1,0) band of tolyporphin P. The inclusion of a larger set of tolyporphins causes the long-wavelength absorption band to broaden, as seen in going from pure tolyporphin A, to 50% tolyporphin A (composite 2), to all tolyporphins present equally (composite 1).

Finally, when the goal is to assess whether tolyporphins are present or not, and quantitation of the yield of tolyporphins relative to the dried cell mass is not important, a simpler process of lipophilic extraction can be employed. The steps of cell mass lyophilization required to generate ***LE1*** are omitted, and the lipophilic extract is obtained following cell homogenization. The resulting lipophilic extract, termed LE1*, can be employed directly for absorption spectral analysis. The use of LE1* for absorption spectral examination is shown as Process 4* in Figure 30.

### 10.5. Processes 5 and 5*

**Figure 38:**
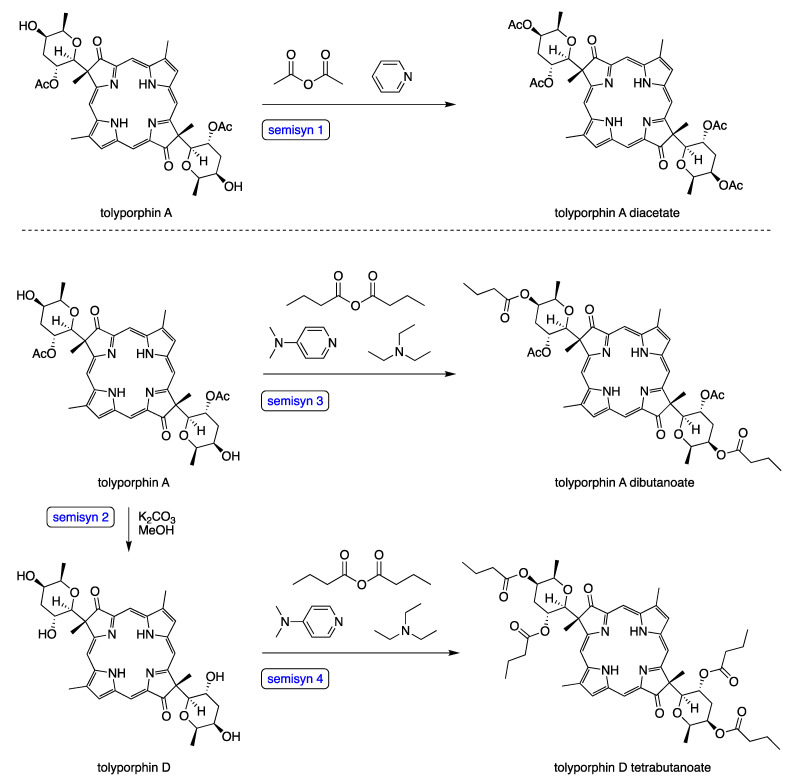
Semisynthetic modifications of native tolyporphins: semisyn 1 [20]; semisyn 2 [40,104]; semisyn 3 [104]; and semisyn 4 [104].

Process 5 is for the identification of relative amounts of individual tolyporphins. For the purpose of assessing the distribution of tolyporphins, and their relative quantity, but not their amount quantitatively relative to the dried cell mass, the new method of dynamic multiple reaction monitoring (dMRM) can be applied. The method requires separation of the constituents by liquid chromatography (LC), accompanied by mass spectral (MS/MS) analysis. The method enables effective integration of the total elution peak attributed to a given molecular entity. Stock solutions are required. In this case, the standards were tolyporphin A, tolyporphin A dibutanoate, and tolyporphin D tetrabutanoate (Figure 38). The synthetic approach extends that of Prinsep et al., who, as part of the initial structure elucidation, treated tolyporphin A with acetic anhydride in the presence of pyridine to yield tolyporphin A diacetate (which is shown as **Wang-1** in Figure 5) [20]. The derivatization or semisynthesis is labeled “semisyn 1” in Figure 38. Tolyporphin A, which contains two *O*-acetyl groups and two hydroxy groups, was subjected to hydrolysis to yield the corresponding tetrol, which is equivalent to tolyporphin D (“semisyn 2”) [40,104]. Tolyporphin A dibutanoate was prepared by treatment of native tolyporphin A with butanoic anhydride in the presence of triethylamine and 4-dimethylaminopyridine (“semisyn 3”) [104]. Similar treatment with butanoic anhydride in the presence of triethylamine and 4-dimethylaminopyridine yielded tolyporphin D tetrabutanoate (“semisyn 4”) [104]. Each was prepared in small quantity (unspecified amount), without experimental procedures or reported characterization data other than accurate mass data [20,104]. Semisynthesis is a well-known practice with complex biomolecules and has been applied even with other bacteriochlorins (bacteriochlorophyll *a*) [141,142,143,144], but the conversions displayed here are nearly the entirety of those organic transformations that have been implemented with tolyporphins. More extensive semisynthesis with tolyporphins would likely require more facile access to workable quantities of pure members of the tolyporphins family.

**Figure 39:**
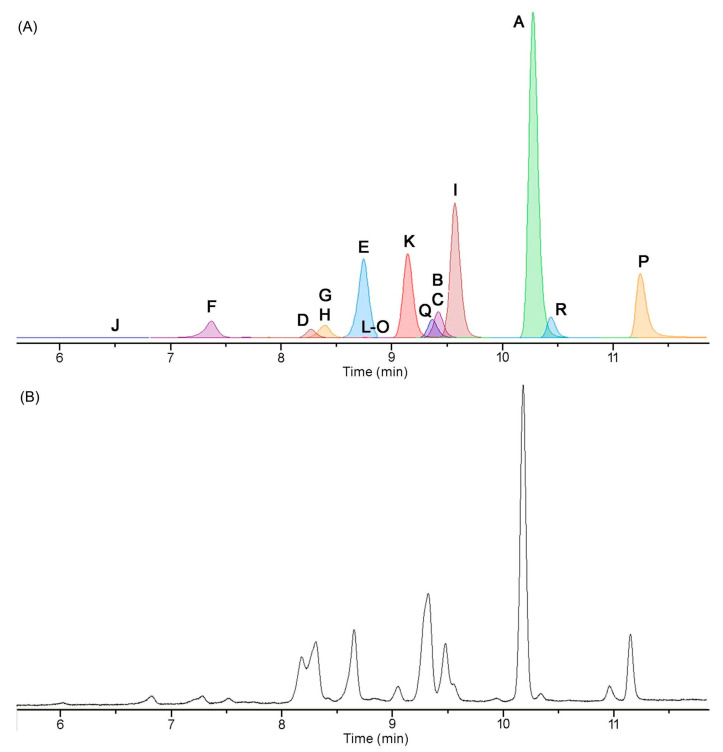
Representative chromatograms (C8 columns) of tolyporphins (LE1*) with mass spectral detection and assignment (**A**) or absorption spectral detection at 400 nm (**B**) [104].

One standard (tolyporphin A dibutanoate) was added to each harvested cell mass prior to extraction for quantitation of extraction efficiency. The other standard (tolyporphin D tetrabutanoate) was added to each sample vial prior to analysis for instrument calibration [104]. The addition of standards is not shown in the process flow in Figure 30. Equivalent ionization efficiencies are assumed, which, among the examples studied, appears to be reasonable. Application to extracts (LE1*) of the HT-58-2 culture afforded the chromatogram shown in Figure 39, panel A. The chromatogram can be compared with that obtained by HPLC coupled with absorption spectroscopy (Figure 39, panel B). The LC-MS-dMRM method enables identification of individual eluting tolyporphin species (except for isomers B,C; G,H; and L–O); by contrast, absorption spectroscopy does not allow unambiguous assignment given the similar spectra among many constituents. This powerful mass spectrometric method was applied to compare the effects of growth conditions (BG-11 versus A3M7 media) on the relative distribution of individual tolyporphins. The results are shown in Table 5 (see Section 8).

The lipophilic extract without gravimetric determination of the quantity of dried cell mass can also be analyzed by HPLC coupled with absorption spectroscopy (Process 5*). The method enables qualitative identification of tolyporphins on the basis of retention time, assuming one has in hand authentic standards of the individual tolyporphins. In the absence of known retention times, the three types of tolyporphins—porphyrin, oxochlorin, and dioxobacteriochlorin—can easily be categorized on the basis of absorption spectra, but distinctions within a given class (i.e., dioxobacteriochlorins or oxochlorins), for example, are subtle, in which case absorption spectra alone are not reliable for structure determination. With standards in hand, however, and known molar absorption coefficients (Table 7), the method also enables determination of relative quantities of members among the tolyporphins family, but not with respect to dried cell mass.

In summary, quantitating the total amount and individual distribution of tolyporphins appears simple, perhaps beguilingly so. Structures of similar polarity complicate chromatography; the similar, but non-identical, absorption of subsets of tolyporphins partially limits the use of absorption spectroscopy; and the presence of isobaric members renders mass spectrometry an incompletely decisive tool. The generation of ***LE1***, which enables determination of the quantitative yield of pigments versus dried cell mass, entails ≥10 steps and is laborious because the cell mass must be dried thoroughly for accurate weighing. The generation of ***LE2***, which enables quantitative analysis by HPLC of the individual tolyporphins, is even more stringent in entailing ~25 steps. Fewer steps are required for LE1*, but quantitative comparisons can only be made of total tolyporphins versus chlorophyll *a*, or relative measurements among individual tolyporphins. Underlying many measurements is a molar absorption coefficient, which is obvious in the methods that use absorption detection. The implicit reliance on a molar absorption coefficient is less evident in the LC-MS-dMRM method, but the internal standards (tolyporphin A dibutanoate and tolyporphin D tetrabutanoate) were prepared from tiny quantities, and the stock solutions were ultimately quantitated through use of an absorption measurement. Determination of the quantity of a tetrapyrrole analyte in a known volume is likely more accurate by absorption spectroscopy with use of a reliable molar absorption coefficient (Table 7) than concentration to dryness and gravimetric analysis; this is at least the case when the analyte is present in a minute amount. The next section concerns a simpler means of analysis, wherein quantitation is not required.

### 10.6. Process 6

**Figure 40:**
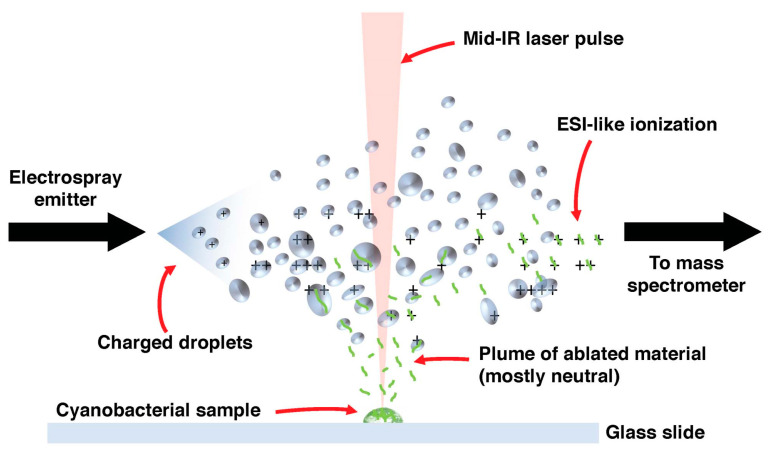
Schematic of IR-MALDESI-FTMS source for direct analysis of cyanobacteria [133]. Adapted with permission from ref [133]; 2017, World Scientific Publishing Co Pte Ltd.

Process 6 is for the detection of a dominant tolyporphin (e.g., tolyporphin A) in an unadulterated cyanobacterial culture. The technique known as IR-MALDESI-FTMS (Figure 40) is a recently developed analytical method that affords accurate mass measurements in the open air of samples that typically have undergone little or no preparation [145,146]. The IR-MALDESI-FTMS acronym stands for infrared matrix-assisted laser desorption electrospray ionization–Fourier-transform mass spectrometry. The technique relies on laser irradiation of a sample to ablate material (the “IR-MALD” part), followed by generation of ions in a charged plume (the “ESI” part) and subsequent passage of the ions into the mass spectrometer for analysis (the “FTMS” part). The technique combines some of the best features of traditional MALDI-MS and ESI-MS.

A study was carried out to explore whether IR-MALDESI-FTMS could be used to detect tolyporphins in intact cyanobacteria [133]. If so, the method might be suitable for screening cyanobacterial samples for the presence of tolyporphins; such a rapid screening approach would be valuable not only for addressing the qualitative effects of distinct growth conditions on a known producer (i.e., HT-58-2), but perhaps for searching for other producers of tolyporphins.

Cyanobacterial samples were treated with a mechanical disperser (30,000 rpm) for 15 s, which dices the filaments, but does not kill many of the cells. Subsequent vortexing led to a sample that was free flowing and readily pipetted. The combination of dicing and vortexing is referred to as shearing, given that large filaments are converted into smaller filaments. The process does not require chemical extraction and does not use a matrix for mass spectrometry.

**Figure 41:**
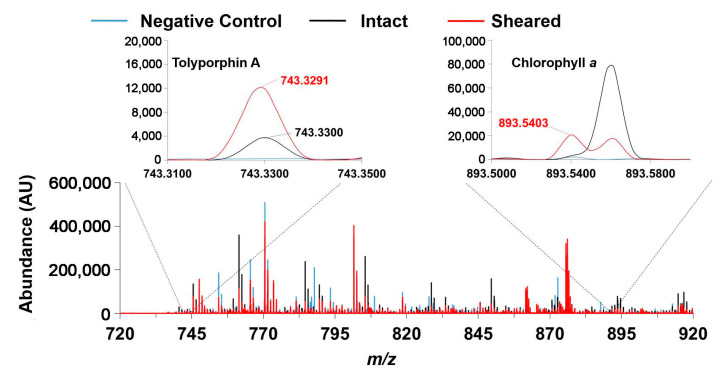
Overlay of mass spectra obtained upon IR-MALDESI-FTMS screening of samples from PCC 7120 (negative control, shown in blue), intact HT-58-2 (shown in black), and sheared HT-58-2 (shown in red). The two inset charts at top illustrate the normalized abundances (percentage of base peak in each mass spectrum) of the protonated molecular ions [(M + H)^+^] for tolyporphin A (*m*/*z*_theo_ = 743.3287) and chlorophyll *a* (*m*/*z*_theo_ = 893.5426). Each mass measurement was within 3 ppm of the theoretical value [133].

As an example, a sample of HT-58-2 following shearing was pipetted (5 μL) onto a glass slide for direct analysis by IR-MALDESI-FTMS [133]. A peak for tolyporphin A was observed only for the culture grown in BG-11_0_, not BG-11; the remaining results in this section concern the sample grown in BG-11_0_. A scan of *m*/*z* revealed a peak (*m*/*z* = 893.5403) corresponding to chlorophyll *a*, as well as a peak corresponding to tolyporphin A (*m*/*z* = 743.3291) (Figure 41). The theoretical observed mass (*m*/*z*_theo_) of chlorophyll *a* or tolyporphin A is 893.5426 or 743.3287, respectively. Analysis of a sample of intact HT-58-2 (no shearing) also yielded a signal for tolyporphin A, albeit of weaker intensity. A ~3-fold increase in the ion abundance of tolyporphin A in the sheared sample was observed compared to that of the intact sample. Analysis of a sample from the well-known cyanobacterium *Nostoc* PCC 7120 (also known as *Anabaena* UTEX 2576, SAG 25.82) did not show the presence of any tolyporphin A, as expected for this negative control. The mass spectra obtained from analysis of all three samples (PCC 7120, intact HT-58-2, and sheared/vortexed HT-58-2) are shown in Figure 41.

The overall analysis process is only partially destructive, as the sheared sample (even after illumination as part of the IR-MALDESI-FTMS technique) upon return to the culture medium yielded growth, indicating that numerous cells remained alive during the shearing process [133].

**Figure 42:**
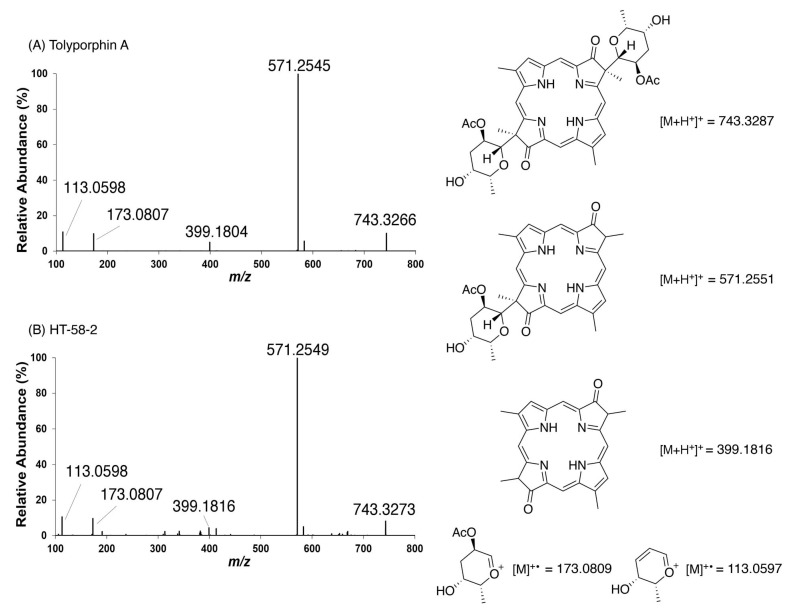
IR-MALDESI-FTMS validation with MS/MS analysis of tolyporphin A (*m*/*z*_theo_ 743.33) from an authentic standard (panel **A**) and a sample from HT-58-2 (panel **B**). Calculated masses of the fragment ions are shown at right [133]. Reprinted with permission from ref [133]; 2017, World Scientific Publishing Co Pte Ltd.

The presence of tolyporphin A in the cyanobacterial sample was further examined by MS/MS spectroscopy. In the authentic sample of tolyporphin A, the molecular ion [M + H^+^]^+^ at *m*/*z* = 743.3266 and fragment ions at *m*/*z* = 571.2545, 399.1804, 173.0807, and 113.0598 were detected (Figure 42, panel A). The fragment ions are assigned to loss of one *C*-glycoside moiety (*m*/*z* = 571.2545) or two *C*-glycoside moieties (*m*/*z* = 399.1804), with the former being the dominant ion in the spectrum for *m*/*z* > 100. In each case, the corresponding macrocycle was formed with loss of the oxocarbenium ion of the glycoside (*m*/*z* = 173.0807), which could undergo dehydration to form the α,β-unsaturated oxocarbenium ion (*m*/*z* = 113.0598). The structures and expected molecular ion masses of the parent tolyporphin A and fragments therefrom are shown in Figure 42, right column.

In the HT-58-2 sheared samples, all five peaks characteristic of tolyporphin A were detected, as in the authentic sample (Figure 42, panel B). The five peaks in the HT-58-2 sample also were in similar abundance to those in the authentic sample. Together, the data (1) strongly support the production of tolyporphin A in HT-58-2 and (2) illustrate the incisive capability of IR-MALDESI-FTMS for direct analysis of intact cyanobacterial specimens.

IR-MALDESI-FTMS is a highly efficient screening method for use with cyanobacteria, but there are some notable restrictions. First, multiple mass windows need to be examined to ensure detection of the various tolyporphins of distinct masses, especially if knowledge is not in hand concerning which tolyporphins are produced. Second, the reproducibility is quite low, as the laser focus may miss the filaments, taking into account, of course, that the filaments themselves are intrinsically heterogeneous. Third, the detection limit is high, which precludes application to samples that contain a small quantity of tolyporphins. Indeed, the samples from cultures of HT-58-2 grown in BG-11_0_ yielded positive results, whereas those in BG-11 did not, even though tolyporphins are known to be present in cultures grown in the latter medium. Fourth, unambiguous detection of a tolyporphin can only be assured upon detection of both the parent ion, as well as key molecular fragments. In short, the present technique does not yet appear suitable for application in widespread screening of diverse cyanobacterial cultures, unless the search is aimed at identifying substantial producers of a specific tolyporphin (i.e., of known mass). IR-MALDESI-MS has been used to measure analytes in plants, including *Artemisia annua* [147], *Arabidopsis thaliana* seedlings [148], fungal (*Magnaporthe oryzae*)-infected barley [149], and maize [150]; in plant products, including cucumbers [151] and cherry tomatoes [152]; and in surrogate samples related to plants, including β-carotene [146]. The IR-MALDESI-FTMS method remains under rapid development. The continued advances of the IR-MALDESI-FTMS method for analyzing complex samples may augur well for future screening studies of cyanobacteria.

### 10.7. Process 7

Process 7 is for the visualization of tolyporphins in cyanobacteria. Hyperspectral confocal fluorescence spectroscopy (HCFM) has been applied to identify the locale of tolyporphins in the cyanobacterium–microbial consortium (see Section 7) [106]. No sample preparation is required—no extraction, lyophilization, or gravimetry; instead, intact cells are imaged with light illumination and light detection. The method is applicable for live samples, as is IR-MALDESI-FTMS, but unlike IR-MALDESI-FTMS, can detect tolyporphins at low content, such as those grown in BG-11 media. On the other hand, HCFM effectively detects all tolyporphins of a given spectral type (e.g., dioxobacteriochlorins), whereas IR-MALDESI-FTMS—through use of MS windows, followed by MS/MS fragmentation methods—enables identification of the composition of individual tolyporphins. In general, HCFM seems both powerful and underutilized. HCFM has been shown to be an incisive tool for imaging the locale of one type of tetrapyrrole macrocycle in the presence of another, namely, tolyporphins in the presence of chlorophyll *a*, but is at the antipode of tools for *screening* for the production of tolyporphins. In short, HCFM enables detection of tolyporphins in the presence of chlorophyll *a* and identification of the location of the tolyporphins within the filamentous cyanobacteria. On the other hand, the spectral deconvolution steps required to identify location are burdensome for answering the simple yes/no question of whether tolyporphins are present. The next section concerns a means of more facile screening of cyanobacterial samples for tolyporphins.

### 10.8. Process 8**

Process 8** is for screening cultures for the presence of dioxobacteriochlorin-type tolyporphins. Fluorescence is well known to be an exquisitely sensitive detection technique, particularly for many tetrapyrrole macrocycles. Tetrapyrrole macrocycles display a characteristic series of absorption bands, many with relatively narrow fwhm, across the ultraviolet and visible spectral regions. For those that are fluorescent [119], emission typically occurs in the red (or near-infrared) region, where there is little interference due to ordinary UV-absorbing species. Tetrapyrroles invariably show behavior consistent with “Kasha’s rule” [153]—that all photochemistry emanates from the lowest vibrational level of the first singlet excited state—hence, regardless of wavelength of excitation (λ_exc_), the emission pattern is identical. While perhaps counterintuitive to the photosciences novitiate, the concept is central to understanding tetrapyrrole emission spectra and photochemical behavior [154]. The emission bands of tetrapyrrole macrocycles are characteristic in terms of wavelength and pattern.

**Figure 43:**
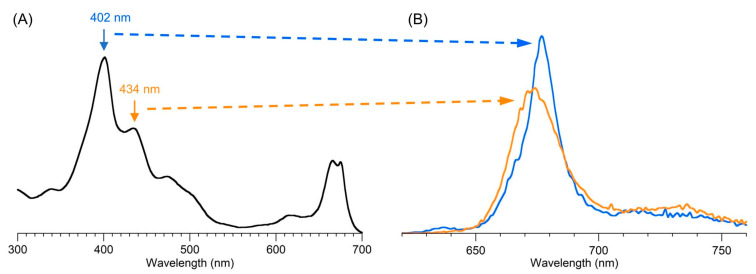
Spectra in methanol at room temperature of LE1* from HT-58-2 grown in BG-11_0_ media. (**A**) Absorption spectrum. (**B**) Fluorescence upon excitation at 402 nm (where tolyporphin A absorbs preferentially) and 434 nm (where chlorophyll *a* absorbs preferentially).

The absorption spectrum of LE1* from HT-58-2 grown in BG-11_0_ is shown in Figure 43, panel A. The characteristic peaks of chlorophyll *a* and tolyporphins are evident at defined sites in the broad spectrum. The spectrum of chlorophyll *a* and the spectrum of tolyporphin A are shown in Figure 13. Fluorescence spectroscopy (in a solution in a cuvette, not imaging of cells, as in HCFM) was examined for detection of tolyporphins in a lipophilic extract of cyanobacteria. The fluorescence spectra obtained with excitation at 402 and 434 nm are shown in Figure 43, panel B. Illumination at 402 nm preferentially excites tolyporphin A (and other dioxobacteriochlorin-type tolyporphins), whereas illumination at 434 nm preferentially excites chlorophyll *a*. There is no wavelength where one of the macrocycles absorbs to the exclusion of the other, precluding completely selective excitation. The fluorescence spectra upon illumination at the two wavelengths are very similar. Because the quantity of tolyporphins is typically much lower than that of chlorophyll *a* under most growth conditions, direct detection of tolyporphins in HT-58-2 with use of fluorescence spectroscopy did not appear to be viable.

Semisynthetic modification was pursued to create spectra where the two macrocycle types are distinguishable. The approach relied on knowledge of the effects of auxochromes—substituents appended to a conjugated π-system that alter the position of bands—on the spectral properties of tetrapyrroles. In chlorophyll *a*, the two key auxochromes (3-vinyl, 13-keto) are positioned coincident with the *y*-axis and collinear with the transition that gives rise to the Q_y_ band. The two auxochromes impart a bathochromic shift on the transition. In tolyporphin A, the two keto groups are aligned coincident with the *x*-axis and hence are perpendicular to the Q_y_ transition. The two keto groups impart a hypsochromic shift. The fact that auxochromes can cause shifts to lower energy is well known, but to higher energies is less appreciated. The evolution in understanding of auxochromes, since first identified in the late 19th century, has been outlined [73].

**Figure 44:**
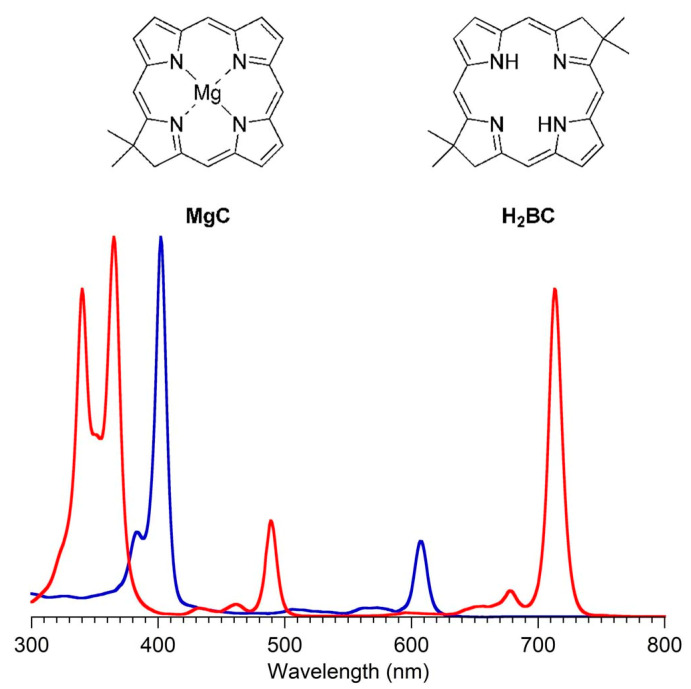
Absorption spectra in toluene at room temperature of the core chromophore of chlorophyll *a* (**MgC**, blue trace; λ_abs_ = 402 and 607 nm) and of tolyporphin A (**H_2_BC**, red trace; λ_abs_ = 340, 365, 489, and 713 nm). Both macrocycles are unadorned with peripheral substituents.

The absorption spectra of the core chromophore of chlorophyll *a* (**MgC**) [155,156] and of tolyporphin A (**H_2_BC**) [157] are shown in Figure 44. Both macrocycles are equipped with a gem-dimethyl group in each pyrroline ring to preclude adventitious dehydrogenation in an aerobic environment, but otherwise are unadorned with peripheral substituents. The macrocycle **MgC** is a chlorin, whereas **H_2_BC** is a bacteriochlorin. The Q_y_ band for **MgC** or **H_2_BC** appears at 607 nm or 713 nm, respectively. The Δλ of 106 nm for the sparsely substituted **MgC** and **H_2_BC** macrocycles can be compared with Δλ of 12 nm for chlorophyll *a* (665 nm) and tolyporphin A (676 nm). The small value of the latter Δλ stems from the counter-directional bathochromic and hypsochromic effects of the auxochromes in the respective native macrocycles versus the core benchmark compounds.

**Figure 45:**
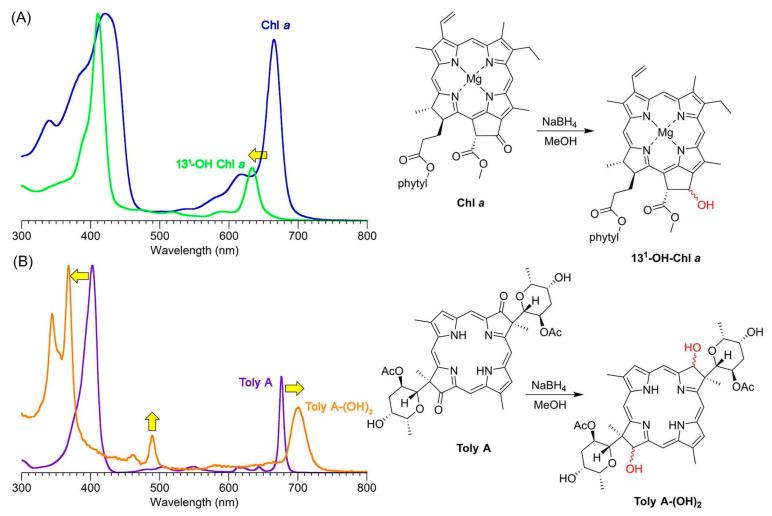
Left column: normalized absorption spectra in methanol at room temperature of (**A**) **Chl *a*** (blue line, Q_y_ = 665 nm) and **13^1^-OH Chl *a*** (lime line, Q_y_ = 634 nm), and (**B**) **Toly A** (purple line, Q_y_ = 676 nm) and **Toly A-(OH)_2_** (orange line, Q_y_ = 701 nm). The yellow arrows indicate spectral shifts upon chemical reduction; horizontal arrows indicate hypsochromic or bathochromic shifts, whereas the vertical arrow indicates a hyperchromic shift. Right column: reduction of keto groups in the two macrocycles [158]. Reprinted with permission from ref [158]; 2021, John Wiley and Sons, Inc.

Reduction of chlorophyll *a* (**Chl *a***) with NaBH_4_ converts the keto group to an alcohol and removes the π-conjugation, in which case the auxochrome potentiation is lost. The Q_y_ band position shifts hypsochromically from 665 nm (**Chl *a***) to 634 nm (**13^1^-OH Chl *a***), as shown in Figure 45, panel A, becoming more like an ordinary chlorin. Similar reduction of tolyporphin A (**Toly A**) with NaBH_4_ converts the two keto groups to alcohols, releasing the negative auxochromic effect. The Q_y_ band position shifts bathochromically from 676 nm (**Toly A**) to 701 nm (**Toly A-(OH)_2_**), as shown in Figure 45, panel B, becoming more like an ordinary bacteriochlorin. In short, the two Q_y_ bands move in opposite directions, with a net magnitude of ~60 nm. A further change is the shift of the B band of tolyporphin A to the 350–380 nm region, a region where the absorption of chlorophyll *a* is less pronounced.

**Figure 46:**
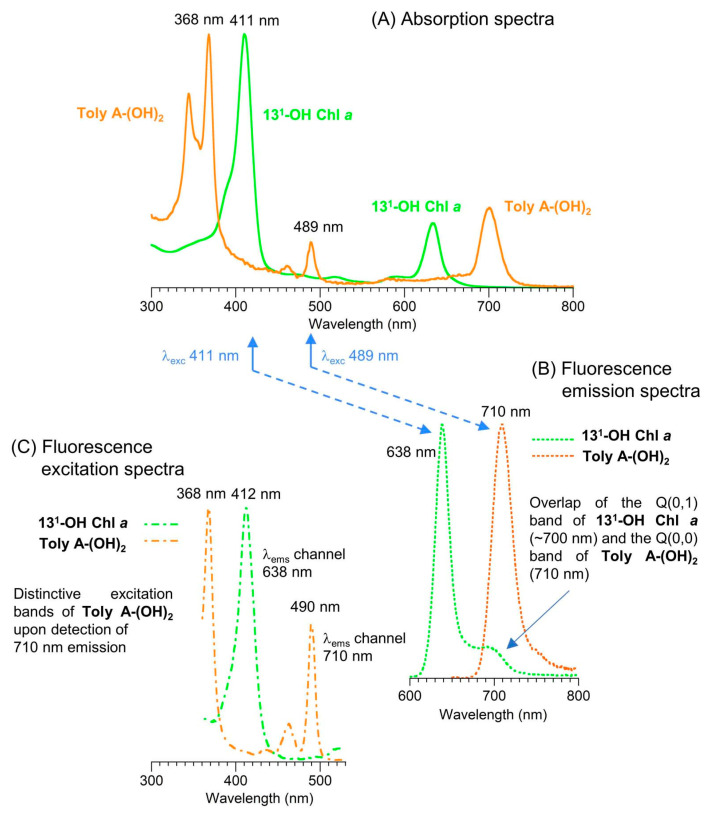
All spectra are in methanol at room temperature and are normalized. (**A**) Absorption spectra of **13^1^-OH Chl *a*** (green line, Q_y_ = 634 nm) and **Toly A-(OH)_2_** (orange line, Q_y_ = 701 nm). (**B**) Fluorescence emission spectra of **13^1^-OH Chl *a*** (green dashed line, λ_exc_ = 411 nm) and **Toly A-(OH)_2_** (orange dashed line, λ_exc_ = 489 nm). (**C**) Fluorescence excitation spectra of **13^1^-OH Chl *a*** (green dot–dashed line, λ_em_ = 638 nm) and **Toly A-(OH)_2_** (orange dot–dashed line, λ_em_ = 710 nm) [158]. Adapted with permission from ref [158]; 2021, John Wiley and Sons, Inc.

The spectra of the reduced products **13^1^-OH Chl *a*** and **Toly A-(OH)_2_** are overlaid in Figure 46, panel A, which shows the clear spectral distinctions. The fluorescence spectrum of each compound is shown in panel B. In each case, the Stokes’ shift is small, as is typical for tetrapyrrole macrocycles. In principle, fluorescence emission spectroscopy could be used for direct interrogation of reduced samples from the lipophilic extract of HT-58-2. A complication, however, is that there is usually a larger quantity, often a far larger quantity, of chlorophyll *a* than of tolyporphins. The emission spectrum of **13^1^-OH Chl *a*** shows a strong Q(0,0) band and a weaker Q(0,1) band. The latter intrudes in the spectral region of the fluorescence emission of **Toly A-(OH)_2_**. On the other hand, the fluorescence excitation spectrum of **Toly A-(OH)_2_**, achieved with the emission channel set at 710 nm, showed strong sharp peaks at 368 and 490 nm, as shown in panel C. The distinctive features of the fluorescence excitation spectrum of **Toly A-(OH)_2_** prompted use of this technique in a screening assay. In general, a *fluorescence emission spectrum* is obtained by applying a fixed wavelength of excitation (λ_exc_) and scanning across the emission wavelengths, thereby asking the question “what is the nature of the emitters that absorb at λ_exc_?” Conversely, a *fluorescence excitation spectrum* is obtained by applying a fixed wavelength of emission (λ_em_) and scanning across the excitation wavelengths, thereby asking the question “what is the nature of the absorbers that emit at λ_em_?” Both types of measurements are performed using a fluorescence spectrometer that is equipped with two monochromators (or other wavelength-selective device), one for emission and one for excitation.

**Figure 47:**
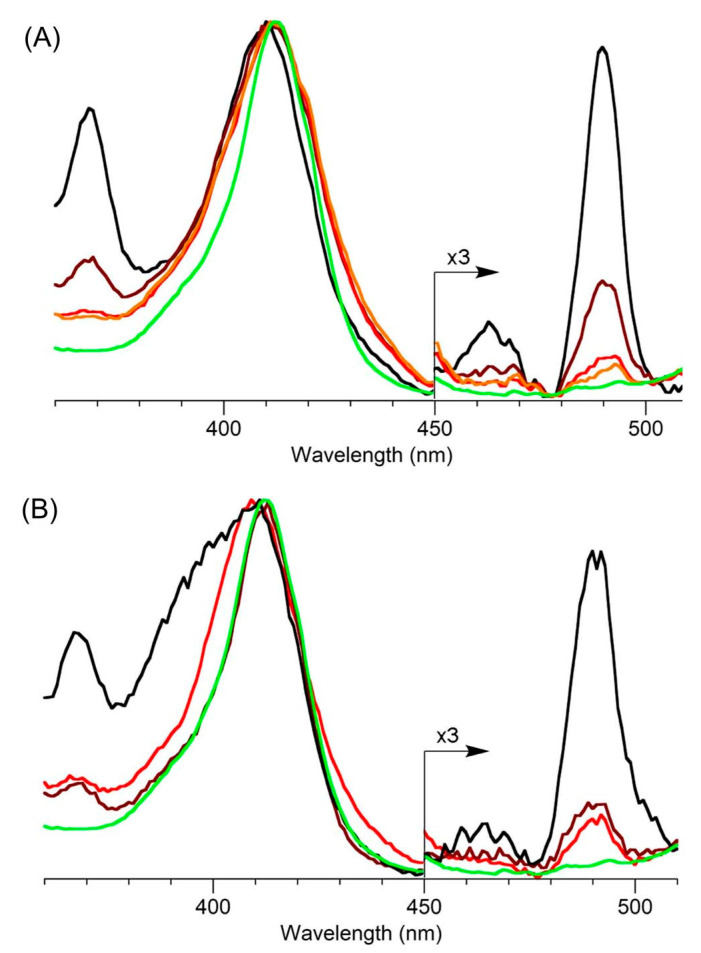
Fluorescence excitation spectra (normalized) in methanol at room temperature with λ_em_ 710 nm. (**A**) shows mock samples: reduced mixtures of ratios of **Chl *a*: Toly A** = 1:1 (black), 3:1 (brown), 9:1 (red), or 19:1 (orange). (**B**) shows experimental samples: extract of HT-58-2 (LE1**), followed by reduction with NaBH_4_. The reduced species from cultures **Ia** (black, 40 days in BG-11_0_), **Ib** (brown, 40 days in BG-11), and **Ic** (red, 20 days in BG-11). In both panels, the fluorescence excitation spectrum of **13^1^-OH Chl *a*** (green) is provided for comparison [158]. Adapted with permission from ref [158]; 2021, John Wiley and Sons, Inc.

Mock samples of mixtures of **Chl *a*** and **Toly A** (ratios of 1:1, 3:1, 9:1, 19:1) in methanol yielded the fluorescence excitation spectra (λ_em_ 710 nm) shown in Figure 47, panel A. The distinct bands of **Toly A-(OH)_2_** at ~373 nm and ~495 nm (corresponding to 368 nm and 490 nm in Figure 46) were clearly evident in the 1:1, 3:1, and 9:1 mixtures, and marginally evident in the 19:1 mixture (orange line). The ability to detect **Toly A** at the 5% level relative to chlorophyll *a* constitutes a substantial improvement over direct use of absorption spectroscopy (as in Processes 4 and 4*).

The assay was applied to HT-58-2 grown under distinct conditions, including BG-11_0_ for 40 days (**Ia**); BG-11 for 40 days (**Ib**); and BG-11 for 20 days (**Ic**). By absorption spectroscopic analysis, the presence of tolyporphins was beginning to become evident in **Ia**, but not in the other media. Lipophilic extracts were prepared from **Ia–Ic** in a simplified manner versus ***LE1*** or LE1*; the cells were suspended in CH_2_Cl_2_/2-propanol, followed by disruption via mechanically agitated beads. Filtration then afforded the lipophilic extract referred to as LE1** (Figure 30). The lipophilic extract LE1** from each of **Ia–Ic** was reduced with NaBH_4_, followed by fluorescence excitation spectroscopy; in each case, characteristic peaks (368, 490 nm) of hydroxy-tolyporphins were observed (Figure 47, panel B). The assay by reduction and fluorescence excitation spectroscopy enabled detection of tolyporphins in an unstressed culture at an intermediate growth period—growth in BG-11 media for 20 days (**Ic**)—which is not possible by the method of direct absorption spectroscopy (e.g., Processes 4 and 4*). The fluorescence excitation assay developed with use of tolyporphin A proved suitable for the HT-58-2 culture, which produces a constellation of tolyporphins (comprised of slightly less than 50% of tolyporphin A), as well as other pigments. In summary, the process of chemical reduction, followed by fluorescence excitation spectroscopy, can be implemented efficaciously for detecting tolyporphins in crude cyanobacterial samples of small quantity without fractionation.

## 11. HT-58-2 Genome—Genes for Tolyporphins

**Figure 48:**
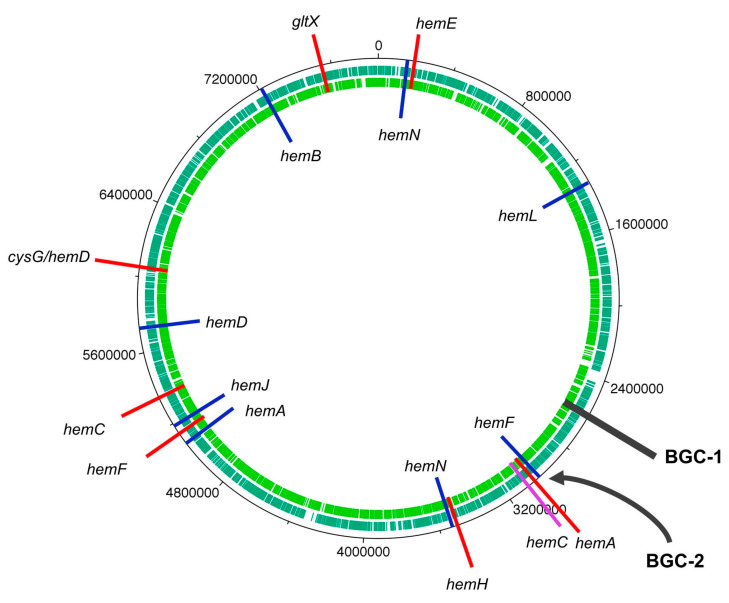
Genome of HT-58-2 cyanobacterium with annotations for heme genes and two putative biosynthetic gene clusters for tolyporphins. Different coloration is provided only for visual clarity [79,164].

The chromosome of the cyanobacterium in the HT-58-2 culture is circular, composed of double-stranded DNA, and contains 7,846,907 bp [79]. The sequencing and assembly of the genome is described in Section 6.2. The focus here is solely on genes possibly engaged in the biosynthesis of tolyporphins. All genes for the biosynthesis of the tetrapyrroles heme, chlorophyll *a*, and phycocyanobilin, and almost all the genes for the biosynthesis of cobalamin, were identified dispersed throughout the chromosome. Our understanding is that the clustering of *hem* genes is known in Gram-positive bacteria [159,160,161] whereas *hem* genes tend to be dispersed in Gram-negative bacteria (e.g., in *Escherichia coli* [162,163]). The biosynthesis begins with glutamic acid as the immediate precursor to δ-aminolevulinic acid (Figure 11). The genome of the HT-58-2 cyanobacterium annotated with heme biosynthesis genes is shown in Figure 48 [79,164].

**Figure 49:**
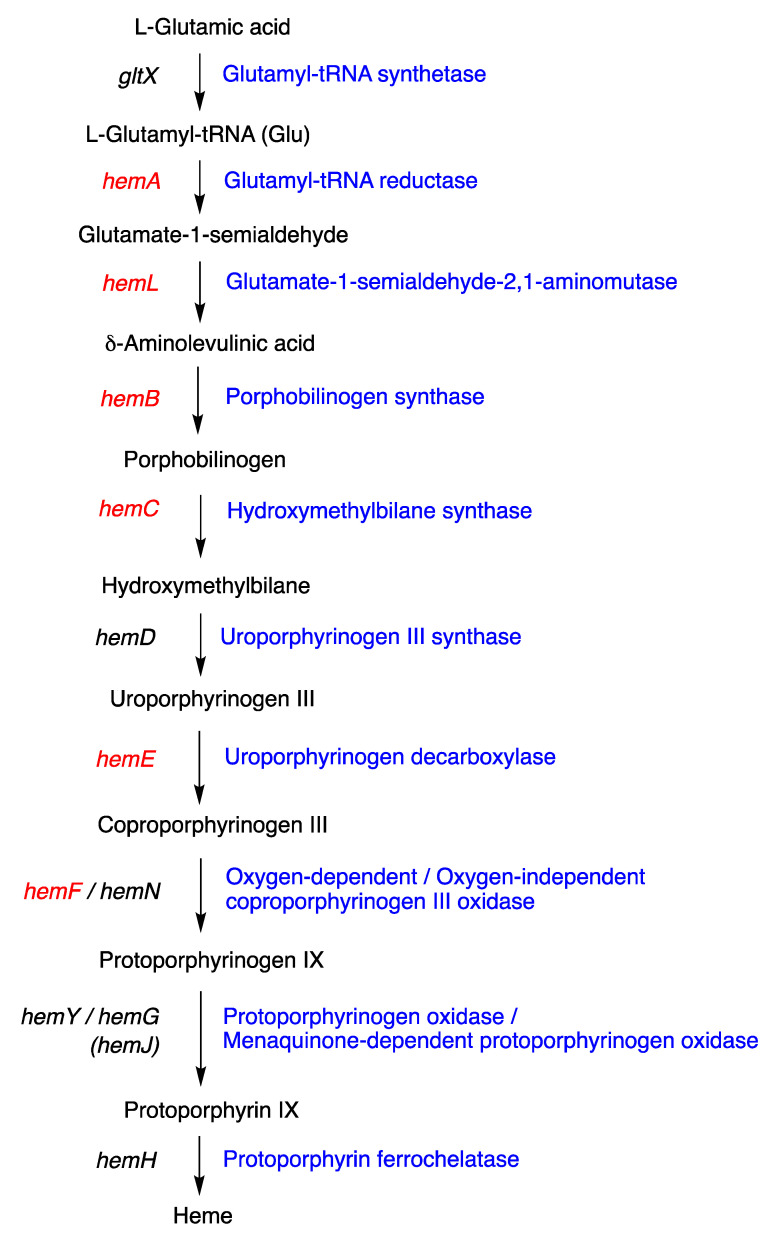
Tetrapyrrole biosynthesis pathway beginning with L-glutamic acid [58]. The ‘*hemX*’ nomenclature describes genes encoding enzymes, whereas the encoded enzymes are displayed in blue. The *hem* genes that are present in BGC-1 are displayed in red [164].

The core biosynthetic pathway for heme, as occurs in prokaryotes, is shown in Figure 49 [58]. The pathway begins with L-glutamic acid (C5 pathway) and proceeds through δ-aminolevulinic acid, porphobilinogen, and uroporphyrinogen III. Uroporphyrinogen III is known as the last universal precursor to all native tetrapyrrole macrocycles [165,166].

The dispersal of tetrapyrrole biosynthesis genes throughout the genome provided no obvious toehold for searching for genes for tolyporphins. Studious inspection of the genome, however, also revealed a cluster of additional genes encoding six of the required eight enzymes for conversion of glutamic acid to protoporphyrinogen IX [79]. The genes in the cluster (2,586,793–2,609,884 bp) include *hemA*, *hemL*, *hemB*, *hemC*, *hemE* and two *hemF* genes (thus seven *hem* genes in total); the two missing genes are *gltX* and *hemD*, which encode glutamyl tRNA synthetase and uroporphyrinogen III synthase, respectively. Eleven other genes thought to code for enzymes engaged in the biosynthesis of tolyporphins were termed “*tol*” genes. Provisional assignments were made for the selected *tol* genes (encoded protein). The *tol* genes and assignments are listed in Table 10.

The presence of the uncommonly clustered tetrapyrrole biosynthesis genes and additional genes suggested a role as a putative gene cluster for tolyporphins biosynthesis. The gene cluster was first recognized in the report of the genome [79], initially was termed BGC-T [88], and later was renamed as BGC-1 [164] upon identification in the HT-58-2 cyanobacterial genome of a second gene cluster (BGC-2, see Section 13) possibly also responsible for tolyporphins biosynthesis [164].

**Figure 50:**
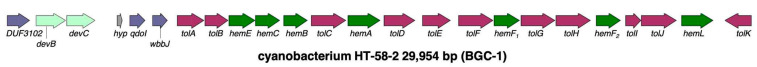
The putative tolyporphins biosynthetic gene cluster BGC-1 in the HT-58-2 cyanobacterial genome. Core tetrapyrrole biosynthetic genes (*hem*) are shown in green, *tol* genes in maroon, adjacent transport genes in light green, other identified genes in dark blue, and genes of unknown function in gray [164].

The arrangement of BGC-1 is shown in Figure 50. Beyond the presence of 7 *hem* and 11 *tol* genes, a further feature in BGC-1 is the presence of genes for secretory and transport proteins (leftmost region). Studies are underway to identify the function of specific genes in BGC-1 (see Section 16). The length of BGC-1 was initially regarded as spanning bp 2,586,793–2,609,884, yielding 23,091 bp [79]. On further analysis, the region of BGC-1 was expanded to include six additional genes, thereby spanning 2,579,931–2,609,884 (29,954 bp). One rationale for the expansion was to include the added genes (*dev* genes) aligned with those from other cyanobacteria (see Section 13).

Much more information is available concerning the genome of the HT-58-2 cyanobacterium, which contains 6177 protein-encoding genes [79]. The published report includes information concerning 219 essential genes; genes for nitrogen fixation; genes for chlorophyll and phycocyanobilin biosynthesis; genes for cyanophycin biosynthesis and degradation; genes for heterocyst formation; and genes associated with the biosynthesis of the orange carotenoid protein [79]. The next section turns to focus on other natural products.

## 12. HT-58-2 Genome—Genes for Other Natural Products

**Figure 51:**
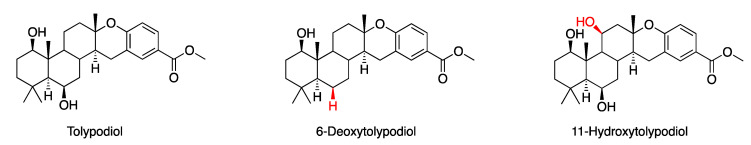
Structure of tolypodiol and two analogues isolated from HT-58-2 [88].

In 1996, Prinsep and coworkers reported the isolation of “tolypodiol”, a meroterpenoid, from the HT-58-2 culture [51]. In 2020, Gurr et al. reported the isolation of two additional members of the meroterpenoid family [112]. The structures of the three meroterpenoids are shown in Figure 51. The three compounds display varying degrees of anti-inflammatory behavior [51,112].

**Figure 52:**
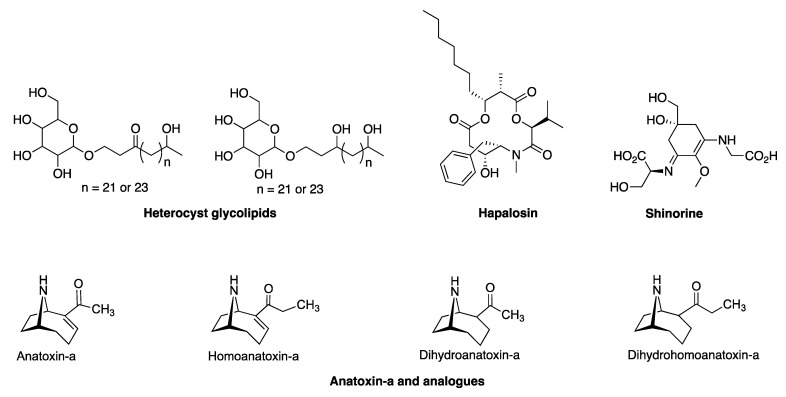
Expected natural products derived from putative BGCs (>50% similarity with reported ones) in HT-58-2 [88].

The discovery of previously unknown natural products from a cyanobacterial culture is hardly novel; examining cyanobacteria (and other marine bacteria) has proved so scientifically rewarding as to have been referred to [35] as “panning for chemical gold”. As Dittmann and coworkers stated (in 2015) [167] “The known chemical diversity of cyanobacterial natural products includes over 1100 secondary metabolites with intricate and exotic chemical structures reported from 39 genera of cyanobacteria”. Note that the terms natural products and secondary metabolites are used interchangeably. The prior annotation of the HT-58-2 cyanobacterial genome, and the discovery of the meroterpenoids, prompted examination of the genome for biosynthetic gene clusters (BGCs) for other natural products [88]. Use of standard bioinformatics tools (including anti-SMASH [168]) led to identification of 18 gene clusters, of which four contained >4 core genes with high similarity to known BGCs (Table 11). The natural products predicted from the corresponding BGCs are shown in Figure 52 and include heterocyst glycolipids, hapalosin, anatoxin-a, and shinorine.

The properties of each are as follows.

- Heterocyst glycolipids (HGs) are known to form a protective layer for oxygen-sensitive nitrogenase enzymes [169] in the envelope of heterocystous nitrogen-fixing cyanobacteria.
- Hapalosin is a cyclodepsipeptide that has been reported to reverse multidrug resistance in tumor cell lines [170].
- Anatoxin-a, also known as Very Fast Death Factor (VFDF), has high acute oral toxicity [171], and also causes tremors, paralysis, and death within minutes upon intraperitoneal injection in mice [172].
- Shinorine is one example of a mycosporine-like amino acid that functions as an ultraviolet sunscreen in cyanobacteria [173].

**Figure 53:**
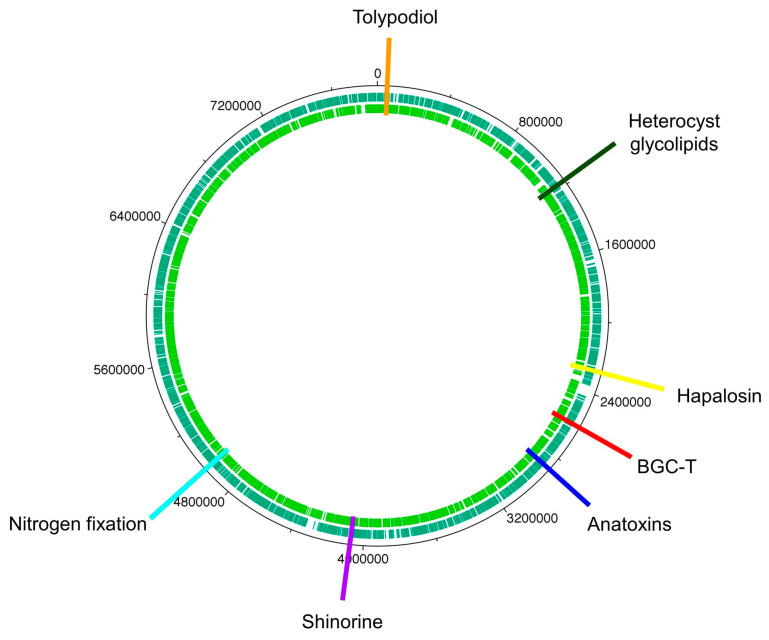
Annotation of the HT-58-2 cyanobacterial genome for putative BGCs [88].

The annotation of the HT-58-2 genome for the aforementioned BGCs is shown in Figure 53. In addition, a biosynthetic gene cluster (BGC) was identified that contains six of the eight genes for the non-mevalonate pathway to the diterpene synthon geranylgeranyl diphosphate, with the other two genes found elsewhere in the genome [88]. In addition to the six genes in the cluster is a handful of genes likely responsible for the terpene–quinonoid compounds, as required to elaborate the arene moiety of the tolypodiols. The location of the resulting assigned tolypodiol BGC (containing 15 genes), at 38,701–55,799 bp of the HT-58-2 genome, is shown in Figure 53. Very recently, the Williams group has reported heterologous expression in *Anabaena* sp. PCC 7120 of tolypodiol using some of the genes in the tolypodiol BGC region amplified from HT-58-2 [174].

Genes for nitrogen fixation also were identified (Figure 53) and found to be concentrated in two regions: 4,862,885–4,900,602 bp (*nif* cluster 1) and 4,972,977–4,986,558 bp (*nif* cluster 2). Five of the six genes (missing *nifB*) were in the former cluster, whereas three genes were in the latter cluster; together, all six required genes are represented among the two clusters. Genes for Fe-, Mo-, and V-containing metalloproteins are included in the clusters [79]. Genes for molybdate-dependent transporters and nitrogenases are co-localized. In this regard, the role of metal ion concentrations and environmental stimuli on gene expression for nitrogen fixation in HT-58-2 may warrant investigation. Cyanobacteria generally can assimilate nitrogen from a variety of sources, which is achieved by a finely tuned regular network of genes and gene products [116]. While not coding for a natural product per se*,* the ability to fix nitrogen was expected on the basis of general cyanobacterial behavior, the presence of nodules (the given name of the HT-58-2 cyanobacterium is *T. nodosa*), and growth in BG-11_0_ media. The interested reader is referred to the report for in-depth consideration of the putative BGCs and others with lesser extent of homology [88].

While only tolypodiols have to date been identified (and isolated) from the HT-58-2 cultures, the likely presence of other natural products, such as heterocyst glycolipids, hapalosin, anatoxin-a, and shinorine, indicates that bioassays of crude extracts should be interpreted with caution. Much has been written about BGCs in cyanobacteria. Dittman and coworkers pointed out that, on one hand [167], “The secondary metabolite gene clusters are widely held to be an ancient part of the cyanobacterial genome”. While on the other [167], despite BGCs as established entities, “…the genetic diversity underpinning natural product biosynthesis far exceeds the known chemical diversity, and that just a fraction of the pathways identified could be linked to known end products. Conversely, there remain many natural products for which a biosynthetic origin is unknown [175]”.

## 13. Searching for New Producers of Tolyporphins—Informed by Bioinformatics

At present, the HT-58-2 cyanobacterium–microbial consortium is the only known producer of tolyporphins. Identification of additional cyanobacteria that produce tolyporphins—identical with, similar to, or structurally distinct from known tolyporphins—would perhaps enable a deeper understanding of biosynthesis, evolutionary origin, and perhaps endogenous physiological function. The presence of clustered *hem* genes in the HT-58-2 cyanobacterial genome led to the identification of BGC-1. BGC-1 contains six *hem* genes, one in two copies (*hemABCEF_1_F_2_*); 11 putative *tol* genes; and genes for secretory and transport proteins. The cluster of genes suggested a significant marker for recognizing presumptive BGCs in other possible producers of tolyporphins.

**Figure 54:**
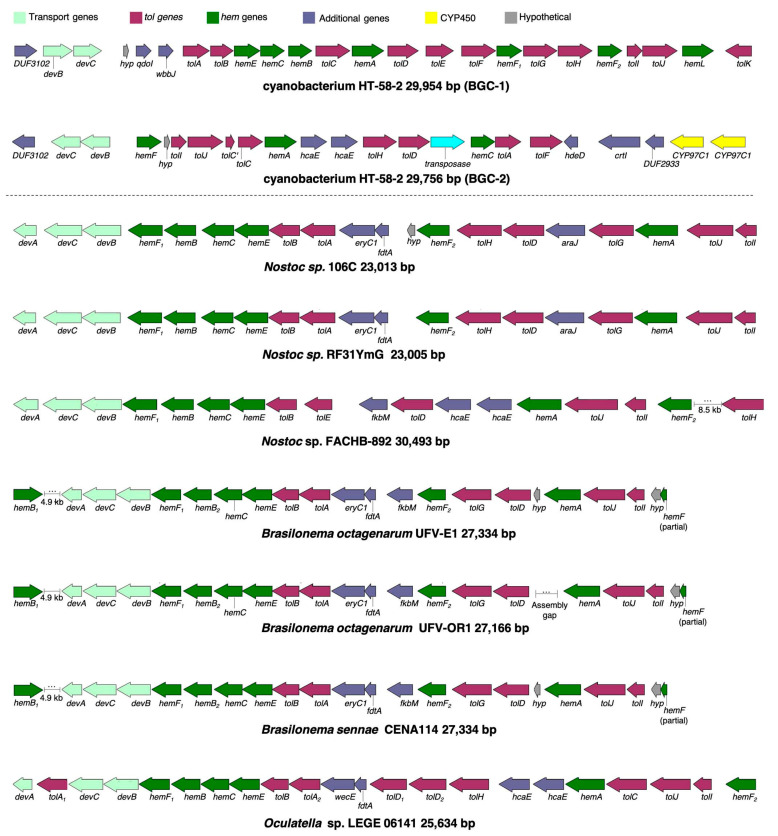
Putative BGCs identified in filamentous cyanobacteria. BGC-1 from HT-58-2 is compared to BGC-2 from HT-58-2 and seven newly detected clusters (each from distinct cyanobacteria). Core tetrapyrrole biosynthetic genes (*hem*) are displayed in green, *tol* genes in maroon, adjacent transport genes in light green, cytochrome P450s in yellow, other identified genes in dark blue, and genes of unknown function in gray [164].

An automated bioinformatics search to identify clusters of homologous sequences in BLAST searches (such as cblaster [176] or CAGECAT, comparative gene cluster analysis toolbox [177]) was not available to the authors’ knowledge at the time that BGC-1 was used as a search template. A manual search using BLASTP (basic local alignment search tool for proteins) was carried out using sequences for individual putative *tol* proteins. The expectation was to identify new organisms that might be producers of tolyporphins, given the well-known process of horizontal gene transfer of BGCs [178]. In so doing, remarkably, a second partial BGC was identified (termed BGC-2) in the HT-58-2 cyanobacterial genome itself (Figure 54). The BGC-2 was listed as spanning bp 2,994,941–3,043,548 [164], which was a slight clerical error; the actual region is 3,001,573–3,031,328 bp, yielding a 29.7 kbp cluster (Figure 54).

The similarities and differences of BGC-2 versus BGC-1 are as follows:

- BGC-2 contains coding sequences pertaining to seven putative proteins that correspond with >50% identity to Tol proteins from BGC-1 (TolACDHIJ), and all are arranged in the same orientation.
- There is only one cytochrome P450 in BGC-2, which shares higher identity to TolH (CYP88A) than TolG, versus two in BGC-1. However, two other P450 genes (yellow arrows in Figure 54) are adjacent to BGC-2.
- Duplicate *hcaE* genes encoding aromatic ring-hydroxylating dioxygenases [179] are present within BGC-2, but not within BGC-1. The relevance of such genes is unknown.
- Three transport-related protein genes (DUF3102 domain-containing proteins DevB and DevC) are present at the leftmost end of BGC-2, similar to that of BGC-1.

Studies to identify the roles of BGC-1 and BGC-2 and genes therein in the biosynthesis of tolyporphins are described in Section 16.

Using BGC-1 of HT-58-2 as the query template led to identification of clusters in seven cyanobacteria other than HT-58-2. The organisms, along with their location and environmental origin, are listed in Table 12. The number of *hem* and *tol* genes is also listed. The alignment of the seven BGCs is displayed in Figure 54 for comparison with BGC-1 and BGC-2 of HT-58-2.

The seven organisms were isolated from environmental sites as diverse as their geographical locations in Brazil, China, Mexico, and Portugal. The environments include coralloid roots, soil crusts, surface of leaves, surface of a pipe, and the intertidal zone of a luxurious sandy beach. The existence of cyanobacteria as symbionts within coralloid roots of cycads (ancient gymnosperms) is a testament to biological adaptability [180]. While the filamentous or unicellular nature of cyanobacteria is often not stated, at least the *Nostoc* strains were explicitly indicated to be non-axenic. The similarities among the predicted BGCs include the following:

- Each BGC contains the unusual clustering of multiple *hem* genes, including *hemABCEF*, that are adjacent to several *tol*-like genes found in BGC-1 of HT-58-2.
- None of the BGCs includes *hemD* (UroS).
- Each *Nostoc* spp. and *Brasilonema* spp. contains two *hemF* genes (the aerobic coproporphyrinogen decarboxylase).
- Each BGC contains *tolB* (the RfbA orthologue, glucose-1-phosphate thymidylyltransferase) and *tolD* (the glycosyltransferase).
- Each BGC has nearby genes for secretory and transport proteins (DevB and DevC families).

**Figure 55:**
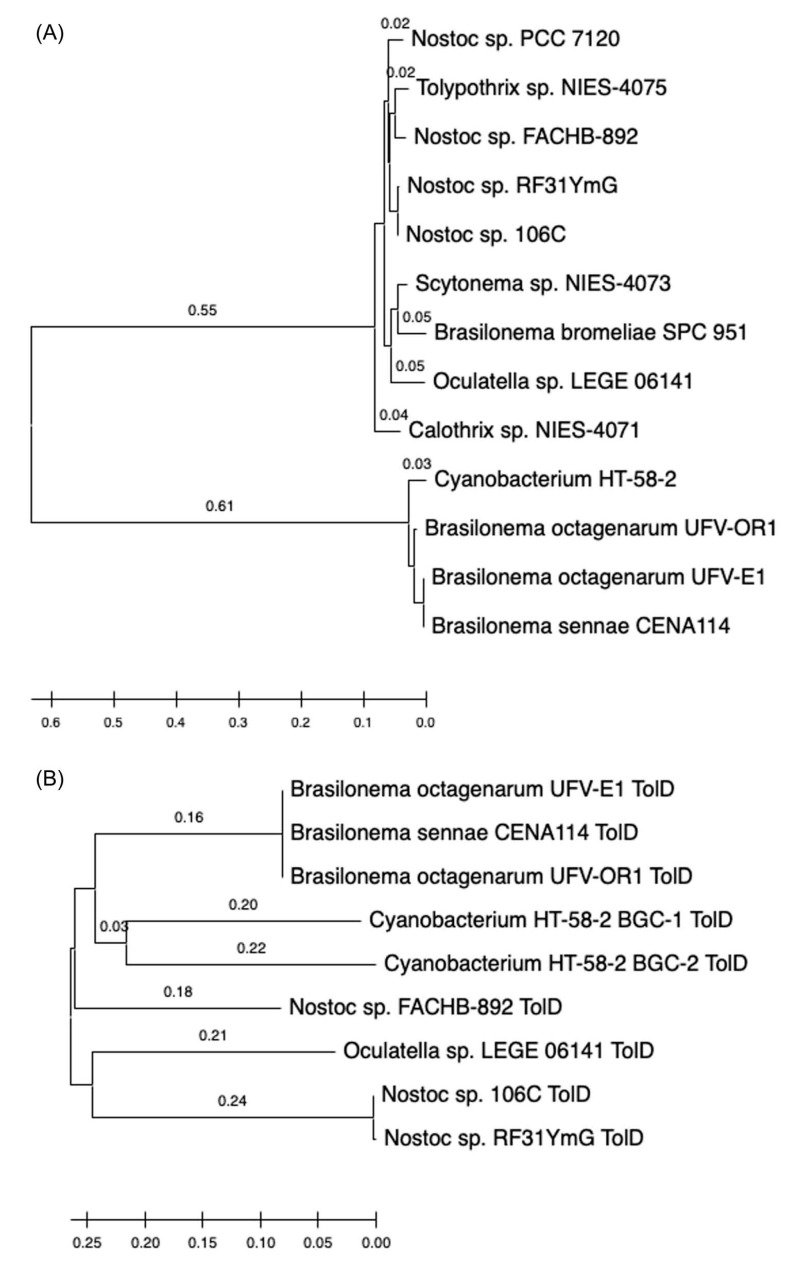
Phylogenetic trees of cyanobacteria containing putative BGCs for tolyporphins. (**A**) Phylogenetic tree on the basis of aligned 16S rRNA sequences of 1027 bases; sum of branch length = 1.49. (**B**) Phylogenetic tree on the basis of aligned TolD proteins of 420 amino acids; sum of branch length = 1.28. The trees are drawn to scale with branch unit lengths (above the line, or hidden if shorter than 0.02) the same as those used to infer the phylogenetic tree. Italics font for denoting organisms was not accessible when the figure was composed [164].

The phylogenetic tree for the eight strains constructed on the basis of 16S rRNA (1027 bp) is shown in Figure 55, panel A. The HT-58-2 cyanobacterial strain clearly shows a closer relatedness to the three *Brasilonema* strains than to other filamentous cyanobacteria [79,164]. A phylogenetic tree also was generated for the protein sequence of TolD, for which the tolD gene is present in each of the eight BGCs. The TolD phylogenetic tree shows a similar branching pattern as for 16S rRNA (Figure 55, panel B). The results for the two trees suggest that (1) the BGCs have the same evolutionary history as the cyanobacteria, and (2) the BGCs were not separately acquired via recent lateral gene transfer [164].

Three of the cyanobacterial samples listed in Table 12 (*Nostoc* sp. 106C, *B. octagenarum* UFV-E1, *Oculatella* sp. LEGE 06141, and HT-58-2) were each procured and grown in BG-11 or BG-11_0_. The other four cyanobacterial samples listed in Table 12 could not be obtained. Three methods of detection for tolyporphins shown in Figure 30—absorption spectroscopy (Process 4*), HPLC with absorption spectroscopic detection (Process 5*), and the fluorescence assay (Process 8**)—each yielded a positive signal for the extract from HT-58-2. On the other hand, extracts from the three procured cyanobacterial samples did not yield a positive signal for tolyporphins from any of the three assays [164].

The absence of a signal for tolyporphins has several possible interpretations: (1) The BGCs (including in HT-58-2) shown in Figure 54 are not responsible for the production of tolyporphins. (2) The BGCs are essential, but alone not sufficient, for tolyporphins production; other gene products also are required. (3) The identified BGCs suffice for tolyporphins production; however, for the newly procured cyanobacteria: (i) the appropriate stimulus to elicit tolyporphins production has not yet been identified; (ii) tolyporphins are produced, but at a level below the limits of detection in the assays; or (iii) the composition of tolyporphins (e.g., oxochlorin or porphyrin type) escapes detection by the analytical methods examined [164].

The story presented here appears very incomplete. The complexity of the HT-58-2 culture, composed of a filamentous cyanobacterium and community bacteria, may play a role in production of tolyporphins. The yield of tolyporphins is strongly influenced by the presence/absence of a soluble source of nitrogen. The three procured cyanobacteria examined for tolyporphins production are non-axenic. In this regard, filamentous cyanobacteria living in complex environments might share nutrients or products with other members [182]. Thus, it cannot be ruled out that one or more precursors or intermediates in the biosynthesis of tolyporphins may be contributed by other members of the community [164].

## 14. Searching for New Producers of Tolyporphins—Classical Approach

Cyanobacteria are among the most abundant and widespread organisms on Earth [183] The number of cyanobacterial *species* described has been estimated to be 2000–5000 [184,185], whereas the total number extant has been estimated to be 8000 [184], although this number is uncertain if not controversial [185]. The number of cyanobacterial *specimens* (i.e., samples) available is said to be 8000, given collections such as that of the Natural History Museum in London [186], as well as repositories such as the Pasteur Culture Collection of Cyanobacteria [187].

Prior to finding that the HT-58-2 culture was not in fact lost, had been resuscitated, and would be made available to us (October, 2015), we launched two searches for cyanobacteria that might produce tolyporphins. The first search initially was focused on procuring strains of the *Tolypothrix* genus or similar strains on the basis of phylogeny. Once the genomic sequence of the HT-58-2 cyanobacterium was obtained, where phylogenetic analysis pointed not to *Tolypothrix*, but instead to *Brasilonema* [79], strains were sought of that genus or that were believed to be similar thereto. Strains were obtained from commercial collections and from individual scientists. Ultimately, 62 strains were obtained. Each strain was cultured in our labs and then extracted; the lipophilic extracts were examined by absorption spectroscopy, ESI-MS, MALDI-MS, and IR-MALDESI-FTMS processes, shown in Figure 30. In no case were tolyporphins identified. The sources and studies are provided in the Supplementary Materials (Section S4, Tables S2 and S3). This work was completed prior to the development of the more sensitive fluorescence assay.

**Figure 56:**
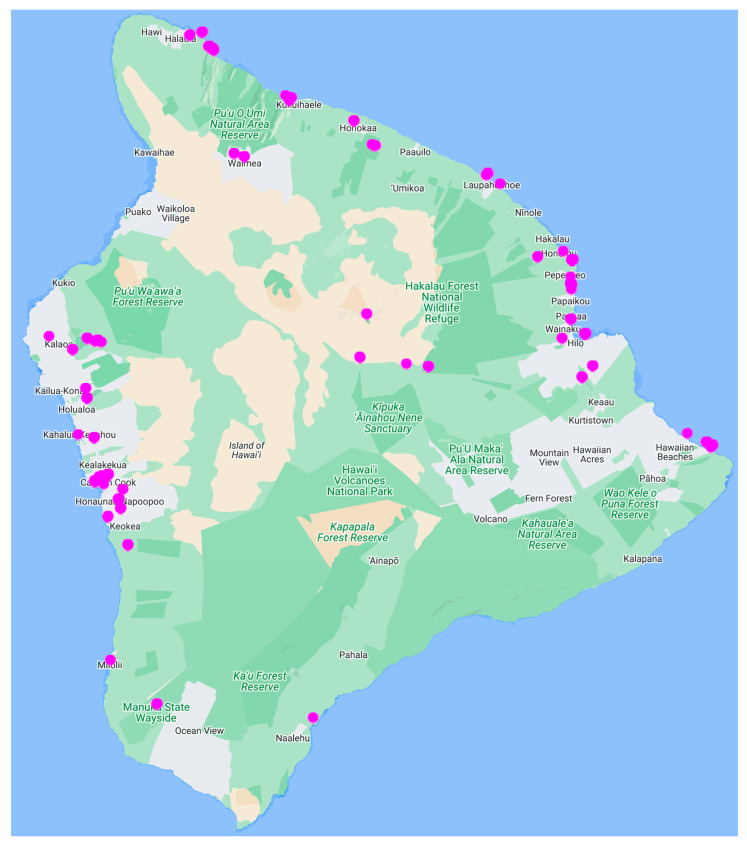
Collection sites in Hawaiʻi. GPS coordinates for each collection site were entered into a mapping program (maptive.com) to display the diagram. Each magenta circle indicates the site of collection of 1 to as many as 20 samples.

The initial phase of the first search proved frustrating in terms of obtaining strains for examination, although over the course of several years, a collection of modest size was obtained. Hence, a second, decidedly more Ahabian search, aimed to create an independent collection of cyanobacteria. The search focused exclusively on the island of Hawaiʻi (Figure 56), the largest in the Hawaiian chain.

The rationale for Hawaiʻi was not simply “magic allure” [188], but more the striking plant life [189,190], the “within island abiotic heterogeneity” [191], and the diversity of climactic zones including a tropical zone in a compact geographical region (10,430 km^2^), which is smaller than the U.S. state of Connecticut (12,559 km^2^) and larger than the French island of Corsica (8722 km^2^). Hawaiʻi contains four of the five climate groups identified by Köppen-Geiger [192], and ten of fourteen subgroups. The major climate groups include (1) humid and tropical; (2) arid and semi-arid; (3) temperate; (4) cold, continental; and (5) ice or alpine. Hawaiʻi contains all but the cold, continental climate. It has been pointed out that [193,194] “The Hawaiian Islands have one of the most diverse rainfall patterns on earth”. The mean annual rainfall in Hawaiʻi ranges from 19 cm near Kawaihae to as much as 676 cm some 90 km away in Honomū [192], for example, giving closely spaced isohyets over much of the island [193]. But, rainfall is not the whole story, because (1) the lapse rate (temperature decrease with altitude) is 0.65 °C/100 m below 1250 m [195], and (2) “ascending orographic clouds compressed between the rising mountain slope and an upper-air temperature inversion produce frequent ground level mountain fog, an important moisture source for upland vegetation” [192,196]; hence, terrestrial regions of little rainfall can still afford a moist environmental milieu rich for cyanobacterial growth.

Samples from a wide variety of terrestrial locales, chiefly in the humid/tropical or temperate regions (Figure 56), were collected by the communicating author and returned to his lab in Raleigh, North Carolina, for culturing. Of the 202 samples collected, 139 yielded green, putative cyanobacterial cultures, and 101 of those were grown to sufficient quantity for testing. Each was extracted for analysis by the fluorescence assay (Process 8**, Figure 30) at that time or was examined subsequently. Again, in no case was a signal for tolyporphins observed. The procedures and results are provided in the Supplementary Materials (Section S5, Table S4, Figures S5–S7). A more full account of the field study including the individual sites of collection of the 202 samples may be published elsewhere.

Both types of searches have limitations. The phylogenetic-based search focuses on organisms defined by sequencing to be in the same clade as the sole known producer, yet such strains obtained from the culture collections of others may lack essential community bacteria from their native environment, and often have limited information about their culture and storage history, which over time can cause alteration of innate biosynthesis capacity. Moreover, gene clusters responsible for natural products formation often can be exchanged between microorganisms; hence, phylogenetic similarity does not necessarily imply a similar natural products portfolio. The Hawaiian field search was completely agnostic with regards to sequence information and affords strains directly from their native environment. While neither search elicited a positive response for production of tolyporphins, in both cases, the issue of whether the search has satisfactorily covered adequate space, either phylogenetically or geographically, is not clear. In the Hawaiian survey, each sample was collected from a ~3 cm × 3 cm surface scraping, which is practically infinitesimal (~10^−13^ fold) versus the 10^4^ km^2^ surface area (and still a minute fraction of the verdant region) of the island of Hawaiʻi. A second issue concerns whether a cyanobacterial culture that has the capacity to produce tolyporphins is suitably cultured to elicit production of tolyporphins. Samples from both searches were grown in one of several media: BG-11, BG-11_0_, and/or AA media. As pointed out by Castenholz [77], “no medium is perfect for all cyanobacteria, and individual tailoring or modifications are needed depending on the purpose intended. For example, if the need is to obtain the greatest yield of cyanobacterial cells, perhaps in the shortest time, a rich medium may be required. If the purpose is to elicit morphological, cytological, and physiological features that might be characteristic of natural populations, low nutrient medium is needed. A deficiency of combined nitrogen is generally required to elicit heterocyst differentiation and the synthesis of nitrogenase”. Many BGCs remain silent during laboratory culture, and indeed, the stimulus required to elicit production of the corresponding natural products is often unknown [197].

On reflection, should a more sensitive and higher-throughput assay for tolyporphins be developed, the survey for tolyporphins should be repeated of many of the *Brasilonema* (or related) strains sourced commercially and from individuals, as well as perhaps the samples collected in Hawaiʻi. In the meantime, focusing on those strains identified by bioinformatics comparisons on the basis of putative BGCs appears a more prudent avenue for exploration. Whether enlightened, appropriate, or ill-fated, the goal remains to find additional producers of tolyporphins beyond that of the present sole source, HT-58-2.

## 15. Biosynthesis Considerations

The diverse structures of members of the tolyporphins family (Figure 6) might at first appear perplexing. Analysis, however, reveals common structural features [66]. First and foremost is the pattern of substituents about the perimeter of the macrocycle. As thoroughly delineated in Section 4.2, the pattern of peripheral methyl groups in circumnavigating all tolyporphins is MeX-MeX-MeX-XMe (tracking positions 2,3–7,8–12,13–17,18), where X is a variable substituent. The pattern coheres with that of all other native tetrapyrrole macrocycles. This point is illustrated for tolyporphin A and uroporphyrinogen III in Figure 12 and companion text. As all native tetrapyrroles share uroporphyrinogen III as a common precursor, it was a strong hypothesis that tolyporphins inevitably must also derive from uroporphyrinogen III [66].

Further structural features across the repertoire of tolyporphins that are germane to biosynthesis considerations are as follows: (1) the absence of substituents at four β-pyrrolic positions; (2) the appended *C*-glycosides in oxochlorin-type and dioxobacteriochlorin-type macrocycles (i.e., all members except the porphyrin tolyporphin P); and (3) the molecular diversity in the appended glycosides (including the somewhat rare glycosides) and other units.

**Figure 57:**
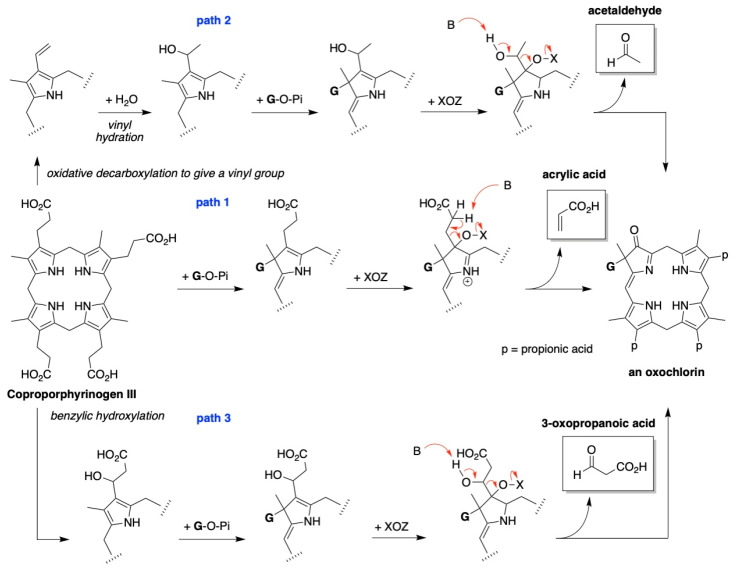
Distinct mechanisms proposed for dealkylation and *C*-glycosylation of coproporphyrinogen III. Adapted with permission from ref [66]; 2021, The Royal Society of Chemistry.

To the tetrapyrrole aficionado, the most intriguing, if not the strangest, of these three structural features, without doubt, is not the *C*-glycosides and their molecular diversity, but rather the presence of open β-pyrrolic positions [66]. Uroporphyrinogen III is well known to undergo enzymatic processing of peripheral groups, including decarboxylation of the four acetic acid units to yield the tetramethyl counterpart, coproporphyrinogen III. The structure of coproporphyrinogen III is shown in Figure 57. Two of the propionic acid groups undergo decarboxylation and dehydrogenation to yield the two vinyl groups found in protoporphyrin IX and many other downstream tetrapyrrole macrocycles. But, loss of the four propionic acid groups altogether, as required to generate tolyporphins from uroporphyrinogen III, presented a mystery. (Here, we note the longstanding and more popular usage of propionic acid in the context of tetrapyrrole biosynthesis, versus the equivalent and more formal IUPAC term, propanoic acid.) Attempts to probe the biosynthesis in the HT-58-2 cyanobacterium by use of isotopically labeled substrates were not successful (Supplementary Materials, Section S6, Figures S8–S11, Table S5).

Three distinct routes for removal of propionic acid groups from coproporphyrinogen III (or analogue) were proposed on the basis of enzymatic precedent and chemical intuition [66]. The enzymatic precedent resides in the biosynthesis of heme *d*_1_, where two propionic acid groups are removed via an enzyme containing the *S*-adenosylmethionine (SAM) cofactor. The structures of heme *d*_1_ (lacking two propionic acid groups) and its biosynthetic precursor, siroheme (containing two propionic acid groups), are shown in Figure 12. Three proposals were described and are shown in Figure 57. In path 1, alkylation with a glycosylation agent (**G**-O-Pi) leads to elimination of acrylic acid to yield the gem-dialkyl-substituted oxochlorin. In path 2, hydration followed by similar alkylation leads to elimination of acetaldehyde. In path 3, benzylic hydroxylation followed by similar alkylation leads to elimination of 3-oxopropanoic acid. In each case, the pyrroline ring forms as part of the *C*-glycosylation process [66]. A second run along any of these paths would afford the dioxobacteriochlorin [66].

**Figure 58:**
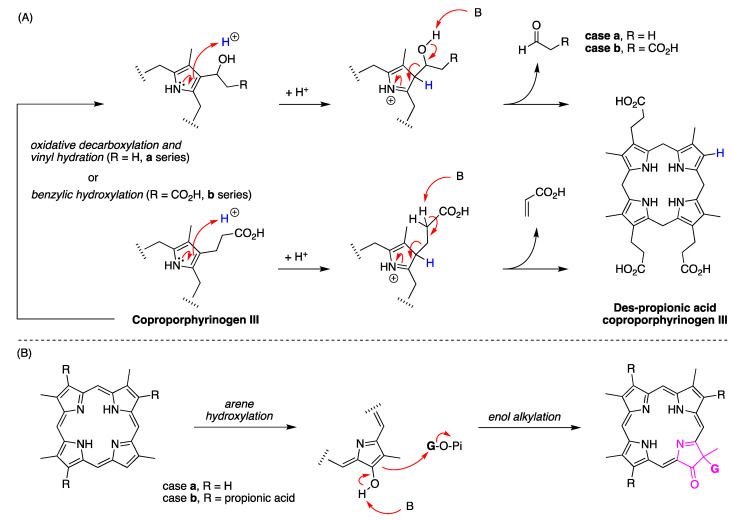
(**A**) Distinct mechanisms proposed for dealkylation of coproporphyrinogen III. (**B**) Arene hydroxylation, followed by enol alkylation. Adapted with permission from ref [66]; 2021, The Royal Society of Chemistry.

Analogous pathways were described for removal of propionic acid groups from coproporphyrinogen III without the initial alkylation step, as shown in Figure 58, panel A. Continuation of this process would afford the porphyrinogen counterpart of tolyporphin P. In each case, the pyrrole ring remains intact and there is no *C*-glycosylation process [66].

An open β-pyrrole position is highly susceptible to attack by electrophiles. Electrophilic attack can occur with a pyrrole embedded in a porphyrinogen, in a porphyrin, or in a species intermediate between the two bookends. In this context, it warrants mention that the presence of a single alkyl group substantially increases the reactivity of a pyrrole toward electrophiles [198,199]. Indeed, a pyrrole with three methyl groups versus pyrrole itself has reactivity increased by a factor of 10 million, “which corresponds to relative reaction times of 1 min to 20 years!” [198]. Thus, in a porphyrinogen, a β-methylpyrrole (which is a trialkylpyrrole) is expected to be profoundly more reactive toward electrophiles than pyrrole itself. A β-methylpyrrole embedded in a porphyrin (or other tetrapyrrole macrocycle) is also reactive given the stabilization of charged or radical intermediates by the macrocycle π-system. Oxidation to install a β-hydroxy group sets up facile enol-keto tautomerization; the latter can be accompanied by alkylation with a *C*-glycosylation agent to create the oxochlorin as shown Figure 58, panel B [66].

**Figure 59:**
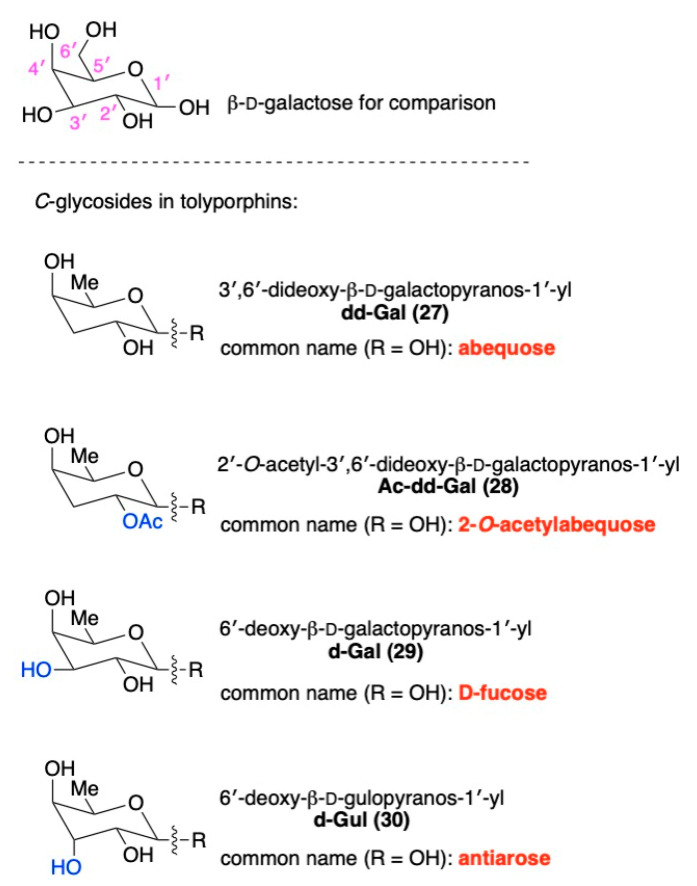
Glycoside diversity in tolyporphins. Reprinted with permission from ref [66]; 2021, The Royal Society of Chemistry.

The nature of the appended glycosides and the diversity across the repertoire of tolyporphins has been the subject of study. Formation of *C*-glycosides is well known in natural products chemistry [33], but apparently unprecedented in tetrapyrrole science outside of the family of tolyporphins. Each glycoside is a pyranose with D-configuration, contains a 4′-axial hydroxy group characteristic of galactose, and lacks a 6′-hydroxy group. Variation accrues at the 3′-position (including an equatorial, axial, or no OH) and the 2′-position (OH or *O*-acetyl). The structures and common names of the glycosides are shown in Figure 59 and include abequose, 2′-*O*-acetylabequose, D-fucose, and antiarose. Note that antiarose is also known as 6′-deoxygulose, where gulose is a 3′-epimer of galactose. The structure of β-D-galactose is provided for comparison. Such glycosyl units are found elsewhere in natural products, although some are quite rare among bacteria; the interested reader is referred to ref [66] for in-depth information.

The various substituents in the pyrroline motifs of tolyporphins are shown in the structures (Figure 6) and also are tabulated here for more concise consideration (Table 13). Possible biosynthetic processes for diversification of the pyrroline substituents (*C*-glycoside, hydroxy, acetoxy) have been described in some depth [66] and will not be repeated here. Key distinctions are whether there pre-exists a full repertoire of glycosyl units each of which is processed individually in each biosynthesis of a tolyporphin, or whether there is enzymatic processing of one or more generic glycosyl motifs following attachment to the tetrapyrrole macrocycle. The enzymatic processing could entail hydroxylation, reductive dehydroxylation, *O*-acetylation, *O*-deacetylation, and epimerization. The late-stage diversification of intact glycosides by introduction of *O*-acetyl groups is a known biological strategy [200].

The interrelationships of various tolyporphins have been described as follows [66]: “Tolyporphins interrelated by *O*-acetylation of a *C*-glycosyl unit (abequose) include the following: D → B,C → A. Tolyporphins interrelated by *O*-acetylation of a hydroxyl group include the following: F → E; J → G,H → I; and Q → R. (Such relationships assume congruent stereochemistry of the corresponding groups, which has not yet been established.) Thus, if tolyporphins A, E, I, and R are regarded as fully *O*-acetylated, then eight additional tolyporphins with incomplete or no *O*-acetylation reside in the same respective structural lineage. Tolyporphins L and M are isomers and contain one 2′-*O*-acetylabequose and one antiarose, whereas tolyporphins N and O are isomers and contain one 2′-*O*-acetylabequose and one D-fucose. As abequose is the only sugar in the tolyporphins family that is present as an *O*-acetyl entity, tolyporphins L–O are regarded as fully *O*-acetylated. In other words, biosynthesis of four core tolyporphins accompanied by incomplete *O*-acetylation affords 2/3 of the tolyporphins: set A–D; set E,F; set G–I; and set Q,R. The remaining 1/3 of the tolyporphins are K, P, isomers L,M, and isomers N,O”. The upshot is that the rich diversity of the tolyporphins A–R family may be produced by a limited set of enzymes. Diversity in natural products [68] is known to be achieved by promiscuous enzymes, including *C*-glycosyltransferases [201]. Whether there is method to the madness of tolyporphins diversity, or the diversity simply reflects a broad and indiscriminate profusion of microbial defense products, remains unclear. The considerations in this section are theoretical and were largely posited on chemical intuition and literature precedent. Experimental studies of biosynthesis of tolyporphins are described in the next section.

## 16. Abe’s Studies of Tolyporphins Biosynthesis

**Figure 60:**
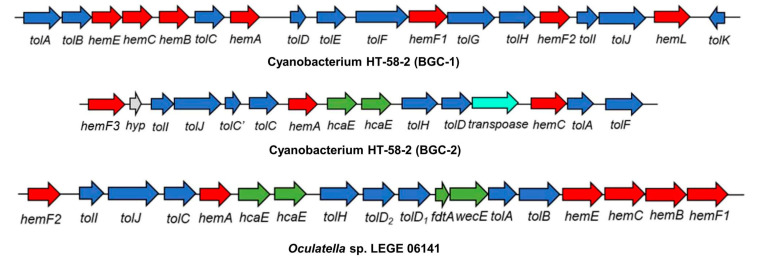
Alignment of putative BGCs for tolyporphins. Adapted with permission from ref [202]; 2023, American Chemical Society.

A very recent publication described the results from a bold research program aimed at recapitulating the biosynthesis of tolyporphins [202]. The work, reported by Ushimaru, Lyu, Ling, and Abe, relied on heterologous expression of *tol* and *hem* genes identified in the two BGCs of the HT-58-2 cyanobacterium, as well as that of *Oculatella* sp. LEGE 06,141 [164]. The location of *hem* genes in BGC-1 and BGC-2 of HT-58-2 are shown in Figure 48. The *tol* genes identified in BGC-1 are listed in Table 10. The three BGCs are shown in Figure 50 and Figure 54. The origin of *Oculatella* sp. LEGE 06141, identified by bioinformatics searching with BGC-1 as a query [164], is shown in Table 12. The three gene clusters are displayed in Figure 60 for direct comparison. The protein sequence similarities corresponding to the genes in BGC-1 and BGC-2, compared to those in *Oculatella* sp. LEGE 06141, are displayed in Table 14.

**Figure 61:**
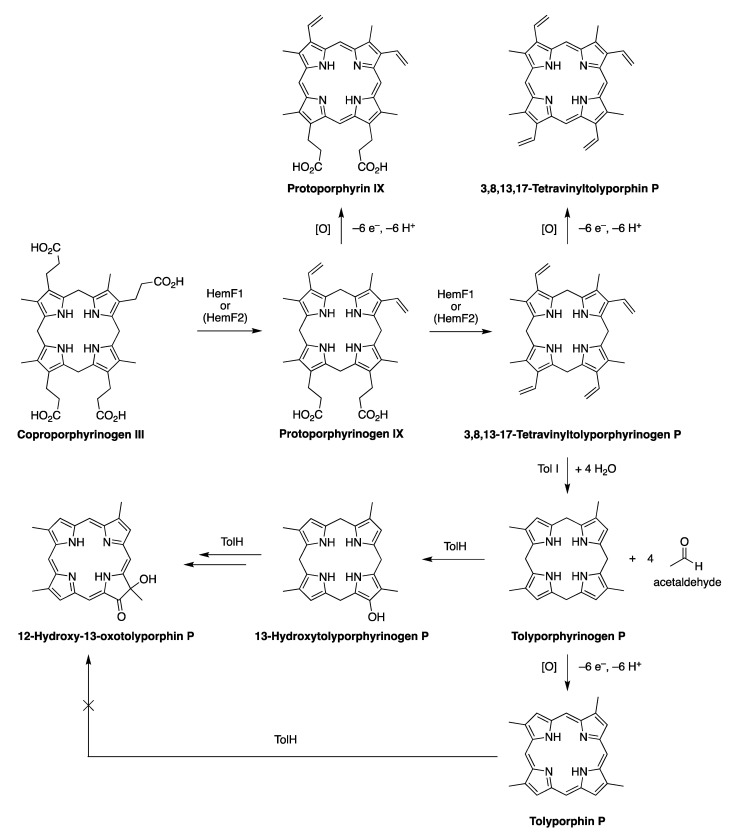
Emerging picture of a possible biosynthetic pathway to tolyporphins.

The Abe group carried out a brilliant series of experiments that together have afforded an emerging picture of the biosynthesis of the core scaffold of tolyporphins. The emerging picture is shown in Figure 61. The experiments are outlined in the following [202].

**Figure 62:**
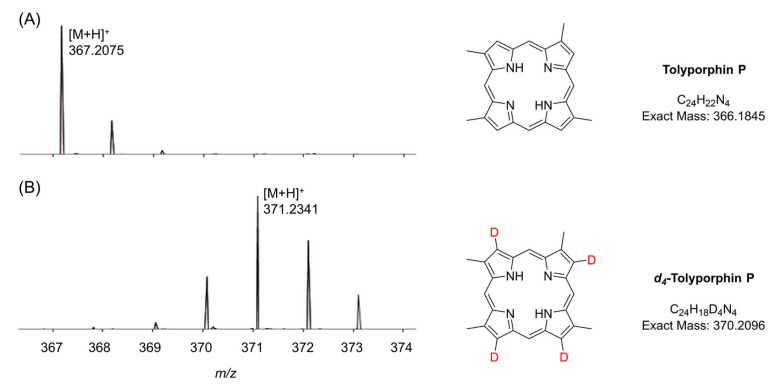
ESI-MS data. (Panel **A**): Tolyporphin P. (Panel **B**): The deuterated *d_4_-*tolyporphin P produced from the in vitro reaction of coproporphyrinogen III in D_2_O with enzymes OsHemF1, HtHemF2 and OsTolI-CΔ41 [202]. Adapted with permission from ref [202]; 2023, American Chemical Society.

**Figure 63:**
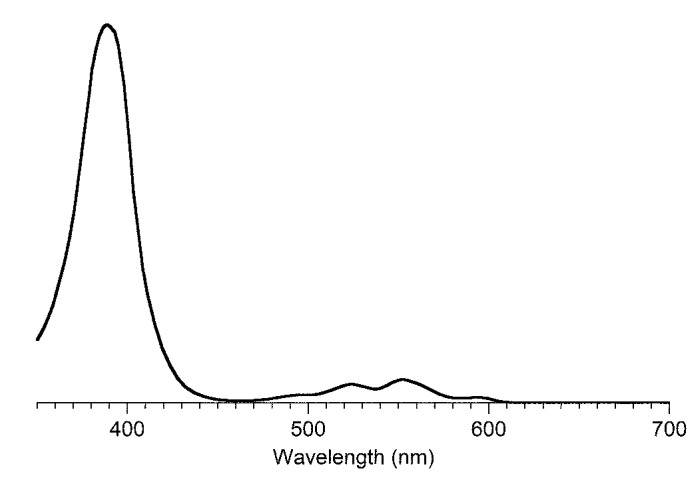
Absorption spectrum of tolyporphin P product derived by heterologous gene expression. Adapted with permission from ref [202]; 2023, American Chemical Society.

(1) A key insight by the Abe group was to focus on the duplicated HemF homologues of BGC-1. While HemF1 shows 79% similarity to the canonical HemF, the similarity of HemF2 is only 38%, for which the Abe groups hypothesized similar enzymatic function, but perhaps different substrate tolerance. Heterologous expression of *hemF1* (cloned from *Oculatella* sp. LEGE 06141) in *Escherichia coli* afforded a recombinant protein (termed OsHemF1) that upon incubation with coproporphyrinogen III, followed by oxidation (to convert porphyrinogens to porphyrins), yielded protoporphyrin IX (a normal outcome in heme biosynthesis). The oxidation is a dehydrogenation process that entails removal of 6e^–^ and 6H^+^ to form the aromatic macrocycle. The experiment confirmed the identify of OsHemF1 as a coproporphyrinogen oxidase, which converted two propionic acid groups to two vinyl groups.

(2) Heterologous expression of a codon-optimized *hemF2* (from HT-58-2) in *E. coli* upon treatment with δ-aminolevulinic acid resulted in formation of a tetravinyl-tetramethylporphyrin (here termed **3,8,13,17-tetravinyltolyporphin P**). Also, treatment of the proteins OsHemF1 and HtHemF2 (from the expressed *hemF1* and *hemF2*, respectively) with coproporphyrinogen III, followed by oxidation, yielded protoporphyrin IX. The results imply HtHemF2 is a non-canonical decarboxylase that can lead to 3,8,13,17-tetravinyltolyporphin P from protoporphyrinogen IX.

(3) The individual genes, *tolC* (acyl transferase), *tolG* (P450), *tolH* (P450), *tolI* (hypothetical protein), and *tolJ* (FAD-binding protein) were expressed in *E. coli* with the codon-optimized *HthemF2*. The genes were obtained by chemical synthesis and/or amplification of genomic DNA from *Oculatella* sp. LEGE 06141. The co-expression solely of *OstolI* resulted in formation of the porphyrin product tolyporphin P. The product (2.2 mg), which contained some inseparable impurities, was confirmed by ESI-MS and ^1^H NMR spectroscopy, as well as absorption spectroscopy. The ESI-MS spectrum is shown in Figure 62. The absorption spectrum is shown in Figure 63. The absorption spectrum is clearly that of a porphyrin, but differs slightly from that of tolyporphin P identified upon isolation from HT-58-2 as shown in Figure 28 (panel P).

(4) The protein OsTolI was posited to be membrane bound, prompting expression of an aqueous-soluble analogue (OsTolI-CΔ41) that lacked an amphipathic tail. The following experiments were carried out with revealing results: (a) Incubation of the proteins OsTolI-CΔ41, OsHemF1, and HtHemF2 with coproporphyrinogen III, followed by oxidation, yielded tolyporphin P. (b) Similar reaction in D_2_O yielded ***d_4_*-tolyporphin P** (69%), as well as the trideutero (29%) and dideutero (4%) products. The ESI-MS spectrum of *d_4_*-tolyporphin P is shown in Figure 62. (c) Treatment of the aqueous reaction mixture with 2,4-dinitrophenylhydrazine yielded the hydrazone derived from acetaldehyde. The results imply that TolI is a hydrolase that carries out the four carbon-carbon bond cleavages of 3,8,13,17-tetravinyltolyporphyrinogen P to form **tolyporphyrinogen P**, the hexahydro analogue of, and hence direct progenitor of, tolyporphin P. The incorporation of deuterium at each β-pyrrolic site speaks to the mechanism of the net dealkylation process. [A note on nomenclature—here, we retain the “toly” name, elide the irregular “porphin”, and append the standard “porphyrinogen” to denote members of the porphyrinogen family].

(5) The heterologous expression of *HthemF2* and *OstolI* in *E. coli* was carried out along with the genes *fdx* and *fdr* from *Synechococcus elongatus* to supply ferredoxin and ferredoxin reductase (Fdx-FdR) as a cytochrome P450 reducing system. The inclusion of *tolH* (but not *tolG*) led to ESI-MS and absorption spectra consistent with the α-hydroxyketoporphyrin **12-hydroxy-13-oxotolyporphin P**. On the other hand, exposure of tolyporphin P to *E. coli* cells expressing TolG/Fdx-FdR did not yield the 12-hydroxy-13-oxotolyporphin P. The results suggest that TolH, a cytochrome P450, “is involved in the oxidation of tolyporphyrinogen P”, and that tolyporphyrinogen P, not tolyporphin P, is an intermediate of tolyporphins biosynthesis [202].

In summary, the penetrating work of the Abe group has revealed features of key *tol* genes and now provides a compelling roadmap for understanding the biosynthesis of tolyporphins. Their central findings include the following: (1) the HemF1 and HemF2 coproporphyrinogen III decarboxylases act on the four propionic acids, not merely two propionic acids as in the canonical biosynthesis of protoporphyrin IX; (2) the TolI Fe-S protein catalyzes a yet little-understood reaction to remove the resulting vinyl groups; and (3) the TolH P450 hydroxylates the open β-pyrrole position of tolyporphyrinogen P.

An outcome of the pioneering work by Abe and coworkers is to pinpoint tolyporphyrinogen P as a precursor to tolyporphin P; moreover, they have suggested tolyporphyrinogen P as an intermediate on the path to oxochlorin-type and dioxobacteriochlorin-type tolyporphins. This insight, while at present a provocative hypothesis because the attachment of the *C*-glycosides of the latter tolyporphins has not yet been demonstrated, if found to be valid suggests a general pathway for late-stage derivatization to form the entire repertoire of tolyporphins. While not causing a paradigm shift, as previously there was no experimentally supported paradigm, the results have certainly altered the focus. The longstanding focus in tolyporphins research has naturally centered on the dioxobacteriochlorin-type tolyporphins, given the initial discovery of tolyporphin A [20]. The focus did not shift given identification of 14 of 18 tolyporphins as dioxobacteriochlorin types, and has been sharpened with repeated finding of tolyporphin A as the most abundant member of the tolyporphins family. By contrast, tolyporphin P is a latecomer to the tolyporphins family, having only been reported in 2020 by the Williams group (and isolated in 1.0 mg quantity) [48]. One consideration about tolyporphin P and tolyporphyrinogen P concerns their inherent hydrophobicity. Tolyporphin P, for example has a cLog*P* value of 5.0 (Table 3), which is a reflection of the absence of any polar substituents. Whether the hydrophobic nature of tolyporphin P was a factor in its only recent isolation is unclear.

**Figure 64:**
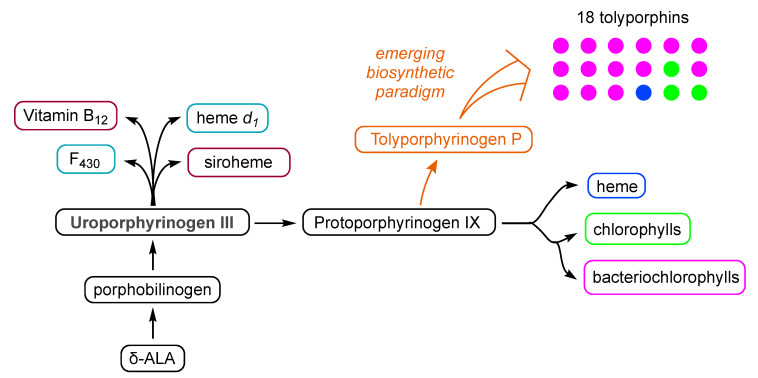
Outline of landmarks in the biosynthesis of tetrapyrrole macrocycles including the tentative picture of the origin of tolyporphins via late-stage derivatization of tolyporphyrinogen P. Much additional experimental work is required to confirm the emerging biosynthetic paradigm sketched here. Tolyporphins may be distinct, if not unique, among tetrapyrrole macrocycles in comprising a diverse set of natural products. Heretofore, tetrapyrrole biosynthesis has been known to afford singular end products (e.g., vitamin B_12_, F_430_) or a small set of tailored products (e.g., chlorophylls) wherein each typically serves a specific biological role in conjunction with a complementary protein host.

The focus on tolyporphyrinogen P is provocative and intriguing especially given the parallel with uroporphyrinogen III as the last universal precursor of all members of the pigments-of-life family (Figure 11 and Figure 64). Thus, the Abe work suggests tolyporphyrinogen P as the last universal precursor of all tolyporphins. If so, the gem-dialkyl substitution features in dioxobacteriochlorin-type and oxochlorin-type tolyporphins would form in a biosynthetic branch unrelated to those that form the other known gem-dialkyl-containing hydroporphyrins (e.g., cobalamin, F_430_, siroheme, heme *d*_1_).

The origin of the family of tolyporphins in the context of the established biosynthesis of the pigments of life [58] is sketched in Figure 64. The sketch shows the provisional late-stage diversification from the scaffold tolyporphyrinogen P to form the family of tolyporphins. Beyond biosynthesis, if tolyporphyrinogen P is indeed established as such a versatile workhorse intermediate, opportunities sparkle for chemoenzymatic manipulation and semisynthetic derivatization of this potential molecular chassis. The four open β-pyrrole positions are distinct features of tolyporphyrinogen P and tolyporphin P, and are ripe for substitution with a broad array of electrophiles.

## 17. Potential Pharmacological Properties of Tolyporphins

Tolyporphins were discovered through application of a biological assay to a Linnaean collection of cyanobacteria. This section describes the studies that have been carried out concerning the biological properties of tolyporphins. The biological properties of initial and ongoing interest are those that led to the discovery of tolyporphins in the first place: as potential antineoplastic agents in treatment of human disease. A complementary, and scientifically more fundamental, interest concerns the native role of tolyporphins in the cyanobacterium–microbial consortium. More is known at present about the potential pharmacological roles of tolyporphins than why tolyporphins are produced by the cyanobacterium–microbial consortium. Indeed, the endogenous role of tolyporphins remains largely unexplored.

### 17.1. Reversal of Multidrug Resistance

The initial communication by Prinsep et al. in 1992 [20] concerning the discovery and general structure elucidation of tolyporphin A did not contain extensive information about the methods of cyanobacterial screening. That information could be pieced together from an earlier report by Patterson et al. in 1991 [19], and a 1994 report by Smith et al. [21] concerning potential pharmacological properties of tolyporphin A.

The 1991 report by Patterson et al. [19] describes an initial screen of lipophilic extracts from cyanobacteria for antineoplastic activity in KB cells (a human nasopharyngeal carcinoma cell line [22]). In this screen, 19 of 291 strains (and later, 54 of 702 strains) yielded positive results [19]. No mention of HT-58-2 appears therein.

The 1994 report by Smith et al. [21], then focused on tolyporphin A, delineated a “bioactivity-directed fractionation process” that had actually been used in the search that led to identifying tolyporphin A [21]. Two cell types were chiefly used [203]: (1) SKVLB1 cells, which over-express P-glycoprotein and hence pump-out xenobiotics from the intracellular space, thereby thwarting the action of anti-cancer drugs; and (2) SKOV3 cells, which lack the over-expressed P-glycoproteins and hence are drug-sensitive (thereby providing a negative control). The SKOV3 cells are from a human ovarian carcinoma, whereas SKVLB1 is a subline that was selected for resistance to vinblastine. The P-glycoprotein is a membrane-spanning protein that functions as an energy-dependent, broad-spectrum, drug efflux pump [45,204].

Extracts from the collection of cyanobacterial strains were incubated with the cells along with the presence or absence of anti-cancer agents daunomycin, vinblastine, or actinomycin D; antiproliferative activity and cell toxicity were assessed after 48 hours. Four classes were identified: (I) Neither cytotoxic nor able to potentiate the effects of the three anti-cancer agents (90% of the extracts). (II) Significant toxicity to SKOV3, but not SKVLB1 cells (5% of the extracts). (III) Similar toxicities to both SKOV3 and SKVLB1 cells (~3% of the extracts). (IV) Little or no cytotoxicity, but able to potentiate the activity of daunomycin, vinblastine, or actinomycin D (~2% of the extracts). Case IV was most interesting in a search for compounds with the ability to reverse multidrug resistance.

In reflection by Moore on the screening program begun at Hawaiʻi in 1981 [205], “Extracts of more than 1500 strains representing some 400 species of blue-green algae were tested over the next 12 years [18], using mostly cell-based assays to discover new anticancer, antifungal, and antiviral agents. Six percent of the extracts were cytotoxic against human tumor cell lines at MICs <20 μg mL^−1^ [19]; however, less than 1% of the extracts were solid tumor-selective [206] and/or tumor-selective. Some of the non-cytotoxic extracts (<1%) showed multiple-drug-resistance (MDR)-reversing activity”. Elsewhere, the authors point out that the screen of extracts of cyanobacteria for anti-multidrug resistance activity experienced a “hit” rate of about 1% [170]. Upon identification of a hit extract, the source cyanobacterium was grown at scale and the cell-based assay was used to guide the purification process. Tolyporphin A was the first multidrug resistance-reversing cyanobacterial natural product identified in the Hawaiʻi program [21].

Studies by Smith et al. showed that tolyporphin A in the multidrug resistance assays inhibited proliferation of SKOV3 and SKVLB1 cells with IC_50_ values of 0.8 and 4 μM, respectively [21]. Tolyporphin A sensitized the cell lines SKVLB1 and MCF-7/ADR (now known to be an ovarian cancer cell line) [207,208], which also over-express P-glycoprotein, to daunomycin, taxol, actinomycin D, and colchicine, “without increasing cell sensitivity to compounds that are not subject to removal by P-glycoprotein, i.e., cisplatin and mephalan” [21]. Tolyporphin A was a more effective agent in reversing multidrug resistance than verapamil, with sensitization of SKVLB1 cell lines at concentrations of 0.1 μM [21].

Tolyporphin A blocked the ability of [^3^H]-azidopine to label the P-glycoprotein via photoaffinity procedures. The binding of [^3^H]-vinblastine to cell membranes containing the P-glycoprotein was completely abrogated with 25 μM tolyporphin A [21]. All of the results taken together at the time led to the interpretation that tolyporphin A was a potent substrate for binding to P-glycoprotein, blocking cellular efflux pump activity, and thereby reversing multidrug resistance of cancer cell lines.

The discovery of tolyporphins B–I led to studies similar to those above [45]. Competition for the [^3^H]-azodipine binding site of P-glycoprotein decreased in the following order: tolyporphins D, G/H > verapamil > tolyporphins A-C, F > tolyporphins E and I [45]. For increasing the accumulation of [^3^H]-vinblastine in MCF-7/ADR cells, the order decreased as follows: tolyporphins F, G/H (fivefold at 0.5 μg/mL) > verapamil > tolyporphins A, E, I (3–6-fold at 10 mg/μL) >> tolyporphins B–D (inactive up to at least 10 μg/mL) [45].

The discovery of tolyporphins J and K also led to studies similar to those above. Tolyporphin J was about twofold less potent than tolyporphin A in increasing the accumulation of [^3^H]-vinblastine by MCF-7/ADR cells, and both were more potent than verapamil. On the other hand, tolyporphin K only yielded modest increases in the accumulation of [^3^H]-vinblastine [46].

The authors highlighted a key point: while tolyporphins are cytotoxic at doses 5–10-fold higher than those necessary for inhibition of P-glycoprotein, which comprises a fairly narrow therapeutic window, cautious interpretation is advised because of the distinct mechanisms involved. A possible complicating feature stems from the production of reactive oxygen species (ROS) by the strong, visible-light absorption of tolyporphins [46]. In general, studies carried out with photoactive tetrapyrrole macrocycles in the presence of ambient lighting are now known to likely generate ROS, which can occur in parallel with, or perhaps central to, observed phenomena. In this regard, the authors stated that “these porphyrin-based compounds pose a unique difficulty” given that negligible light would be available in vivo, hence the laboratory studies of the therapeutic index underestimate the practical utility of the tolyporphins for use as multidrug reversal agents [46]. Some caveats and reconsiderations of the aforementioned findings followed a dozen years later upon studies by Prinsep and coworkers [209] concerning cytotoxicity effects of tolyporphin A in the presence of light. In retrospect, the aforementioned [^3^H]-vinblastine binding assay and photolabeling studies may not provide evidence beyond reproach of the binding of tolyporphin A to P-glycoprotein. The cytotoxicity of tolyporphins may stem from a photodynamic effect that generates ROS more so than by physical binding as an efflux pump inhibitor to P-glycoprotein, as stated by Prinsep and coworkers upon reevaluation in later studies [209]. The photodynamic features of tolyporphins are described in the next two subsections.

### 17.2. Photodynamic Inactivation

Numerous tetrapyrrole macrocycles, including bacteriochlorins, have been shown to be potent agents for the generation of a photodynamic effect [210]. The phenomenon upon treating cells in vitro or of microbial infections in vivo is typically termed photodynamic inactivation (PDI); the same phenomenon when ameliorating tumors in vivo is referred to as photodynamic therapy (PDT). Morlière et al. studied the utility of tolyporphin A as a PDI agent against EMT-6 (murine mammary) tumor cells [52]. To summarize extensive studies: (i) The quantum yield of formation of singlet oxygen upon illumination of tolyporphin A was estimated to be 0.25. (ii) The photoactivated cellular killing efficacy of tolyporphin A was ~5000 times greater than that of Photofrin II, a well-known porphyrin-based PDT agent, where efficacy is assessed on the light dose necessary to kill 50% of the cells and the concentration of the photoactive agents. (iii) Comparison of a dioxobacteriochlorin with a porphyrin pits a strong red-region absorber versus a weak red-region absorber, respectively. Pheophorbides, which are derivatives of chlorophyll wherein the magnesium has been removed and the phytyl group has been removed or replaced, retain strong absorption in the red region of the spectrum [70]. Tolyporphin A exhibited 6 times or 70 times greater cellular killing efficacy versus that of two pheophorbides. (iv) Tolyporphin A localizes in the endoplasmic reticulum of the EMT-6 tumor cells [52].

### 17.3. Studies with Metal Chelates of Tolyporphins

Inspection of the general structure of tetrapyrrole macrocycles suggests facile metal chelation. Such is the case with porphyrins and many metals. Bacteriochlorins, on the other hand, are known to undergo metalation with less facility [72,211]. Indeed, tolyporphin A upon exposure to copper triflate [Cu(O_3_SCF_3_)_2_] in methanol containing triethylamine at room temperature yielded no observable copper insertion [209], conditions that would readily metalate a porphyrin, whereas elevated temperature did afford metalation. The copper(II) chelate of tolyporphin E, and the Ag(II) chelates of tolyporphins A and E, were also prepared. No detectable metalation occurred with either tolyporphin A or E upon exposure to Zn(II), Ni(II), Cd(II), Pb(II), Fe(II), Fe(III), V(II)O, Mg(II), or Pd(II), each containing counteranions that are amenable to metalation with porphyrins.

The copper(II) complexes of tolyporphins A and E were ~10-fold less cytotoxic than the parent, free base tolyporphins. The silver(II) complex of tolyporphin A was threefold less cytotoxic than the parent tolyporphin, whereas the silver(II) complex of tolyporphin E was threefold more cytotoxic than the parent tolyporphin.

The cytotoxic effect of tolyporphin A was tenfold less in the dark than in the light [209]. In the presence of the antioxidant α-tocopherol (or γ-tocopherol), the concentration-dependent cytotoxicity of tolyporphin A was diminished, but no diminution occurred upon examination of a set of other free radical scavengers. The latter included membrane-soluble and aqueous-soluble species. A further surprise was that α-tocopherol also greatly diminished the reversal of multidrug resistance of tolyporphin A in SKVLB1 cells. The variable results with the copper(II) and silver(II) complexes in the cytotoxicity and multidrug resistance reversal experiments were attributed to distinct capacities for free radical formation. The authors state that free radicals generated by tolyporphin A seem to mediate both its cytotoxicity and inhibition of P-glycoprotein activity [209]. While the decrease in cytotoxicity in the presence of a radical scavenger suggests the involvement of free radicals, the absence of an effect by other free radical scavengers may point to a specific interaction of tolyporphin A alone or at the sites where tolyporphin A exerts cytotoxicity. Moreover, copper(II) chelates of tetrapyrrole macrocycles are not thought to be sufficiently photoactive to generate ROS. Additional studies will be required to draw more definitive conclusions. Access to larger quantities of tolyporphins would be essential to do so.

### 17.4. Computational Studies

**Figure 65:**
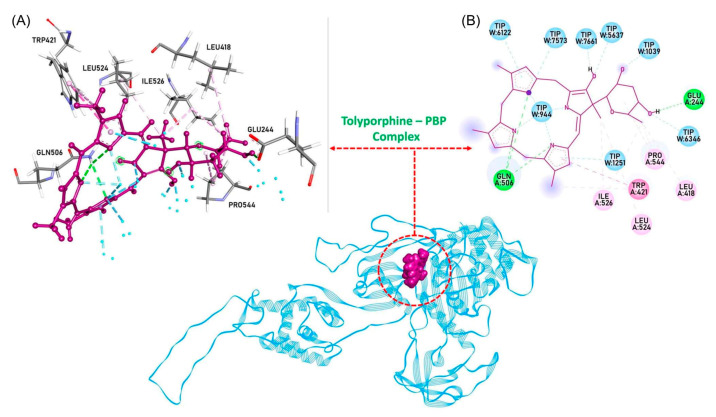
Interaction of tolyporphin K with the penicillin-binding protein. (**A**) Ball and stick model showing key interactions. (**B**) Interactions of tolyporphin K with water (blue circles) and specific amino acid residues [108]. Reprinted with permission from ref [108]; 2023, Taylor and Francis (Informa UK Limited).

In an in silico search for antibacterial lead compounds, 85 compounds from cyanobacteria were examined in docking studies with the penicillin-binding protein from *Acetinobacter baumannii* [108]. The compounds were drawn from the following classes: abietane, diterpenes, alkaloids, cyclic peptides, cyclophane, diterpenoids, hapaloindoles, lipopeptides, macrolides, polyketides, and porphinoids. Protein–ligand docking studies flagged tolyporphin K, Noscomin, Calothrixin, and Nitrocefin (a control compound) as having the highest affinity for the penicillin-binding protein. Further binding studies among this group identified the oxochlorin tolyporphin K as the tightest binding ligand (–8.9 kcal/mol). A three-point transferable interaction potential (TIP3P) was used to mimic the presence of water molecules around the protein. Molecular dynamics simulations indicated specific molecular interactions that showed little fluctuations over the course of 100 ns [108]. The structure of tolyporphin K bound to the penicillin-binding protein is shown in Figure 65. The authors suggest that the binding of tolyporphin K in a conserved domain of the penicillin binding protein suggests possible utility as an inhibitor against microbial pathogens [108]. Further studies are required to gain a deeper understanding of the properties of tolyporphins and possible utility as lead pharmaceutical compounds.

The studies listed above delineate features of tolyporphins identified through experiments with non-cyanobacterial cell types and, through simulation, with proteins from non-cyanobacterial organisms. Such studies focus on possible beneficial roles of tolyporphins as potential pharmacological agents in treatment of human disease. Deep questions remain concerning the *native* physiological function(s) of tolyporphins in the HT-58-2 cyanobacterium. Understanding why tolyporphins are present in a cyanobacterium is one of the three questions that have captivated the present authors, as described in Section 4.3). Several hypotheses have been put forth. The endogenous roles of tolyporphins might include the following:

(1) As a photoprotective agent by absorption of light, given that the absorption spectrum of a dioxobacteriochlorin-type tolyporphin resembles that of chlorophyll [49]; indeed, cyanobactera are known to make scytonemins, which provide a sunscreen-like function to protect the organisms from the adverse effects of ultraviolet light [212,213].
(2) As a photo-toxin by production of ^1^O_2_ following absorption of light, serving as a defense function against other organisms [49,106].
(3) As an efflux pump inhibitor of the cyanobacterium itself or of attached or proximal community bacteria, serving as a defense mechanism against exogenous molecular toxins [49,106].

In short, despite considerable work, the jury remains out concerning the “why tolyporphins” question.

## 18. Perspective

A Linnaean search some 35 years ago to collect novel cyanobacterial samples led, inter alia, to the identification of not only a new tetrapyrrole macrocycle, but an entire new family of tetrapyrrole macrocycles—the tolyporphins—and in so doing ushered in new directions in research. Studies centered around tolyporphins have since occupied multiple research teams from France, India, Japan, New Zealand, and the United States (Hawaiʻi, Massachusetts, North Carolina, and Pennsylvania). The individuals in tolyporphins research number 81 on authored papers [19,20,21,30,38,39,40,41,42,45,46,47,48,49,51,52,69,79,88,92,104,106,108,112,120,133,164,202,209,214].

The research has been multifaceted, touching on analytical chemistry, assay development, bioinformatics, biosynthesis, biosynthetic gene clusters, computational simulations, evolutionary diversity, genomics, heterologous expression, imaging, mass spectrometry, metabolism, microbial consortia, molecular structure elucidation, multidrug resistance, organic synthesis, pharmacology, photophysics, phylogenetics, phytochemical analysis, semisynthesis, and so on, a set of topics so rich and fecund as to conjure the ‘world in a grain of sand’ [215] metaphor.

Advances to date owe to the courage of Moore and Patterson to launch the global search for diverse cyanobacteria; the persistence and acumen of Prinsep to identify tolyporphins A–M; the single-minded dedication of Williams to find the lost culture of HT-58-2 and then identify tolyporphins N–R, along with structural elucidation studies across the family of tolyporphins; the incisive syntheses of Kishi; and the decisive biosynthetic advances of Abe. Our own groups have worked on understanding the cyanobacterium–microbial consortium, cyanobacterial genome, biosynthesis and gene clusters, molecular diversity, and methods for quantitation and analysis of tolyporphins. Much has been learned, yet the growing knowledge base seems dwarfed by the magnitude of the unanswered outstanding questions. In this regard, the Wilsonian objective [1] “*to find and take full account of each and every species of organism on Earth”* remains unfulfilled for tolyporphins and the HT-58-2 culture. A short list of unanswered questions includes the following:

(1) What?—the molecular structures of tolyporphins have been fairly established, although stereochemical configuration remains to be elucidated for a number of members. The molecular diversity suggests that tolyporphins are secondary metabolites (natural products), not the singular end product of core metabolism. While the molecular structures of tolyporphins have largely been elucidated, studies of electronic and tautomeric features remain to be carried out (for components other than tolyporphin A [49]); results in this regard can be compared with those for a variety of synthetic lactone-containing tetrapyrrole macrocycles [216].
(2) Why?—the microbial–physiological rationale for the biosynthesis of tolyporphins is unclear, although the increase in production upon nitrogen stress is a hallmark of a microbial defensive function. Suggestions for physiological function include an efflux pump inhibitor in the cyanobacterium; a photodynamic agent; or a photoprotective agent. The existential question concerning dioxobacteriochlorins in an organism that ordinarily produces a chlorin (i.e., chlorophyll *a*) in ample quantity remains perhaps almost koanistic. Could it be that tolyporphins have hardly any native physiological function, and the biosynthetic machinery is merely a vestigial rarity of no significance other than an intriguing scientific curiosity?
(3) Where?—imaging experiments have shown tolyporphins in the cyanobacterial envelope and septa between cyanobacterial cells, yet the questions of how tolyporphins arrived there, and where they arrived from, are unknown. The extent to which one or more community bacteria may participate in the biosynthesis remains undetermined.
(4) When?—the evolutionary origin of tolyporphins is unknown. The finding of additional native producers beyond that of the now sole producer HT-58-2 (among the universe of cyanobacteria and other organisms) would help to inform evolutionary considerations.
(5) How?—knowledge of the biosynthesis is rapidly advancing given heterologous expression of genes from the putative BGCs of the HT-58-2 cyanobacterium, as well as *Oculatella* sp. LEGE 06141. Understanding how hydrocarbon (vinyl) groups are removed from the β-pyrrolic positions downstream from uroporphyrinogen III, presumably by the TolI enzyme, is of utmost interest. Research on this topic is under active investigation [217]. One pathway entails formation of tetravinylporphyrinogen P, followed by hydration and loss of acetaldehyde from each position, thereby forming tolyporphyrinogen P. The role of tolyporphyrinogen P as a possible intermediate for late-stage diversification to form the entire repertoire of tolyporphins is a recent and intriguing hypothesis. The presence of the gem-dialkyl motifs in tolyporphins (all except P, which is a porphyrin) has structural relationship with the hydroporphyrins siroheme, heme *d_1_*, cobalamin and F_430_; but such may be purely coincidental—the results of Abe and coworkers indicate that tolyporphins emanate from a new biosynthetic branch in the tree of the pigments of life. The question of whether there are protein hosts or chaperones for tolyporphins (and tolyporphyrinogens), which are relatively hydrophobic structures, is unknown.

The great lacuna from a chemistry perspective is the absence of the power of organic synthesis to make available the entire gamut of tolyporphins. The deservedly celebrated synthetic work of Minehan, Wang, and Kishi provided tolyporphin A diacetate and a diastereomer thereof, each in tiny quantity. Still, no full route to tolyporphin A or other native tolyporphins has yet been established. The semisynthetic reactions of tolyporphins to date include *O*-acetylation, *O*-deacetylation, *O*-butanoylation, metal chelation, and ketone reduction; the totality of such reactions pales against that known for native bacteriochlorophylls. Synthetic routes to gem-dialkyl-substituted chlorins and bacteriochlorins are available [218], but much remains to be done to accommodate the peripheral substituents that adorn the various tolyporphins. Efficacious access to an abundance of each member of the repertoire of tolyporphins, including precursors and analogues and derivatives, would open doors to study biosynthesis and physiology.

**Figure 66:**
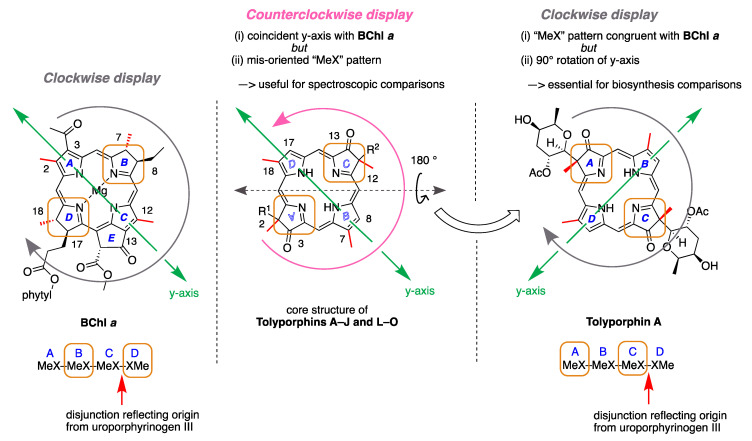
Displays of line drawings of tolyporphins and bacteriochlorophyll *a* (BChl *a*). The *clockwise* and *counterclockwise* displays are defined by readout of the MeX–MeX–MeX–XMe substituent pattern upon circumnavigating the molecule from positions 2 to 18. The *clockwise* and *counterclockwise* displays of tolyporphins are germane to studies of biosynthesis and spectroscopy, respectively, upon comparison with native photosynthetic tetrapyrroles, such as bacteriochlorophyll *a*.

Finally, we close with consideration of line drawing displays of tolyporphins. The structure of bacteriochlorophyll *a* is shown in Figure 66 in the customary manner [65]: rings A–D are labeled circumnavigating the macrocycle in *clockwise* fashion, starting with the upper left ring. In so doing, the pyrroline units are labeled B and D; and the *y*-axis bisects the nitrogen atoms of the pyrrole rings (as also shown in Figure 12 and Figure 13). One display of dioxobacteriochlorin-type tolyporphins is shown at the center of Figure 66 (also in Figure 6, Figure 7, Figure 13, Figure 42 and Figure 45). The display causes the pyrroline units and *y*-axis to cohere with those of bacteriochlorophyll *a;* however, the all-informative pattern of peripheral methyl groups is incongruent upon superposition of the respective displays. The telltale MeX–MeX–MeX–XMe pattern of the tolyporphins displayed at the center of Figure 66 is oriented *counterclockwise* (beginning at lower left) to that of bacteriochlorophyll *a.* Rotation of the tolyporphins macrocycle in the center about the horizontal axis by 180° yields the *clockwise* display shown at right in Figure 66 (also in Figure 3, Figure 5, Figure 12 and Figure 38). For the *clockwise* display of tolyporphin A, the pattern of peripheral methyl groups coheres upon superposition with that of bacteriochlorophyll *a*, but the pyrroline units and *y*-axis are misaligned with their respective counterparts.

The question is not which display is correct and which is not, as both are accurate molecular depictions, but rather which display is more appropriate and insightful? For spectroscopic comparisons of tolyporphins with other native hydroporphyrins (e.g., bacteriochlorophylls and chlorophylls), the *counterclockwise* display at the center of Figure 66 is most meaningful. For biosynthetic comparisons, the *clockwise* display at the right of Figure 66 is most meaningful. The *clockwise* displays illustrate a key biosynthetic issue. In bacteriochlorophyll *a*, it is rings B and D that undergo reductive saturation upon conversion from protoporphyrin IX. In the biosynthesis of dioxobacteriochlorin-type tolyporphins, it is rings A and C that undergo reduction. The pattern of methyl groups is an inviolate signature reflecting origin from uroporphyrinogen III, the last universal precursor to all native tetrapyrroles, including the entire mesmerizing repertoire of tolyporphins.

## Figures and Tables

**Table 1:** Photophysical properties of tolyporphin A, a synthetic dioxobacteriochlorin, and photosynthetic pigments *^a^.*

Compound	B_y_ λ_abs_ (nm)	B_x_ λ_abs_ (nm)	Q_y_ λ_abs_ (nm)	Q_y_ λ_em_ (nm)	τ_s_ (ns)	Φ_f_	Φ_isc_	Φ_ic_
tolyporphin A	397	406	679	681	3.9	0.14	0.77	0.09
**H_2_BC-O^7,17^**	391	401	680	682	3.8	0.16	0.77	0.07
pheophytin *^a b^*	400	415	671	676	6.7	0.24	0.60	0.16
chlorophyll *^a b^*	413	432	665	671	6.4	0.33	0.55	0.12
bacteriopheophytin *^a c^*	363	389	758	768	2.7	0.1	0.57	0.33
bacteriochlorophyll *^a d^*	363	396	781	789	3.1	0.12	0.30	0.58

*^a^* All values were measured using argon-purged toluene samples at room temperature. *^b^* Wavelengths and Φ_f_ from ref [73]. *^c^* From ref [71]. *^d^* From ref [72].

**Table 2:** Photophysical properties of tolyporphin A *^a^.*

Solvent	SDC *^b^*	B_x_ λ_abs_ (nm)	Q_y_ λ_abs_ (nm)	Q_y_ λ_em_ (nm)	τ_s_ (ns)	Φ_f_	Φ_isc_	Φ_ic_
toluene	2.4	406	679	681	3.9	0.14	0.77	0.09
diethyl ether	4.3	401	678	679	4.2	0.11	0.81	0.08
ethyl acetate	6.1	402	677	678	3.9	0.12	0.80	0.08
dichloromethane	8.9	406	679	680	3.7	0.11	0.83	0.06
pentan-1-ol	13	404	677	678	4.4	0.11	0.82	0.07
butan-2-one	18.5	402	677	682	4.1	0.13	0.82	0.05
ethanol	24.6	405	676	677	4.1	0.12	0.81	0.07
methanol	32.7	402	676	677	4.3	0.11	0.85	0.04
DMF *^c^*	36.7	404	678	680	3.8	0.11	0.79	0.10
dimethyl sulfoxide	46.7	406	679	680	3.9	0.11	0.79	0.10

*^a^* All values were measured using argon-purged samples at room temperature. *^b^* This term is the solvent static dielectric constant, typically indicated by ε, which we avoid here to prevent confusion with the molar absorption coefficient, which also is indicated by the ε symbol. *^c^* *N*,*N*-dimethylformamide.

**Table 3:** Table of partition coefficients for tetrapyrrole macrocycles.

Entry	Tetrapyrrole Macrocycle	cLog*P* Value *^a^*
1	Tolyporphin A	3.4
2	Tolyporphin B	2.6
3	Tolyporphin C	2.6
4	Tolyporphin D	1.7
5	Tolyporphin E	2.6
6	Tolyporphin F	1.9
7	Tolyporphin G	1.7
8	Tolyporphin H	1.7
9	Tolyporphin I	2.4
10	Tolyporphin J	0.9
11	Tolyporphin K	3.1
12	Tolyporphin L	2.2
13	Tolyporphin M	2.2
14	Tolyporphin N	2.2
15	Tolyporphin O	2.2
16	Tolyporphin P	5.0
17	Tolyporphin Q	3.0
18	Tolyporphin R	3.7
19	Chlorophyll *a*	14.8
20	Bacteriochlorophyll *a*	13.3

*^a^* Calculated here using Chemdraw 22.2.0.

**Table 4:** Amount of tolyporphins from HT-58-2 obtained >20 years apart.

Component *^a^*	1995 Culture *^b^*		2017 Culture *^e^*		2017 vs. 1995 *^f^*
	Amount (mg)	% vs. Cells	Amount (mg)	% vs. Cells	
cells	93,000		79,500		
extract	7500		4750		
A	123	0.13	6.2	0.008	–17 X
B + C	38 (4:1)	0.032, 0.008	3.0	0.0038	–11 X
D	5	0.005	0.8	0.001	–5 X
E	46	0.049	2.0	0.0025	–19 X
F	12	0.013	0.5	0.0006	–21 X
G + H	-- (2:1)	0.004, 0.002	1.0	0.0013	–5 X
I	1.5	0.002	0.5	0.0006	–3 X
J	1.5 *^c^*	0.0016 *^c^*	--	--	--
K	1 *^c^*	0.0011 *^c^*	0.4	0.0005	–2 X
L + M	3.5 (2:1) *^d^*	0.0025, 0.0013 *^d^*	--	--	--

*^a^* Components are as follows: “cells” refers to dried cell mass; “extract” is the lipophilic extract of the dried cell mass; A–M are tolyporphins. *^b^* All data are from ref [45] unless noted otherwise. The cell mass was 93 g; the lipophilic extract was 7.5 g. *^c^* Data reported in ref [46]. *^d^* Data reported in ref [47]. *^e^* Data reported in ref [69]. *^f^* The factor of change in the % yield in 2017 versus that in 1995.

**Table 5:** Relative amount of tolyporphins upon growth in different media [48,104].

Component	Relative Abundance of Tolyporphins	
	A3M7 Medium	BG-11 Medium
A	22.0%	11.0%
B + C	1.7%	1.6%
D	0.8%	1.0%
E	11.4%	2.8%
F	6.0%	3.9%
G + H	1.5%	1.7%
I	13.5%	9.4%
J	0.5%	1.2%
K	4.8%	4.7%
L + M + N + O	0.6% *^a^*	0.9% *^a^*
P	13.1%	20.4%
Q	5.2%	2.7%
R	18.8%	38.7%

*^a^* Tolyporphins L–O are isobaric and coelute, so only the total amount could be estimated.

**Table 6:** Tolyporphins formulas and structural features.

Tolyporphin	Formula	Mol wt (Da)	# of Sugars	Chromophore
A	C_40_H_46_N_4_O_10_	742.83	2	dioxobacteriochlorin
L, M, N, O	C_38_H_44_N_4_O_10_	716.79	2	dioxobacteriochlorin
B, C	C_38_H_44_N_4_O_9_	700.79	2	dioxobacteriochlorin
D	C_36_H_42_N_4_O_10_	690.75	2	dioxobacteriochlorin
E	C_34_H_36_N_4_O_8_	628.68	1	dioxobacteriochlorin
F	C_32_H_34_N_4_O_7_	586.64	1	dioxobacteriochlorin
I	C_28_H_26_N_4_O_6_	514.54	0	dioxobacteriochlorin
G, H	C_26_H_24_N_4_O_5_	472.50	0	dioxobacteriochlorin
J	C_24_H_22_N_4_O_4_	430.46	0	dioxobacteriochlorin
K	C_30_H_32_N_4_O_4_	512.61	1	oxochlorin
R	C_26_H_24_N_4_O_3_	440.50	0	oxochlorin
Q	C_24_H_22_N_4_O_2_	398.47	0	oxochlorin
P	C_24_H_22_N_4_	366.47	0	porphyrin

**Table 7:** Absorption spectral data for tolyporphins [69,104].

Tolyporphin	Solvent	Absorption in nm (ε in M^−1^cm^−1^)		fwhm (Q_y_), nm	mg *^g^*	Reference	Proposed (ε in M^−1^cm^−1^)
		Soret Band	Q_y_ Band				Q_y_ Band
A	MeOH	401 (49,000)	675 (22,000)	7.7	---	[20]	100,000
A	EtOH	402 (148,000)	676 (68,500)	--	---	[52]	100,000
**Wang-1**	CH_2_Cl_2_	406 (107,463)	678 (44,600)	8.9 *^f^*	0.38	[42]	100,000
**Minehan-2**	CH_2_Cl_2_	407 (110,500)	684 (51,530)	10.8	0.5	[120]	100,000
B, C	MeOH	406 (2100) *^c^*	680 (13,000)	9.0	38	[45]	100,000
D	MeOH	368 (24,000)	680 (12,000)	8.9	5	[45]	100,000
E	MeOH	388 (16,000)	686 (7300)	9.4	46	[45]	100,000
F	MeOH	386 (39,000)	684 (22,000)	15.3	12	[45]	100,000
G, H	MeOH	368 (18,000)	686 (10,000)	11.2	--	[45]	100,000
I	MeOH	376 (14,000)	684 (7800)	9.6	1.5	[45]	100,000
J	MeOH	396 (7900)	688 (1500)	--	1.5	[46]	100,000
L, M	MeOH	401 (130,000)	677 (50,000)	--	0.9	[48]	100,000
N, O	MeOH	401 (130,000)	677 (50,000)	--	2.3	[48]	100,000
K *^a^*	MeOH	397 (3500)	635 *^d^* (380)	10.6	1	[46]	35,000
Q *^a^*	MeOH	394 (63,000)	666 (5000)	--	3.5	[48]	35,000
R *^a^*	MeOH	395 (50,000)	636 (13,000)	--	1.0	[48]	35,000
P *^b^*	CHCl_3_	398 (79,000)	619 *^e^* (1300)	--	1.0	[48]	14,500 *^h^*

*^a^* An oxochlorin. *^b^* A porphyrin. *^c^* A likely typographic error. *^d^* Other longer wavelength bands with weaker intensity are present. *^e^* In the porphyrin, this is the Q_x_(0,0) band. *^f^* Kishi, personal communication (14 January 2015 and 7 July 2017) from the notebook of coworker Dr. W. Wang; see ref [42]. *^g^* Quantity of sample used to determine the spectral features, including ε, in the columns to the left. *^h^* The value is given for the dominant band in the Q manifold, the Q_y_(1,0) band.

**Table 8:** Chlorophyll *a* content in cyanobacteria.

Organism	Chl *a*/Organism *^b^*	%	Reference
*Oscillatoria brevis*	12–16 mg/g	1.2–1.6	[124]
*Spirulina* sp.	7.2 mg/g	0.72	[125]
*Nostoc muscorum*	2.61–3.80 μg/g	0.00026–0.00038	[126]
HT-58-2 (BG-11) *^a^*	9.1–17.0 mg/g	0.91–1.7	[69]
HT-58-2 (BG-11_0_) *^a^*	4.4–10.1 mg/g	0.44–1.01	[69]

*^a^* 2017 culture. *^b^* Dried weight of the organism.

**Table 9:** Tolyporphins and mass spectral detection methods [69].

Tolyporphin	Mass *^a^*	*LE1 ^b,c^*		HPLC fractions *^c^*	
		MALDI-MS	ESI-MS	Abs *^d^*	MALDI-MS *^e^*
A	742.3214	+	+	+	+
L, M	716.3057	−	+	−	−
B, C	700.3108	+	+	+	+
D	658.3003	+	−	+	+
E	628.2533	−	+	+	−
F	586.2428	−	+	+	−
I	514.1852	−	+	+	−
K	512.2424	+	+	+	−
G, H	472.1747	−	+	+	−
J	430.1641	+	+	−	−

*^a^* Calculated exact mass. *^b^* From a small-scale (50 mL) culture without chromatographic fractionation. *^c^* The +/− indicates detected or not detected, respectively. *^d^* Absorption spectral detection upon HPLC analysis from a large-scale culture. *^e^* Data for isolated tolyporphins after HPLC separation of ***LE1***.

**Table 10:** The *tol* genes [164].

Gene	Possible Function	Start	End	Size (bp)	Direction
*tolA*	dTDP-glucose 4,6-dehydratase	2,586,793	2,587,887	1095	+
*tolB*	glucose-1-phosphate thymidylyltransferase	2,587,907	2,588,851	945	+
*tolC*	acyltransferase	2,592,203	2,593,597	1395	+
*tolD*	glycosyltransferase	2,595,115	2,596,395	1281	+
*tolE*	UDP-glucose 4-epimerase	2,596,678	2,597,817	1140	+
*tolF*	aminotransferase	2,598,123	2,599,451	1329	+
*tolG*	cytochrome P450	2,600,647	2,602,017	1371	+
*tolH*	cytochrome P450	2,602,062	2,603,444	1383	+
*tolI*	L-2-amino-thiazoline-4-CO_2_H hydrolase	2,604,869	2,605,492	624	+
*tolJ*	FAD-binding protein	2,605,501	2,606,913	1413	+
*tolK*	aldo/keto reductase	2,608,856	2,609,884	1029	−

**Table 11:** Putative BGCs for natural products identified in HT-58-2 [88].

Number	Region (bp)	Length (nt)	Type	Similar Known Cluster
4	958,778–1,011,938	53,161	hglE-KS, *^a^* T1PKS *^b^*	heterocyst glycolipids
9	2,268,098–2,291,911	240,666	NRPS, *^c^* T1PKS	hapalosin
11	2,683,411–2,768,967	85,557	T1PKS	anatoxin-a
15	4,105,987–4,016,220	42,348	NRPS	shinorine

*^a^* Heterocyst glycolipid synthase-like polyketide synthase (PKS). *^b^* Type I polyketide synthase (PKS). *^c^* Non-ribosomal peptide synthetase cluster.

**Table 12:** Cyanobacteria with putative BGCs for tolyporphins [164].

Strain	Location	Origin	Number of Genes		Reference
			*hem*	*tol*	
HT-58-2 BGC-1	Pohnpei, Micronesia	Soil	7	11	[79,164]
HT-58-2 BGC-2	Pohnpei, Micronesia	Soil	3	7	[164]
*Nostoc* sp.106C	Chiapas, Mexico	Coralloid roots	6	7	[180]
*Nostoc* sp. RF31YmG	Chiapas, Mexico	Coralloid roots	6	7	[180]
*Nostoc* sp. FACHB-892	Tengger Desert, China	Soil crusts	6	6	[181]
*Brasilonema octagenarum* UFV-OR1	Minas Gerais, Brazil	Orchid leaves	8	6	[82]
*Brasilonema octagenarum* UFV-E1	Minas Gerais, Brazil	Eucalyptus grandis leaves	8	6	[82]
*Brasilonema sennae* CENA114	São Paulo, Brazil	Iron water pipe	8	6	[82]
*Oculatella* sp. LEGE 06141	Praia de Luz, Lagos, Portugal	Green macroalgae in an intertidal zone	6	9	[164]

**Table 13:** Pyrroline substituents in tolyporphins [66].

Substituent		Tolyporphin Where the Substituent Is Found
Abbreviation	Common Name	
Ac-dd-Gal	2′-*O*-acetylabequose	A, *^a^* B, C, E, F, L, M, N, O
dd-Gal	abequose	B, C, D, *^a^* K
d-Gal	D-fucose	N, O
d-Gul	antiarose	L, M
AcO	acetoxy	E, G, H, I, *^a^* R
HO	hydroxyl	F, G, H, J, *^a^* Q

*^a^* The substituent appears twice in the tolyporphin.

**Table 14:** Comparison of protein sequence similarities [164,202].

*Oculatella* sp. LEGE 06141	HT-58-2 BGC-1 (% identity) *^a^*	HT-58-2 BGC-2 (% identity) *^a^*
HemF2	56	67
TolI	59	66
TolJ	57	72
TolC	52	53
HemA	56	57
HcaE	N/A	50
HcaE	N/A	51
TolH	55	66
TolD2	69	62
TolD1	66	60
FdtA	N/A	N/A
WecE	N/A	N/A
TolA	82	46
TolB	83	N/A
HemE	86	N/A
HemC	76	80
HemB	88	N/A
HemF1	78	41

*^a^* Versus that for *Oculatella* sp. LEGE 06141.

## Data Availability

All data described herein are contained herein or in the cited literature.

## Supplementary Materials

The following supporting information can be downloaded at: https://www.mdpi.com/article/10.3390/molecules28166132/s1, Discovery record of HT-58-2 (Figures S1–S3); tolyporphin A purity analysis (Figure S4); culture media formulations (Table S1); sources of strains sought for study (Tables S2 and S3); search for new tolyporphins producers (Table S4, Figures S5–S7); isotopic labeling studies (Table S5, Figures S8–S11), comprising 47 pages. References [219,220,221] are cited in the supplementary materials.
