# Supplementary material for: Tolyporphins–Exotic Tetrapyrrole Pigments in a Cyanobacterium—A Review
Source: Molecules. 2023 Aug 18;28(16):6132. doi: 10.3390/molecules28166132 (PMC10459692; doi:10.3390/molecules28166132)
Supplement: Supplementary file 1 [file molecules-28-06132-s001.zip › molecules-2529521-supplementary.pdf]

Supplementary Materials for:

# Tolyporphins–Exotic Tetrapyrrole Pigments in a Cyanobacterium—A Review

**Kathy-Uyen Nguyen [1], Yunlong Zhang [1], Qihui Liu [1], Ran Zhang [1], Xiaohe Jin [1], Masahiko Taniguchi [1], Eric S. Miller [2] and Jonathan S. Lindsey [1,*]**

[1] Department of Chemistry, North Carolina State University, Raleigh, NC 27695-8204, USA; uvnguyen@ncsu.edu (K.-U.N.); yzhang95@alumni.ncsu.edu (Y.Z.); qliu26@ncsu.edu (Q.L.); rzhang0915@gmail.com (R.Z.); xjin4@ncsu.edu (X.J.); mtanigu@ncsu.edu (M.T.)

[2] Department of Plant and Microbial Biology, North Carolina State University, Raleigh, NC 27695-7612, USA; esm@ncsu.edu

* Correspondence: jlindsey@ncsu.edu; Tel.: +1-919-515-6406

## Table of Contents

| Topic | Page |
|---|---|
| S1. The strain HT-58-2 (main text, section 1) | S1 |
| S2. Purity analysis of native tolyporphin A (main text, section 5) | S5 |
| S3. The culture media (main text, section 6) | S6 |
| S4. Search for tolyporphins in known strains (main text, section 14) | S7 |
| S5. Search for tolyporphins in strains newly collected in Hawai'i (main text, section 14) | S13 |
| S6. Isotopic labeling to probe tolyporphins biosynthesis (main text, section 15) | S43 |

## S1. The strain HT-58-2

The following information was graciously provided (via email, September 22, 2017) by Prof. Philip G. Williams at University of Hawai'i [54], and is presented here (lightly edited) for the historical record.

> 'Examination of old computer records revealed that HT-58-2 was collected on or around February 15, 1989. The uncertainty stems from the fact that other samples were stated to have been collected on the same date in the Chuuk Islands; both collections on the same day are unlikely from a logistical perspective. HT-58-2 was collected from a soil sample by Gregory Patterson at 6 50.33 N, 158 19.57 E, which is on the south side of the island of Nan Madol near the ocean. At some point some degree of sequencing was carried out, leading to the conclusion that the strain was likely a *Tolypothrix*. Figure S1 is a picture of parts of the old database record with some of the culture information.
>
> Two notebook entries from 1989 provide information concerning the first two large-scale grow ups (Figures S2 and S3). While a note suggests examination of BG media, it appears HT-58-2 was originally grown in A3M7 media, which is a modified Allen's Medium that is low in nitrate compared to BG-11, and also contains unique trace metals. A3M7 media, while more involved to make, was a common media of that era.'

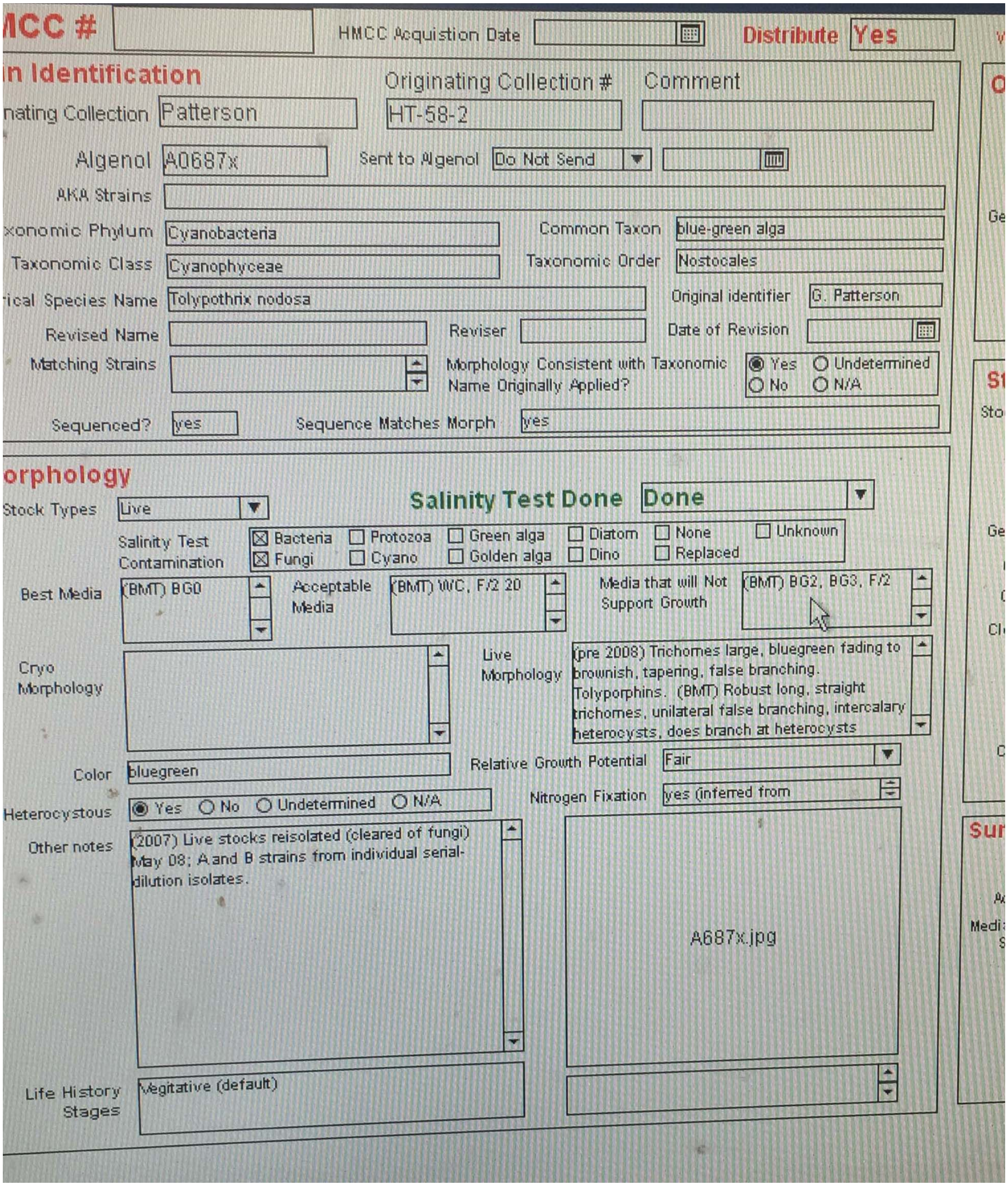


**Figure S1.** Discovery record.

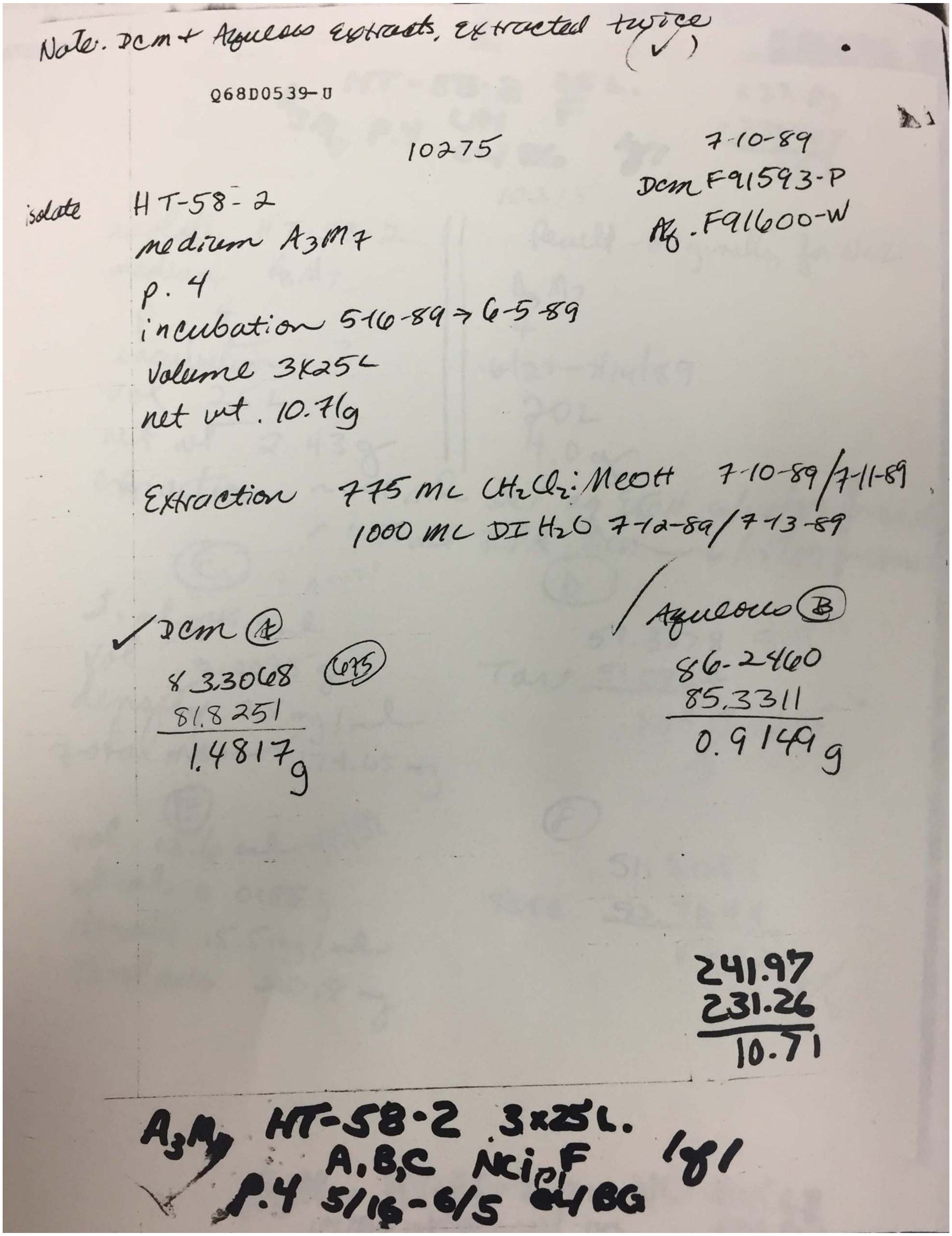

Note. DCM + Aqueous extracts, extracted twice (✓)

Q68D0539-U

10275

7-10-89
DCM F91593-P
Aq. F91600-W

isolate HT-58-2
medium $A_3M_7$
p. 4
incubation 5-16-89 → 6-5-89
Volume 3×25L
net wt. 10.71g

Extraction 775 mL $CH_2Cl_2$:MeOH 7-10-89/7-11-89
1000 mL DI $H_2O$ 7-12-89/7-13-89

✓ DCM (A)

83.3068 (675)
81.8251
1.4817 g

Aqueous (B)

86.2460
85.3311
0.9149 g

241.97
231.26
10.71

$A_3M_7$ HT-58-2 3×25L.
A, B, C NCI, F 181
P.4 5/16-6/5 [illegible] BG

**Figure S2.** Culture conditions – notebook page from July 10, 1989.

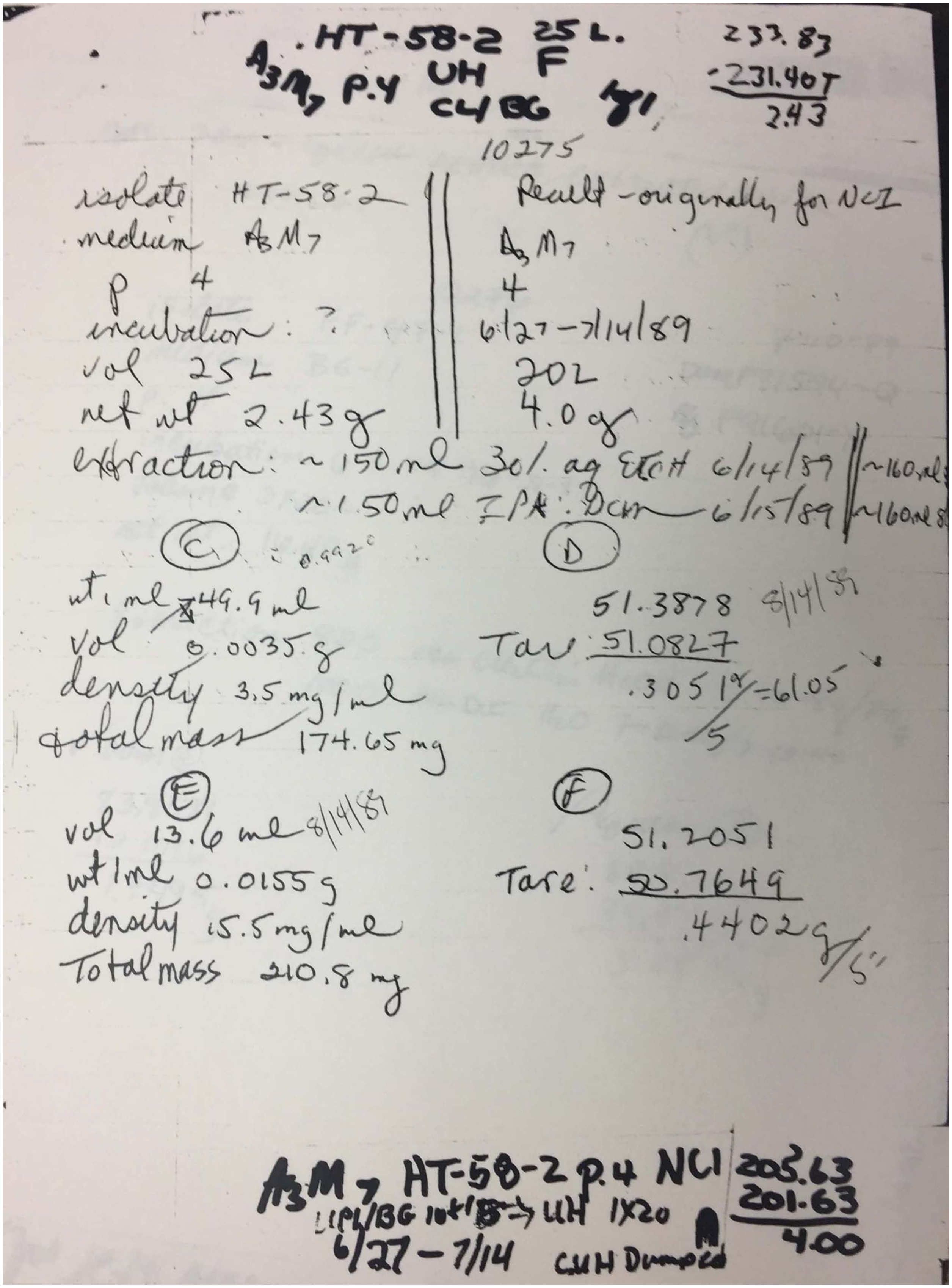
HT-58-2 25 L.
$A_3M_7$ P.4 UH F
C4/BG
233.83
-231.40T
2.43
10275

isolate HT-58-2 || Recult - originally for NCI
medium $A_3M_7$ || $A_3M_7$
p 4 || 4
incubation: ? || 6/27-7/14/89
vol 25L || 20L
net wt 2.43g || 4.0g
extraction: ~150ml 30% aq EtOH 6/14/89 || ~160ml
~150ml IPA: DCM 6/15/89 || ~160ml

(C)
wt/ml 49.9ml
vol 0.0035g
density 3.5 mg/ml
total mass 174.65 mg

(D)
51.3878 8/14/89
Tare: 51.0827
.3051 g = 61.05
/5

(E)
vol 13.6 ml 8/14/89
wt/ml 0.0155g
density 15.5mg/ml
Total mass 210.8 mg

(F)
51.2051
Tare: 50.7649
.4402g
/5

$A_3M_7$ HT-58-2 P.4 NCI
[illegible] UH 1X20
6/27-7/14 CUH Dumped
205.63
201.63
4.00

**Figure S3.** Culture conditions – June to July, 1989.

**S2. Purity analysis of native tolyporphin A**

The sample of tolyporphin A provided by the group of Prof. Philip Williams at University of Hawaiʻi was examined in the authors' lab by HPLC analysis with absorption spectral detection (401 nm, reference 360 nm). The chromatogram is shown in Figure S4. The purity of the sample is remarkably high. This high-purity sample was used for spectroscopic and photophysical analysis in a range of solvents [49].

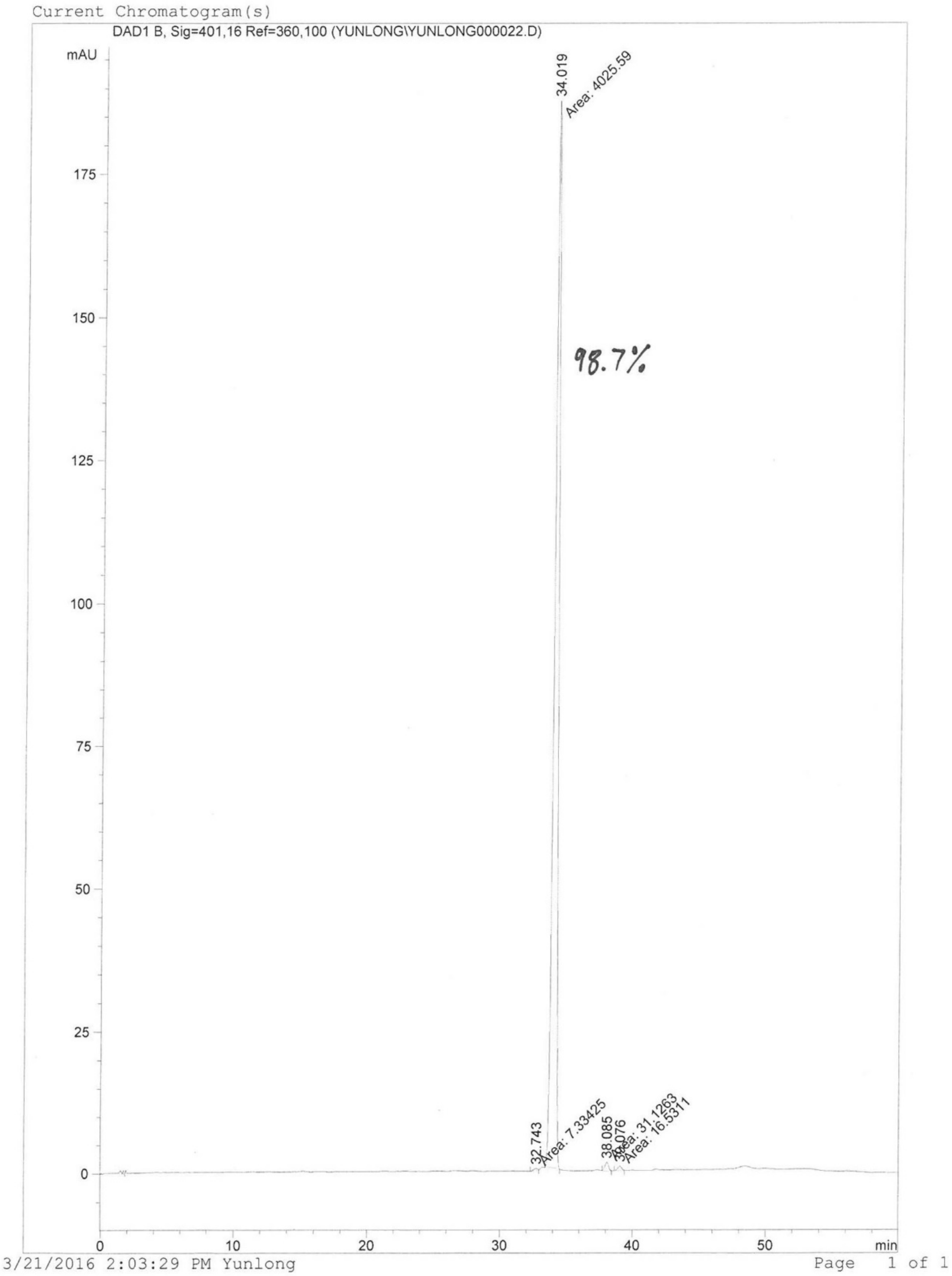


**Figure S4.** HPLC trace with absorption detection (401 nm) of a sample of tolyporphin A.

## S3. Culture media

**Table S1.** Composition of culture medium (Unit: mg/L)[a]

| | A3M7[b] | Allen and Arnon plus nitrate[c] | BG11[d] | $BG11_0$ |
|---|---|---|---|---|
| $NaNO_3$ | 200 | 212 | 1500 | ---- |
| $KNO_3$ | | 253 | | ---- |
| $NH_4Cl$ | 10 | | | ---- |
| $K_2HPO_4 \cdot 3H_2O$ | 50 | 350 | 40 | 40 |
| $MgSO_4 \cdot 7H_2O$ | 50 | 250 | 75 | 75 |
| $CaCl_2 \cdot 2H_2O$ | 13 | 75 | 36 | 36 |
| MOPS | 627 | | | |
| NaCl | | 238 | | |
| $Na_2CO_3$ | | | 20 | 20 |
| KOH | | 8.32 | | |
| $C_6H_8O_7 \cdot H_2O$ | | | 6 | 6 |
| $FeCl_3$ | 0.32 | | | |
| $FeSO_4 \cdot 7H_2O$ | | 21.9 | | |
| $(NH_4)_5[Fe(C_6H_4O_7)_2]$ | | | 6 | 6 |
| $Na_2EDTA$ | 3.0 | 32.6 | 1 | 1 |
| $H_3BO_3$ | 0.62 | 2.86 | 2.86 | 2.86 |
| $MnCl_2 \cdot 4H_2O$ | 1.4 | 1.8 | 1.81 | 1.81 |
| $ZnCl_2$ | 0.1 | | | |
| $ZnSO_4 \cdot 7H_2O$ | | 0.22 | 0.222 | 0.22 |
| $CoCl_2 \cdot 6H_2O$ | 0.005 | 0.040 | | |
| $Co(NO_3)_2 \cdot 6H_2O$ | | | 0.0494 | 0.0494 |
| $CuCl_2 \cdot 2H_2O$ | 0.000034 | | | |
| $CuSO_4 \cdot 5H_2O$ | | 0.079 | 0.079 | 0.079 |
| $MoO_3$ | 0.00249 | 0.18 | | |
| $NaMoO_4 \cdot 2H_2O$ | | | 0.39 | 0.39 |
| $NH_4VO_3$ | 0.002755 | 0.023 | | |
| $NiSO_4 \cdot H_2O$ | 0.005374 | | | |
| $Na_2WO_4 \cdot H_2O$ | 0.002153 | | | |
| $Al_2(SO_4)_3$ | 0.003805 | | | |
| $CdCl_2$ | 0.000978 | | | |
| $SrSO_4$ | 0.001259 | | | |
| $PbCl_2$ | 0.000805 | | | |
| LiCl | 0.003666 | | | |
| NaBr | 0.000773 | | | |
| KI | 0.000785 | | | |
| NaF | 0.001326 | | | |

[a]Entries shown in red highlight nitrogenous ingredients. [b]Ref [19]. [c]Ref [219]. [d]Ref [114].

## S4. Search for tolyporphins in known strains

The strains procured from commercial or private collections are listed in Table S2. Altogether 62 strains were obtained. In general, the strains were cultured in BG-11 or BG-$11_0$. In some cases growth was slow; then on contact with the originating scientists, AA media was recommended. When BG-$11_0$ is shown, BG-11 also was examined. In each case, the medium that afforded better growth was used for studies.

Extracts were prepared and analyzed. The culture media and the analytical results are listed in Table S3. The cases that gave a single positive result (entries 24, 26, 30, 46, 48, and 49) did not afford confirmatory positive results upon subsequent examination, for example, by MS/MS analysis. Such results at present are regarded as false positives.

**Table S2. Sources of strains sought for study**

| Entry | Source | Strain | Received | Origin |
|---|---|---|---|---|
| 1 | UTEX | UTEX 2349 = PCC 9009 = *Scytonema hofmanni / Tolypothrix* | 06/30/15 | Watkins Glen State Park, NY (rocks, gorge trail) |
| 2 | Pasteur Culture Collection | PCC 7126 (*Microchaete, Scytonema*) | 07/01/15 | UC Berkeley Botany Department (freshwater aquarium) |
| 3 | UTEX | UTEX B379 = PCC 7708 = *Tolypothrix sp.* | 07/07/15 | India (soil sample) |
| 4 | UTEX | UTEX B424 = *Tolypothrix distorta* | 07/07/15 | Utrecht (flowerpot) |
| 5 | UTEX | UTEX B481 = PCC 7710 = *Tolypothrix sp. or Calothirx* | 07/07/15 | New Haven, CT (freshwater) |
| 6 | UTEX | UTEX B2963 = *Scytonematopsis contorta* | 07/07/15 | Oahu, Hawaii (rocks in waterfall) |
| 7 | UTEX | UTEX B2964 = *Scytonematopsis contorta* | 07/07/15 | Oahu, Hawaii (rocks in waterfall) |
| 8 | Alison Sherwood | AS01 = *Brasilonema sp.* | 07/21/15 | From a wooden fence on Hipawai St Honolulu, Hawaii |
| 9 | Arun Kumar Mishra | *Tolypothrix nodosa Ind17* | 08/02/15 | India |
| 10 | Arun Kumar Mishra | *Scytonema bohnerii Ind25* | 08/02/15 | India |
| 11 | Sucheta Tripathy | *Tolypothrix boutellei* VB521301 | 08/18/15 | India |
| 12 | Philip Williams | *Nostocaceae* HT-58-2 | 10/28/15 | Soil sample, Nan Madol, Micronesia |
| 13 | Philip Williams | *Scytonema sp.* DB 1-1 | 10/28/15 | Chuuk |
| 14 | National Center for Marine Algae and Microbiota | CCMP 1185 = *Tolypothrix sp.* | 04/14/16 | Freshwater and warm water |
| 15 | Pasteur Culture Collection | PCC 7423 = *Nostoc sp.* | 6/29/16 | Senegal, Soil dry samples |
| 16 | Pasteur Culture Collection | PCC 7902 = *Synechococcus sp.* | 6/29/16 | Polar bear, hair, San Diego Zoo, CA, USA. |
| 17 | Madaiah Rajashekhar | *Scytonema bohnerii* | 9/23/15 | Sulfur spring, Western Ghats of Karnataka, India |
| 18 | Culture Collection of Algae U. Göttingen, Germany | PCC 7120 = UTEX 2576 = SAG 25.82 = *Anabaena sp.* | 5/15/16 | Bot. Gard. Univ. Göttingen, bench with nutrient solution in greenhouse |
| 19 | A. Sherwood, G. Presting | AS02 = *Brasilonema sp.* | 6/14/16 | Moleka trail, Honolulu, Hawaii |
| 20 | A. Sherwood, G. Presting | AS03 = *Brasilonema sp.* | 07/18/17 | Moleka trail, Honolulu, Hawaii |
| 21 | Gustavo Montejano Zurita and Itzel Becerra Absalón | UNAM-01 = *B. tolantongesis* | 07/19/17 | National Autonomous University of Mexico |
| 22 | Gustavo Montejano Zurita | UNAM-02 = *B. roberti-lamii* | 07/19/17 | National Autonomous University of Mexico |
| 23 | Francisco Barona Gómez | Cycas 32_K59 = *Nostoc sp.* | 09/11/17 | LANGEBIO |

| | | | | |
|---|---|---|---|---|
| 24 | Francisco Barona Gómez | JP6 106C = *Nostoc sp.* | 09/11/17 | LANGEBIO |
| 25 | Francisco Barona Gómez | RF1 4 114 = *Nostoc sp.* | 09/11/17 | LANGEBIO |
| 26 | Francisco Barona Gómez | JP2 5 105 = *Nostoc sp.* | 09/11/17 | LANGEBIO |
| 27 | Francisco Barona Gómez | RF1 5 115 = *Nostoc sp.* | 09/11/17 | LANGEBIO |
| 28 | Francisco Barona Gómez | Edule_3salida = *D. edule* | 09/11/17 | LANGEBIO |
| 29 | Francisco Barona Gómez | DED5J7A = *Nostoc sp.* | 09/11/17 | LANGEBIO |
| 30 | Francisco Barona Gómez | Edule 31_K59 = *Nostoc sp.* | 09/11/17 | LANGEBIO |
| 31 | Francisco Barona Gómez | Edule 35K_25 = *Nostoc sp.* | 09/11/17 | LANGEBIO |
| 32 | Francisco Barona Gómez | DED5J7B = *D. edule* | 09/11/17 | LANGEBIO |
| 33 | Francisco Barona Gómez | Edule 33_K59 = *Nostoc sp.* | 09/11/17 | LANGEBIO |
| 34 | Francisco Barona Gómez | DCO5J3D = *Nostoc sp.* | 09/11/17 | LANGEBIO |
| 35 | Francisco Barona Gómez | Cycas 32_K59 = *Nostoc sp.* | 09/11/17 | LANGEBIO |
| 36 | Marcelo Vaz, Diego Genuário | CMM-UFV E1 = *Brasilonema sp.* | 09/27/17 | Universidade de São Paulo |
| 37 | Marcelo Vaz, Diego Genuário | CMM-UFV E2 = *Brasilonema sp.* | 09/27/17 | Universidade de São Paulo |
| 38 | Marcelo Vaz, Diego Genuário | CMM-UFV 046 = *Brasilonema sp.* | 09/27/17 | Universidade de São Paulo |
| 39 | Marcelo Vaz, Diego Genuário | BR04 = *Brasilonema sp.* | 09/27/17 | Universidade de São Paulo |
| 40 | Marcelo Vaz, Diego Genuário | BR11 = *Brasilonema sp.* | 09/27/17 | Universidade de São Paulo |
| 41 | Marcelo Vaz, Diego Genuário | BR12 = *Brasilonema sp.* | 09/27/17 | Universidade de São Paulo |
| 42 | Marcelo Vaz, Diego Genuário | BR14 = *Brasilonema sp.* | 09/27/17 | Universidade de São Paulo |
| 43 | Marcelo Vaz, Diego Genuário | BR19 = *Brasilonema sp.* | 09/27/17 | Universidade de São Paulo |
| 44 | Marcelo Vaz, Diego Genuário | BR32 = *Brasilonema sp.* | 09/27/17 | Universidade de São Paulo |
| 45 | Marcelo Vaz, Diego Genuário | BR50 = *Brasilonema sp.* | 09/27/17 | Universidade de São Paulo |
| 46 | Marcelo Vaz, Diego Genuário | BR54 = *Brasilonema sp.* | 09/27/17 | Universidade de São Paulo |
| 47 | Marcelo Vaz, Diego Genuário | BR56 = *Brasilonema sp.* | 09/27/17 | Universidade de São Paulo |
| 48 | Marcelo Vaz, Diego Genuário | BR57 = *Brasilonema sp.* | 09/27/17 | Universidade de São Paulo |
| 49 | Marcelo Vaz, Diego Genuário | BR58 = *Brasilonema sp.* | 09/27/17 | Universidade de São Paulo |
| 50 | Marcelo Vaz, Diego Genuário | BR71 = *Brasilonema sp.* | 09/27/17 | Universidade de São Paulo |
| 51 | Marcelo Vaz, Diego Genuário | BR95 = *Brasilonema sp.* | 09/27/17 | Universidade de São Paulo |
| 52 | Marcelo Vaz, Diego Genuário | BR98 = *Brasilonema sp.* | 09/27/17 | Universidade de São Paulo |
| 53 | Marcelo Vaz, Diego Genuário | BR101 = *Brasilonema sp.* | 09/27/17 | Universidade de São Paulo |

| | | | | |
|---|---|---|---|---|
| 54 | Marcelo Vaz, Diego Genuário | BR102 = *Brasilonema sp.* | 09/27/17 | Universidade de São Paulo |
| 55 | Marcelo Vaz, Diego Genuário | BR106 = *Brasilonema sp.* | 09/27/17 | Universidade de São Paulo |
| 56 | Pasteur Culture Collection | PCC73104 = *Nodularia spumigena* | 12/13/17 | Ioslated from a toxic bloom in the Baltic sea |
| 57 | NIES | NIES-2098 = *Calothrix sp.* | 01/14/18 | National Institute for Environmental Studies, Japan |
| 58 | NIES | NIES-2100 = *Calothrix sp.* | 01/14/18 | National Institute for Environmental Studies, Japan |
| 59 | NIES | NIES-3585 = *Nodularia sp.* | 01/14/18 | National Institute for Environmental Studies, Japan |
| 60 | NIES | NIES-4103 = *Nostoc sp.* | 01/14/18 | National Institute for Environmental Studies, Japan |
| 61 | Xuefeng Lu | HK-05 = NIES-2130 = *Scytonema sp.* | 01/16/18 | Qingdao Institute of Bioenergy and Bioprocess Technology (QIBEBT) |
| 62 | Xuefeng Lu | HK-06 = NIES-2101 = *Scytonema sp.* | 01/16/18 | Qingdao Institute of Bioenergy and Bioprocess Technology (QIBEBT) |

**Table S3. Results upon study of strains**

| Entry | Strain | Media | ESI-MS | MALDI-MS | IR-MALDESI | Absorption |
|---|---|---|---|---|---|---|
| 1 | UTEX 2349 = PCC 9009 = *Scytonema hofmanni / Tolypothrix* | BG-11 | Negative | Negative | Negative | Negative |
| 2 | PCC 7126 (*Microchaete, Scytonema*) | BG-11 | Negative | Negative | Negative | Negative |
| 3 | UTEX B379 = PCC 7708 = *Tolypothrix sp.* | BG-11 | Negative | Negative | Negative | Negative |
| 4 | UTEX B424 = *Tolypothrix distorta* | BG-11 | Negative | Negative | Negative | Negative |
| 5 | UTEX B481 = PCC 7710 = *Tolypothrix sp. or Calothirx* | BG-11 | Negative | Negative | Negative | Negative |
| 6 | UTEX B2963 = *Scytonematopsis contorta* | BG-11 | Negative | Negative | Negative | Negative |
| 7 | UTEX B2964 = *Scytonematopsis contorta* | BG-11 | Negative | Negative | Negative | Negative |
| 8 | AS01 = *Brasilonema sp.* | BG-11 | Negative | Negative | Negative | Negative |
| 9 | *Tolypothrix nodosa Ind17* | BG-11 | Negative | Negative | Negative | Negative |
| 10 | *Scytonema bohnerii Ind25* | BG-11 | Negative | Negative | Negative | Negative |
| 11 | *Tolypothrix boutellei* VB521301 | BG-11 | Negative | Negative | Negative | Negative |
| 12 | *Nostocaceae* HT-58-2 | BG-11$_0$ | Positive | Positive | Positive | Positive |
| 13 | *Scytonema sp.* DB 1-1 | BG-11 | Negative | Negative | Negative | Negative |
| 14 | CCMP 1185 = *Tolypothrix sp.* | BG-11 | Negative | Negative | Negative | Negative |
| 15 | PCC 7423 = *Nostoc sp.* | BG-11 | Negative | Negative | Negative | Negative |

| | | | | | | |
|---|---|---|---|---|---|---|
| 16 | PCC 7902 = *Synechococcus sp.* | BG-11 | Negative | Negative | Negative | Negative |
| 17 | *Scytonema bohnerii* | BG-11 | Negative | Negative | Negative | Negative |
| 18 | PCC 7120 = UTEX 2576 = SAG 25.82 = *Anabaena sp.* | BG-11 | Negative | Negative | Negative | Negative |
| 19 | AS02 = *Brasilonema sp.* | BG-11 | Negative | Negative | Negative | Negative |
| 20 | AS03 = *Brasilonema sp.* | BG-11 | Negative | Negative | Negative | Negative |
| 21 | UNAM-01 = *B. tolantongesis* | BG-11 | Negative | Negative | Negative | Negative |
| 22 | UNAM-02 = *B. roberti-lamii* | BG-11 | Negative | Negative | Negative | Negative |
| 23 | Cycas 32_K59 = *Nostoc sp.* | BG-11 | Negative | Negative | Negative | Negative |
| 24 | JP6 106C = *Nostoc sp.* | BG-$11_0$ | Positive | Negative | Negative | Negative |
| 25 | RF1 4 114 = *Nostoc sp.* | BG-11 | Negative | Negative | Negative | Negative |
| 26 | JP2 5 105 = *Nostoc sp.* | BG-11 | Negative | Negative | Positive | Negative |
| 27 | RF1 5 115 = *Nostoc sp.* | BG-11 | Negative | Negative | Negative | Negative |
| 28 | Edule_3salida = *D. edule* | BG-11 | Negative | Negative | Negative | Negative |
| 29 | DED5J7A = *Nostoc sp.* | BG-11 | Negative | Negative | Negative | Negative |
| 30 | Edule 31_K59 = *Nostoc sp.* | BG-11 | Positive | Negative | Negative | Negative |
| 31 | Edule 35K_25 = *Nostoc sp.* | BG-11 | Negative | Negative | Negative | Negative |
| 32 | DED5J7B = *D. edule* | BG-11 | Negative | Negative | Negative | Negative |
| 33 | Edule 33_K59 = *Nostoc sp.* | BG-11 | Negative | Negative | Negative | Negative |
| 34 | DCO5J3D = *Nostoc sp.* | BG-11 | Negative | Negative | Negative | Negative |
| 35 | Cycas 32_K59 = *Nostoc sp.* | BG-$11_0$ | Negative | Negative | Negative | Negative |
| 36 | CMM-UFV E1 = *Brasilonema sp.* | BG-$11_0$ | Negative | Negative | Negative | Negative |
| 37 | CMM-UFV E2 = *Brasilonema sp.* | BG-$11_0$ | Negative | Negative | Negative | Negative |
| 38 | CMM-UFV 046 = *Brasilonema sp.* | BG-$11_0$ | Negative | Negative | Negative | Negative |
| 39 | BR04 = *Brasilonema sp.* | BG-$11_0$ | Negative | Negative | Negative | Negative |
| 40 | BR11 = *Brasilonema sp.* | BG-$11_0$ | Negative | Negative | Negative | Negative |
| 41 | BR12 = *Brasilonema sp.* | BG-$11_0$ | Negative | Negative | Negative | Negative |
| 42 | BR14 = *Brasilonema sp.* | AA | Negative | Negative | Negative | Negative |
| 43 | BR19 = *Brasilonema sp.* | BG-$11_0$ | Negative | Negative | Negative | Negative |

| | | | | | | |
|---|---|---|---|---|---|---|
| 44 | BR32 = *Brasilonema sp.* | BG-$11_0$ | Negative | Negative | Negative | Negative |
| 45 | BR50 = *Brasilonema sp.* | BG-$11_0$ | Negative | Negative | Negative | Negative |
| 46 | BR54 = *Brasilonema sp.* | AA | Negative | Negative | Positive | Negative |
| 47 | BR56 = *Brasilonema sp.* | AA | Negative | Negative | Negative | Negative |
| 48 | BR57 = *Brasilonema sp.* | AA | Negative | Negative | Positive | Negative |
| 49 | BR58 = *Brasilonema sp.* | AA | Negative | Negative | Positive | Negative |
| 50 | BR71 = *Brasilonema sp.* | AA | Negative | Negative | Negative | Negative |
| 51 | BR95 = *Brasilonema sp.* | BG-$11_0$ | Negative | Negative | Negative | Negative |
| 52 | BR98 = *Brasilonema sp.* | BG-$11_0$ | Negative | Negative | Negative | Negative |
| 53 | BR101 = *Brasilonema sp.* | AA | Negative | Negative | Negative | Negative |
| 54 | BR102 = *Brasilonema sp.* | AA | Negative | Negative | Negative | Negative |
| 55 | BR106 = *Brasilonema sp.* | AA | Negative | Negative | Negative | Negative |
| 56 | PCC73104 = *Nodularia spumigena* | BG-11 | Negative | Negative | Negative | Negative |
| 57 | NIES-2098 = *Calothrix sp.* | BG-11 | Negative | Negative | Negative | Negative |
| 58 | NIES-2100 = *Calothrix sp.* | BG-11 | Negative | Negative | Negative | Negative |
| 59 | NIES-3585 = *Nodularia sp.* | BG-11 | Negative | Negative | Negative | Negative |
| 60 | NIES-4103 = *Nostoc sp.* | BG-11 | Negative | Negative | Negative | Negative |
| 61 | HK-05 = NIES-2130 = *Scytonema sp.* | BG-11 | Negative | Negative | Negative | Negative |
| 62 | HK-06 = NIES-2101 = *Scytonema sp.* | BG-11 | Negative | Negative | Negative | Negative |

### S5. Search for tolyporphins in strains newly collected in Hawaiʻi

One of us (JSL) collected 202 specimens of putative cyanobacteria on the island of Hawaiʻi. Samples were returned by express mail to North Carolina State University for attempted culturing and analysis. Of the 202 samples, 139 afforded green cultures; of those, 101 gave sufficient quantity for analysis. The general regions for collections are listed in Table S4. The names follow Hawaiian spelling [220].

**Table S4. Hawaii samples**

| Region | Sample numbers | Total | Grew | Tested |
|---|---|---|---|---|
| Kailua | 39–55, 79–84 | 23 | 19 | 14 |
| Hōnaunau | 19–29, 35–38, 176–200 | 40 | 23 | 17 |
| Hoʻōpūloa | 30 | 1 | 1 | 1 |
| Honuʻapo | 31–34 | 4 | 2 | 2 |
| Makuu | 133–143 | 11 | 10 | 7 |
| Hilo | 6–9, 118–130, 144–147 | 21 | 17 | 10 |
| Humuʻula | 152–159 | 8 | 2 | 2 |
| Honomū | 1–5, 10, 85–117, 131–132, 148–151, 175 | 46 | 33 | 19 |
| Hāmākua | 58–64 | 7 | 6 | 6 |
| Waipiʻo | 11–13, 56, 57, 160–174, 201, 202 | 22 | 11 | 8 |
| Kohala | 14–18, 65–78 | 19 | 15 | 15 |
| **Total** | | 202 | 139 | 101 |

The organization of regions in the table proceeds counterclockwise around the island of Hawaiʻi through the regions as shown in Figure S5. Samples 1–52 were collected in the period July 29 – August 5, 2017. Samples 53–200 were collected in the period May 14 – May 25, 2019. Samples 201 and 202 were collected on December 4, 2022. Sectors of the island where no samples were collected include Kalapana, Puna, Kilauea, Pāhala, Ka Lae, Mauno Loa, Kaʻohe and Puakō. With the exception of Kalapana, the landmass in each such sector is largely very dry and hence not particularly conducive to growth of cyanobacteria. A more expansive description of the culture collection will be published elsewhere.

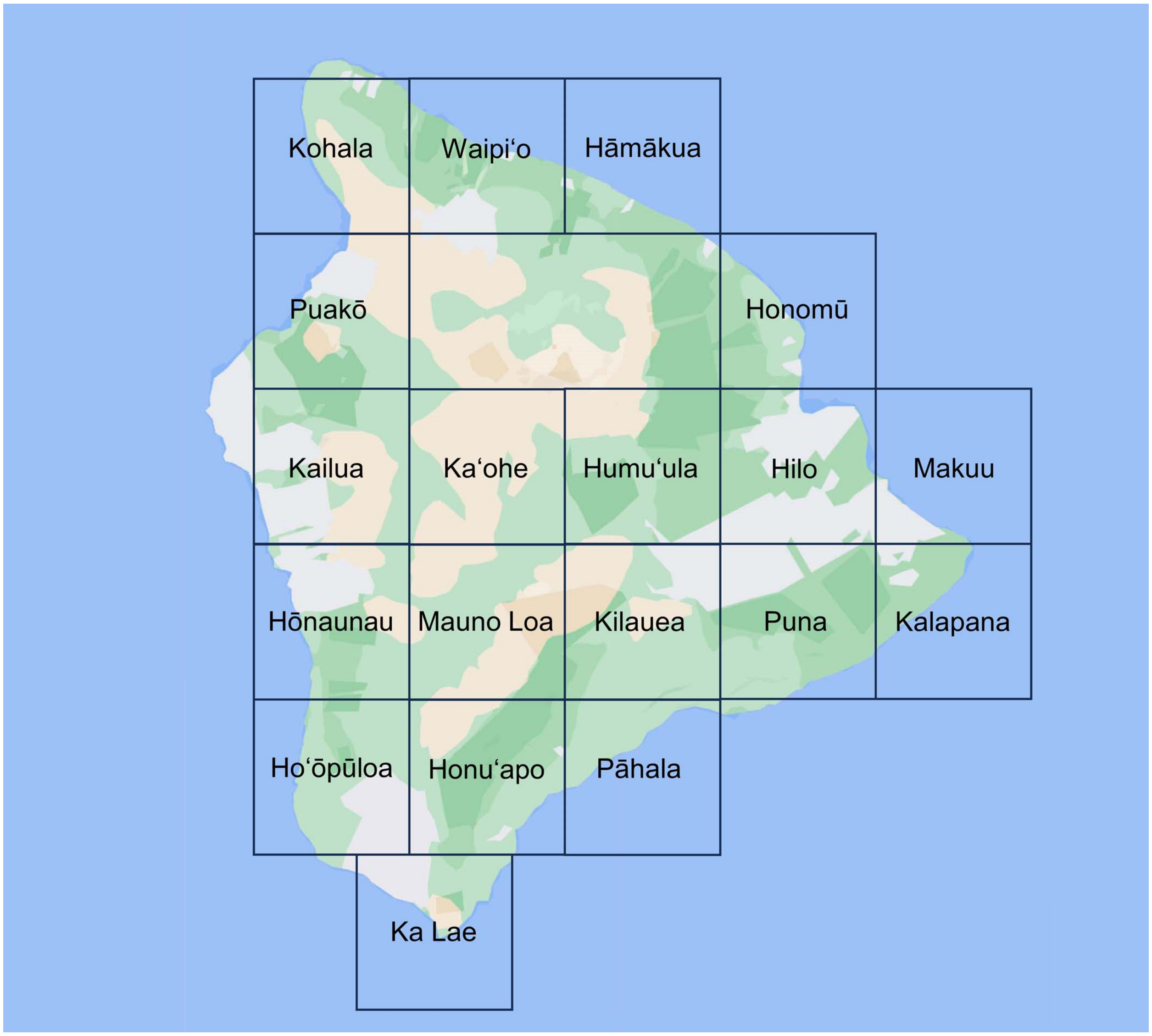


**Figure S5.** Regions of Hawaii.

To facilitate analysis, we developed an expedited procedure to generate a lipophilic extract (***LE1*****). A key feature of the expedited procedure concerned the method of homogenization. The prior method employed a high-speed microblade for disrupting the filamentous cyanobacteria (0.5 –100 mL samples), which was very effective but could only be used serially, and required extensive cleaning between samples to preclude contamination [133,158]. The new method employed zirconium beads (0.1 mm diameter) inside small tubes (≤ 2 mL) for cellular disruption, which is amenable to implementation of multiple small-scale samples in parallel. A set of 4-6 samples (2 mL each) was easily prepared per hour, which could yield 20–30 lipophilic extracts each day. Further increases in sample throughput may be achievable. The lipophilic extracts were then examined by fluorescence excitation analysis, which required 1 hour per sample to obtain publication-quality spectra. In practice, a set of 10 extracts can be analyzed within one day. The flowchart for sample handling is shown in Figure S6. The procedures are described in detail in the following experimental section.

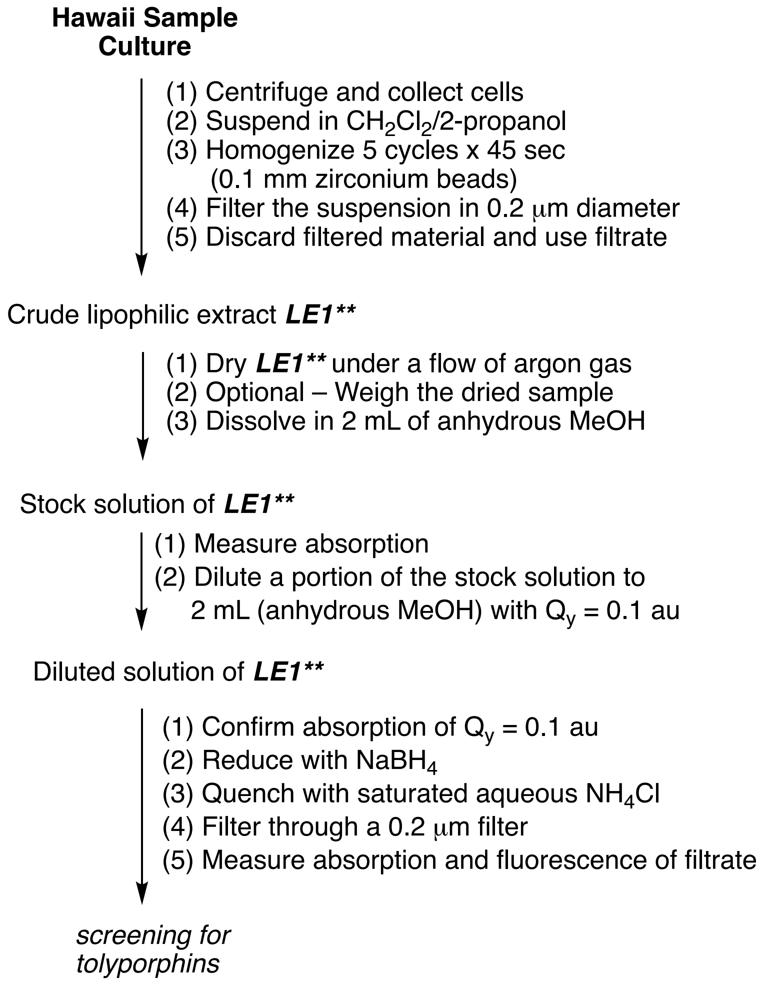


**Figure S6.** Flowchart for expedited screening of cultured field samples for tolyporphins.

**Protocols for rapid screening of cultures.** *(i) Expedited extraction.* Hawaii sample cultures (~30 mL) were grown in BG-11 in 50 mL tissue flasks. Each culture was filamentous. From a 30-mL cultivated culture, 2 mL was taken (including filamentous material) and centrifuged at 14,000 rpm for 5 min. The supernatant was decanted. The resulting clumps of the culture cells were then suspended in 1.8 mL of $CH_2Cl_2$/2-propanol (v/v = 1:1). The suspension was transferred into a pre-assembled 2-mL zirconium bead kit (Benchmark Scientific D1032-01, zirconium beads = 0.1 mm diameter). Using a BeadBug 6 instrument (Benchmark Scientific D1036 BeadBug 6, Microtube Homogenizer) at 3000 rpm, the sample was subjected to 5 cycles of 45 seconds of homogenizing followed by 60 seconds of rest (1 cycle = 105 seconds total). The resulting homogenized culture was filtered through 0.2 μm diameter filter to remove the zirconium beads and unwanted residue. The filtrate containing extracted pigments (termed ***LE1*****) typically constituted ~1.8 mL. The extract was stored in a 3-mL glass vial at 4 °C until it could be dried under a stream of argon for the fluorescence assay (≥ 2 days). On average, the procedure required 15–60 min for one sample, but 6 samples could be treated in parallel.

*(ii) Fluorescence excitation analysis.* The fluorescence analysis was carried out as described previously [158], and is presented here for completeness given the scale and number of

samples. For fluorescence analysis of ***LE1*****, the entire 1.8 mL sample was taken to dryness under a stream of argon. The resulting residue (~10 mg total) was dissolved in its entirety in 2 mL of anhydrous MeOH. The 2-mL sample was examined by absorption spectroscopy at room temperature; a sample therefrom was diluted if needed to attain a $Q_y$ band absorbance of ~0.1 au. Non-diluted material, when available, was dried under argon and set aside as a storage sample. The diluted sample (2 mL with $Q_y$ = 0.1 au) was transferred to a 3-mL glass vial. Then, $NaBH_4$ (30 mg, ACS reagent grade) in solid form was added all at once. The vial was loosely capped, and the mixture was left to react at room temperature away from direct light. After 30 min, 2 drops (~20 µL total) of saturated aqueous $NH_4Cl$ were added to quench the reaction. The cloudy solution was filtered through a Whatman syringe filter (PTFE Puradisc, 0.2 micron). The vial walls were rinsed with 400 µL of methanol and passed through the same filter. The filtrate was allowed to flow directly into an empty 1-cm pathlength, quartz cuvette (this cuvette was previously filled with methanol to establish a background for absorption spectroscopy then emptied). The reduced mixture in the cuvette was examined by absorption spectroscopy followed by fluorescence excitation spectroscopy; both at room temperature with prolonged signal acquisition times. Samples post workup were either measured on the same day or stored at –20 °C (sealed) and analyzed the next morning.

The prolonged signal acquisition times are as follows: absorption spectroscopy (Agilent 8453, diode-array detection), range = 300–900 nm; integration time = 8 s and spectral increments = 1 nm. Fluorescence excitation spectroscopy (PTI with R928 photomultiplier), $\lambda_{em}$ = 710 nm; bandpass = 2 nm (0.375 mm slit width); range = 360–550 nm; integration time = 8 s and and spectral increments = 1 nm.

Note: $NaBH_4$ is prone to deliquesce (depending on the daily humidity index), but this should not affect reduction efficiency as long as the deliquescent solid is added into the vial immediately after weighing.

**Comparison of extraction protocols.** Modifications to the prior extraction procedure [158] allowed rapid preparation of ***LE1***** samples for analysis by fluorescence excitation spectroscopy. The transition from using an IKA T10 homogenizer to a BeadBug6 homogenizer have made the extraction protocol faster and more effective and minimized cross-contamination across different samples. The advantages are as follows:

(i) Using the BeadBug6 homogenizer, 15–60 minutes was required to simultaneously extract pigments from 6 samples. For clumpy samples, additional time might be needed for sufficient extraction. Using the IKA T10 homogenizer with a S10N-8G-ST blade attachment, the pigment extraction process required at least 30 minutes for one sample (6 samples = 3 hours total).

(ii) Each sample was designated a single 0.1 mm Zirconium Bead Kit, and after 5 cycles of homogenization, said kit was immediately discarded. The use of individual kits automatically reduced cross-contamination between samples. When we used the IKA T10 homogenizer for pigment extraction, all samples were processed by the same blade. The blade attachment then needed to be carefully cleaned between each extraction to minimize cross-contamination.

Although the expedited protocol offers better productivity (multiple samples being prepared serially), using a BeadBug6 homogenizer does impose limits on the extraction sample size and thoroughness of pigment extraction in comparison to the prior protocol with an IKA T10 homogenizer [158]. The disadvantages are as follows:

(iii) The maximum capacity limit of each Bead Kit is 2 mL, so the BeadBug6 can only be used to extract pigments from samples containing <2 mL. With an IKA T10 homogenizer, samples ranging from 0.5 to 100 mL can be processed.

(iv) BeadBug6 has a smaller speed range (2,500–4,350 rpm) than the IKA T10 (8,000–30,000 rpm). Because of this, the extent of pigments extracted from samples vary between the two instruments. The former should be used for rapid screening of pigments, and the latter for quantitative applications and exhaustive pigment collection.

**Results upon reduction and fluorescence excitation analysis.** The results obtained upon analysis of each of the 101 cultures are shown in Figure S7. All spectra were collected of samples in methanol at room temperature. At left, the absorption spectrum prior to reduction is shown in a black solid line. The absorption spectrum following reduction is overlaid in a red dashed line. At right, the fluorescence excitation spectrum ($\lambda_{em}$ = 710 nm) of the sample after reduction is shown in a red solid line. In no case was the telltale signature of a dioxobacteriochlorin-type bacteriochlorin observed.

**Figure S7.** Left panels: Absorption spectra before (black solid line) and after reduction (red dashed line). Right panels: fluorescence excitation spectrum ($\lambda_{em}$ = 710 nm) after reduction (red solid line). All spectra are normalized. The panels continue on the following pages.

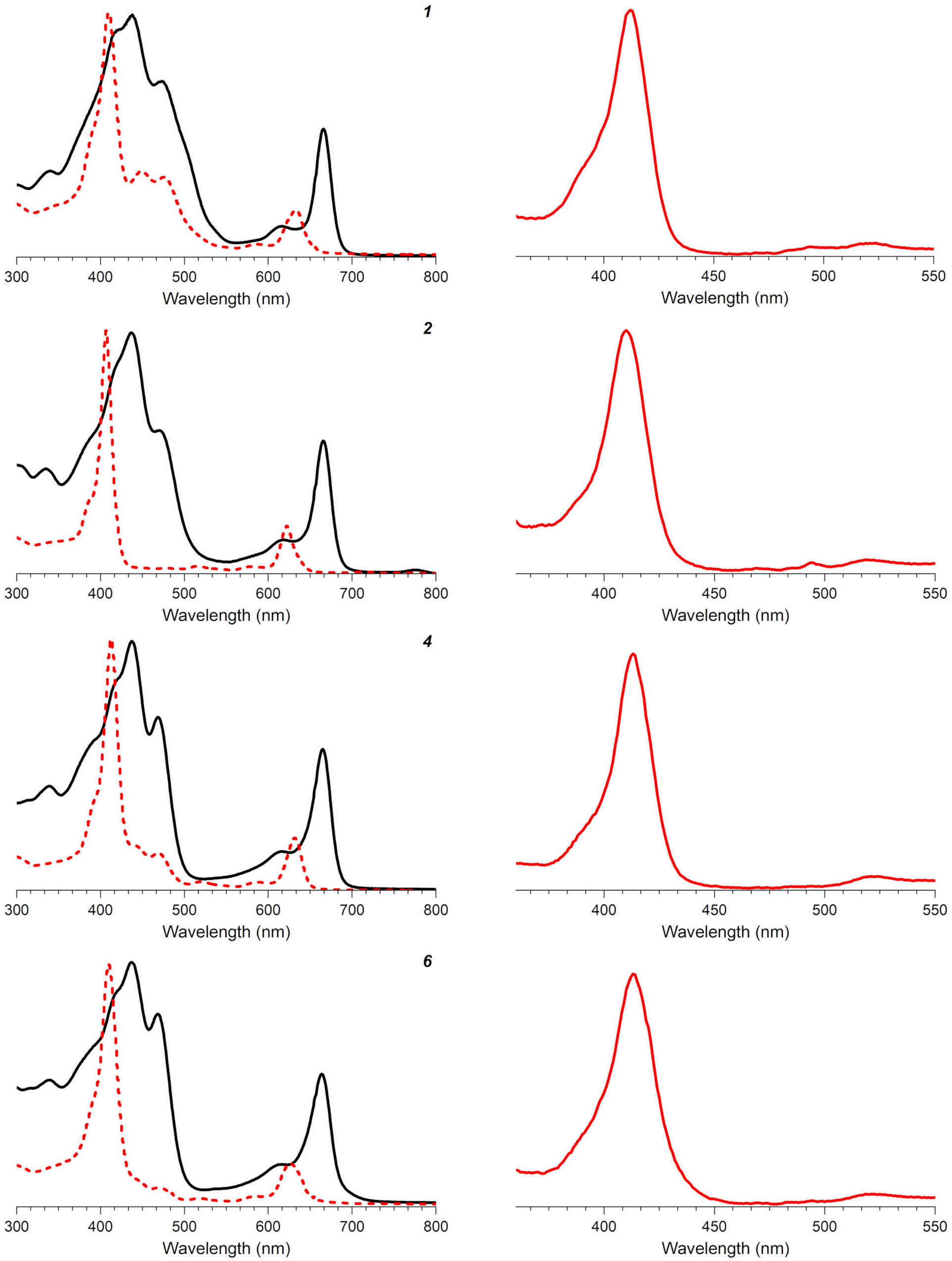

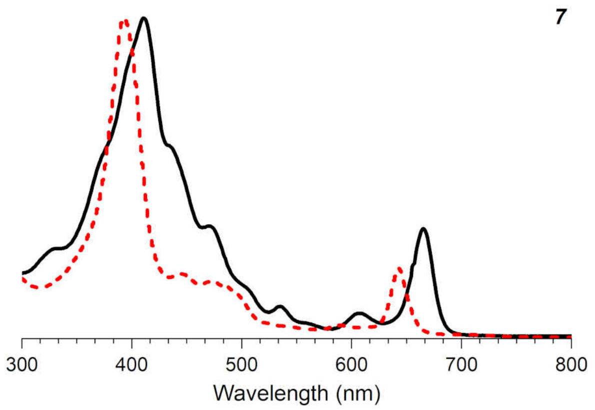
7
300
400
500
600
700
800
Wavelength (nm)
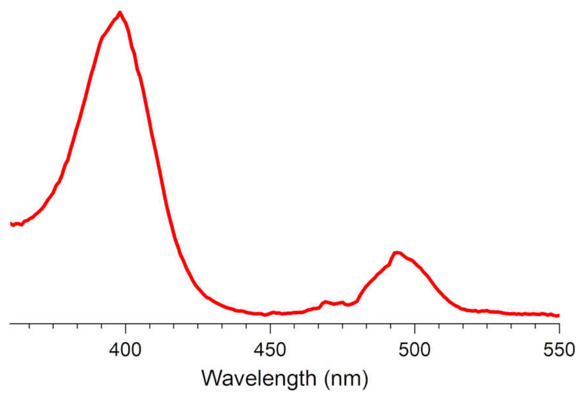
400
450
500
550
Wavelength (nm)
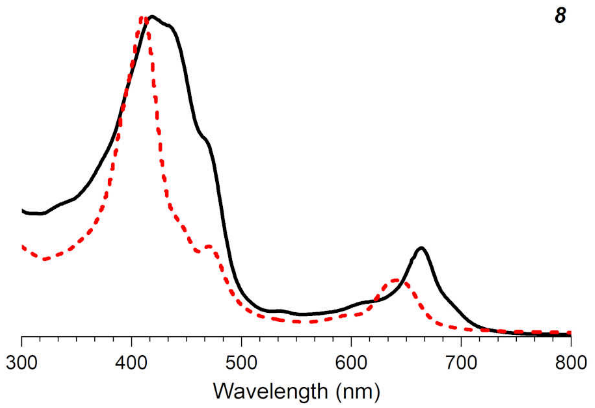
8
300
400
500
600
700
800
Wavelength (nm)
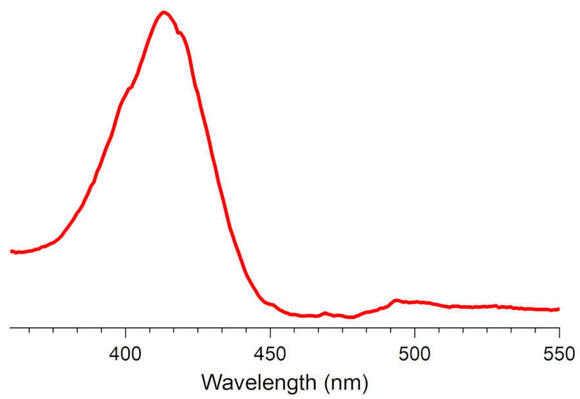
400
450
500
550
Wavelength (nm)
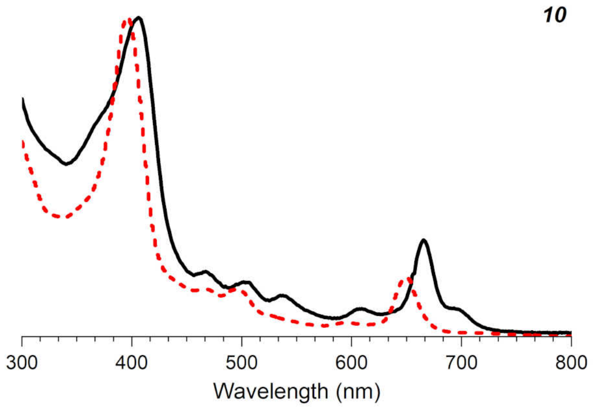
10
300
400
500
600
700
800
Wavelength (nm)
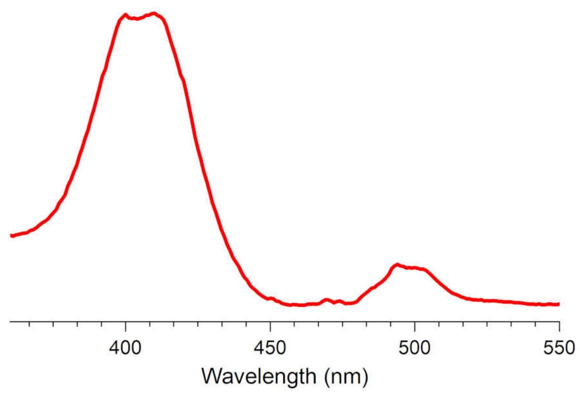
400
450
500
550
Wavelength (nm)
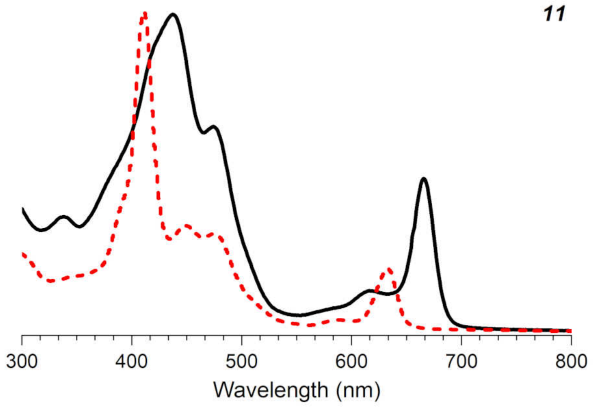
11
300
400
500
600
700
800
Wavelength (nm)
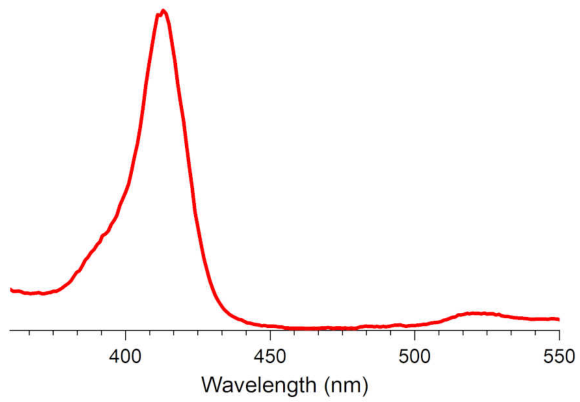
400
450
500
550
Wavelength (nm)

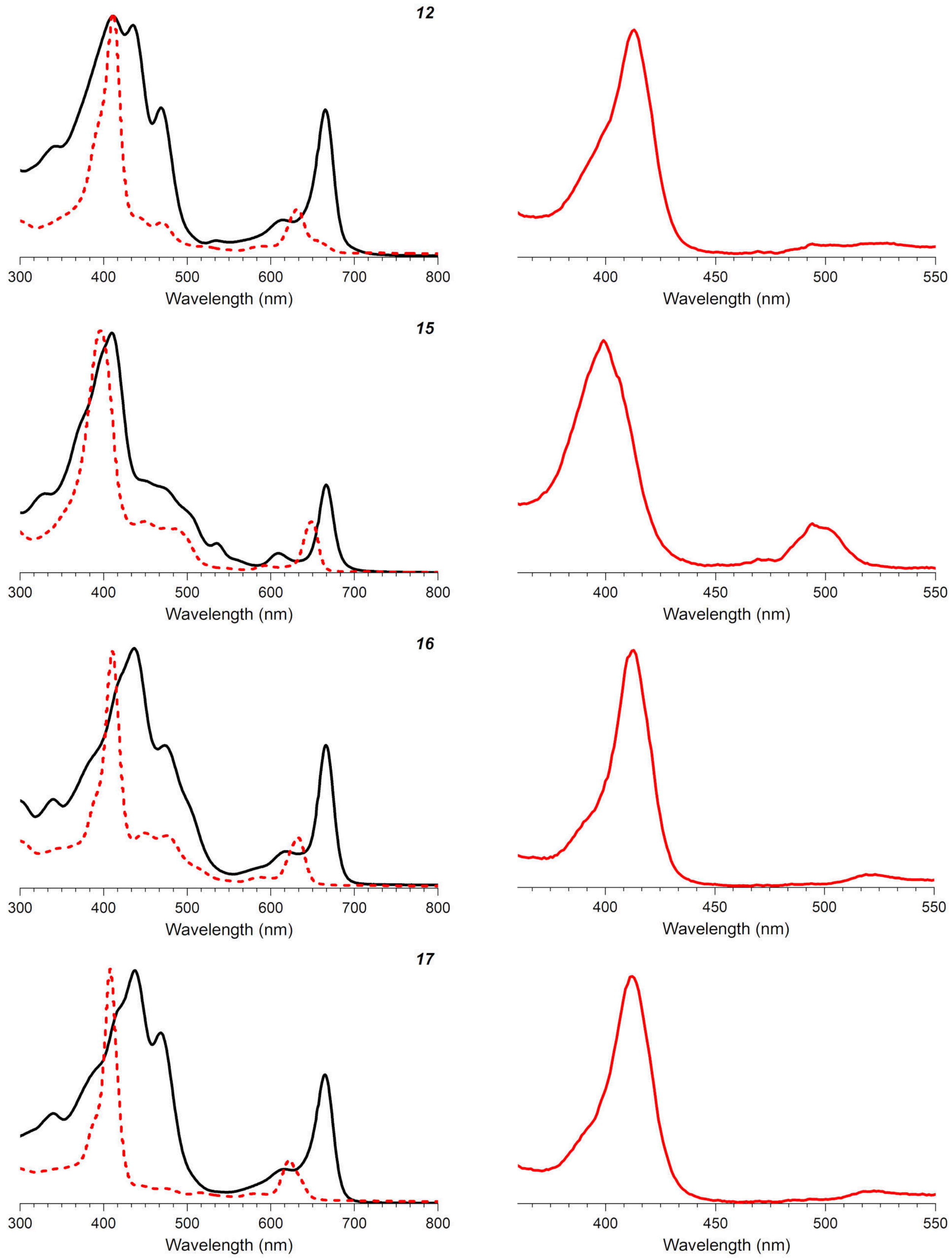
12
300
400
500
600
700
800
Wavelength (nm)
400
450
500
550
Wavelength (nm)
15
300
400
500
600
700
800
Wavelength (nm)
400
450
500
550
Wavelength (nm)
16
300
400
500
600
700
800
Wavelength (nm)
400
450
500
550
Wavelength (nm)
17
300
400
500
600
700
800
Wavelength (nm)
400
450
500
550
Wavelength (nm)

***18***

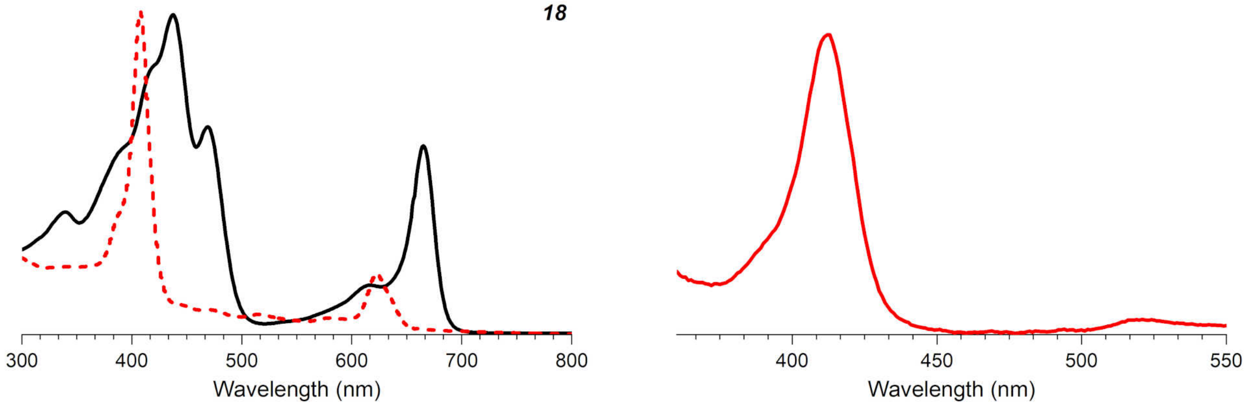
300
400
500
600
700
800
Wavelength (nm)
400
450
500
550
Wavelength (nm)

***20***

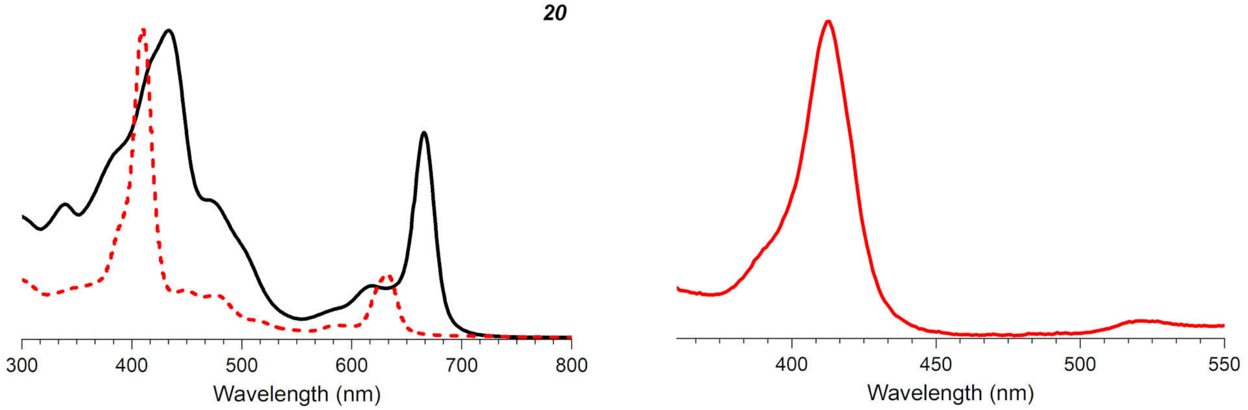
300
400
500
600
700
800
Wavelength (nm)
400
450
500
550
Wavelength (nm)

***26***

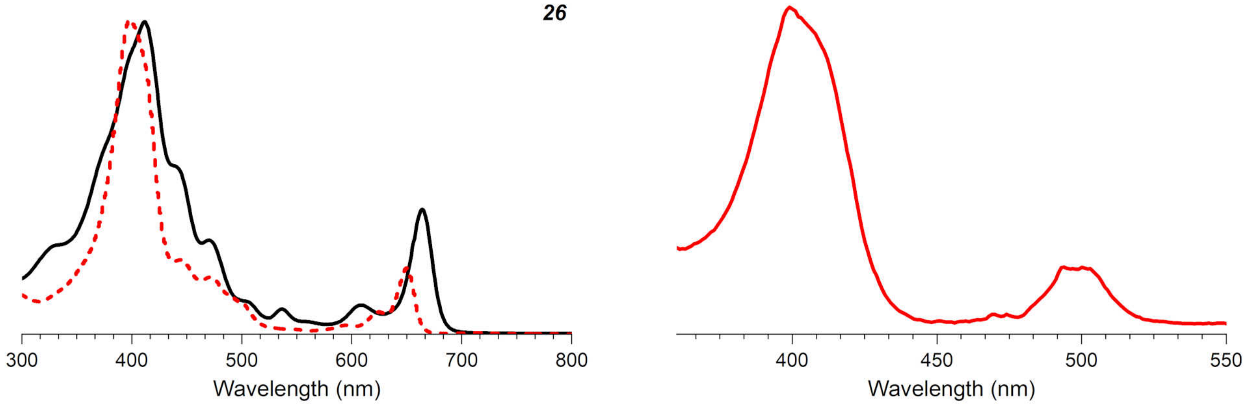
300
400
500
600
700
800
Wavelength (nm)
400
450
500
550
Wavelength (nm)

***27***

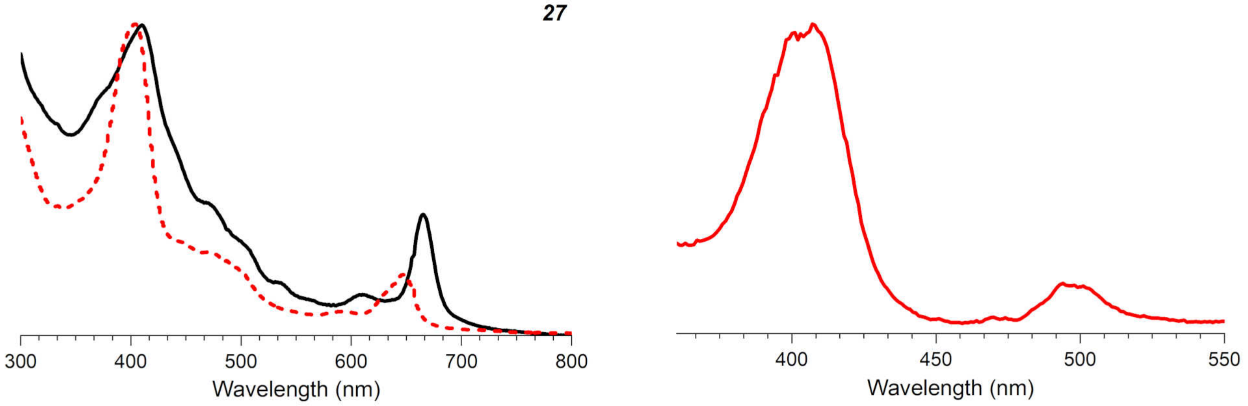
300
400
500
600
700
800
Wavelength (nm)
400
450
500
550
Wavelength (nm)

***28***

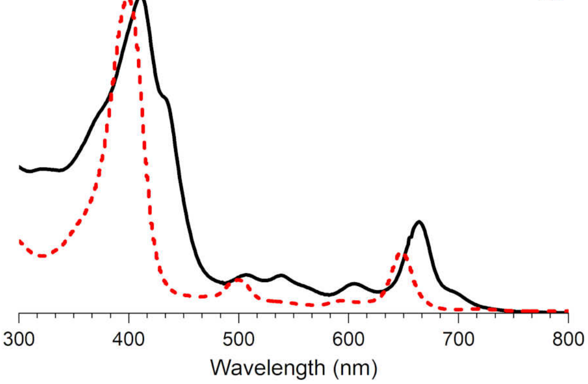
300
400
500
600
700
800
Wavelength (nm)

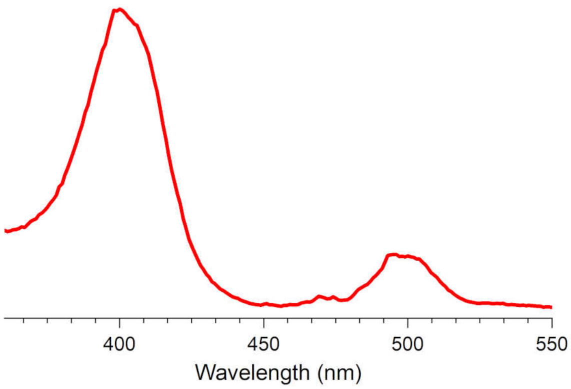
400
450
500
550
Wavelength (nm)

***29***

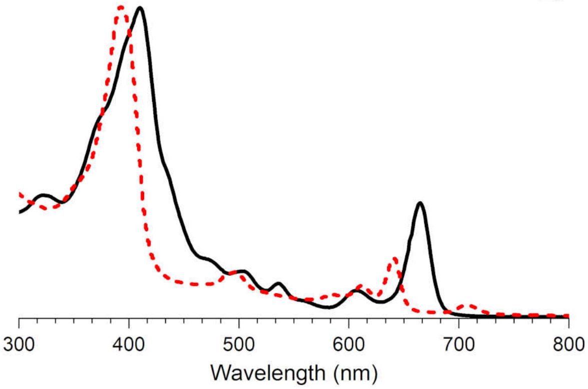
300
400
500
600
700
800
Wavelength (nm)

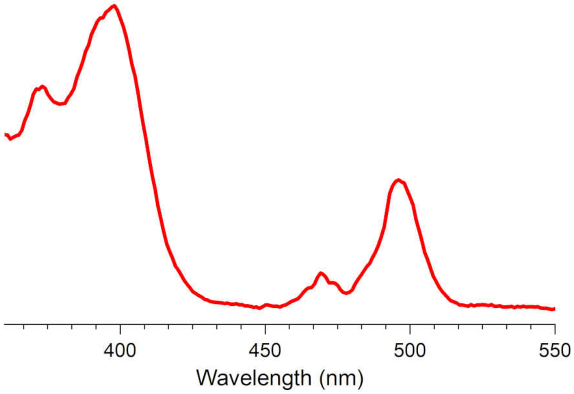
400
450
500
550
Wavelength (nm)

***30***

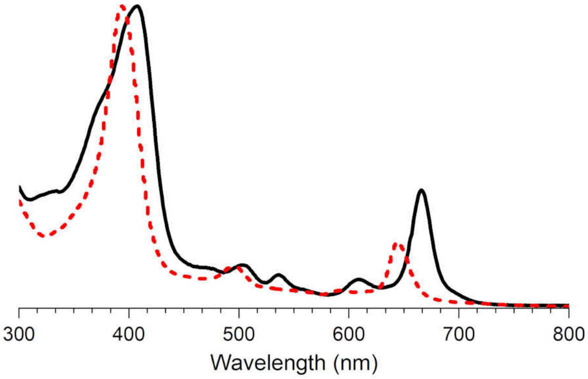
300
400
500
600
700
800
Wavelength (nm)

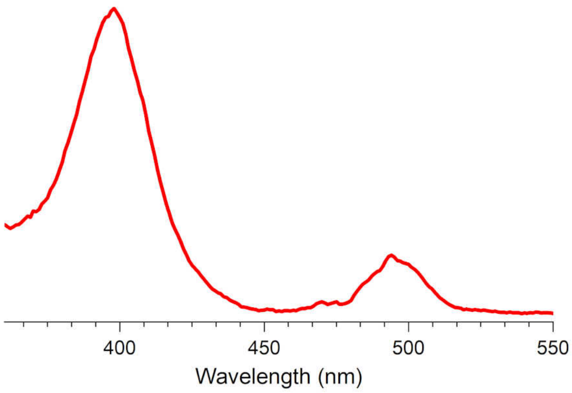
400
450
500
550
Wavelength (nm)

***32***

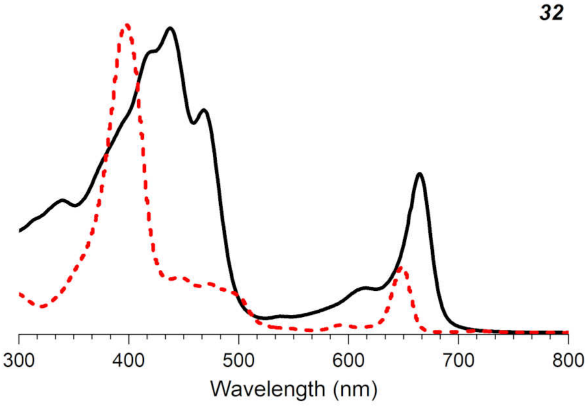
300
400
500
600
700
800
Wavelength (nm)

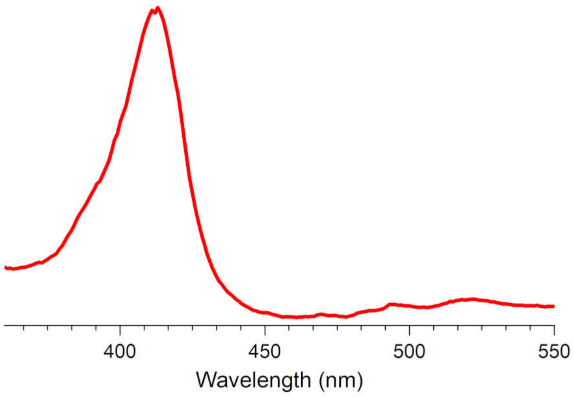
400
450
500
550
Wavelength (nm)

***34***

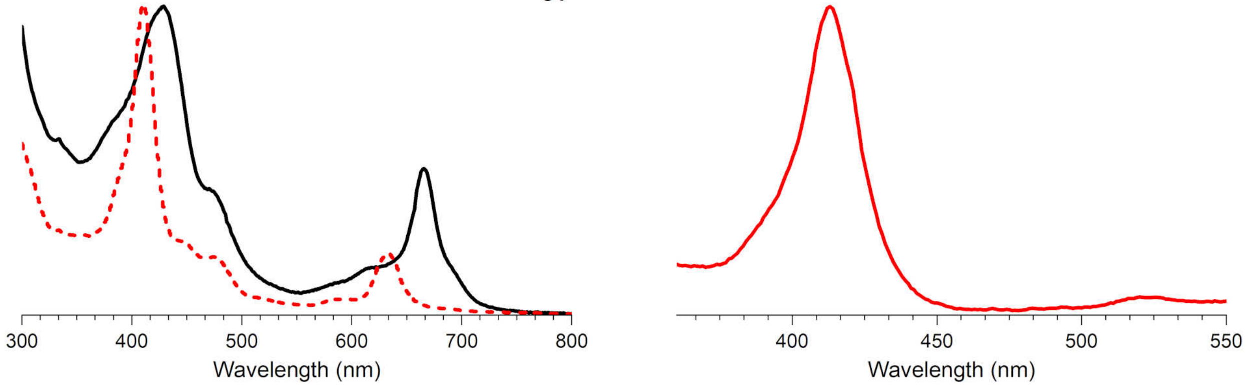
300 400 500 600 700 800
Wavelength (nm)
400 450 500 550
Wavelength (nm)

***35***

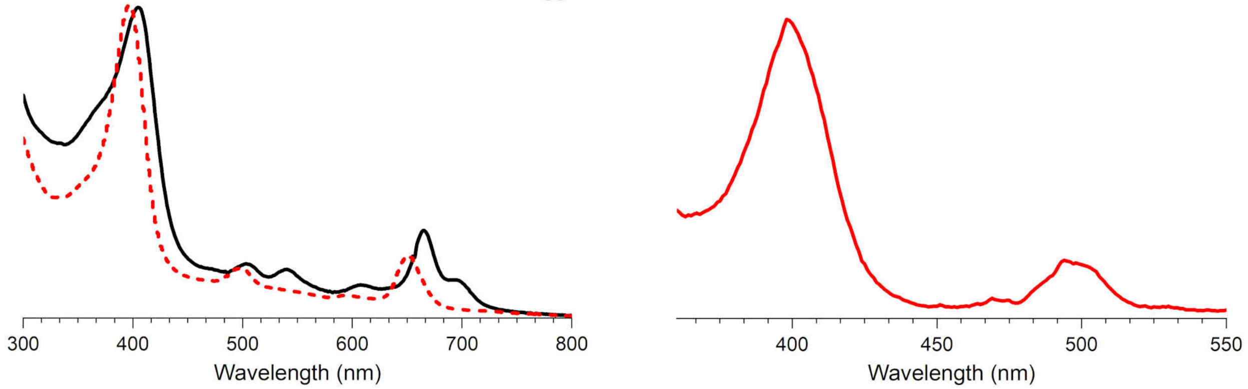
300 400 500 600 700 800
Wavelength (nm)
400 450 500 550
Wavelength (nm)

***36***

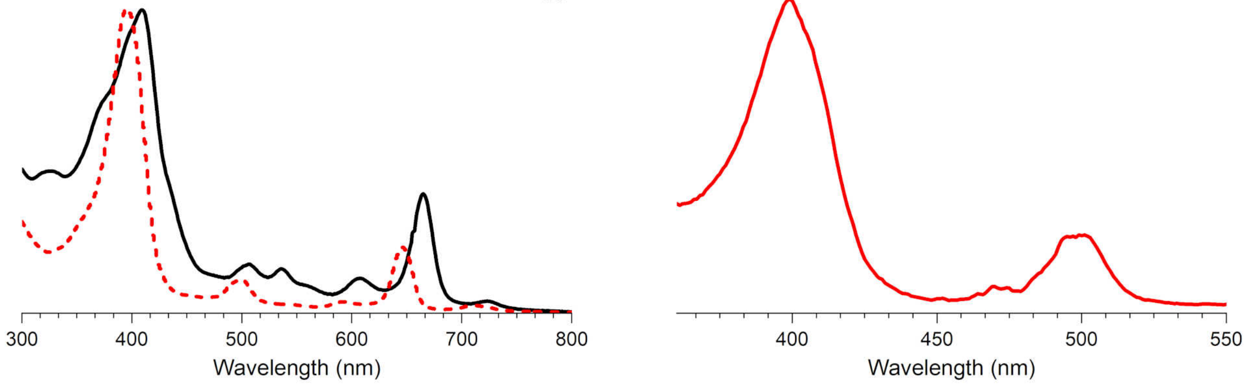
300 400 500 600 700 800
Wavelength (nm)
400 450 500 550
Wavelength (nm)

***37***

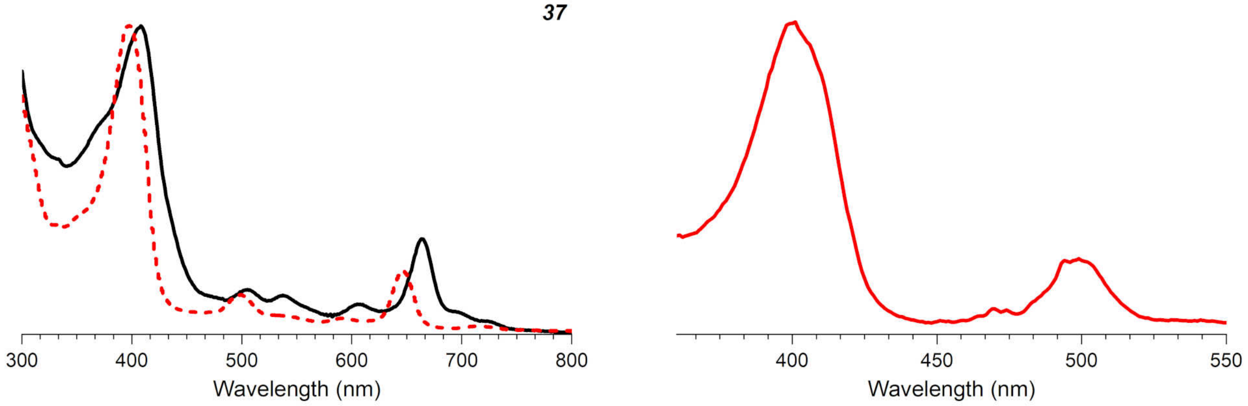
300 400 500 600 700 800
Wavelength (nm)
400 450 500 550
Wavelength (nm)

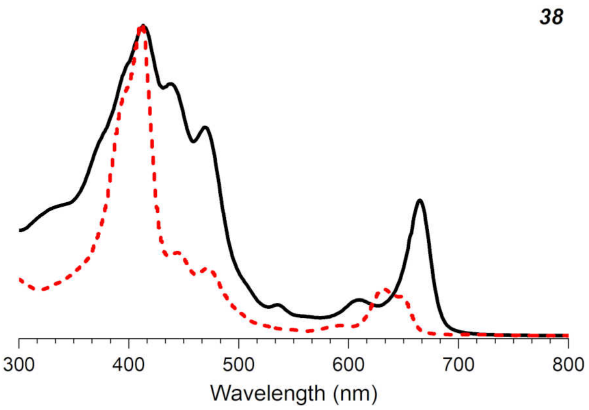
38
300
400
500
600
700
800
Wavelength (nm)

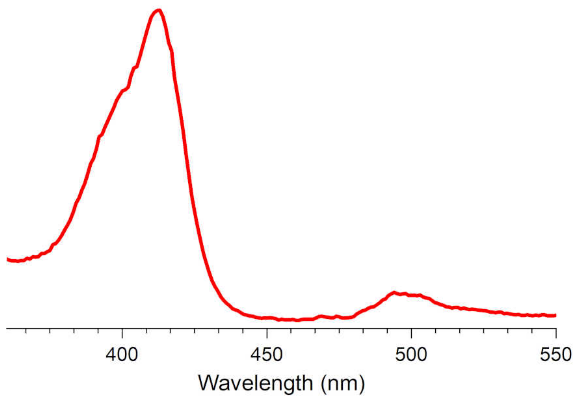
400
450
500
550
Wavelength (nm)

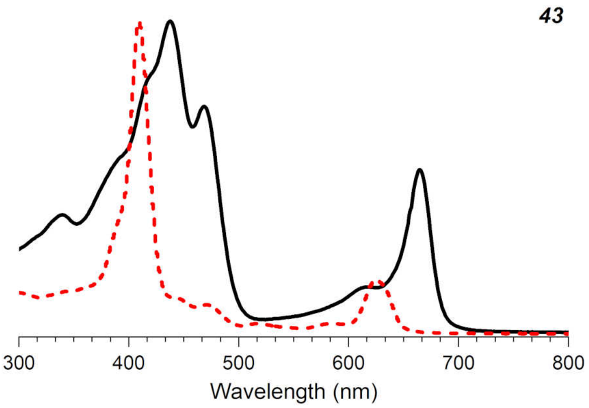
43
300
400
500
600
700
800
Wavelength (nm)

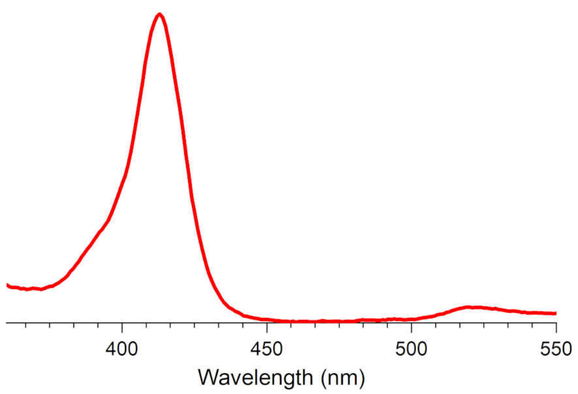
400
450
500
550
Wavelength (nm)

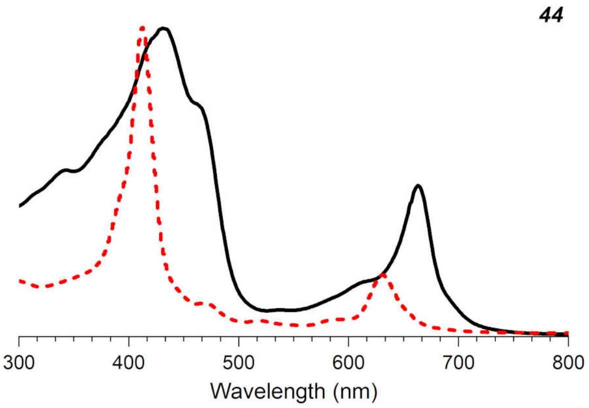
44
300
400
500
600
700
800
Wavelength (nm)

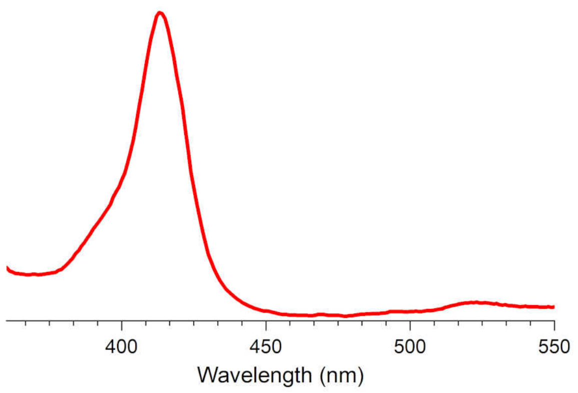
400
450
500
550
Wavelength (nm)

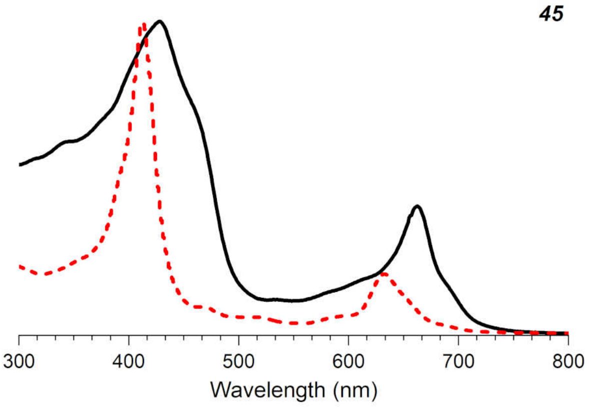
45
300
400
500
600
700
800
Wavelength (nm)

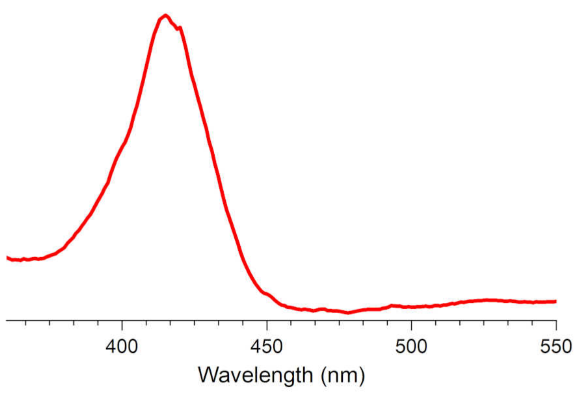
400
450
500
550
Wavelength (nm)

***46***

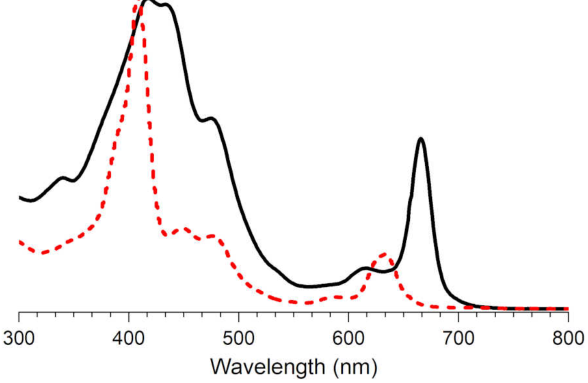
300 400 500 600 700 800
Wavelength (nm)

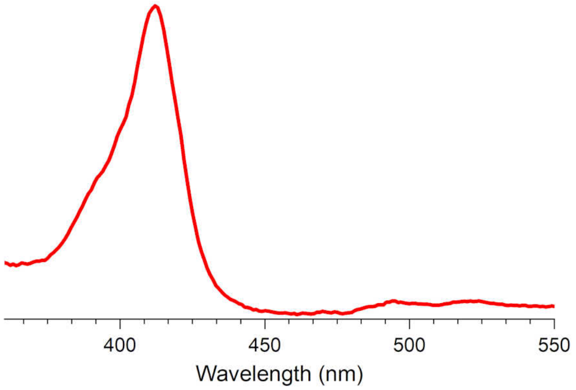
400 450 500 550
Wavelength (nm)

***48***

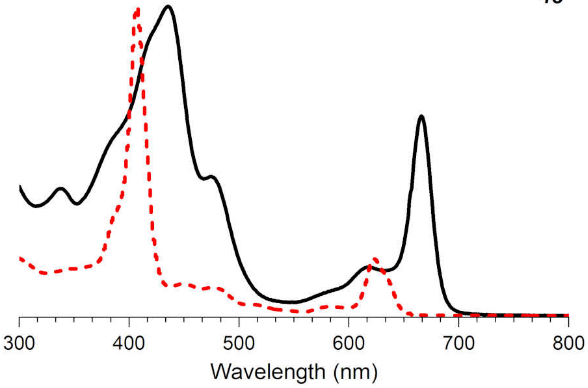
300 400 500 600 700 800
Wavelength (nm)

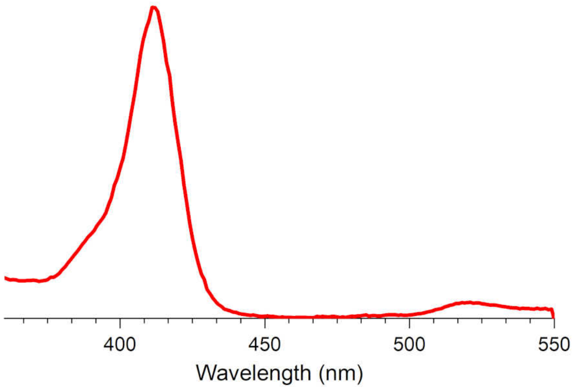
400 450 500 550
Wavelength (nm)

***49***

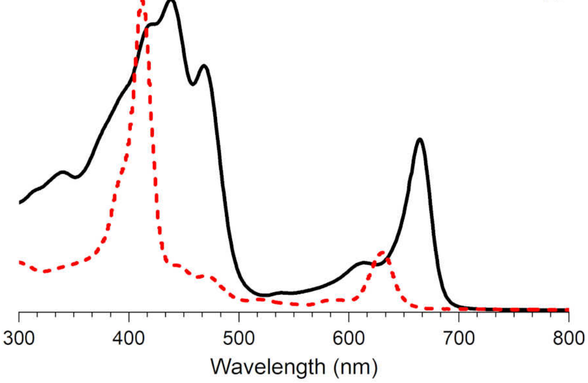
300 400 500 600 700 800
Wavelength (nm)

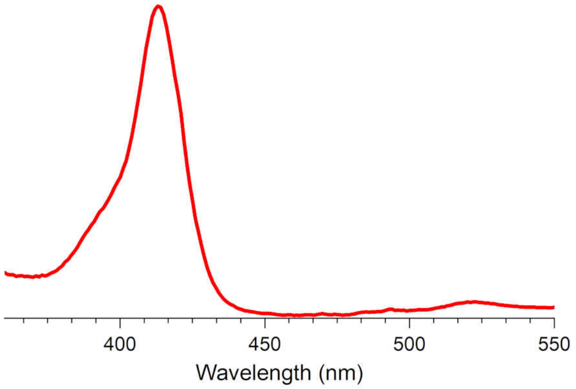
400 450 500 550
Wavelength (nm)

***50***

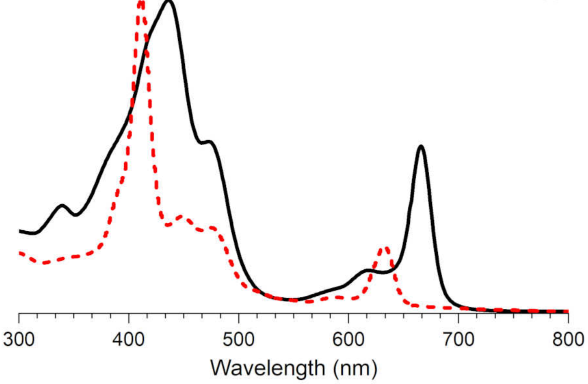
300 400 500 600 700 800
Wavelength (nm)

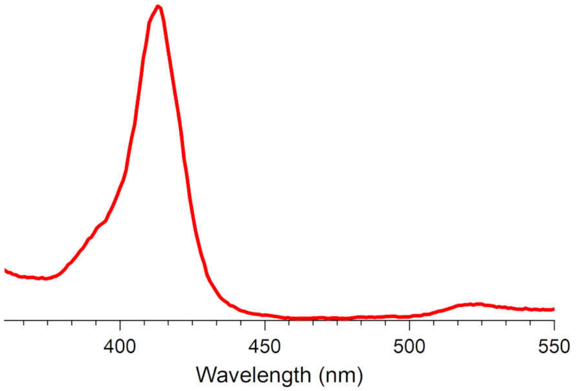
400 450 500 550
Wavelength (nm)

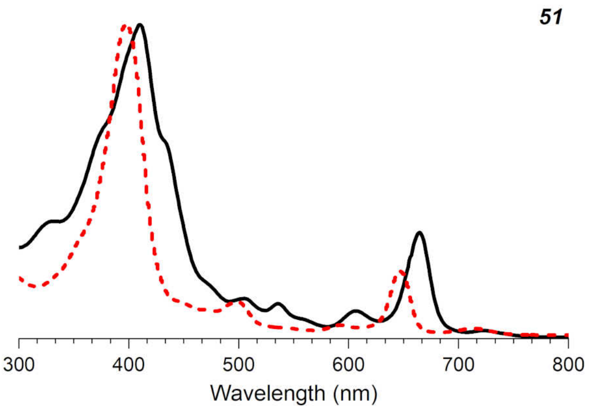
51
300
400
500
600
700
800
Wavelength (nm)

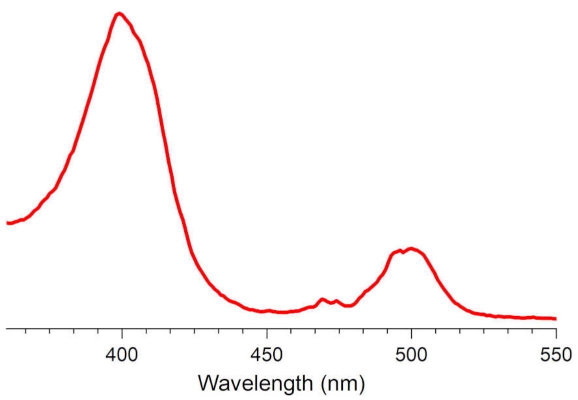
400
450
500
550
Wavelength (nm)

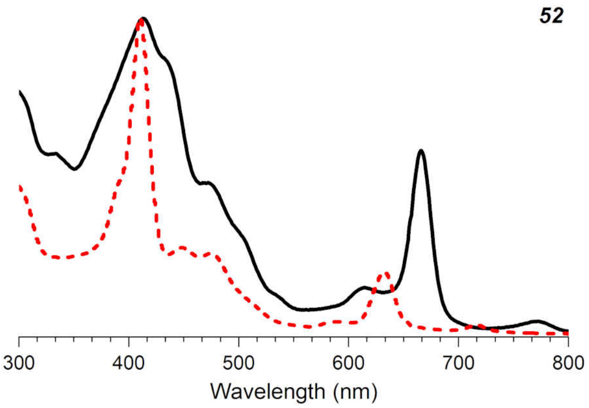
52
300
400
500
600
700
800
Wavelength (nm)

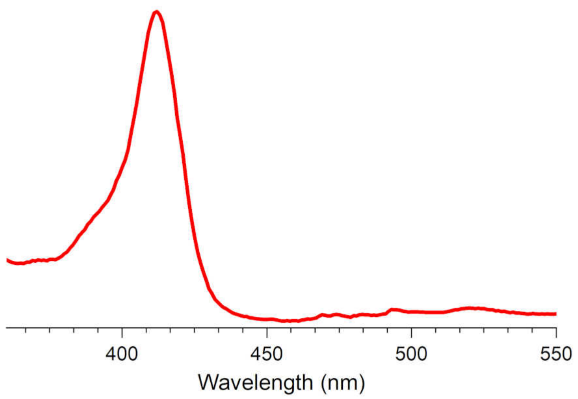
400
450
500
550
Wavelength (nm)

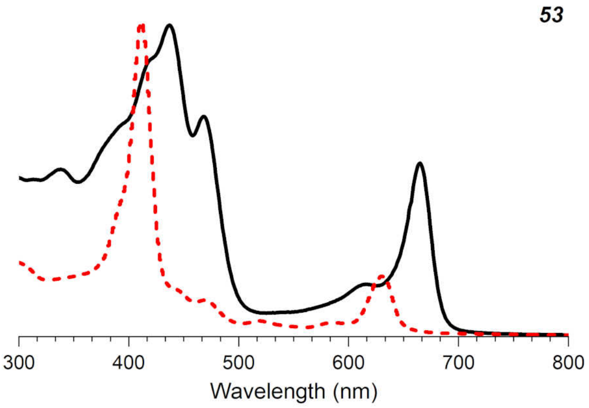
53
300
400
500
600
700
800
Wavelength (nm)

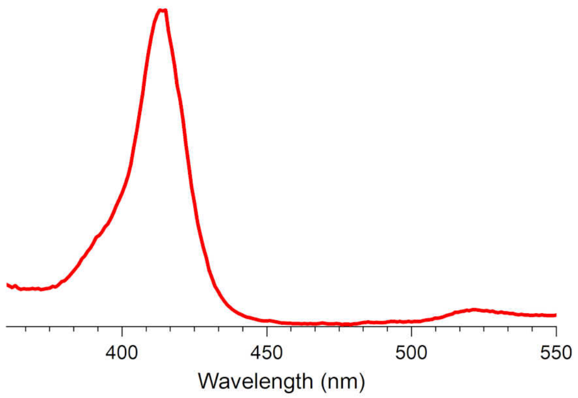
400
450
500
550
Wavelength (nm)

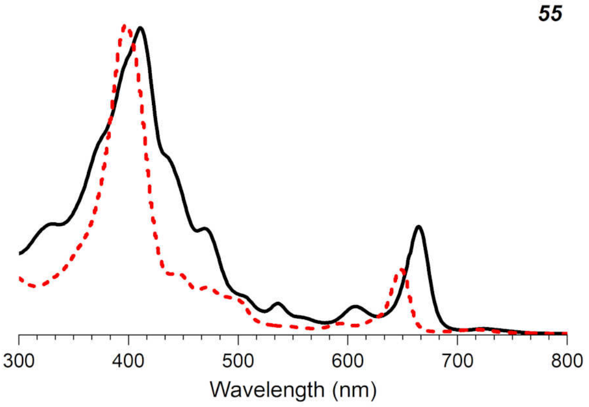
55
300
400
500
600
700
800
Wavelength (nm)

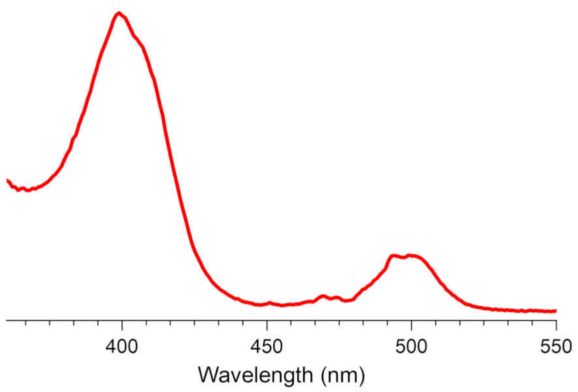
400
450
500
550
Wavelength (nm)

***58***

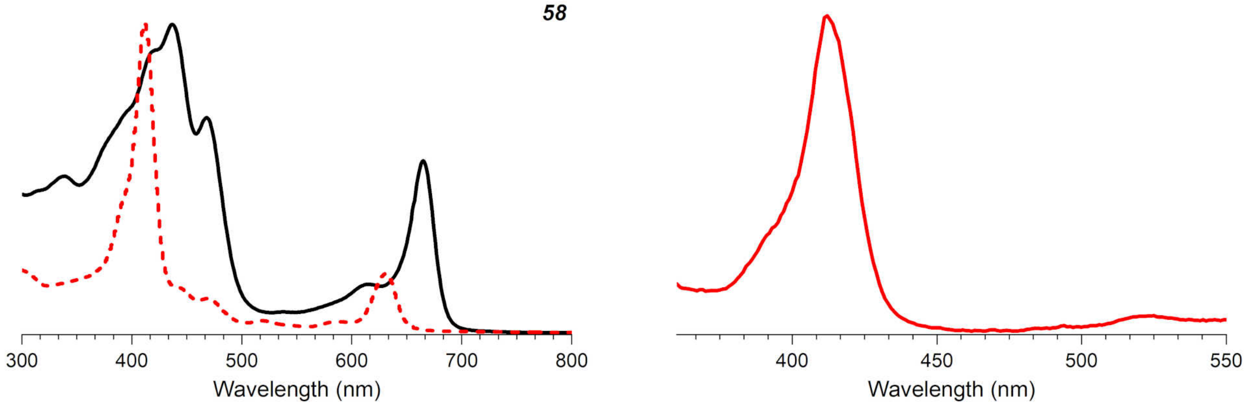
300 400 500 600 700 800
Wavelength (nm)
400 450 500 550
Wavelength (nm)

***59***

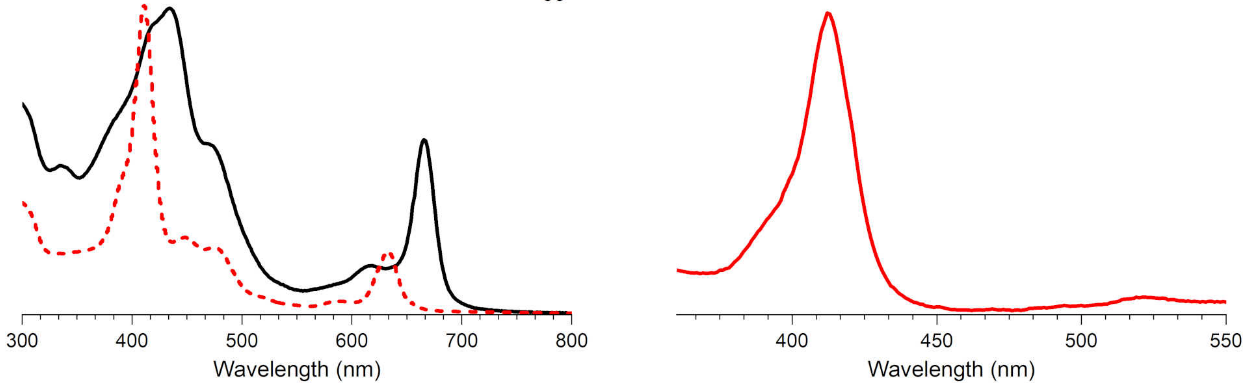
300 400 500 600 700 800
Wavelength (nm)
400 450 500 550
Wavelength (nm)

***60***

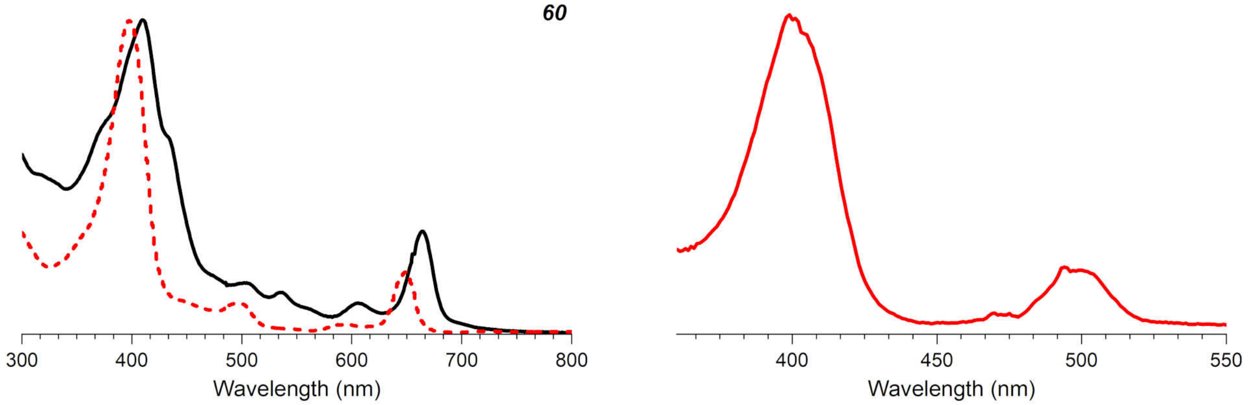
300 400 500 600 700 800
Wavelength (nm)
400 450 500 550
Wavelength (nm)

***61***

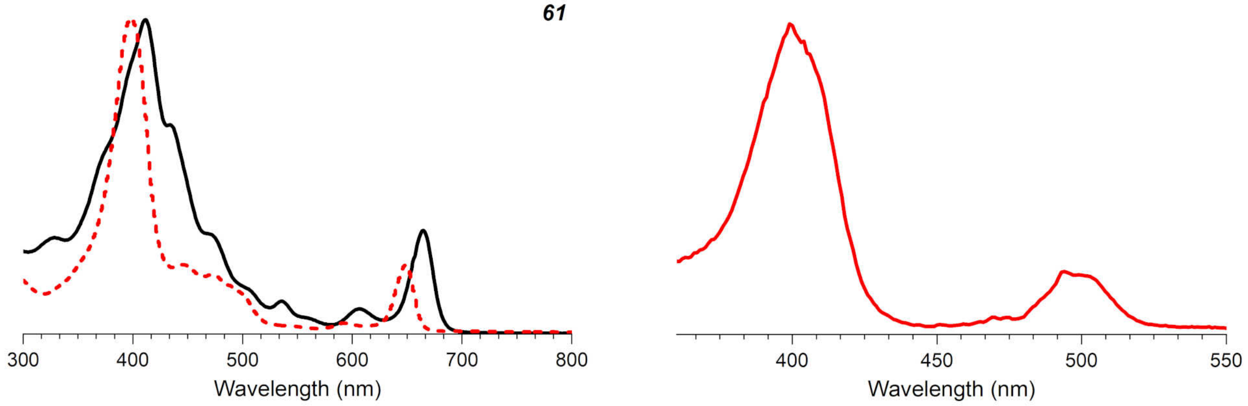
300 400 500 600 700 800
Wavelength (nm)
400 450 500 550
Wavelength (nm)

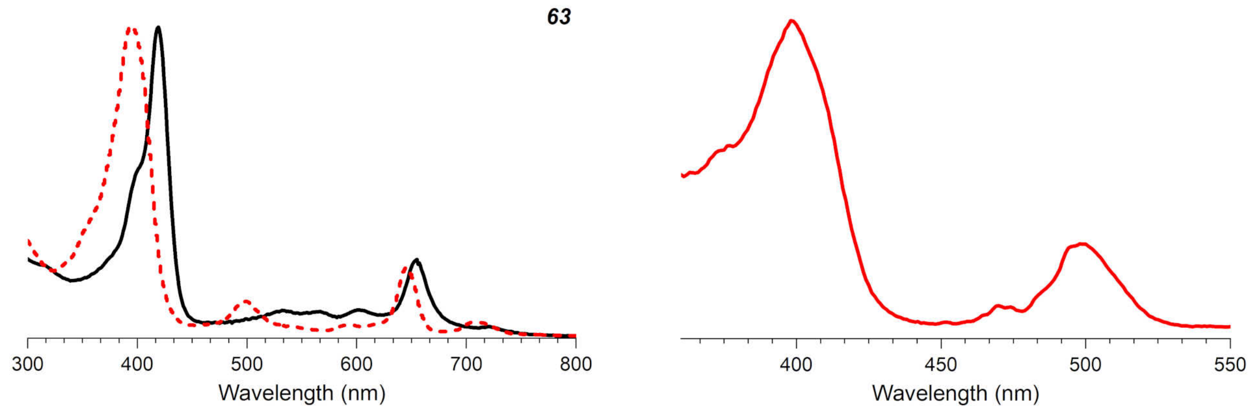
63
300 400 500 600 700 800
Wavelength (nm)
400 450 500 550
Wavelength (nm)

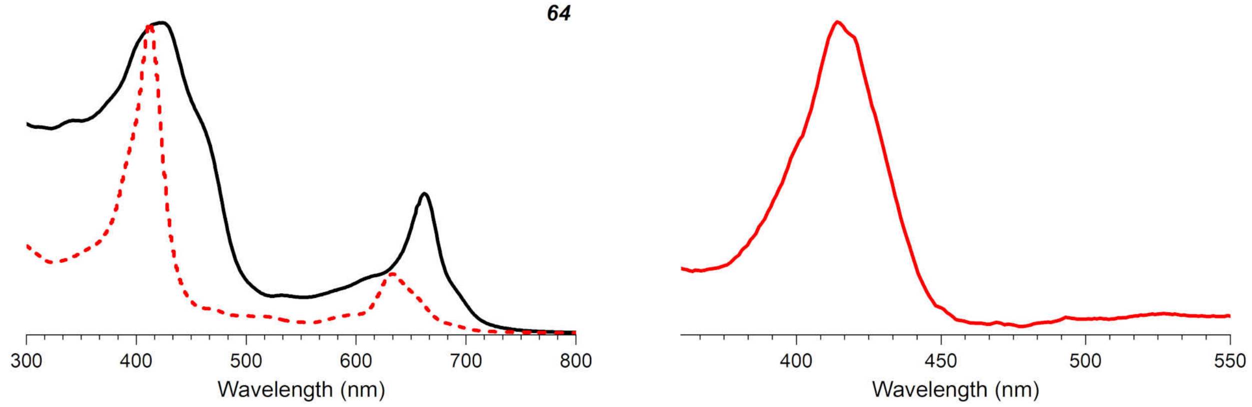
64
300 400 500 600 700 800
Wavelength (nm)
400 450 500 550
Wavelength (nm)

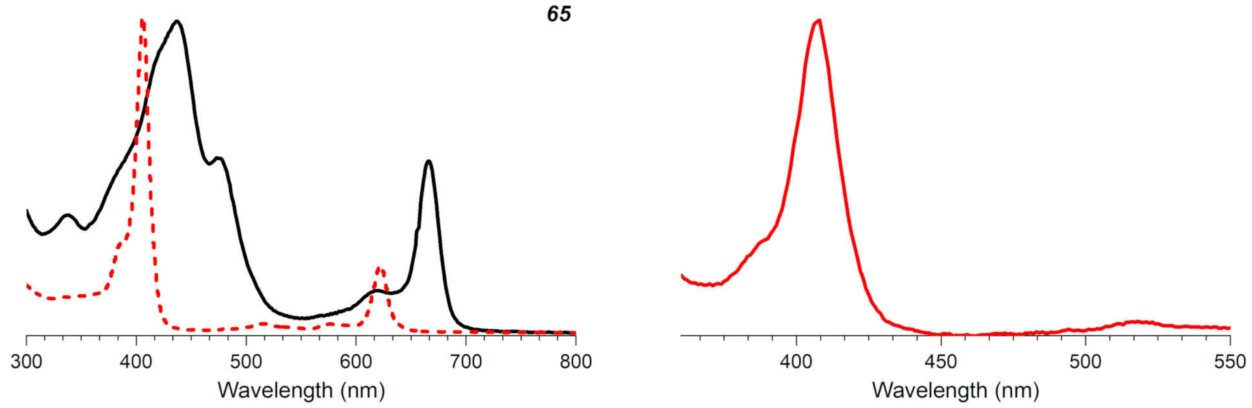
65
300 400 500 600 700 800
Wavelength (nm)
400 450 500 550
Wavelength (nm)

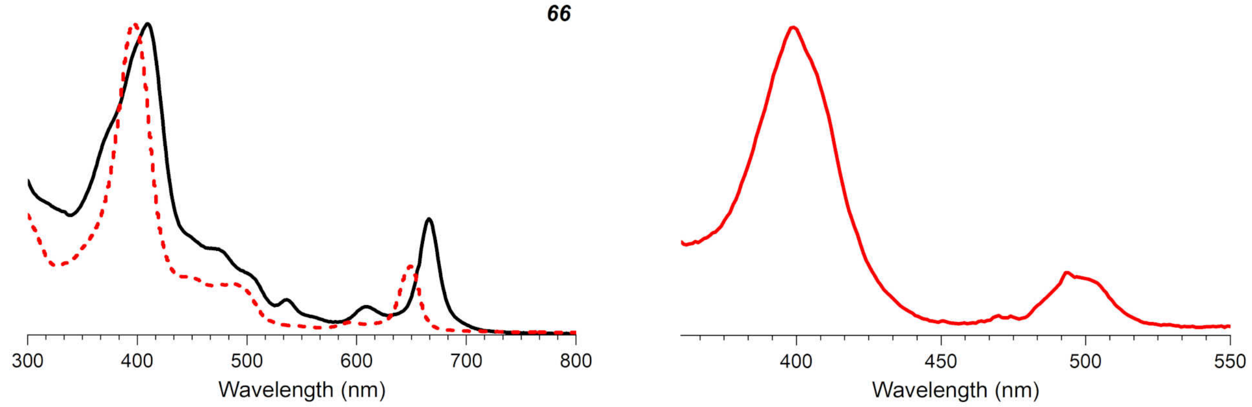
66
300 400 500 600 700 800
Wavelength (nm)
400 450 500 550
Wavelength (nm)

***67***

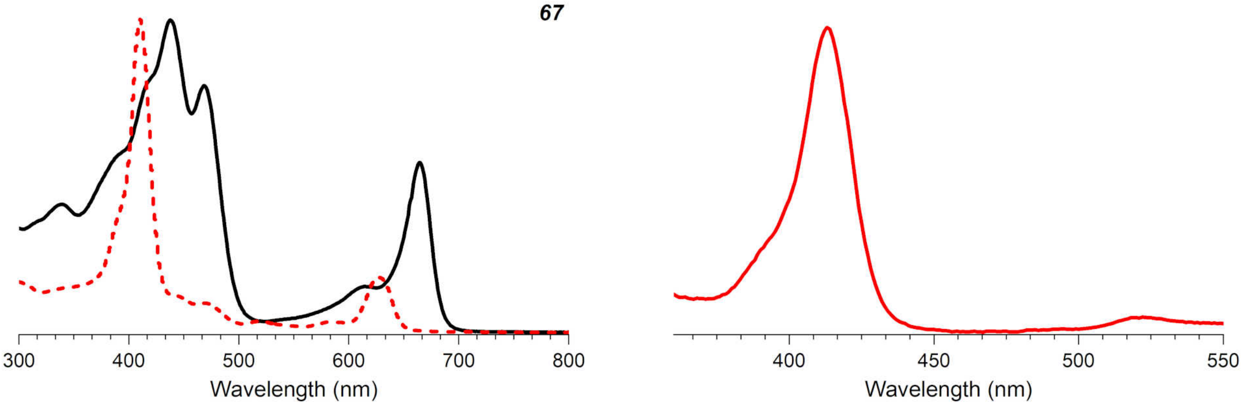
300
400
500
600
700
800
Wavelength (nm)
400
450
500
550
Wavelength (nm)

***68***

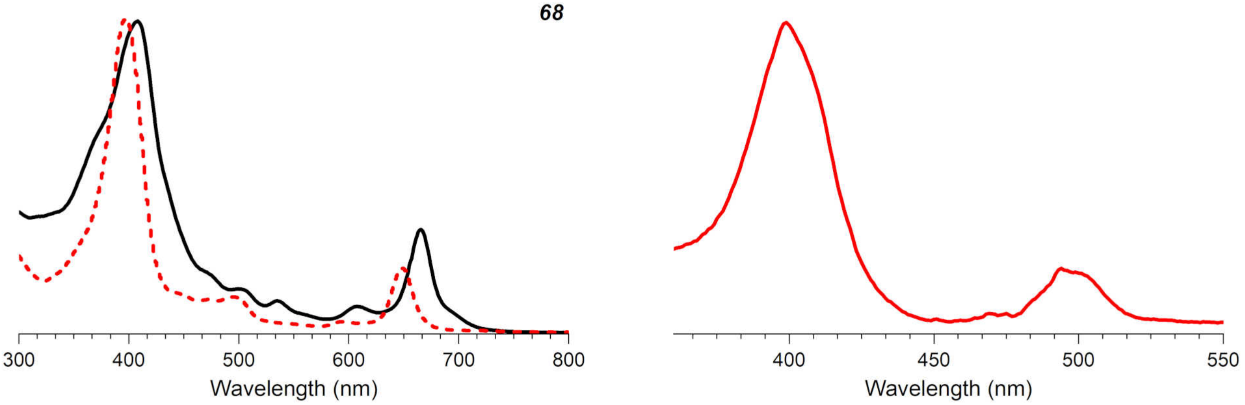
300
400
500
600
700
800
Wavelength (nm)
400
450
500
550
Wavelength (nm)

***69***

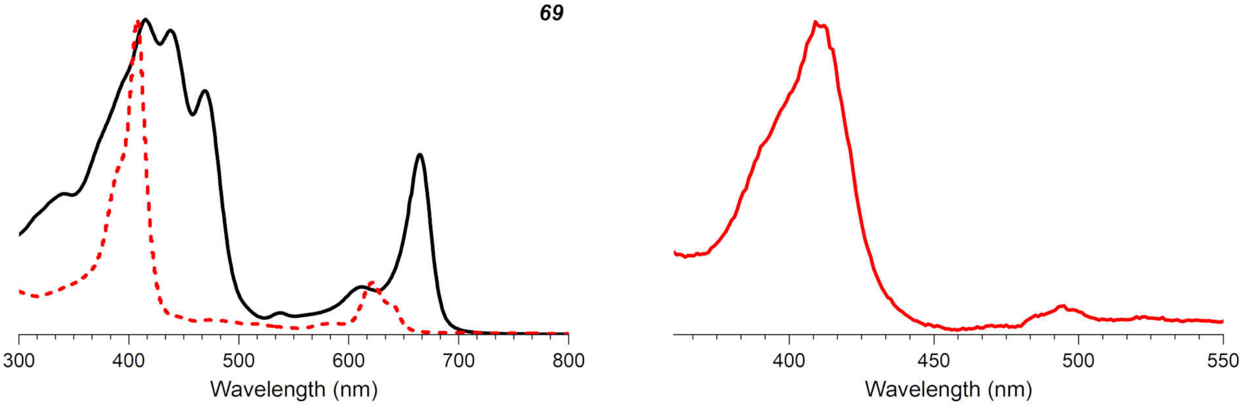
300
400
500
600
700
800
Wavelength (nm)
400
450
500
550
Wavelength (nm)

***70***

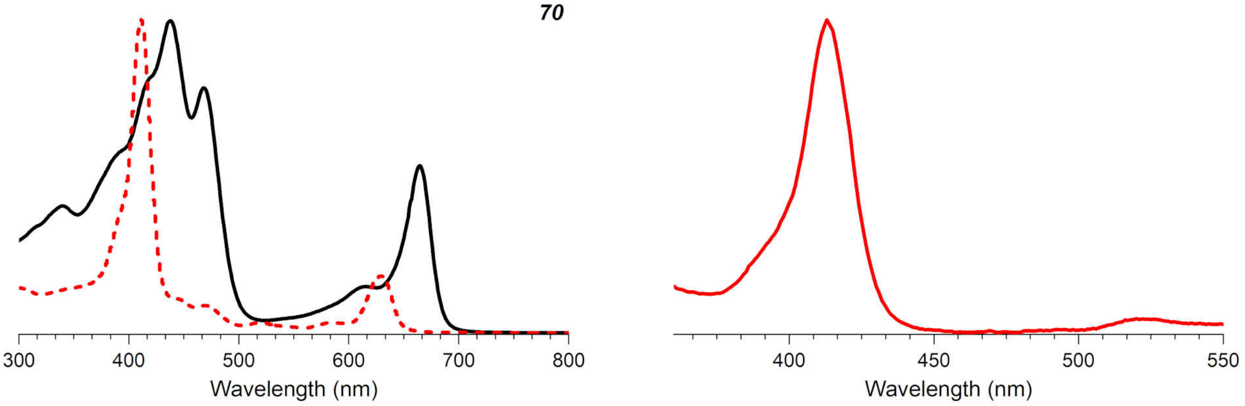
300
400
500
600
700
800
Wavelength (nm)
400
450
500
550
Wavelength (nm)

***71***

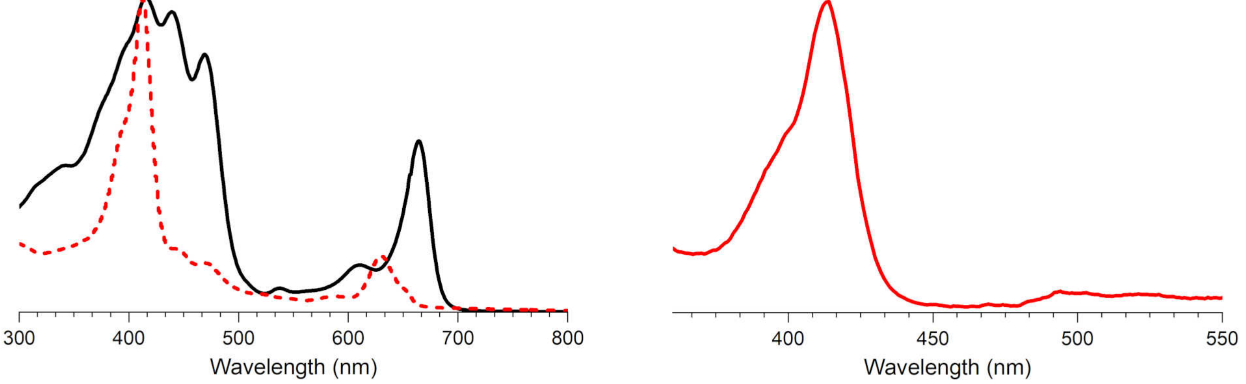
300
400
500
600
700
800
Wavelength (nm)
400
450
500
550
Wavelength (nm)

***73***

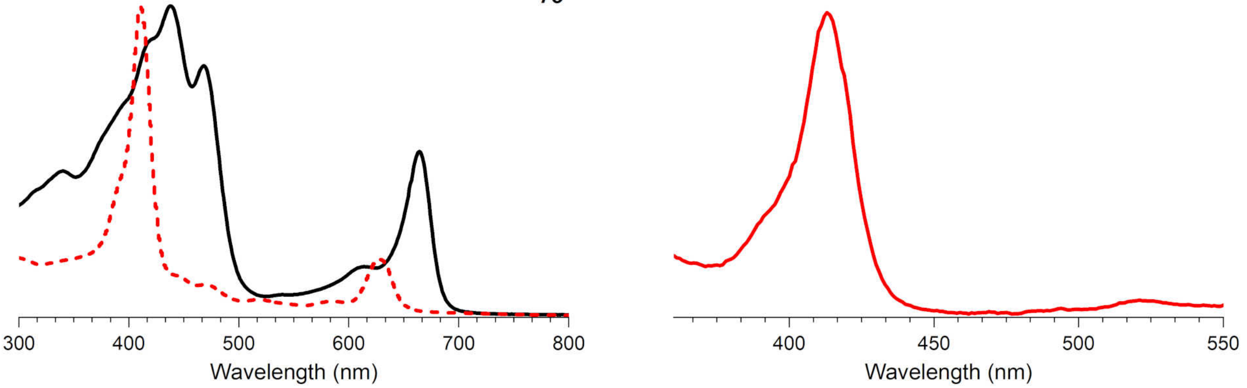
300
400
500
600
700
800
Wavelength (nm)
400
450
500
550
Wavelength (nm)

***74***

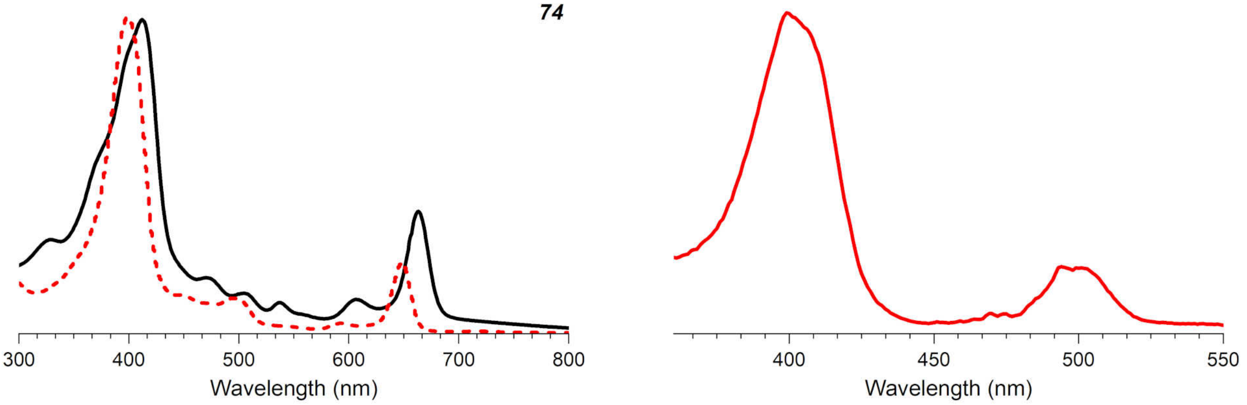
300
400
500
600
700
800
Wavelength (nm)
400
450
500
550
Wavelength (nm)

***75***

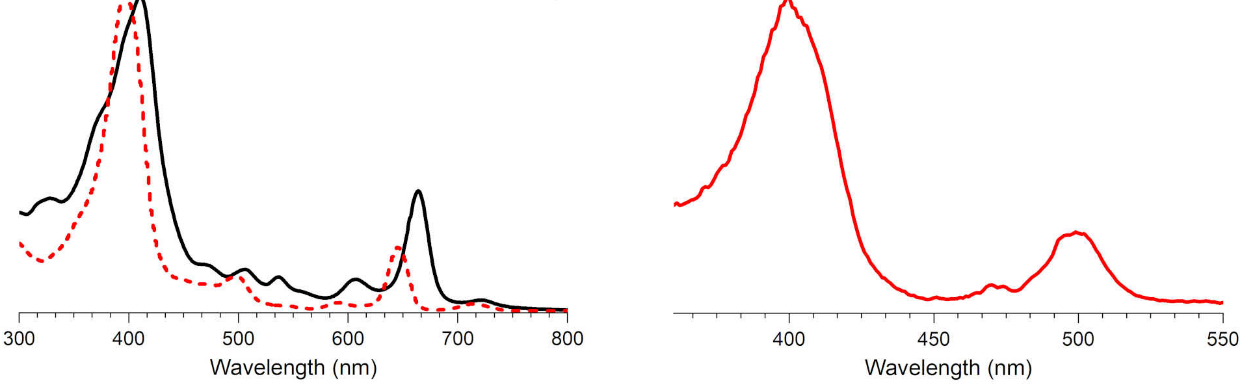
300
400
500
600
700
800
Wavelength (nm)
400
450
500
550
Wavelength (nm)

*77*

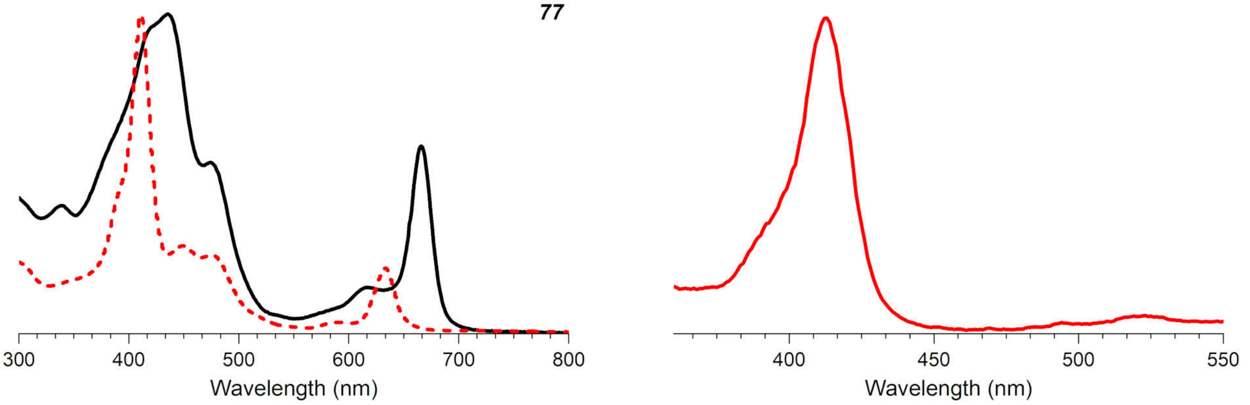
300 400 500 600 700 800
Wavelength (nm)
400 450 500 550
Wavelength (nm)

*81*

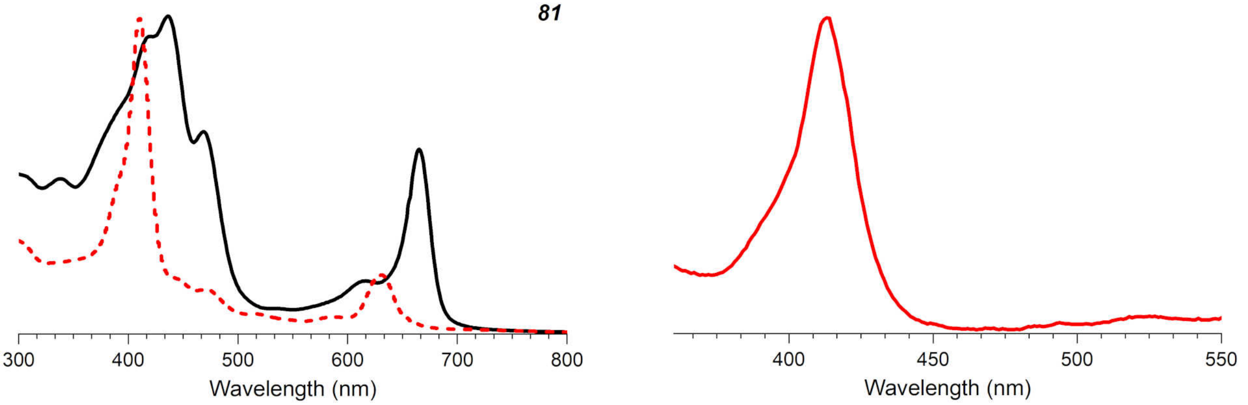
300 400 500 600 700 800
Wavelength (nm)
400 450 500 550
Wavelength (nm)

*82*

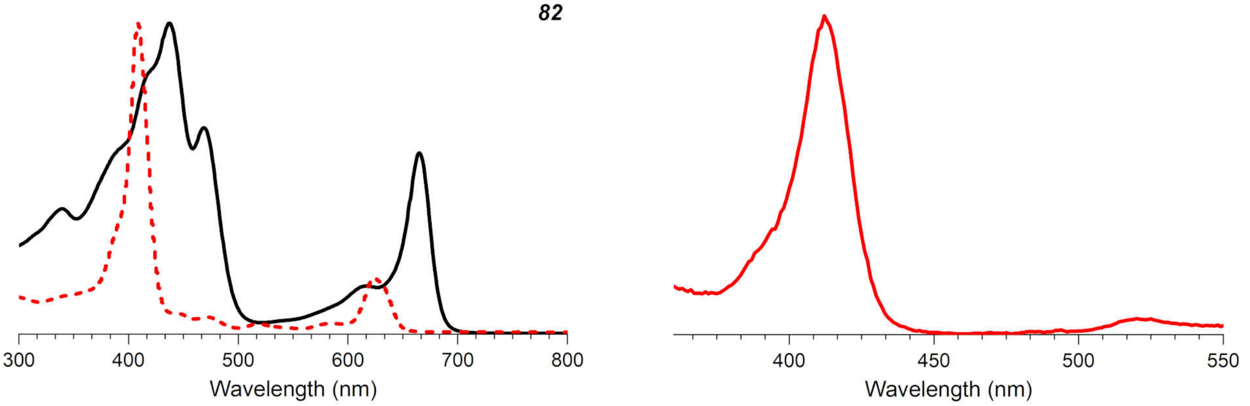
300 400 500 600 700 800
Wavelength (nm)
400 450 500 550
Wavelength (nm)

*83*

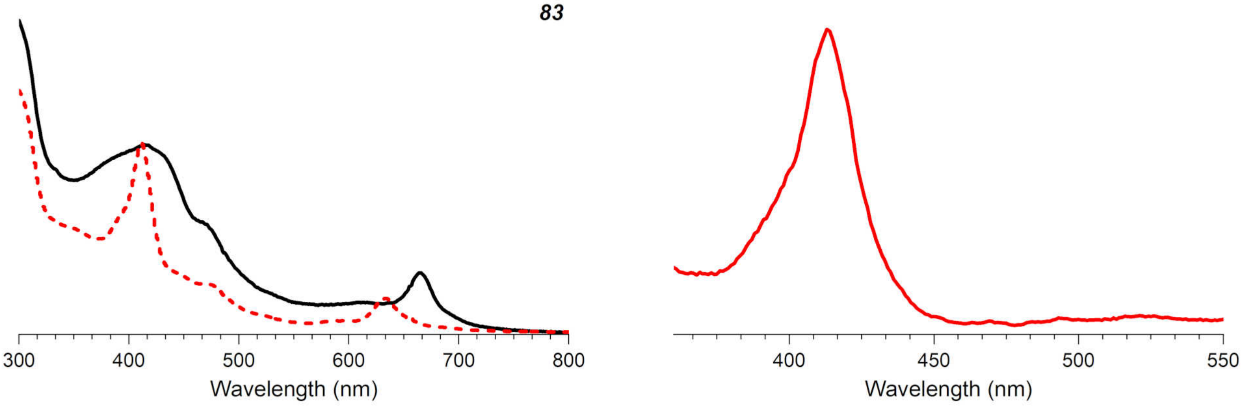
300 400 500 600 700 800
Wavelength (nm)
400 450 500 550
Wavelength (nm)

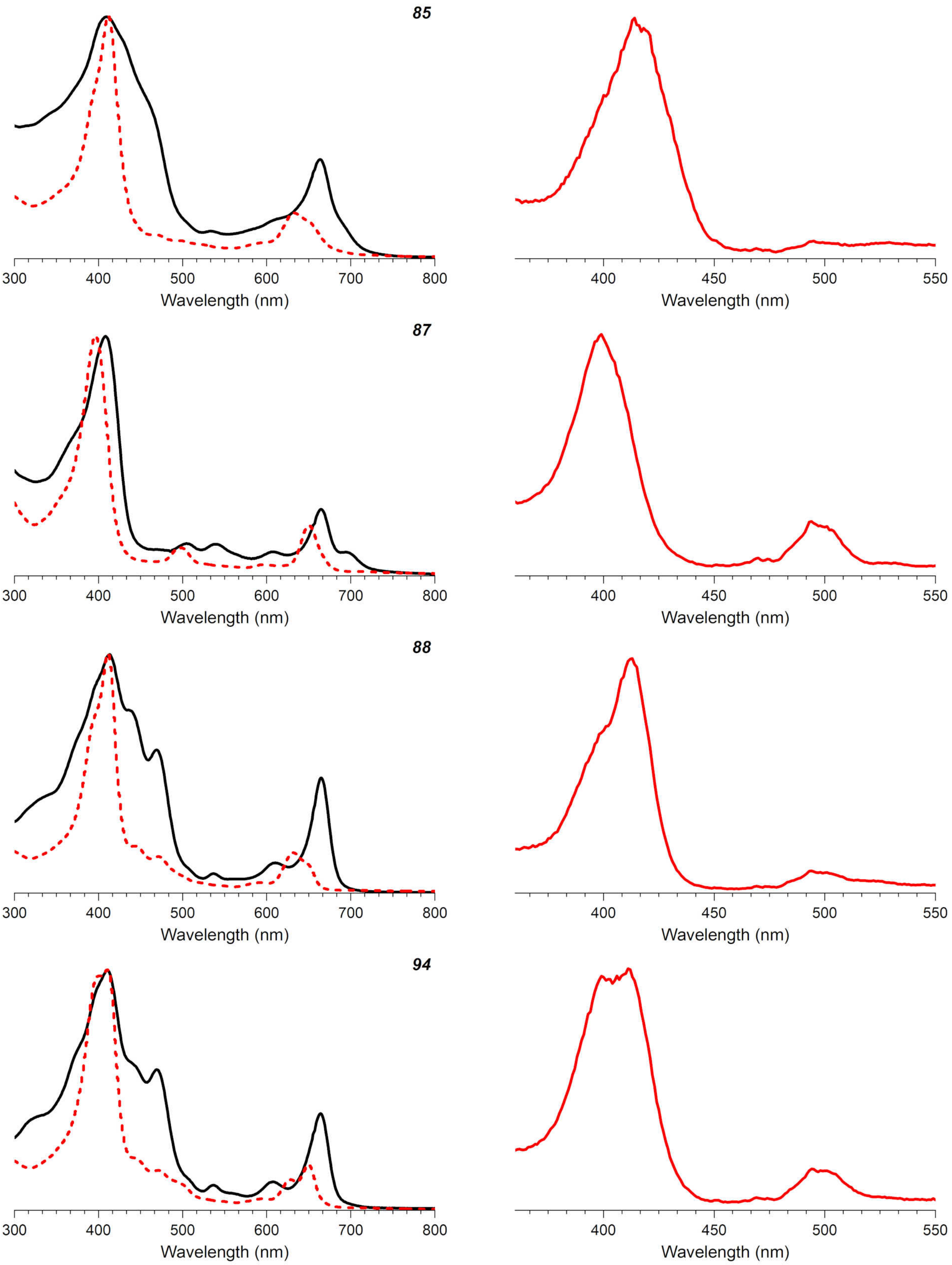
85
87
88
94
300
400
500
600
700
800
Wavelength (nm)
400
450
500
550
Wavelength (nm)

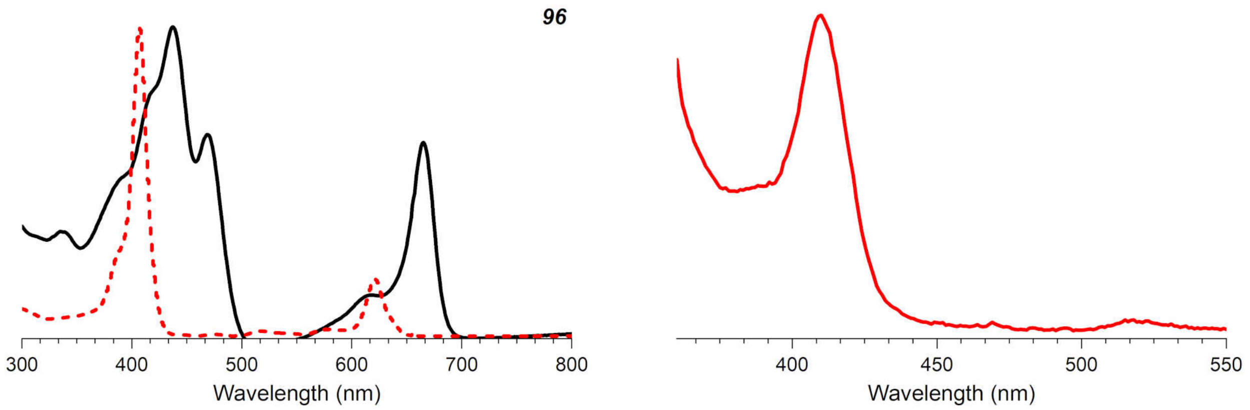
96
300 400 500 600 700 800
Wavelength (nm)
400 450 500 550
Wavelength (nm)

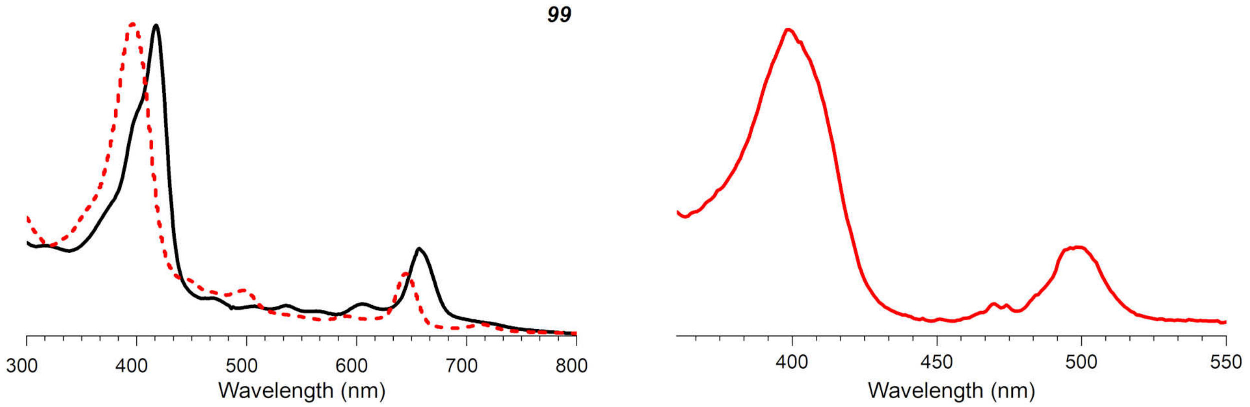
99
300 400 500 600 700 800
Wavelength (nm)
400 450 500 550
Wavelength (nm)

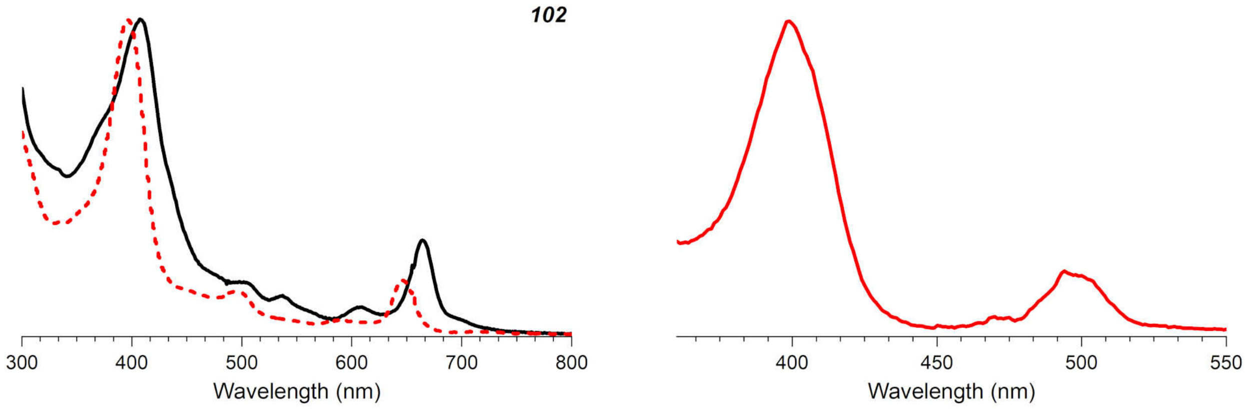
102
300 400 500 600 700 800
Wavelength (nm)
400 450 500 550
Wavelength (nm)

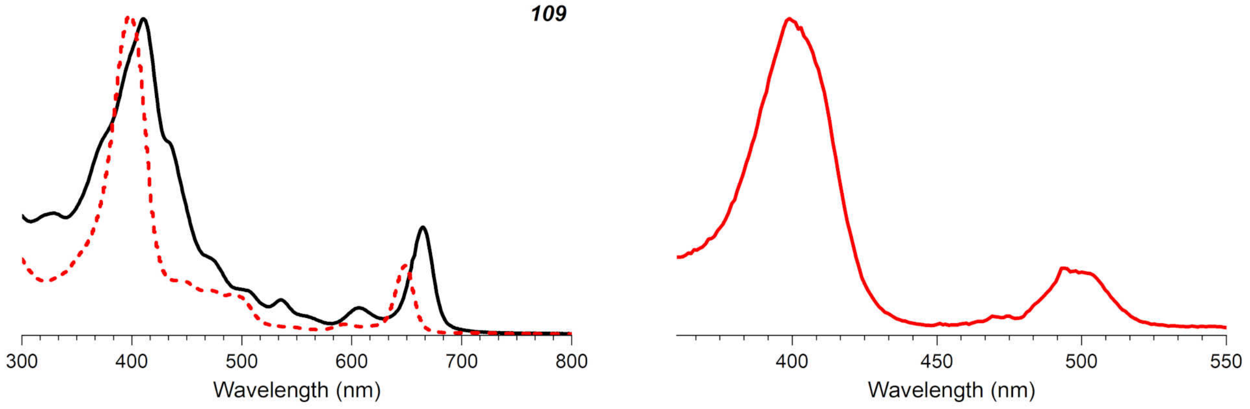
109
300 400 500 600 700 800
Wavelength (nm)
400 450 500 550
Wavelength (nm)

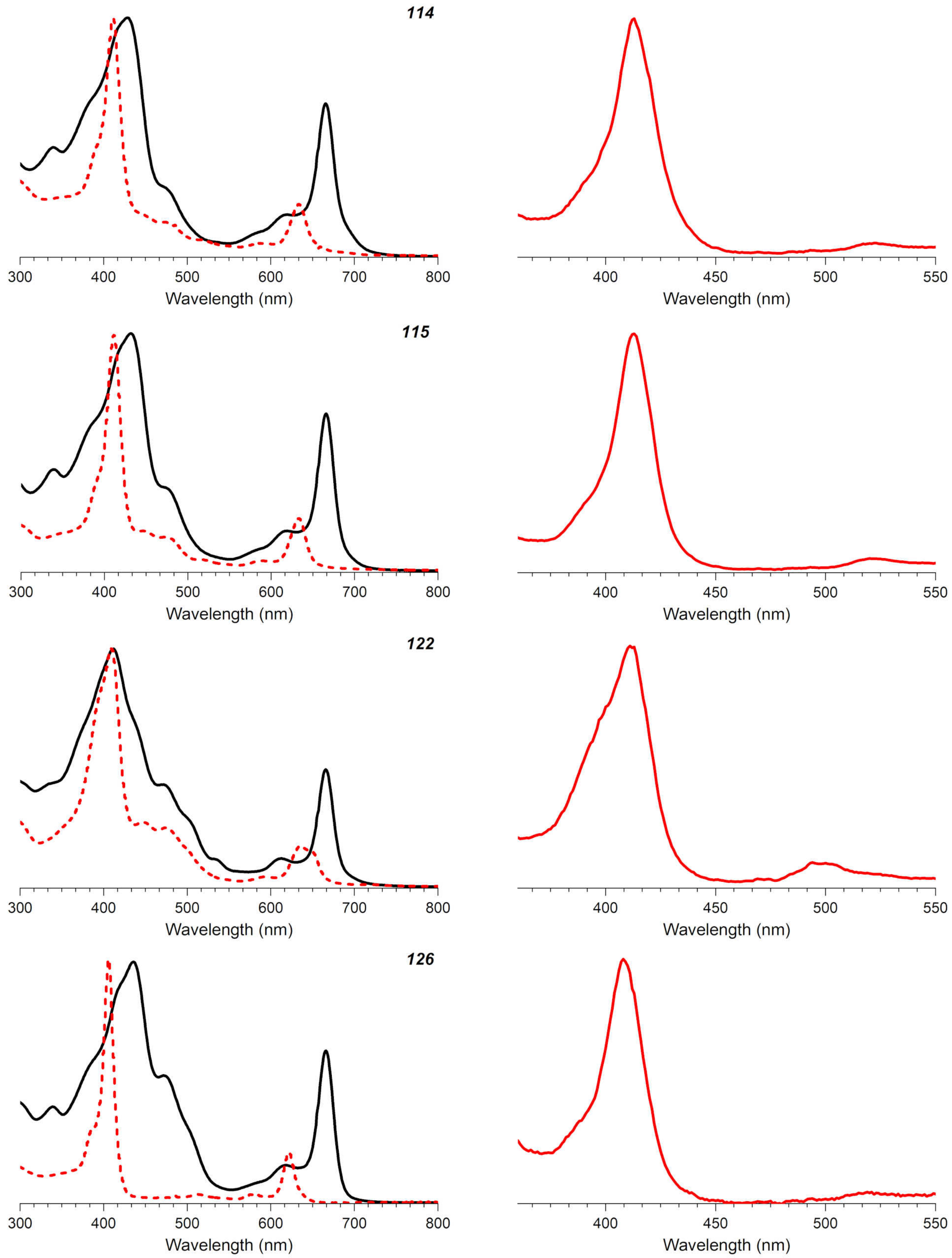
114
300 400 500 600 700 800
Wavelength (nm)
400 450 500 550
Wavelength (nm)
115
300 400 500 600 700 800
Wavelength (nm)
400 450 500 550
Wavelength (nm)
122
300 400 500 600 700 800
Wavelength (nm)
400 450 500 550
Wavelength (nm)
126
300 400 500 600 700 800
Wavelength (nm)
400 450 500 550
Wavelength (nm)

***127***

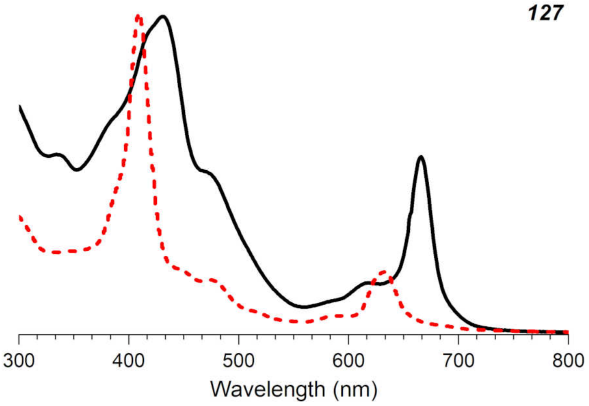


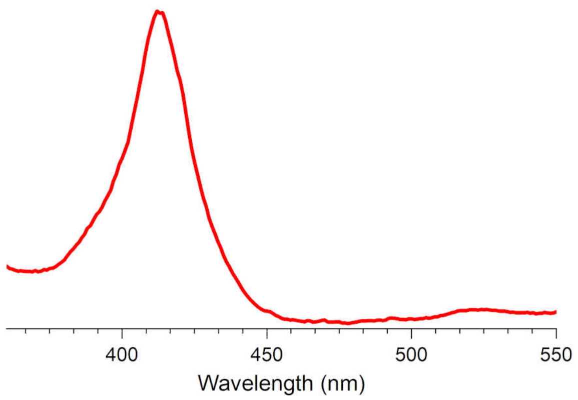


***131***

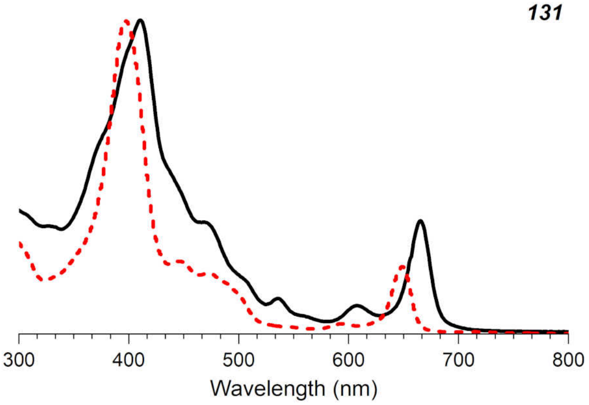


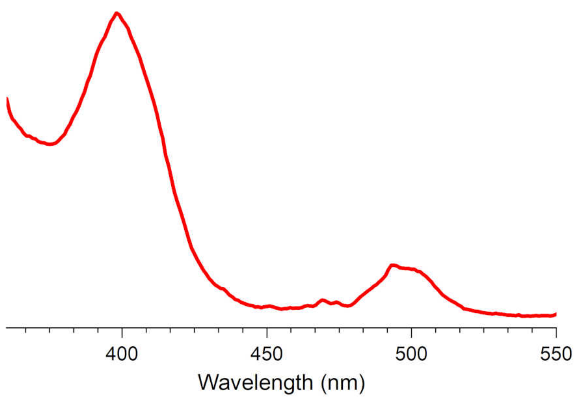


***133***

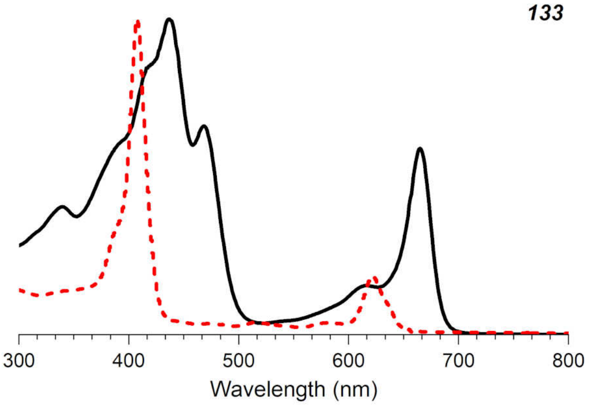


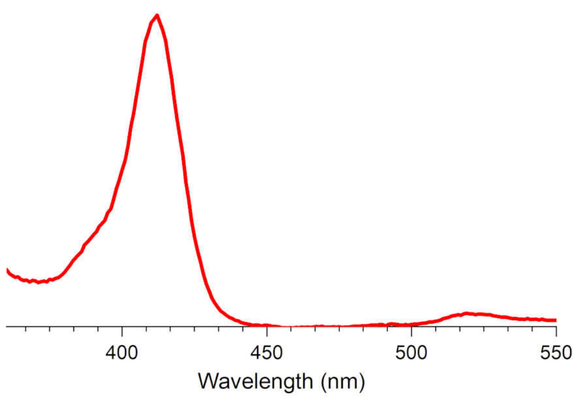


***134***

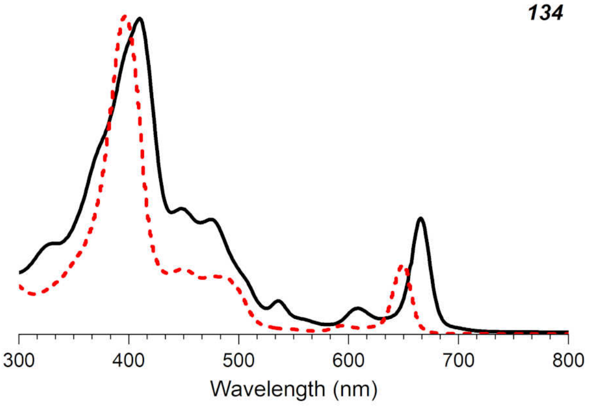


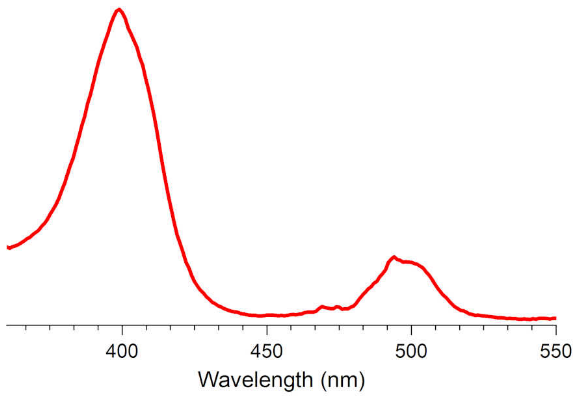

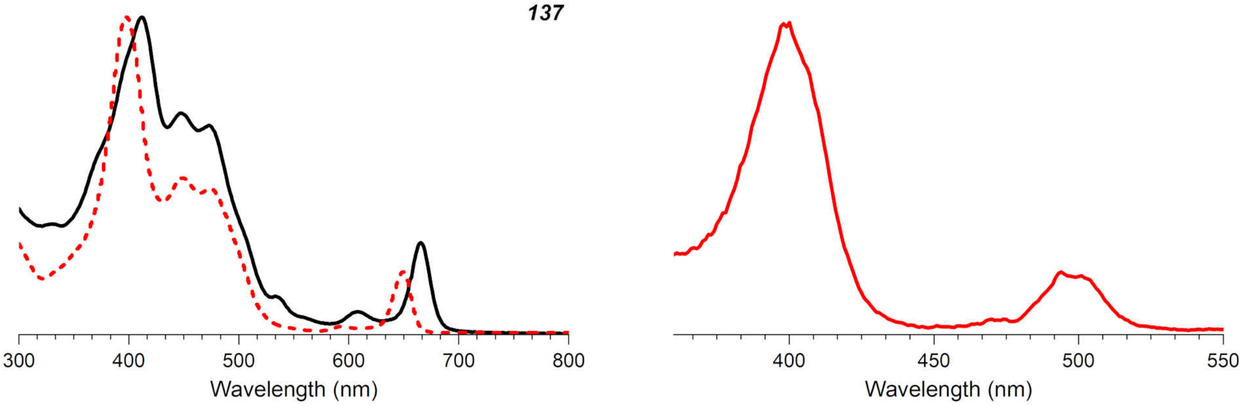
137
300
400
500
600
700
800
Wavelength (nm)
400
450
500
550
Wavelength (nm)

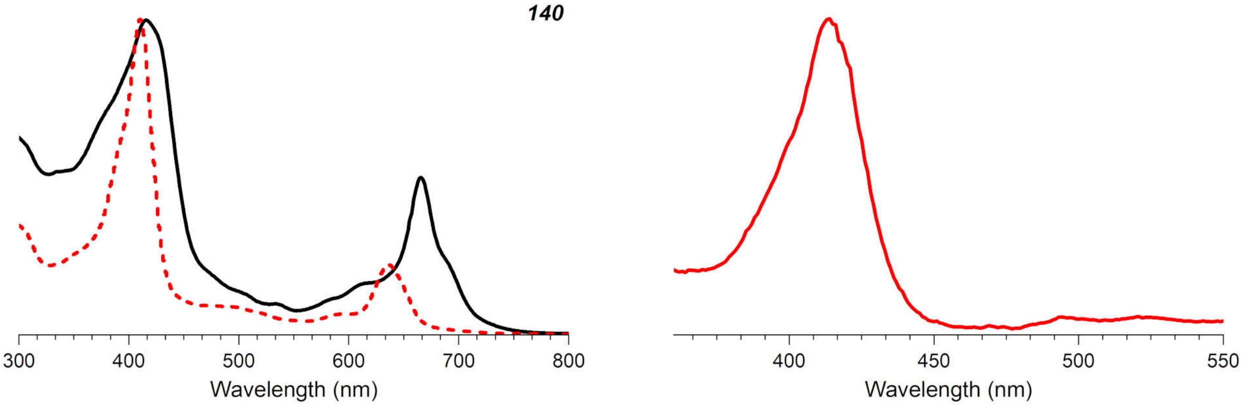
140
300
400
500
600
700
800
Wavelength (nm)
400
450
500
550
Wavelength (nm)

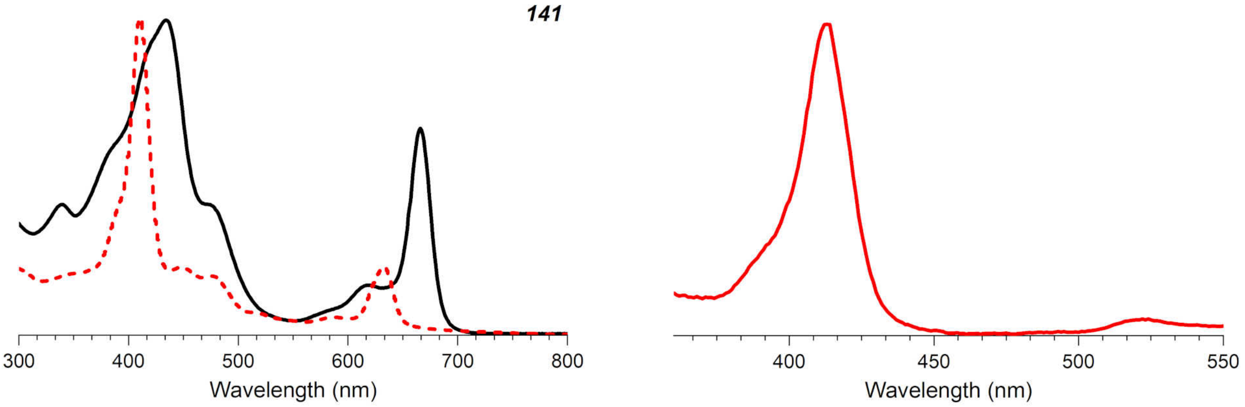
141
300
400
500
600
700
800
Wavelength (nm)
400
450
500
550
Wavelength (nm)

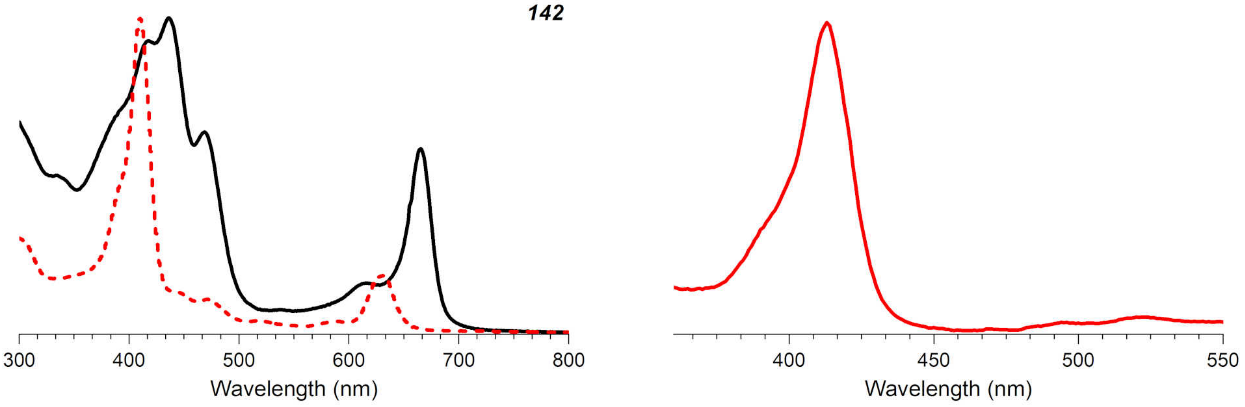
142
300
400
500
600
700
800
Wavelength (nm)
400
450
500
550
Wavelength (nm)

***143***

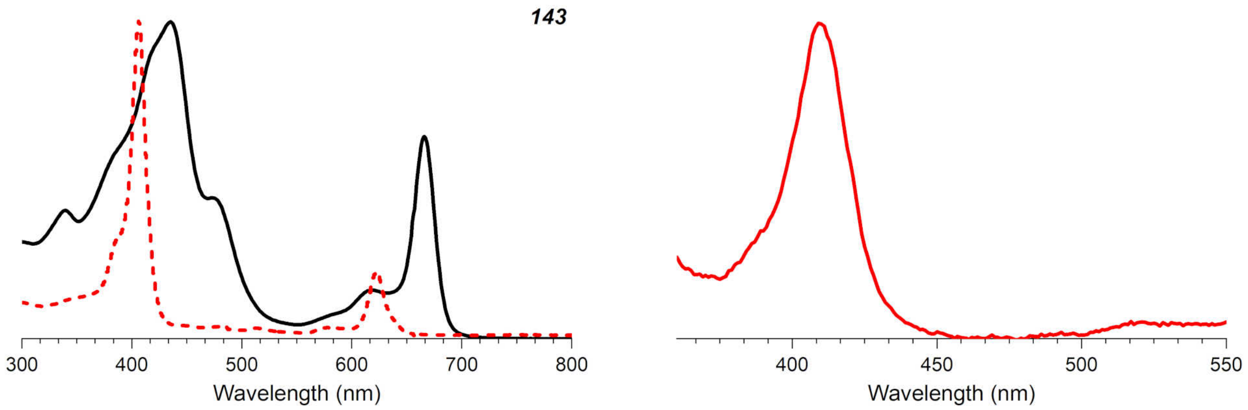


***144***

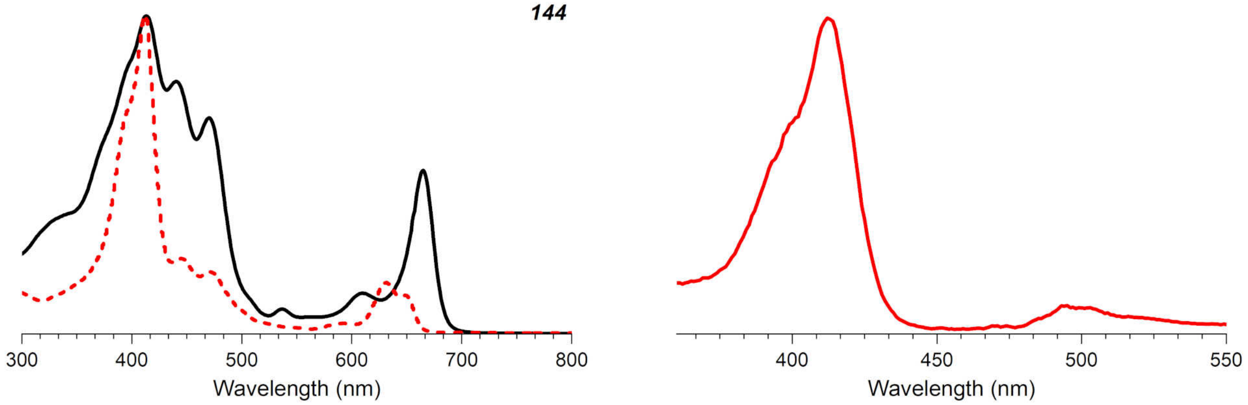


***145***

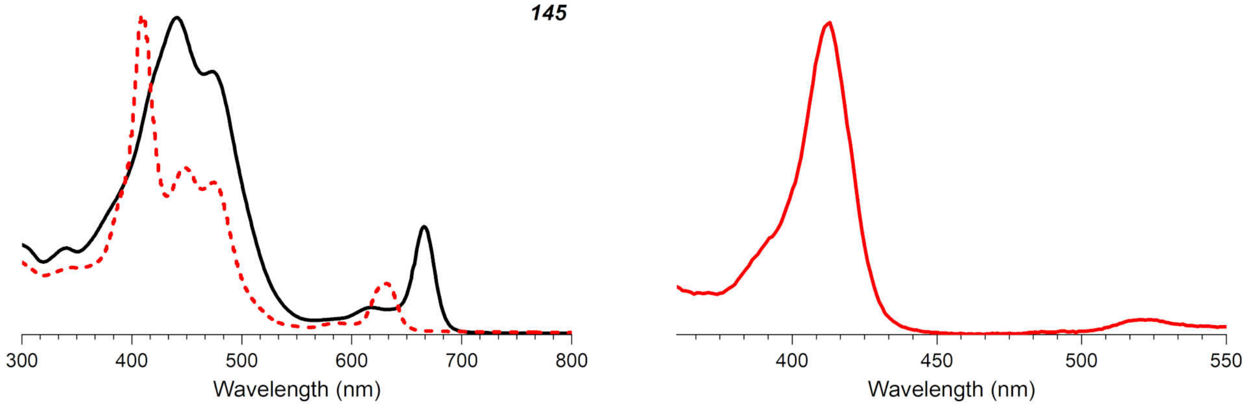


***146***

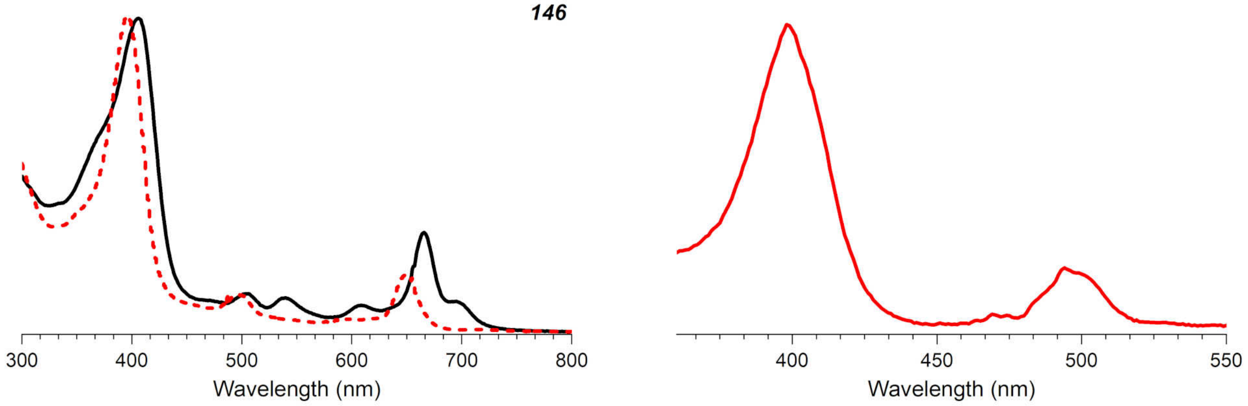

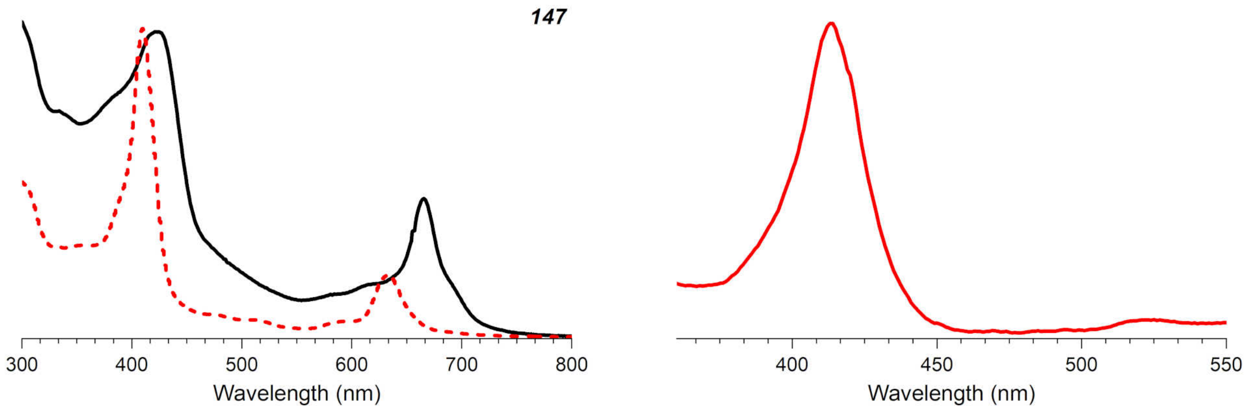
147
300
400
500
600
700
800
Wavelength (nm)
400
450
500
550
Wavelength (nm)

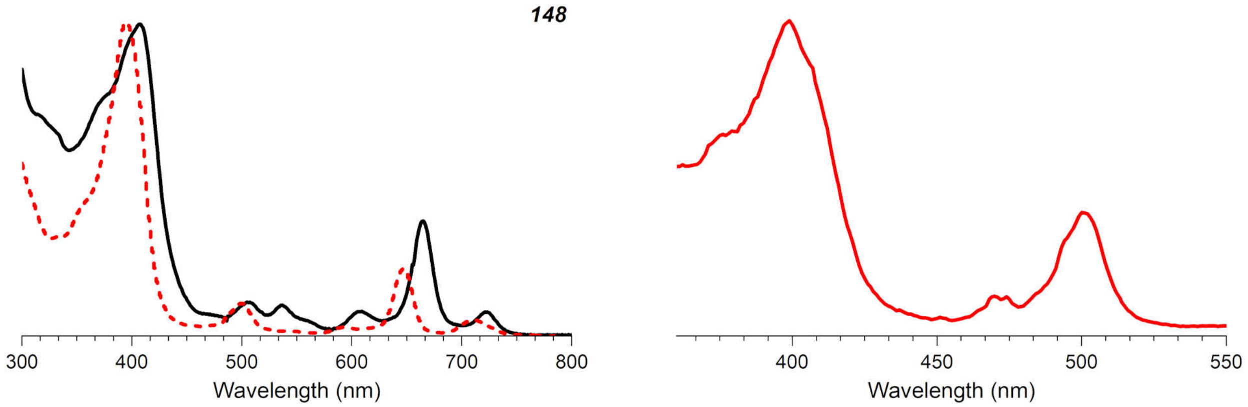
148
300
400
500
600
700
800
Wavelength (nm)
400
450
500
550
Wavelength (nm)

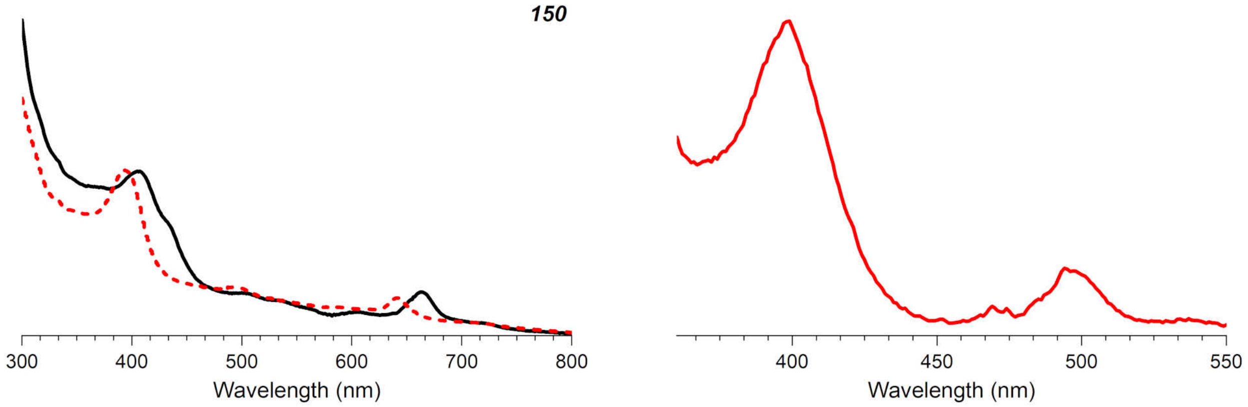
150
300
400
500
600
700
800
Wavelength (nm)
400
450
500
550
Wavelength (nm)

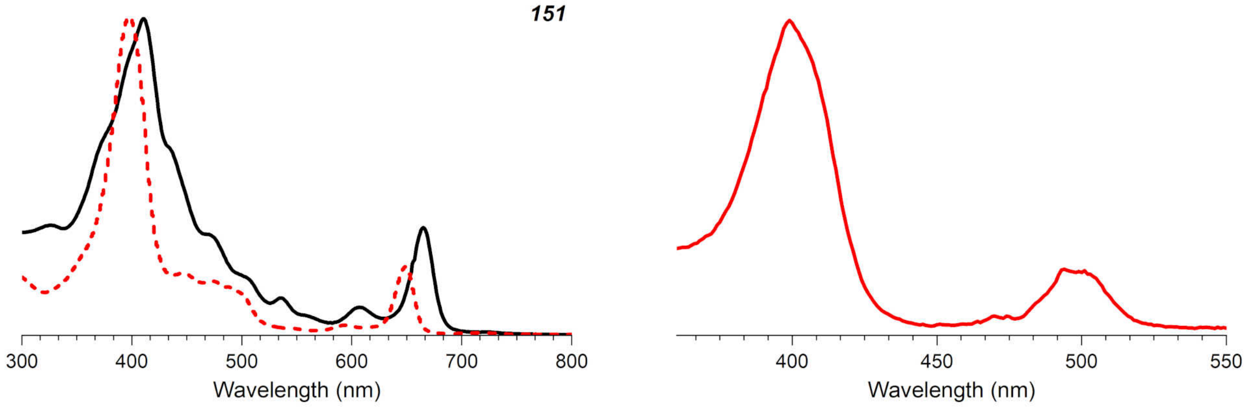
151
300
400
500
600
700
800
Wavelength (nm)
400
450
500
550
Wavelength (nm)

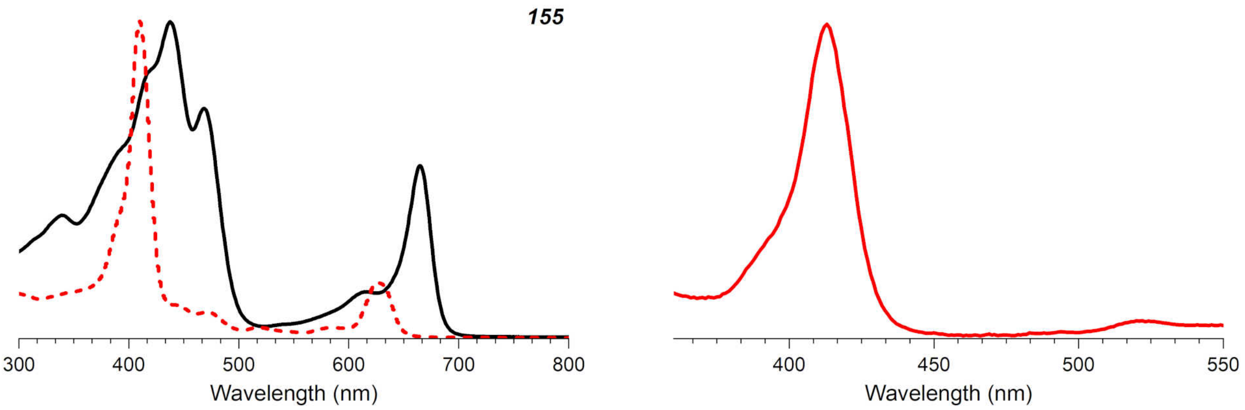
155
300
400
500
600
700
800
Wavelength (nm)
400
450
500
550
Wavelength (nm)

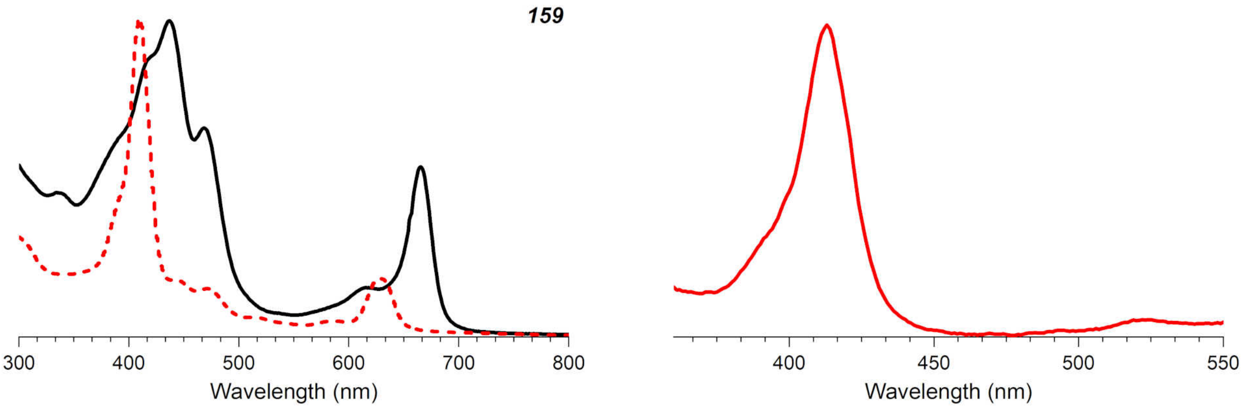
159
300
400
500
600
700
800
Wavelength (nm)
400
450
500
550
Wavelength (nm)

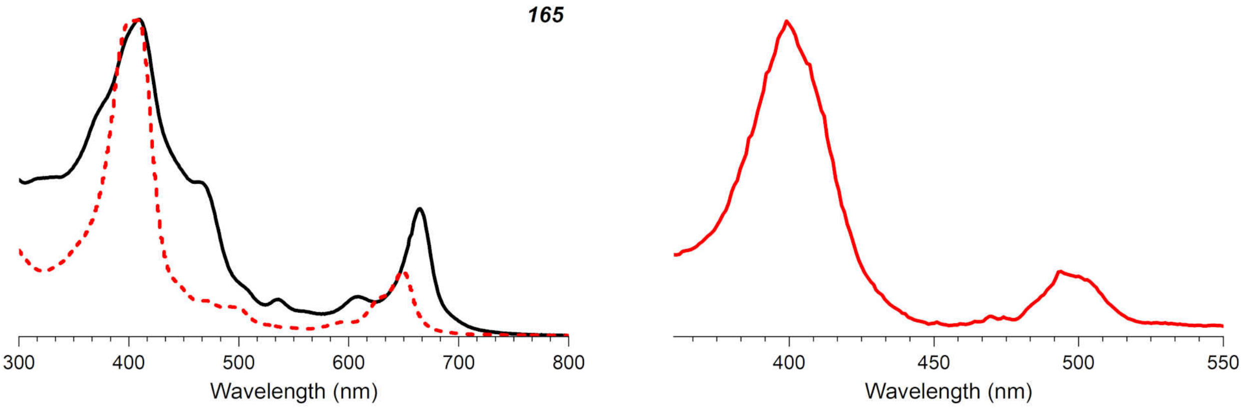
165
300
400
500
600
700
800
Wavelength (nm)
400
450
500
550
Wavelength (nm)

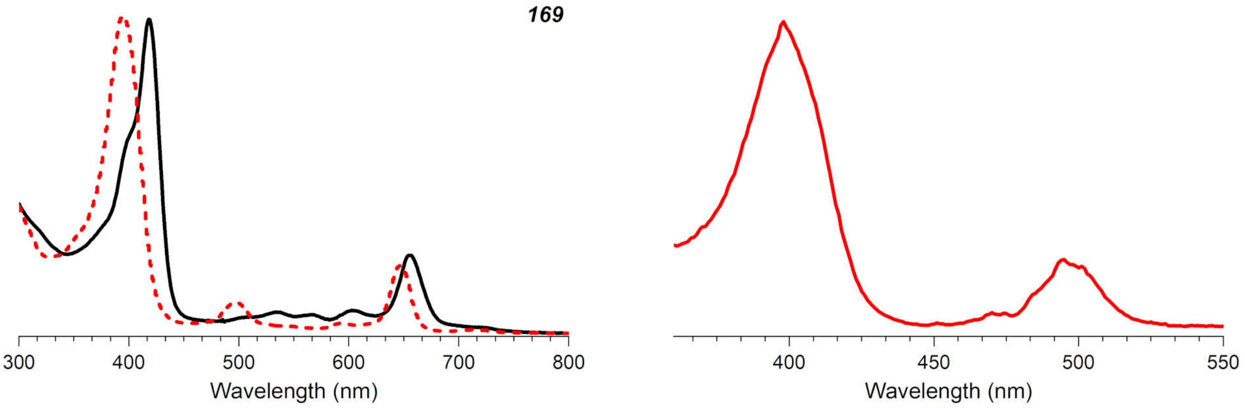
169
300
400
500
600
700
800
Wavelength (nm)
400
450
500
550
Wavelength (nm)

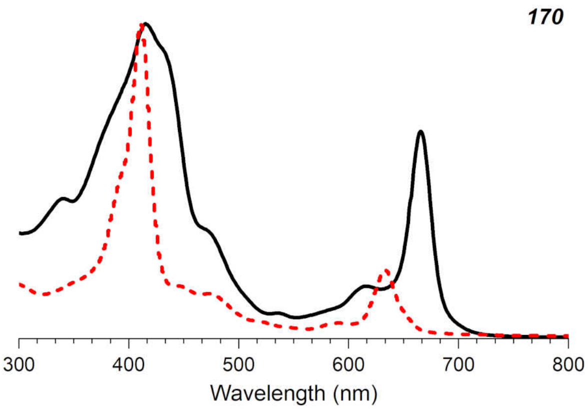
170
300
400
500
600
700
800
Wavelength (nm)

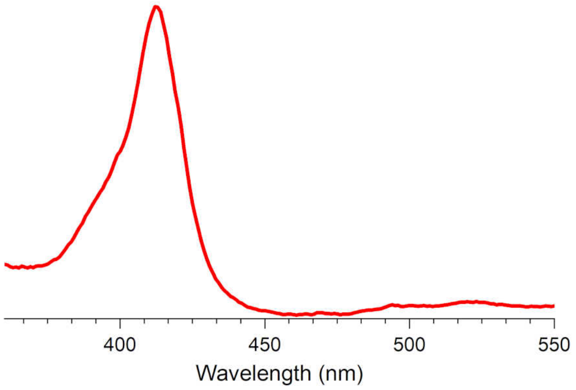
400
450
500
550
Wavelength (nm)

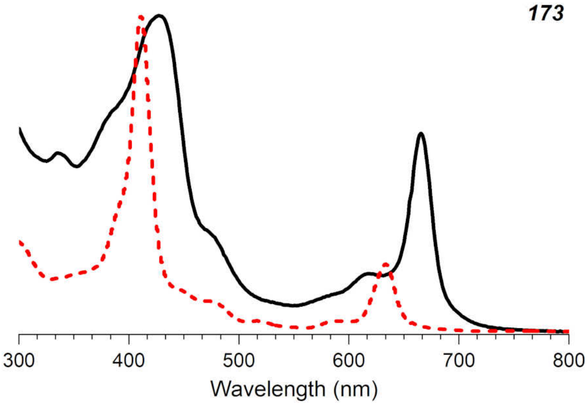
173
300
400
500
600
700
800
Wavelength (nm)

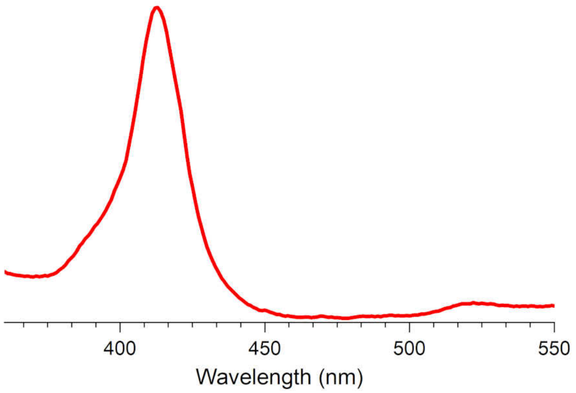
400
450
500
550
Wavelength (nm)

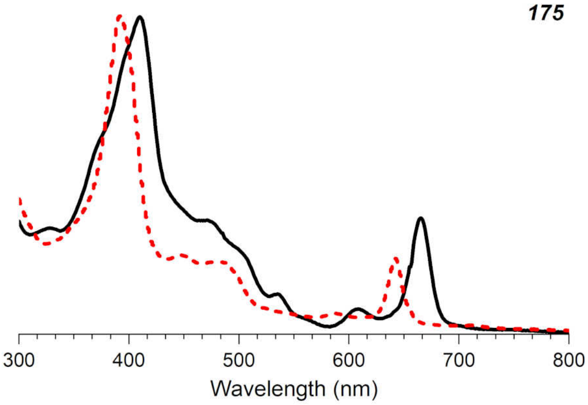
175
300
400
500
600
700
800
Wavelength (nm)

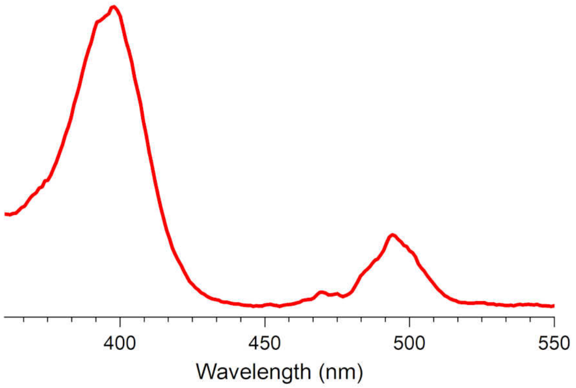
400
450
500
550
Wavelength (nm)

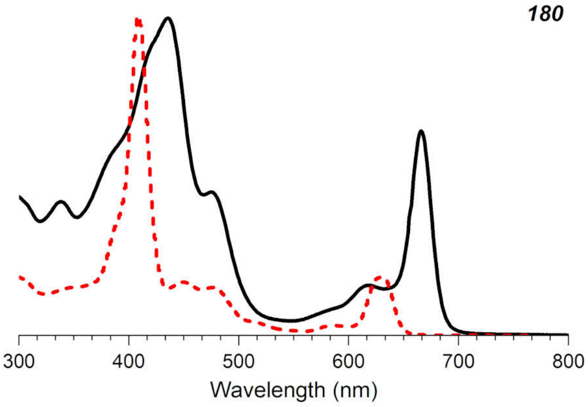
180
300
400
500
600
700
800
Wavelength (nm)

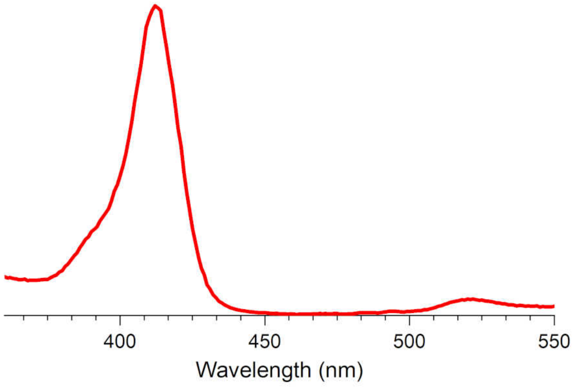
400
450
500
550
Wavelength (nm)

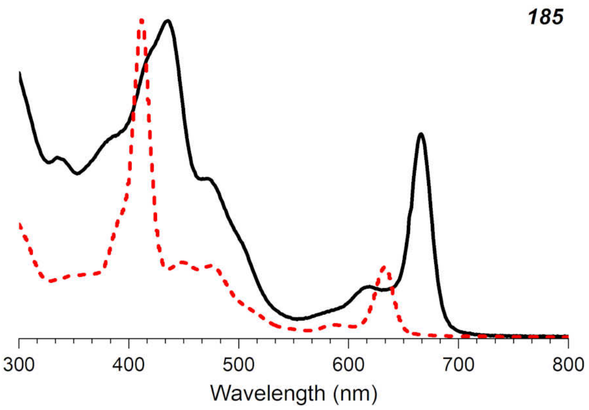
185
300
400
500
600
700
800
Wavelength (nm)

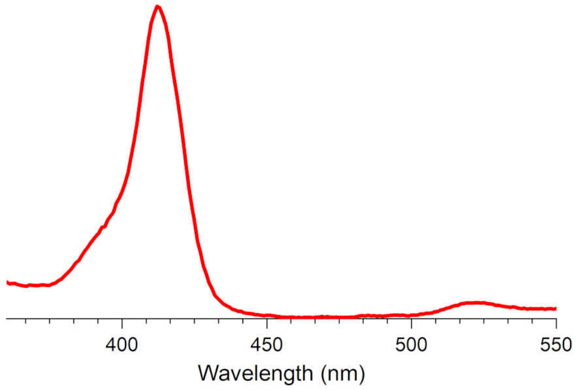
400
450
500
550
Wavelength (nm)

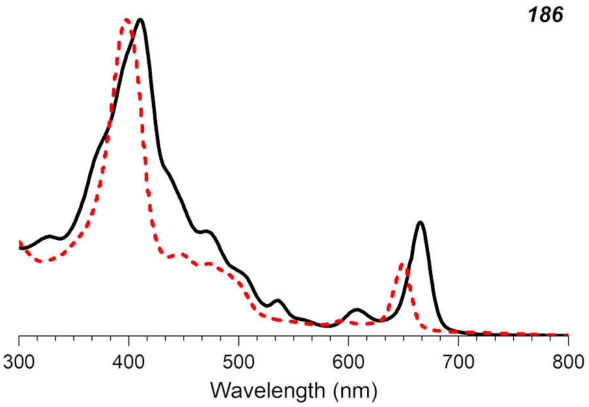
186
300
400
500
600
700
800
Wavelength (nm)

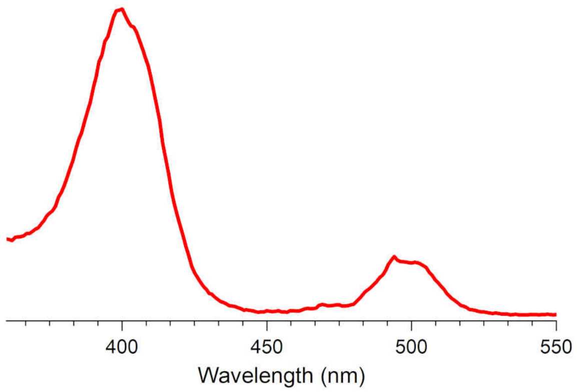
400
450
500
550
Wavelength (nm)

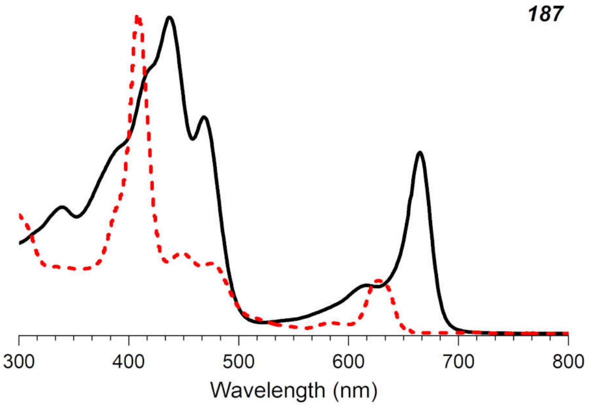
187
300
400
500
600
700
800
Wavelength (nm)

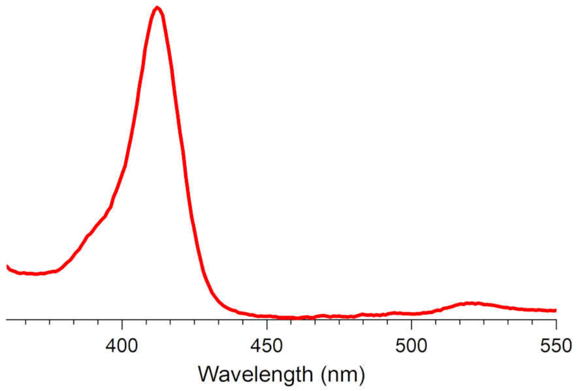
400
450
500
550
Wavelength (nm)

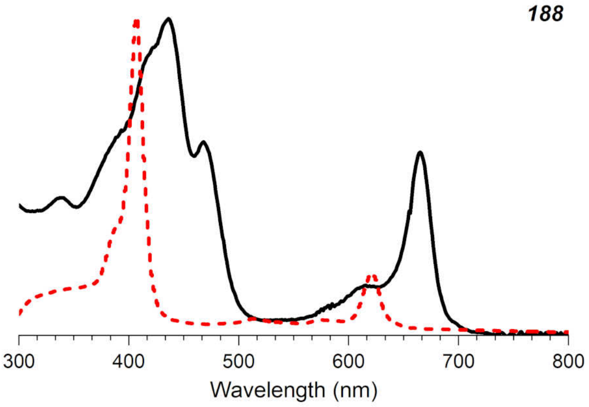
188
300
400
500
600
700
800
Wavelength (nm)

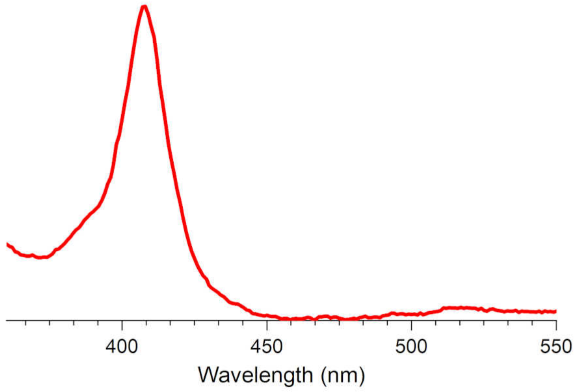
400
450
500
550
Wavelength (nm)

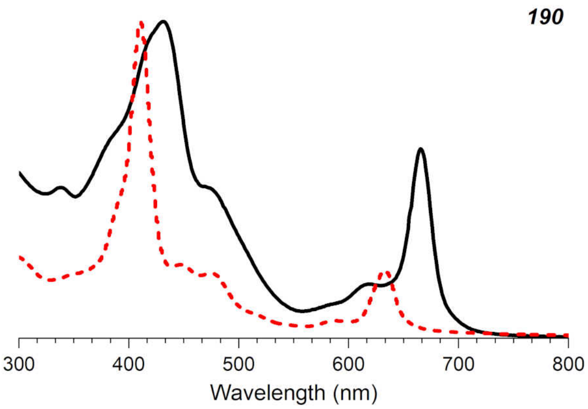
190
300
400
500
600
700
800
Wavelength (nm)

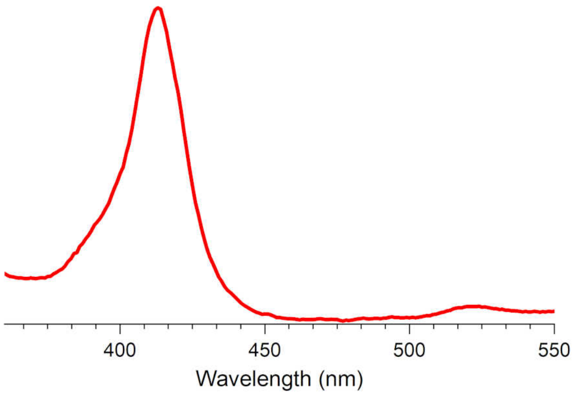
400
450
500
550
Wavelength (nm)

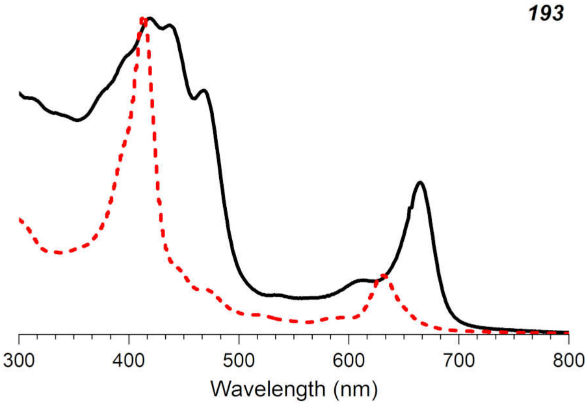
193
300
400
500
600
700
800
Wavelength (nm)

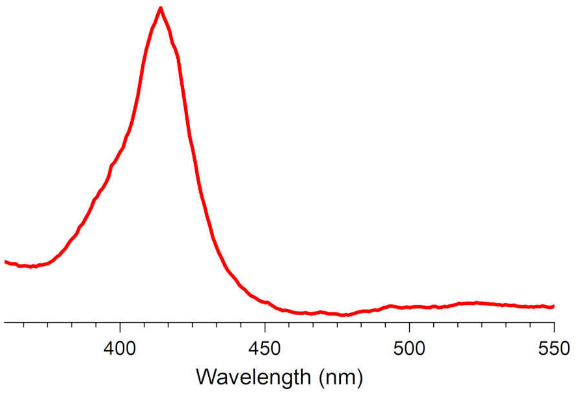
400
450
500
550
Wavelength (nm)

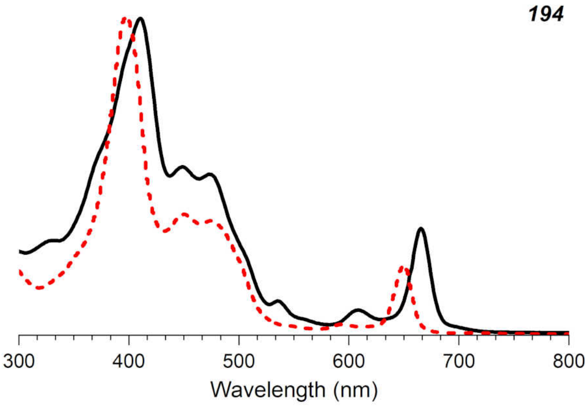
194
300
400
500
600
700
800
Wavelength (nm)

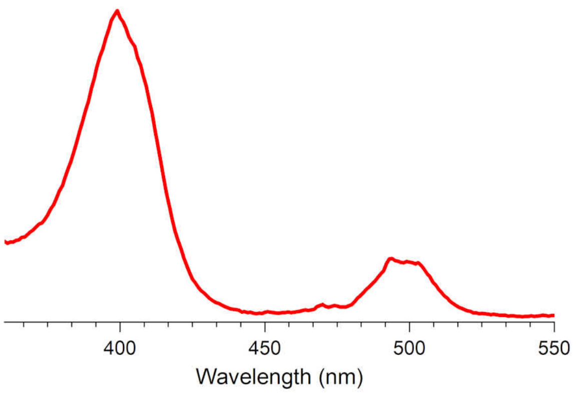
400
450
500
550
Wavelength (nm)

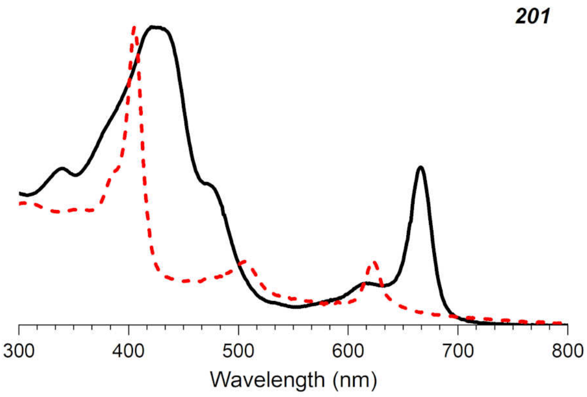
201
300
400
500
600
700
800
Wavelength (nm)

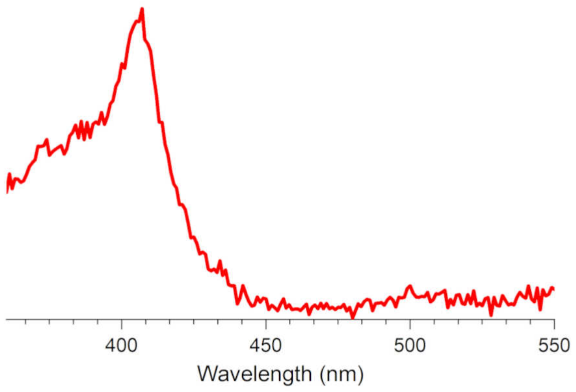
400
450
500
550
Wavelength (nm)

202

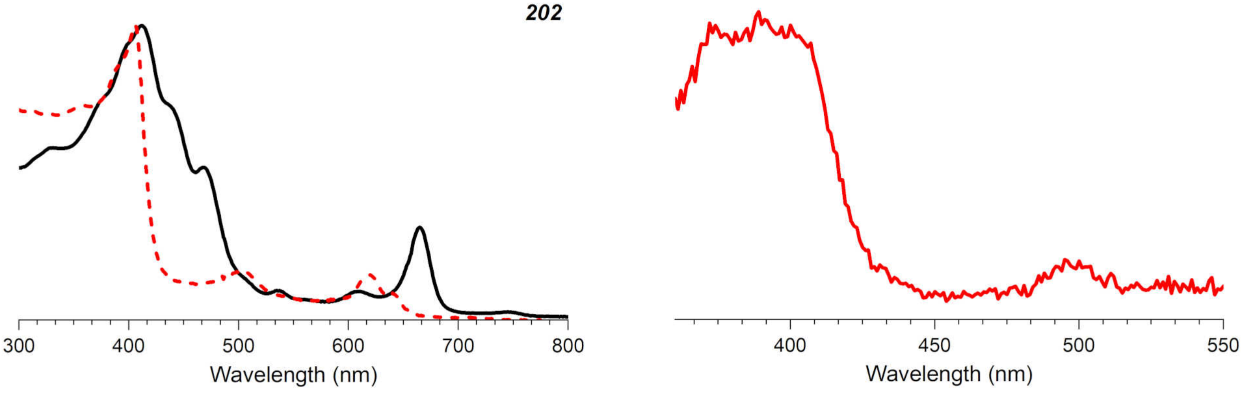


**S6. Isotopic labeling to probe tolyporphins biosynthesis**

One approach to establish whether tolyporphins are derived from the known pathway to tetrapyrroles, versus an alternative pathway, is provide a supply of isotopically labeled precursors, and examine the tolyporphins for incorporation of the corresponding isotopic labels. One tetrapyrrole molecule is derived from 8 molecules of ALA, where 2 molecules of ALA afford one molecule of porphobilinogen, and 4 molecules of porphobilinogen condense to form the tetrapyrrole. Hence, the presence of a single isotopic label in ALA, if the sole precursor, results in 8 isotopic labels in the tetrapyrrole. Upon use of a mixture of natural ALA and singly labeled ALA, the number of isotopic labels in the tetrapyrrole would be a statistical mixture of 0, 2, 4, 6, or 8 where the peak distribution depends on the ratio of the natural ALA and singly labeled ALA molecules. Convenient isotopically labeled precursors are (1,2-$^{13}C_2$) L-glutamic acid and (2-$^{13}$C) glycine for probing the C5 and C4 pathways, respectively (Figure S8). Isotopic feeding experiments to decipher the biosynthetic pathway leading to tolyporphins, even using advanced intermediates, are generally not workable.

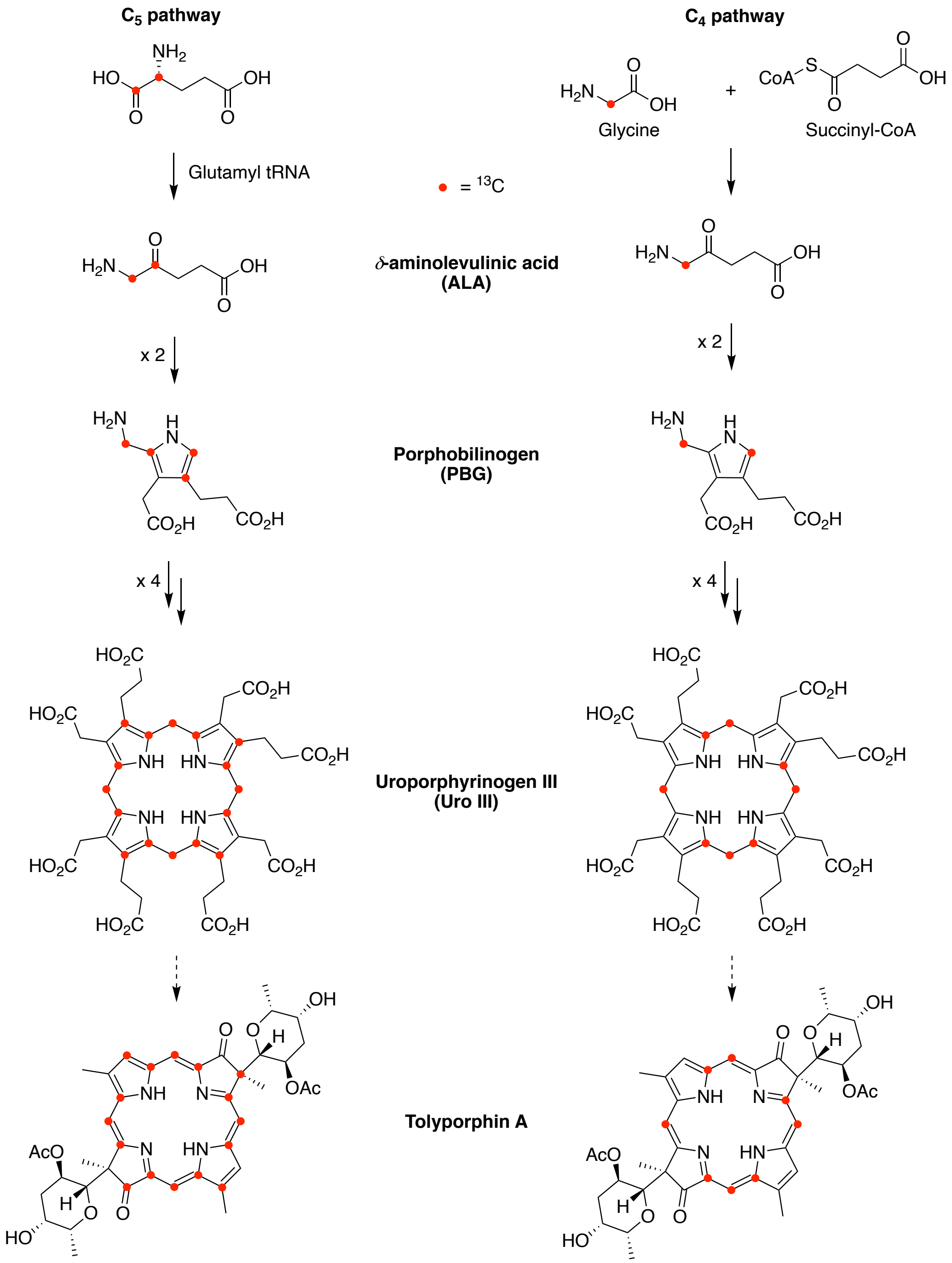


**Figure S8.** Isotopic labeling of ALA precursors and tolyporphin A downstream via two pathways.

The expected mass spectra based on the extent of incorporation of (1-$^{13}$C) glycine / (1,2-$^{13}C_2$) L-glutamic acid ($C_4$/$C_5$ pathway) are shown in Figure S9. The mass spectra are calculated using the binomial distribution, taking into account the natural abundance of $^{13}$C (1.11%) of natural (unlabeled) precursors. Even when the ratio of incorporation of isotopologue versus natural abundance precursor is as low as 10%, the isotopically labeled tolyporphin A is clearly distinguishable from the natural abundance tolyporphin A. The C4 pathway causes incorporation of 0, 2, 4, 6 or 8 isotopic labels, because the ALA precursor is composed of 0 or 1 isotopic labels. The C5 pathway causes incorporation of 0, 4, 8, 12, or 16 isotopic labels, because the ALA

precursor is composed of 0 or 2 isotopic labels. The binomial calculation is described in detail as follows.

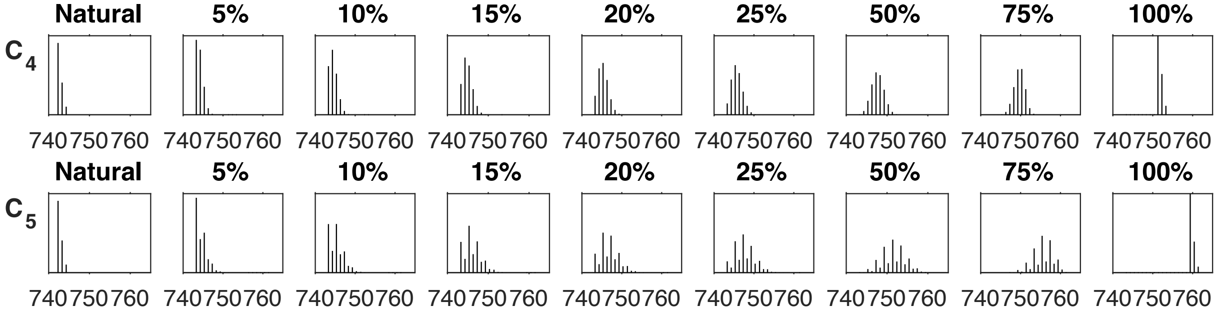


**Figure S9.** Calculated mass spectra of natural abundance tolyporphin A, and tolyporphin A labelled with 5–100% of the $^{13}C$ precursor [(1-$^{13}C$) glycine, (1,2-$^{13}C_2$) L-glutamic acid] via the two pathways.

**Binomial calculations.** For the C4 pathway, to calculate the isotopic pattern, the isotopic abundance of H was ignored. Considering natural abundance of $^{13}C$, the expected mass spectral isotopic pattern for tolyporphin A [$M^+$] is 100% 742.3, 44.8% 744.3 and 11.2% 744.3. Since a tolyporphin is composed of 8 glycine precursors, assume $k$ ($k \in \{0, 1, 2, 3, 4, 5, 6, 7, 8\}$) $^{13}C$ was introduced to tolyporphin A. The corresponding isotope distribution abundance $[M + k]^+$ is easily calculated by the Isotope Distribution Calculator and Mass Spec Plotter [221]; the results are shown in Table S5. The probability ($P_k$) of occurrence of $[M + k]^+$ was calculated by the binomial equation $P_k = \frac{n!}{k!(n-k)!} \times p^k \times (1-p)^{8-k}$ , in which $p$ is the probability of the incorporation of $^{13}C$ for every $^{12}C$ atom. The isotopic distribution pattern ($IDP$) was then calculated by $IDP = \sum_{k=0}^{8} P_k \times IDP_k$, in which $IDP_k$ is the isotopic distribution pattern of $[M + k]^+$. $IDP$ was represented by an array of mass peak abundances in the calculation. An example concluded $IDP$ in the case $p$ = 0.25 is shown in Table S5.

**Table S5.** Isotopic distribution pattern (represented by peak abundance) of $k$ $^{13}C$ incorporated tolyporphin A and an example distribution pattern of tolyporphin A (25% $^{12}C$ precursors were replaced by $^{13}C$ precursors).

| | 742.3 | 743.3 | 744.3 | 745.3 | 746.3 | 747.3 | 748.3 | 749.3 | 750.3 | 751.3 | 752.3 |
|---|---|---|---|---|---|---|---|---|---|---|---|
| $[M]^+$ | 100.0 | 44.8 | 11.2 | 0 | 0 | 0 | 0 | 0 | 0 | 0 | 0 |
| $[M+1]^+$ | 0 | 100.0 | 43.7 | 10.8 | 0 | 0 | 0 | 0 | 0 | 0 | 0 |
| $[M+2]^+$ | 0 | 0 | 100.0 | 42.6 | 10.3 | 0 | 0 | 0 | 0 | 0 | 0 |
| $[M+3]^+$ | 0 | 0 | 0 | 100.0 | 41.5 | 9.9 | 0 | 0 | 0 | 0 | 0 |
| $[M+4]^+$ | 0 | 0 | 0 | 0 | 100.0 | 40.4 | 9.5 | 0 | 0 | 0 | 0 |
| $[M+5]^+$ | 0 | 0 | 0 | 0 | 0 | 100.0 | 39.4 | 9.1 | 0 | 0 | 0 |
| $[M+6]^+$ | 0 | 0 | 0 | 0 | 0 | 0 | 100.0 | 38.3 | 8.7 | 0 | 0 |
| $[M+7]^+$ | 0 | 0 | 0 | 0 | 0 | 0 | 0 | 100.0 | 37.2 | 8.3 | 0 |
| $[M+8]^+$ | 0 | 0 | 0 | 0 | 0 | 0 | 0 | 0 | 100.0 | 36.1 | 7.9 |
| Example: $p$ = 0.25 | 22.8 | 71.0 | 100.0 | 84.0 | 46.6 | 17.9 | 4.8 | 0.9 | 0.1 | 0 | 0 |

For the C5 pathway, $k \in \{0, 2, 4, 6, 8, 10, 12, 14, 16\}$, and the same calculation method was applied. For $p \in \{0, 0.05, 0.10, 0.15, 0.25, 0.5, 0.75, 1\}$, the isotopic distribution pattern is shown in Figure S9. It warrants emphasis that the native tolyporphin A affords a mass envelope

composed of multiple (M, M+1, M+2, etc.) peaks as expected given the natural abundance of $^{13}C$. Similarly, growth with a precursor that is singly labeled or doubly labeled with $^{13}C$ also affords a mass envelope composed of multiple peaks.

**Isotopic feeding experiments.** To probe the pathway HT-58-2 applied for tolyporphins production, (1,2-$^{13}C_2$) L-glutamic acid (MW 149.12, 99%) and (2-$^{13}C$) glycine (MW 76.06, 98%) were purchased from Cambridge Isotope Laboratories (Tewksbury, MA) for the experiment of feeding HT-58-2 sample (Figure S10). HT-58-2 samples (30 days, N+ and N–, referring to BG-11 and BG-$11_0$, respectively) were transferred to two 24-well plates (N+ and N–, 2.5 mL media/well, <0.1 mg dry cells/well) to grow under continuous white light at 28 °C. BG-11 medium (~ 0.5 mL) was added to both cultures (previous N+ and N–) every 3 days to maintain the volume of the cultures. After 15 days (according to the growth curve and tolyporphins production curves consistent with the results of a nitrogen stimulation experiment in ref [69]), the isotopic precursors (10 µM) were added. Cells were harvested at the 50$^{th}$ day and the pigments were extracted by reported methods [79] to assay the MALDI-MS signals of tolyporphins. The experimental timeline is shown in Figure S10.

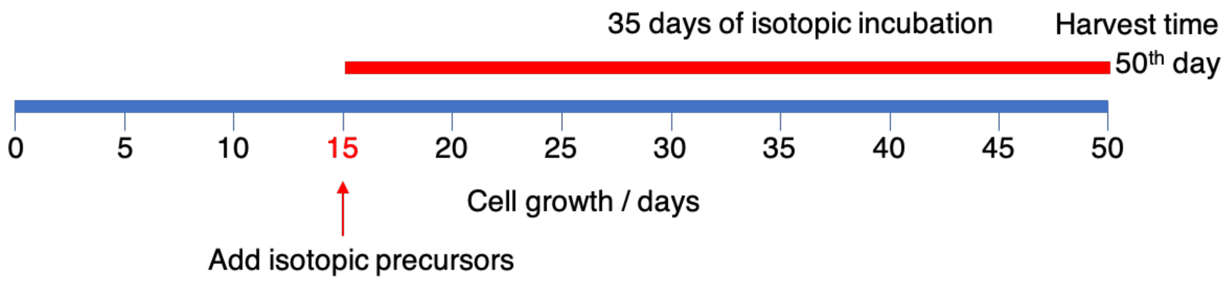


**Figure S10.** Timeline of the isotopic feeding experiment.

After harvesting and lipophilic extraction, the HT-58-2 extract in $CH_2Cl_2$/MeOH (v/v = 1/1) was screened by MALDI-TOF-MS. The control samples show the expected monoisotopic mass M as well as the M+1 and M+2 peaks in the respective mass envelopes of tolyporphin A ($m/z$ = 744) and chlorophyll *a* ($m/z$ = 894) due to incorporation of natural abundance isotopes $^{13}C$, $^{2}H$, and $^{15}N$. By comparison with control samples lacking any isotopic labels, more peaks due to isotopic incorporation in tolyporphin A and chlorophyll *a* were detected for both +Gly* and +Glu* samples, indicating that $^{13}C$ was introduced to the tetrapyrrole products. But the mass peaks occur at 1 Da intervals, whereas for direct incorporation of the isotopically labeled precursors +Gly* and +Glu*, the peaks would occur at 2 or 4 Da intervals, respectfully. Accordingly, the resulting peak distributions have no unambiguous binomial distribution, as required to conclude a C4 or C5 pathway, according to the calculated patterns (Figure S11).

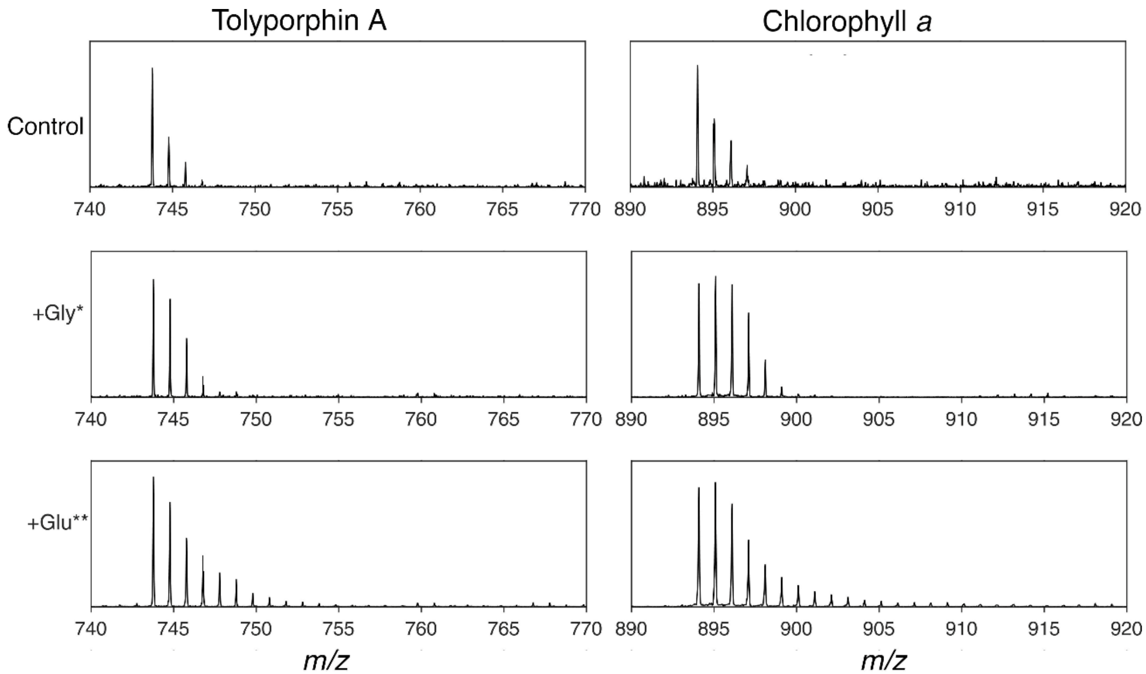

**Figure S11.** MALDI-TOF-MS results of the lipophilic extracts of isotopic fed HT-58-2 samples.

Ambiguous results by definition have multiple interpretations. One interpretation is that the precursors, (1-$^{13}C$) glycine and (1,2-$^{13}C_2$) L-glutamic acid, are degraded more rapidly than incorporated into the tolyporphins biosynthetic pathway. If so, $^{13}C$ carbons become incorporated as single carbon entities, leading to the observed mass spectral patterns. Hence, the results are insufficient to conclude a C4 or C5 pathway via ALA, or an alternative pathway altogether, leading to tolyporphins. Regardless, beyond the goal of delineating such pathways was the objective of probing more deeply the biosynthesis of tolyporphins.
